# Molecular evolution of *Wcor15* gene enhanced our understanding of the origin of A, B and D genomes in *Triticum aestivum*

**DOI:** 10.1038/srep31706

**Published:** 2016-08-16

**Authors:** Fangfang Liu, Hongqi Si, Chengcheng Wang, Genlou Sun, Erting Zhou, Can Chen, Chuanxi Ma

**Affiliations:** 1 School of Agronomy, Anhui Agricultural University, Hefei 230036, China; 2 Key Laboratory of Wheat Biology and Genetic Improvement on South Yellow & Huai River Valley, Ministry of Agriculture, Hefei 230036, China; 3 Biology Department, Saint Mary’s University, Halifax, NS, B3H 3C3 Canada; 4 National United Engineering Laboratory for Crop Stress Resistance Breeding, Hefei 230036, China; 5 Anhui Key Laboratory of Crop Biology, Hefei 230036, China

## Abstract

The allohexaploid bread wheat originally derived from three closely related species with A, B and D genome. Although numerous studies were performed to elucidate its origin and phylogeny, no consensus conclusion has reached. In this study, we cloned and sequenced the genes *Wcor15-2A, Wcor15-2B* and *Wcor15-2D* in 23 diploid, 10 tetraploid and 106 hexaploid wheat varieties and analyzed their molecular evolution to reveal the origin of the A, B and D genome in *Triticum aestivum*. Comparative analyses of sequences in diploid, tetraploid and hexaploid wheats suggest that *T. urartu, Ae. speltoides* and *Ae. tauschii* subsp. *strangulata* are most likely the donors of the *Wcor15-2A, Wcor15-2B* and *Wcor15-2D* locus in common wheat, respectively. The *Wcor15* genes from subgenomes A and D were very conservative without insertion and deletion of bases during evolution of diploid, tetraploid and hexaploid. Non-coding region of *Wcor15-2B* gene from B genome might mutate during the first polyploidization from *Ae. speltoides* to tetraploid wheat, however, no change has occurred for this gene during the second allopolyploidization from tetraploid to hexaploid. Comparison of the *Wcor15* gene shed light on understanding of the origin of the A, B and D genome of common wheat.

Wheat (*Triticum aestivum* L.) is an annual species in the tribe *Triticeae* of the grass family *Poaceae*. It is the most widely cultivated food crop followed by rice and maize, and is the primary cereal in the temperate region, serving as a staple food for about 40% of the world’s population (http://faostat.fao.org)^1^. Common wheat is one of the earliest domesticated crop plants in the Pre-Pottery Neolithic Near East^2^,^3^.

Polyploidization played an important role in the evolution of eukaryotes, and is one of the important mechanisms for creating genetic variation, and major evolutionary factor affecting genome size and gene copy number^4^,^5^,^6^,^7^. Polyploids can be formed via the duplication of genomes, either of the same genomes (autopolyploid) or of diverged genomes with homoeologous relationships (allopolyploid)^8^,^9^. *Triticum aestivum* (AABBDD) as a good example of allopolyploid is derived from the three homologous genomes, A, B, and D, each of which contributes 7 pairs of chromosomes to the wheat’s total genome (2n = 6x = 42)^10^ with an approximate genome size of 16–17 Gb^11^,^12^,^13^. It was suggested that the origin of allohexaploid wheat (*Triticum aestivum* L.) involved two sequential allopolyploidization events^14^,^15^. The first wheat allopolyploidization involved diploid AA genome species and diploid BB species to form tetraploid AABB approximately 0.36 to 0.5 million years ago^16^,^17^. The second polyploidization between diploid goat grass species (DD, *Aegilops tauschii* Coss) and the tetraploid (AABB) emmer wheat (closely related to *Triticum turgidum* subsp. *durum*, genome AABB) led to the formation of common wheat (AABBDD) approximately 8,000 years ago^16^,^18^.

The progenitor of the A genome of the tetraploid and hexaploid wheat species contains *Triticum urartu* Thum ex Gand (genome A^u^)^19^ and *Triticum monococcum* Linn (genome A^m^) including two subspecies: the wild *T. monococcum* subsp*. boeoticum* Boiss. (*T. m. boeoticum*)^20^ and its domesticated form *T. monococcum* subsp*. monococcum (T. m. monococcum*)^21^. The A^u^ and A^m^ genomes have similar genome size and gene content^22^. *T. urartu*, the wild diploid wheat from the Fertile Crescent region, has long been considered as the A-genome donor to tetraploid and hexaploid wheat species^23^,^24^. In polyploid wheat, the origin of the B genome is still under debating, in spite of a large number of attempts to identify the parental species^24^. It has been reported that the B genome is closely related to the S genome of the *Sitopsis* section^25^,^26^,^27^ which contains five species: *Ae. bicornis* (S^b^S^b^, 2n = 2x = 14), *Ae. longissima* (S^l^S^l^, 2n = 2x = 14), *Ae. sharonensis* (S^sh^S^sh^, 2n = 2x = 14), *Ae. searsii* (S^s^S^s^, 2n = 2x = 14) and *Ae. speltoides* (SS, 2n = 2x = 14)^28^,^29^. Previous studies^30^,^31^,^32^,^33^ have shown that *Ae. speltoides* is phylogenetically distinct from the other species in the *Sitopsis* section. *Ae. speltoides* (S genome) has been suggested as the most likely progenitor of the B genome^26^,^27^. However, Huang *et al*.^24^ and Haider^29^ reported that none of the five *Sitopsis* species they investigated is a close relative of the B genome in *T. aestivum*, and concluded that the B genome donor remains unknown. There has been little debate on *Ae. tauschii* Coss (genome DD) as the D genome progenitor of *T. aestivum*^24^.

There has been great interest in the determination of ancestral diploid genome donors of hexaploid wheat^10^,^29^. Understanding the origin of hexaploid wheat not only enhances its genetic improvement, but also is important in the development of artificial synthetic forms^20^,^34^, because genome progenitors of common wheat are very important genetic resources to improve the economical traits of modern cultivars^35^,^36^. However, so far, the direct experimental evidence for clear understanding of the phylogenetic history among the three A, B, and D genome lineages are still challenging. Maybe, this debate can be greatly simplified by analyzing the molecular evolution of a conservative gene among diploid, tetraploid and hexaploid wheat species.

*Wcor15* (GenBank: AB095006), a member of the wheat cold-responsive gene family, which could encode the chloroplast-targeted protein when exposed to low temperature, plays an important role in the cold hardiness of wheat^37^. Based on our sequencing data, we found that the *Wcor15* gene was very conservative in the hexaploid wheat, not only the coding region but also the 5′-upstream non-coding region. In this study, we cloned and sequenced the *Wcor15* gene from diploid, tetraploid and hexaploid wheats to reveal the origin of A, B and D genome in common wheat, and compared their evolution among diploid, tetraploid and hexaploid wheats.

## Results

### Cloning and characterization of homoeologous *Wcor15* genes

The three homoeologous *Wcor15* sequences were identified using the ORF sequence (including the intron, 563 bp) of *Wcor15* gene (GenBank: AB095006) as probe to screen the nucleotides databases of EBI (EBI; http://www.ebi.ac.uk/ena/)^38^, and sequences were found from the wheat genome A, B and D, respectively (Table 1). The specific PCR primers named Wcor15A, Wcor15B and Wcor15D (Table 2) for amplifying three homoeologous *Wcor15* sequences which contained intact ORFs were designed, based on the highly variation region of accession CBTL0110083500 (2AL), CBTL0111257031 (2BL) and CBTL0110522649 (2DL).

The primer pairs were used to amplify genomic DNA of hexaploid wheat cultivar Annong 0822. Each primer pair generated single-band amplicon with the expected size. The genes were designated as *Wcor15-2A* (KT264885), *Wcor15-2B* (KT264957) and *Wcor15-2D* (KT265022) respectively, which contained the 5′ upstream region, two exons, one intron and 3′ downstream region. Further analysis demonstrated that these three sequences are very similar with a few nucleotide insertions, deletions, and substitutions (Supplementary Fig. S1). The *Wcor15-2A* sequence from A genome is exactly the same to the sequence of AB095006 previously reported by Takumi *et al*.^37^, suggesting that the *Wcor15-2A* and *Wcor15* (GenBank: AB095006) is the same gene. After RT-PCR using RNA templates from Annong 0822, all of the three homoeologous *Wcor15* genes were specifically induced by low temperature (data not shown), suggesting the three homoeologous *Wcor15* genes are the cold-responsive gene.

**Figure 1:**
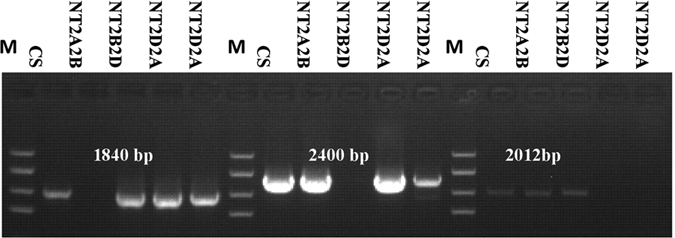
PCR amplification of CS homoeologous group 2 nulli-tetrasomic lines with the genome-specific primer sets Wcor15A, Wcor15B and Wcor15D. Location in a particular chromosome is indicated by absence. M: Marker.

In order to further confirm the location of the gene, one set of nulli-tetrasomic lines of cv. Chinese Spring was used. *Wcor15-2B* was found in the lines except nullisomic 2B–tetrasomic 2D (N2B–T2D). This indicates that the *Wcor15-2B* is located on chromosome 2B. In turn, *Wcor15-2A* and *Wcor15-2D* were assigned to chromosome 2A, and 2D, respectively (Fig. 1).

**Figure 2:**
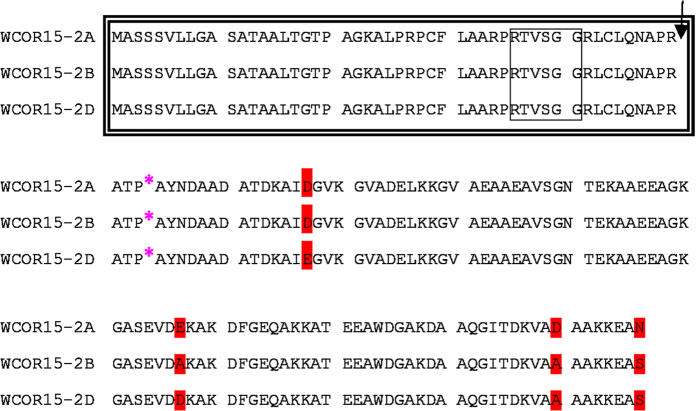
Alignment of the amino acid sequences. Diverse amino acids are indicated by red shade. Boxes with a double and a single line show the conserved region coding for the putative chloroplast signal peptides and a 14-3-3 protein recognition motif, respectively. The arrow indicated the putative cleavage site of the signal peptide determined with ChloroP. The site of an intron insertion is indicated by a pink asterisk.

Each *Wcor15* cDNA clone contained an ORF of 441 nucleotides that putatively encoded a polypeptide with 147 amino acid residues (Fig. 2). They shared common characteristics such as a sorting signal that is predicted to target them to the chloroplast^37^. The properties of the N-terminal end of the Wcor15-2A, Wcor15-2B and Wcor15-2D polypeptides were determined. They have the conserved regions coding for the putative chloroplast signal peptides and the putative cleavage site of the signal peptide (Fig. 2), and shared the common site of an intron insertion and 14-3-3 protein recognition motif that could interact with the 14-3-3 proteins. The binding of the proteins to the signal peptides is essential for the chloroplast precursor proteins to be efficiently transported into chloroplasts^39^,^40^. We also uncovered evidence that WCOR15-2A, WCOR15-2B and WCOR15-2D contained 11-mer amino acid motifs and α-helix structures characterizing LEA Group3^41^. Together these findings suggested that WCOR15-2A, WCOR15-2B and WCOR15-2D might belong to the chloroplast-targeted LEA3 protein.

### Sequence analysis of the *Wcor15-2A, Wcor15-2B* and *Wcor15-2D* genes in hexaploid wheats (AABBDD, *T. aestivum* and *T. spelta*)

The Wcor15A primer was used to amplify the *Wcor15-2A* among individual 106 hexaploid wheats including winter wheats, spring wheats and *T. spelta* from different geographical regions (Table 3). All the studied hexaploid wheats yielded an expected PCR product of approximately 1.8 kb. To further analyze *Wcor15-2A*, we randomly sequenced 100 samples (Supplementary Table S1). All sequences were identical and were exactly same to the *Wcor15-2A* sequence of Annong 0822 (Supplementary Table S2), suggesting that *Wcor15-2A* gene was highly conservative in hexaploid wheat.

The complete sequence of *Wcor15-2B* gene was also amplified from these 106 hexaploid wheats using Wcor15B primer. The PCR products from 54 wheats were sequenced (Supplementary Table S1). The *Wcor15-2B* sequences were highly conserved in the 54 hexaploid wheats (Supplementary Table S3). Fifteen substitutions (13 in the 5′ upstream, 2 in the 3′ downstream) and 2 insertion and deletion (one in the 5′ upstream, another in the intron) were occurred in the untranslational region, however, no significant differences were found in the two exons among the 54 sequences of *Wcor15-2B* (Supplementary Fig. S2). They shared 100% identities in the deduced amino acid sequences.

The *Wcor15-2D* in these 106 hexaploid wheat accessions was also characterized. All of the samples yielded PCR products of ~2 kb. The PCR products from 33 wheat varieties were sequenced (Supplementary Table S1). No variation was found among 33 hexaploid wheat varieties (Supplementary Table S4), indicating highly conservative of *Wcor15-2D* gene in hexaploid wheat.

Our results indicated that the three genes *Wcor15-2A, Wcor15-2B* and *Wcor15-2D* derived from the three homoeologous 2A, 2B and 2D chromosomes were highly conserved among hexaploid wheat varieties from different geographical regions.

### Sequence analysis of the *Wcor15-2A, Wcor15-2B* and *Wcor15-2D* genes in tetraploid species (AABB)

**Figure 3:**
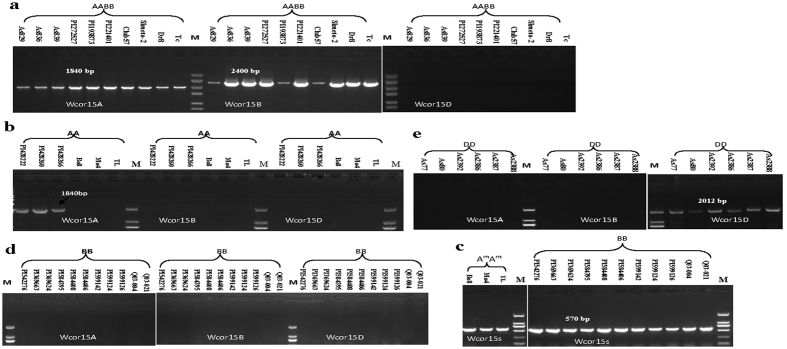
PCR amplification of the Wcor15A Wcor15B and Wcor15D primers in diploid and tetraploid accessions. (**a**) PCR amplification with Wcor15A Wcor15B and Wcor15D primers in tetraploid species. As829, As836 and As839 belong to *T. dicoccoides.* PI272527, PI193873 and PI221401 belong to *T. dicoccum.* Club57, Simeto-2 and Dr8 belong to *T. durum.* Tc belongs to *T. carthlicum.* (**b**) PCR amplification with Wcor15A Wcor15B and Wcor15D primers in *T. urartu, T. monococcum* and *T. boeoticum*. PI428222, PI428260 and PI428266 belong to *T. urartu.* Bo8 belongs to *T. boeoticum.* Mo4 and TL belong to *T. monococcum.* (**c**) PCR amplification with Wcor15s primers in *T. monococcum* and *T. boeoticum* and eleven species of the *Sitopsis* section. Bo8 belongs to *T. boeoticum.* Mo4 and TL belong to *T. monococcum.* PI542276, PI369663 and PI369624 belong to *Ae. speltoides.* Q03-004 belongs to *Ae. longissima.* Q03-021 belongs to *Ae. bicornis.* PI584395, PI584408 and PI584406 belong to *Ae.sharonensis.* PI599142, PI599124 and PI599126 belong to *Ae.searsii.* (**d**) PCR amplification with Wcor15A Wcor15B and Wcor15D primers in eleven species of the *Sitopsis* section. PI542276, PI369663 and PI369624 belong to *Ae. speltoides.* Q03-004 belongs to *Ae. longissima.* Q03-021 belongs to *Ae. bicornis.* PI584395, PI584408 and PI584406 belong to *Ae.sharonensis.* PI599142, PI599124 and PI599126 belong to *Ae.searsii.* (**e**) PCR amplification with Wcor15A Wcor15B and Wcor15D primers in *Ae. tauschii* species. As77, As80 and As2392 belong to *Ae. ssp. tauschii.* As2386, As2387 and As2388 belong to *Ae. ssp. strangulata.*

The DNA from 10 tetraploid materials including three *T. dicoccoides*, three *T. dicoccum*, three *T. durum* and one *T. carthlicum* (Table 3) were amplified using the primer pairs Wcor15A, Wcor15B and Wcor15D (Table 2). As expected, only the Wcor15A and Wcor15B amplified the PCR products with expected size (Fig. 3a). The Wcor15D primer did not give rise to any amplification products (Fig. 3a), confirming absence of *Wcor15-2D* in the tetraploid wheat genome.

The *Wcor15-2A* sequences from A genome in 10 tetraploid species (AABB) (Table 4) are exactly the same with the sequence of *Wcor15-2A* from hexaploid wheats (Supplementary Table S5), suggesting that *Wcor15-2A* gene is highly conserved within tetraploid wheats, and between tetraploid and hexaploid wheats.

Alignment of the 10 *Wcor15-2B* sequences from tetraploid wheat showed a number of single nucleotide substitutions among these sequences whose situation was the same to *Wcor15-2B* in the 54 hexaploid varieties (Supplementary Fig. S2), suggesting that diversification of *Wcor15-2B* did not occur between tetraploids and hexaploids during and after the second polyploidization.

### Sequence analysis of the *Wcor15-2A, Wcor15-2B* and *Wcor15-2D* genes in diploid species (AA, SS and DD)

In order to compare if *Wcor15-2A, Wcor15-2B* and *Wcor15-2D* genes have changed between diploid and polyploid, we sequenced these genes in a set of diploid wild relatives with genome AA, SS and DD, respectively (Table 4).

In all the three varieties of *T. urartu* (genome A^u^A^u^) surveyed, the primer Wcor15B and Wcor15D did not generate any amplification products (Fig. 3b), suggesting that the *Wcor15-2B* and *Wcor15-2D* sequence is absent in *T. urartu*. Amplicons were obtained from all three *T. urartu* with the primer Wcor15A. The three exactly same sequences (designated as *Wcor15-2A1*) showed 100% identity with the *Wcor15-2A* sequences from tetraploid and hexaploid wheats (Supplementary Table S5). Wcor15A, Wcor15B and Wcor15D primers failed to amplify the DNA from *T. monococcum* and *T. boeoticum* (Fig. 3b). In order to obtain the *Wcor15* gene from the *T. monococcum* and *T. boeoticum*, we redesigned a pair of Wcor15s primers which located at near the coding region based on the previously reported *Wcor15* gene (GenBank: AB095006). Three *Wcor15* sequences were obtained (Fig. 3c) and are identical which was designated as *Wcor15-2A2* containing a complete encoding region. The identity between *Wcor15-2A2* and *Wcor15-2A* was 97.87% at the DNA level (Supplementary Fig. S3 and Table S5).

In all eleven accessions of the *Sitopsis* species (1 *Ae. bicornis* S^b^S^b^, 1 *Ae. longissima* S^1^S^1^, 3 *Ae. sharonensis* S^sh^S^sh^, 3 *Ae. searsii* S^s^S^s^ and 3 *Ae. speltoides* SS) (Table 3) surveyed, the primer Wcor15A, Wcor15B and Wcor15D did not generate any amplification products (Fig. 3d). In order to obtain the *Wcor15* gene from the *Sitopsis* section, we again employed the primer Wcor15s which only amplified the coding region of *Wcor15* genes without the 5′ upstream sequence (>1 Kb). Eleven *Wcor15* sequences were obtained (Fig. 3c). Sequences analysis showed that all the three *Ae. speltoides* shared the two same exons of *Wcor15-2B* with tetraploid and hexaploid wheats. However, the intron of *Wcor15-2B* had two haplotypes in tetraploid and hexaploid wheats, one with a G deletion, the other with G insertion at the same location, while all the three *Ae. speltoides* only had one haplotype, a G deletion in the intron (Supplementary Fig. S4). The gene *Wcor15-2B* from *Ae. bicornis* (Q03-021), *Ae. longissima* (Q03-004), *Ae. sharonensis* (PI584395, PI584408 and PI584406), and *Ae. searsii* (PI599142, PI599124 and PI599126) showed 100% identity with each other, nevertheless, besides the difference of base G indel mentioned above, there were still many base differences compared with the gene from *Ae. speltoides*, 2 located in the first exon, 7 in the intron, and 5 in the second exon (Supplementary Fig. S4). These results suggested that *Ae. speltoides* is the most likely gene donor of *Wcor15-2B*, and diversification of the gene occurred during the first polyploidization.

From diploid *Ae. tauschii* (As 80, As 77, As 2392, As 2386, As 2387 and As 2388), six *Wcor15-2D* were cloned with the primer Wcor15D (Table 4). The six *Wcor15-2D* sequences were divided into two types: (I) As 2386, As 2387 and As 2388 with 100% identity, (II) As 80, As 77 and As 2392 with only a base substitution in the upstream non-coding regions. However, the *Wcor15-2D* from As 2386, As 2387 and As 2388 which belong to *Ae. tauschii* subsp. *strangulata* showed 100% identity with the *Wcor15-2D* from hexaploid wheat varieties (Supplementary Table S6). The coding region sequences from *Ae. bicornis* (Q03-021), *Ae. longissima* (Q03-004), *Ae. sharonensis* (PI584395, PI584408 and PI584406), and *Ae. searsii* (PI599142, PI599124 and PI599126) are same to the sequences from As 80, As 77 and As 2392 of *Ae. tauschii* subsp. *tauschii*. The primer Wcor15A and Wcor15B failed to amplify a product from these species (Fig. 3e). The results suggested that *Ae. tauschii* subsp. *strangulata* is the donor to the gene *Wcor15-2D* in hexaploid wheat.

## Discussion

The hexaploid bread wheat is believed to have originated through one or more hybridization events^16^,^17^,^18^. The study on origin of A, B and D genomes of bread wheat has been a hot topic. Understanding the origin of hexaploid wheat would benefit not only the genetic diversity but also expand the genetic basis for wheat breeding^23^,^42^. Previous studies have demonstrated that the sequence data of conserved gene can be used to study the evolution of gene families from different species^43^,^44^,^45^. In this study, we reported the utility of the *Wcor15* sequence to identify the progenitors of the tetraploid and hexaploid wheats and to define the evolution of their close relatives.

*Wcor15* is the member of the *Cor* gene family, which could encode the chloroplast-targeted protein when exposed to low temperature, and play an important role in the cold hardiness of wheat^37^,^46^,^47^,^48^,^49^,^50^,^51^. Based on the previous research on *Wcor15* (GenBank: AB095006) gene^37^, it was found that the gene of AB095006 located on chromosome 2AL, and we named it *Wcor15-2A*, in addition to this gene, we cloned the other two homoeologous *Wcor15* sequences (*Wcor15-2B* and *Wcor15-2D*) from the wheat genome 2BL and 2DL, respectively. Gene characterization analyzing showed that the three homoeologous *Wcor15* genes may belong to the chloroplast-targeted LEA3 protein, which is consistent with previous studies about characterization of *Wcor15-2A*^37^,^41^.

**Figure 4:**
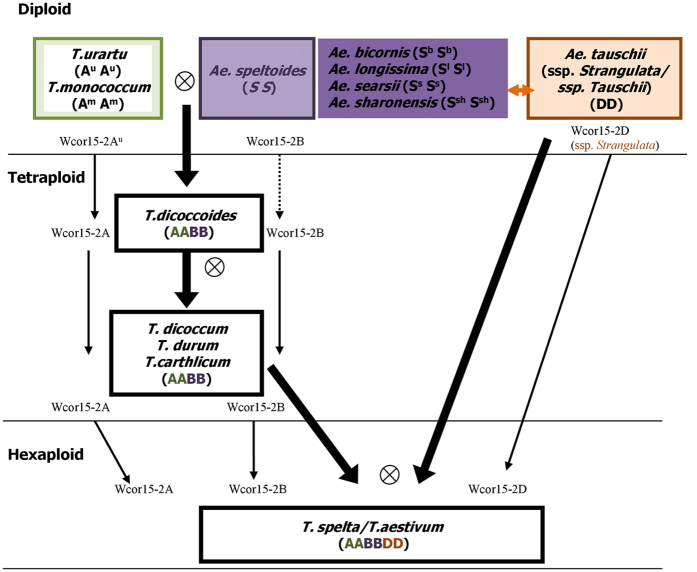
Schematic representation of the evolutionary history of wheat (*Triticum*) and diploid wild relatives. The thin dotted arrow illustrated that the gene sequence had been changed in the process of evolution. Two identical sequences were connected by the thin solid arrow. The *Sitopsis* section was be divided into two types: (I) *Ae. speltoides* and (II) *Ae. bicornis, Ae. longissima, Ae. sharonensis* and *Ae. searsii* by *Wcor15* gene. The orange double-headed arrow represents that the type II share the same sequence as *Ae. ssp. tauschii*.

To see whether *Wcor15-2A, Wcor15-2B* and *Wcor15-2D* genes are a conserved gene or not, the *Wcor15-2A, Wcor15-2B* and *Wcor15-2D* genes were cloned from 106 hexaploid wheat varieties from different geographical areas, 10 tetraploid species and 23 diploid species. Comparative analyses indicated that the *Wcor15-2A* (Supplementary Fig. S5), *Wcor15-2B* (Supplementary Fig. S4) and *Wcor15-2D* (Supplementary Fig. S6) genes were highly conservative during wheat evolution. Moreover, the three genes kept invariable during the second allopolyploidization from tetraploid to hexaploid (Fig. 4).

The *Wcor15* gene is a good candidate gene for investigating the donor of A-, B- and D-genome. The three homoeologous *Wcor15* sequences from the wheat genome A, B and D, respectively (Table 1) were different (Supplementary Fig. S1). Each of the three sequences was highly conservative in respective diploid (Supplementary Figs S4–S6), tetraploid (Supplementary Figs S4 and S5), and hexaploid (Supplementary Figs S7–S10). *Wcor15-2A* and *Wcor15-2B* on the A- and B-genome were very stable from diploid (AA, BB) to tetraploid (AABB) (Supplementary Figs S4 and S5), and from tetraploid (AABB) to hexaploid (AABBDD) (Supplementary Figs S4 and S5). *Wcor15-2D* is also highly conserved from diploid (DD) to hexaploid (AABBDD) (Supplementary Fig. S6). Comparison of the conserved *Wcor15* gene can provide some evidences on the origin of the A, B and D genome of common wheat.

The diploid wheats carrying A-genome included *T. urartu* (genome A^u^), *T. monococcum* (genome A^m^) and *T. boeoticum* (genome A^m^). To investigate the evolutionary relationships of *Wcor15-2A* genes between diploid and polyploid wheats, the sequences from *T. urartu, T. monococcum, T. boeoticum*, tetraploid and hexaploid wheats were compared. The six genes in diploid wheats (genome AA) were classified into two types (Supplementary Fig. S11). The three *T. urartu* (PI428222, PI428260 and PI428266) were type I (*Wcor15-2A1)*. The two *T. monococcum* (Mo4 and TL) and one *T. boeoticum* (Bo8) were type II (*Wcor15-2A2*). Compared to the *Wcor15-2A2* sequence, the *Wcor15-2A1* sequence showed much higher identity (100%) with the *Wcor15-2A* sequences from tetraploid and hexaploid wheats, suggesting that the *T. urartu* might be the direct donor of the *Wcor15-2A* in common wheat and that *Wcor15-2A* gene from A genome has no mutation during two sequential allopolyploidization events from *T. urartu* to tetraploid and hexaploid wheats. The result is consistency with the previous studies^23^,^24^,^52^. However, taking into consideration of no amplicon from *T. monococcum* and *T. boeoticum* when using Wcor15A primer, it suggested that non-coding regions of *Wcor15-2A1* were obviously different from *Wcor15-2A2*. Coding regions alignments also revealed variation between *Wcor15-2A2* and *Wcor15-2A1* from *T. urartu* (Supplementary Fig. S3).

Many researchers have suggested that the B genome is closely related to the S genome of the *Sitopsis* section which was comprised of five diploid species: *Ae. speltoides, Ae. longissima, Ae. sharonensis, Ae. searsii*, and *Ae. bicorni*^25^,^26^,^27^. To validate which species is the potential donor of B genome, eleven accessions of the *Sitopsis* species were amplified using the primers pair Wcor15A, Wcor15B and Wcor15D, but no PCR product was obtained. However, the primer Wcor15s successfully amplified the eleven accessions of the *Sitopsis* species, their sequences were classified into two types: (I) *Ae. speltoides* (PI542276, PI369663 and PI369624), and (II) *Ae. bicornis* (Q03-021), *Ae. longissima* (Q03-004), *Ae. sharonensis* (PI584395, PI584408 and PI584406), and *Ae. searsii* (PI599142, PI599124 and PI599126) (Supplementary Fig. S12). Our results showed that *Ae. speltoides* is distinct from the other species in the *Sitopsis* section, supporting the previous reports^30^,^31^,^32^,^33^.

In terms of coding region, *Wcor15-2B* sequences from different tetraploid and hexaploid wheats were divided into two groups by the insertion and deletion of a nucleotide G in the intron. All three *Ae. speltoides* sequences shared 100% identity, are different from tetraploid and hexaploid wheats with only a G deletion in the intron. On the other hand, no amplicon obtained from *Ae. speltoides* when using Wcor15B primer, suggested that non-coding regions of *Wcor15-2B* might be obvious differences between *Ae. speltoides* and tetraploid and hexaploid wheats. Our results suggested that *Ae. speltoides* might be the direct donor of the *Wcor15-2B* in tetraploid and hexaploid wheat varieties, non-coding region of *Wcor15-2B* gene from B genome might mutate during the first polyploidization from *Ae. speltoides* to tetraploid wheat, however, no change has occurred for this gene during the second allopolyploidization from tetraploid to hexaploid.

The *Wcor15-2D* sequences of D-genome were highly conservative among 106 hexaploid wheats. However, *Wcor15-2D* genes from six accessions of *Ae. tauschii* (Table 4) were divided into two allelic groups (Supplementary Fig. S13), suggesting variations in diploid wheats. Our results supported that subsp. *strangulata* may be the D-genome donor of common wheat suggested by previous studies^53^,^54^,^55^,^56^.

The *Wcor15* coding region of *Ae. tauschii* subsp. *tauschii* is same to the sequences from the S genome species, *Ae. bicornis, Ae. longissima, Ae. sharonensis* and *Ae. searsii*. Mayer *et al*.^57^ also reported that *Ae. sharonensis* was much closer to *Ae. tauschii* than to *Ae. speltoides*. The analysis of the multispecies coalescent species tree for *Aegilops* and *Triticum* diploid suggested that *Ae.bicornis, Ae. longissima, Ae. sharonensis* and *Ae. searsii* are more closely related to *Ae. tauschii* ssp. *tauschii* than *Ae. speltoides*^58^. However, no amplicon obtained from *Ae. bicornis, Ae. longissima, Ae. sharonensis* and *Ae. searsii* when Wcor15D primer was used, indicating that non-coding region of *Wcor15-2D* from *Ae. bicornis, Ae. longissima, Ae. sharonensis* and *Ae. searsii* were obviously different from that of *Ae. tauschii* ssp. *tauschii*.

This paper examined the evolutionary relationship of the *Wcor15* in diploid, tetraploid and hexaploid wheats during wheat allopolyploidization (Fig. 4). *Triticum urartu, Ae. speltoides* and *Ae. tauschii* subsp. *strangulata* are most likely the donors of the *Wcor15-2A, Wcor15-2B* and *Wcor15-2D* locus in common wheat, respectively. The *Wcor15* genes from subgenomes A and D were very conservative without insertion and deletion of bases during evolution of diploid, tetraploid and hexaploid. However, the *Wcor15-2B* genes mutated only during the first allopolyploidization event.

## Materials and Methods

### Wheat germplasm

One hundred and six hexaploid wheat (genome AABBDD) were used in this study, including 4 varieties from Winter wheat region of North China (WWRNC), 24 varieties from North China plain sub-region of Yellow & Huai river winter wheat region (NCPSR), 28 varieties from North Huai river plain sub-region of Yellow & Huai river winter wheat region (NHRPSR), 7 varieties from West upland sub-region of Yellow & Huai river winter wheat region (WUSR), 3 varieties from Jiaodong upland sub-region of Yellow & Huai river winter wheat region (JUSR), 11 varieties from Winter wheat region of middle and lower reaches of the Yangtze river (WWR), 7 varieties from Southwestern winter wheat region (SWWR), 13 varieties from Introduced wheat variety of foreign (IWVF)^59^, 5 spring wheat region of North China (SWRNC) and 4 *T. spelta*, 10 tetraploid species (AABB), and 23 diploid species (AA, BB and DD) (Table 3).

### DNA extraction, primer design, PCR and sequencing

Genomic DNA was extracted from young leaves of ten days seedlings using the Easypure plant Genomic DNA Kit (Sangon Biotech. Shanghai, China). Genome-specific primers were designed for each of the homoeologous Wcor15 genes (Table 2) using the software Primer Premier Version 5.0, and were synthesized by Shanghai Sangon Biological Technology Company.

PCR reaction were performed in total volumes of 20 μl, containing 12.8 μl ddH_2_O, 10 × PCR buffer (with Mg^2+^) 2.0 μl, dNTPs (2.5 mM) 2.0 μl, 0.5 μl of each primer (10 mM), 2.0 μl genomic DNA and *Taq* DNA polymerase (5 U/μl) 0.2 μl. Amplifications were performed using a standard touchdown PCR protocol with the appropriate annealing temperature. Each PCR was done five repeats up to a total of 100 μl.

All PCR products were directly sequenced. Each of 50 μl PCR products were sequenced by Shanghai Sangon Biological Technology Company, and the other 50 μl PCR products were sequenced by Huada Biotech Company in Beijing. To guarantee sequence accuracy, DNA sequencing was repeated three times.

Sequence analysis and characterization were performed using DNAman software at default settings (http://www.lynnon.com). The three homoeologous *Wcor15* sequences were identified at EBI web site (http://www.ebi.ac.uk/ena/)^38^. All of the sequences of the AA, BB, DD, AABB and AABBDD genome homoeologs of *Wcor15-2A, Wcor15-2B* and *Wcor15-2D* were submitted to the National Center for Biotechnology Information (NCBI) (http://www.ncbi.nlm.nih.gov/) (Table 4 and Supplementary Table S1).

## Additional Information

**How to cite this article**: Liu, F. *et al*. Molecular evolution of *Wcor15* gene enhanced our understanding of the origin of A, B and D genomes in *Triticum aestivum. Sci. Rep.* **6**, 31706; doi: 10.1038/srep31706 (2016).

## Supplementary Material

Supplementary Information

## Figures and Tables

**Table 1:** Three homoeologous Wcor15 sequences obtained from the ENA.

Probe	Target Accession	Description for target sequence	Query length (bp)	Target length (bp)	Identity (%)
ORF of *Wcor15*	CBTL0110083500	*Triticum aestivum* WGS project CBTL0000000000 data, contig IWGSC_CSS_2AL_CONTIG_332752	563	563	100
ORF of *Wcor15*	CBTL0111257031	*Triticum aestivum* WGS project CBTL0000000000 data, contig IWGSC_CSS_2BL_CONTIG_357080	563	565	94
ORF of *Wcor15*	CBTL0110522649	*Triticum aestivum* WGS project CBTL0000000000 data, contig IWGSC_CSS_2DL_CONTIG_428903	563	566	95

The ORF sequence (563 bp) of *Wcor15* gene (GenBank: AB095006) was used as a probe. The ORF sequence of CBTL0110083500 on 2AL was 563 bp. The ORF sequence of CBTL0111257031 on 2BL was 565 bp. The ORF sequence of CBTL0110522649 on 2DL was 566 bp.

**Table 2:** Primers used in this study.

Primer set	Primer sequence (5′-3′)	Amplified target	Size of PCR product (bp)
Wcor15A	CCTTCTCATCCATCATAGC	2AL genome-specific	1840
	TACACCTCGTCTCCTCCT		
Wcor15B	CCATCATTAGTGAAGGGT	2BL genome-specific	2400
	AGACACACGATATACTCAG		
Wcor15D	GAGAGAGTAGGTATTTTGC	2DL genome-specific	2012
	CGGTAATCATGTATGTCAGA		
Wcor15s	CCCTACCCACCCATCCAT	Coding region of Wcor15	570
	TTGTCCGTGATGCCCTGT		

**Table 3:** The names of diploid, tetraploid and hexaploid wheat accessions.

Accessions		Genome	Nu. of accessions
Common wheat	WWRNC	AABBDD	4
	NCPSR	AABBDD	24
	NHRPSR	AABBDD	28
	WUSR	AABBDD	7
	JUSR	AABBDD	3
	WWR	AABBDD	11
	SWWR	AABBDD	7
	SWRNC	AABBDD	5
	IWVF	AABBDD	13
Spelt wheat		AABBDD	4
*T.dicoccoides*		AABB	3
*T.dicoccum*		AABB	3
*T.durum*		AABB	3
*T.carthlicum*		AABB	1
*T.urartu*		A^u^A^u^	3
*T.m.boeoticum*		A^m^A^m^	1
*T.m.monococcum*		A^m^A^m^	2
*Ae. speltoides*		SS	3
*Ae. longissima*		S^1^S^1^	1
*Ae. bicornis*		S^b^S^b^	1
*Ae.sharonensis*		S^sh^S^sh^	3
*Ae.searsii*		S^s^S^s^	3
*Ae. tauschii* ssp*. tauschii*		DD	3
*Ae. tauschii* ssp*. strangulata*		DD	3

WWRNC: Winter wheat region of North China, NCPSR: North China plain sub-region of Yellow & Huai river winter wheat region, NHRPSR: North Huai river plain sub-region of Yellow & Huai river winter wheat region, WUSR: West upland sub-region of Yellow & Huai river winter wheat region, JUSR: Jiaodong upland sub-region of Yellow & Huai river winter wheat region, WWR: Winter wheat region of middle and lower reaches of the Yangtze river, SWWR: Southwestern winter wheat region, SWRNC: Spring wheat region of North China, IWVF: Introduced wheat variety of foreign.

**Table 4:** Description of diploid and tetraploid accessions used in this study.

No	Accession	Species	Genome	GenBank accession			
				Primer Wcor15A	Primer Wcor15B	Primer Wcor15D	Primer Wcor15S
1	PI428222^a^	*T.urartu*	A^u^A^u^	KT265050	0	0	—
2	PI428260^a^	*T.urartu*	A^u^A^u^	KT265051	0	0	—
3	PI428266^a^	*T.urartu*	A^u^A^u^	KT265052	0	0	—
4	Bo8^b^	*T.m.boeoticum*	A^m^A^m^	0	0	0	KT265047
5	Mo4^b^	*T.m.monococcum*	A^m^A^m^	0	0	0	KT265049
6	TL^b^	*T.m.monococcum*	A^m^A^m^	0	0	0	KT265048
7	PI542276^a^	*Ae. speltoides*	SS	0	0	0	KU365997
8	PI369663^a^	*Ae. speltoides*	SS	0	0	0	KU365998
9	PI369624^a^	*Ae. speltoides*	SS	0	0	0	KU365999
10	Q03-004^c^	*Ae. longissima*	S^1^S^1^	0	0	0	KU366001
11	Q03-021^c^	*Ae. bicornis*	S^b^S^b^	0	0	0	KU366002
12	PI584395^a^	*Ae.sharonensis*	S^sh^S^sh^	0	0	0	KU366003
13	PI584408^a^	*Ae.sharonensis*	S^sh^S^sh^	0	0	0	KU366004
14	PI584406^a^	*Ae.sharonensis*	S^sh^S^sh^	0	0	0	KU366005
15	PI599142^a^	*Ae.searsii*	S^s^S^s^	0	0	0	KU366006
16	PI599124^a^	*Ae.searsii*	S^s^S^s^	0	0	0	KU366007
17	PI599126^a^	*Ae.searsii*	S^s^S^s^	0	0	0	KU366008
18	As77^a^	*Ae. tauschii* ssp*. tauschii*	DD	0	0	KT265067	—
19	As80^a^	*Ae. tauschii* ssp*. tauschii*	DD	0	0	KT265068	—
20	As2392^a^	*Ae. tauschii* ssp*. tauschii*	DD	0	0	KT265071	—
21	As2386^a^	*Ae. tauschii* ssp*. strangulata*	DD	0	0	KU366000	—
22	As2387^a^	*Ae. tauschii* ssp*. strangulata*	DD	0	0	KT265069	—
23	As2388^a^	*Ae. tauschii* ssp*. strangulata*	DD	0	0	KT265070	—
24	As829^a^	*T.dicoccoides*	AABB	KT264939	KT265004	0	—
25	As836	*T.dicoccoides*	AABB	KT264940	KT265005	0	—
26	As839^a^	*T.dicoccoides*	AABB	KT264941	KT265006	0	—
27	PI272527^a^	*T.dicoccum*	AABB	KT264942	KT265007	0	—
28	PI193873^a^	*T.dicoccum*	AABB	KT264943	KT265008	0	—
29	PI221401^a^	*T.dicoccum*	AABB	KT264944	KT265009	0	—
30	Club57^b^	*T.durum*	AABB	KT264945	KT265010	0	—
31	Simeto-2^b^	*T.durum*	AABB	KT264946	KT265011	0	—
32	Dr8^b^	*T.durum*	AABB	KT264947	KT265012	0	—
33	Tc^b^	*T.carthlicum*	AABB	KT264948	KT265013	0	—

“**—**” represents that the wheat DNA sample is only amplified with appropriate size using corresponding primer but not sequenced. “0” indicated that the corresponding primer did not generate any amplification products.

^a^The accessions were provided by Triticeae Research Institute, Sichuan Agricultural University, China.

^b^The accessions were provided by the Institute of Crop Science, Chinese Academy of Agricultural Sciences, China.

^c^The accessions were provided by Huazhong University of Science and Technology (HUST), China.

## References

[b1] Molnar I. . Flow cytometric chromosome sorting from diploid progenitors of bread wheat, *T. urartu, Ae. speltoides* and *Ae. tauschii*. Theoretical and Applied Genetics 127, 1091–1104 (2014). 24553964 10.1007/s00122-014-2282-2

[b2] Lev-Yadun S., Gopher A. & Abbo S. Archaeology–Che cradle of agriculture. Science 288, 1602–1603 (2000). 10858140 10.1126/science.288.5471.1602

[b3] Gornicki P. . The chloroplast view of the evolution of polyploid wheat. New Phytologis 204, 704–714 (2014). 10.1111/nph.12931 25059383

[b4] Soltis P. S. & Soltis D. E. The role of genetic and genomic attributes in the success of polyploids. Proceedings of the National Academy of Sciences of the United States of America 97, 7051–7057 (2000). 10860970 10.1073/pnas.97.13.7051 PMC34383

[b5] Otto S. P. The evolutionary consequences of polyploidy. Cell 131, 452–462 (2007). 17981114 10.1016/j.cell.2007.10.022

[b6] Otto S. P. & Whitton J. Polyploid incidence and evolution. Annual Review of Genetics 34, 401–437 (2000). 10.1146/annurev.genet.34.1.401 11092833

[b7] Adams K. L. & Wendel J. F. Polyploidy and genome evolution in plants. Current Opinion in Plant Biology 8, 135–141 (2005). 15752992 10.1016/j.pbi.2005.01.001

[b8] Onge K. R. S. . Coalescent-Based Analysis Distinguishes between Allo- and Autopolyploid Origin in Shepherd’s Purse (Capsella bursa-pastoris). Molecular Biology and Evolution 29, 1721–1733 (2012). 22319173 10.1093/molbev/mss024

[b9] Vamosi J. C. & McEwen J. R. Origin, elevation, and evolutionary success of hybrids and polyploids in British Columbia, Canada. Botany-Botanique 91, 182–188 (2013).

[b10] Haider N. Evidence for the origin of the B genome of bread wheat based on chloroplast DNA. Turkish Journal of Agriculture and Forestry 36, 13–25 (2012).

[b11] Hirosawa S. . Chloroplast and nuclear DNA variation in common wheat: insight into the origin and evolution of common wheat. Genes & Genetic Systems 79, 271–282 (2004). 15599057 10.1266/ggs.79.271

[b12] Bennett M. D. & Smith J. B. Nuclear DNA amounts in angiosperms. Philosophical transactions of the Royal Society of London Series B, Biological sciences 274, 227–274 (1976). 6977 10.1098/rstb.1976.0044

[b13] Devos K. M. & Gale M. D. Genome relationships: The grass model in current research. Plant cell 12, 637–646 (2000). 10810140 10.1105/tpc.12.5.637 PMC139917

[b14] Sakamura T. Kurze Mitteilung ueber die Chromosomenzahlen und die Verwandtschaftsverhältnisse der Triticum-arten. Bot Mag 32, 151–154 (1918).

[b15] Kihara H. Cytologische und genetische Studien bei wichtigen Getreidearten mit besonderer Rucksicht auf das Verhalten der Chromosomen und die Sterilitat in den Bastarden. Mem Coll Sci Kyoto Imp Univ Ser 1, 1–200 (1924).

[b16] Dvorak J. & Akhunov E. D. Tempos of gene locus deletions and duplications and their relationship to recombination rate during diploid and polyploid evolution in the aegilops-triticum alliance. Genetics 171, 323–332 (2005). 15996988 10.1534/genetics.105.041632 PMC1456522

[b17] Zhang H. . Evolution of the BBAA Component of Bread Wheat during Its History at the Allohexaploid Level. Plant Cell 26, 2761–2776 (2014). 24989045 10.1105/tpc.114.128439 PMC4145112

[b18] Brenchley R. . Analysis of the breadwheat genome using whole-genome shotgun sequencing. Nature 491, 705–710 (2012). 23192148 10.1038/nature11650 PMC3510651

[b19] Gulbitti-Onarici S. E. L. M. A., Sumer S. & Ozcan S. Determination of phylogenetic relationships between some wild wheat species using amplified fragment length polymorphism (AFLP) markers. Botanical Journal of the Linnean Society 153, 67–72 (2007).

[b20] Gulbitti-Onarici S. E. L. M. A., Sancak C., Sumer S. & Ozcan S. Phylogenetic relationships of some wild wheat species based on the internal transcribed spacer sequences of nrDNA. Current Science 96, 794–800 (2009).

[b21] Sourdille P., Tavaud M., Charmet G. & Bernard M. Transferability of wheat microsatellites to diploid *Triticeae* species carrying the A, B and D genomes. Theoretical and Applied Genetics 103, 346–352 (2001).

[b22] Ozkan H., Tuna M., Kilian B., Mori N. & Ohta S. Genome size variation in diploid and tetraploid wild wheats. Aob Plants, plq015 (2010). 10.1093/aobpla/plq015 PMC2992354 22476073

[b23] Luo G. . Composition, variation, expression and evolution of low-molecular-weight glutenin subunit genes in *Triticum urartu*. Bmc Plant Biology 15, 68 (2005). 25849991 10.1186/s12870-014-0322-3 PMC4364320

[b24] Huang S. . Genes encoding plastid acetyl-CoA carboxylase and 3-phosphoglycerate kinase of the *Triticum/Aegilops* complex and the evolutionary history of polyploid wheat. Proceedings of the National Academy of Sciences of the United States of America 99, 8133–8138 (2002). 12060759 10.1073/pnas.072223799 PMC123033

[b25] Tsunewaki K. & Ogihara Y. The Molecular Basis of Genetic Diversity among Cytoplasms of TRITICUM and AEGILOPS Species. II. on the Origin of Polyploid Wheat Cytoplasms as Suggested by Chloroplast DNA Restriction Fragment Patterns. Genetics 104, 155–171 (1983). 17246126 10.1093/genetics/104.1.155 PMC1202061

[b26] Dvorak J. & Zhang H. B. Variation in repeated nucleotide sequences sheds light on the phylogeny of the wheat B and G genomes. Proceedings of the National Academy of Sciences of the United States of America 87, 9640–9644 (1990). 11607134 10.1073/pnas.87.24.9640 PMC55228

[b27] Wang G. Z., Miyashita N. T. & Tsunewaki K. Plasmon analyses of *Triticum* (wheat) and *Aegilops*: PCR-single-strand conformational polymorphism (PCR-SSCP) analyses of organellar DNAs. Proceedings of the National Academy of Sciences 94, 14570–14577 (1997). 10.1073/pnas.94.26.14570 PMC25058 9405654

[b28] Golovnina K. A. . Molecular phylogeny of the genus *Triticum L*. Plant Systematics and Evolution 264, 195–216 (2007).

[b29] Haider N. The origin of the B-genome of bread wheat (*Triticum aestivum L.*). Genetika 49, 303–314 (2013). 23755530 10.7868/s0016675813030077

[b30] Salina E. A. . Phylogenetic reconstruction of *Aegilops* section *Sitopsis* and the evolution of tandem repeats in the diploids and derived wheat polyploids. Genome 49, 1023–1035 (2006). 17036077 10.1139/g06-050

[b31] Kilian B. . Independent wheat B and G genome origins in outcrossing *Aegilops* progenitor haplotypes. Molecular Biology and Evolution 24, 217–227 (2007). 17053048 10.1093/molbev/msl151

[b32] Adonina I. G., Salina E. A., Pestsova E. G. & Roder M. S. Transferability of wheat microsatellites to diploid *Aegilops* species and determination of chromosomal localizations of microsatellites in the S genome. Genome 48, 959–970 (2005). 16391665 10.1139/g05-072

[b33] Zhang L. Y. . Transferable bread wheat EST-SSRs can be useful for phylogenetic studies among the *Triticeae* species. Theoretical and Applied Genetics 113, 407–418 (2006). 16736140 10.1007/s00122-006-0304-4

[b34] Salamini F., Ozkan H., Brandolini A., Schafer-Pregl R. & Martin W. Genetics and geography of wild cereal domestication in the Near East. Nature Reviews Genetics 3, 429–441 (2002). 10.1038/nrg817 12042770

[b35] Li X. F., Liu H. Y., Gao J. R. & Wang. H. G. Development of Two Powdery Mildew and Stripe Rust Resistant Wheat Lines from (Triticum turgidum x Haynaldia villosa amphiploid) x Synthetic Wheat Hybrids. Cereal Research Communications 38, 307–316 (2010).

[b36] Dudnikov A. J. *Aegilops tauschii Coss*: allelic variation of enzyme-encoding genes and ecological differentiation of the species. Genetic Resources and Crop Evolution 61, 1329–1344 (2014).

[b37] Takumi S. . Cold-specific and light-stimulated expression of a wheat (*Triticum aestivum L.*) *Cor* gene *Wcor15* encoding a chloroplast-targeted protein. Journal of Experimental Botany 54, 2265–2274 (2003). 12909691 10.1093/jxb/erg247

[b38] Pakseresht N. . Assembly information services in the European Nucleotide Archive. Nucleic Acids Research 42, D38–D43 (2014). 24214989 10.1093/nar/gkt1082 PMC3965037

[b39] May T. & Soll J. 14-3-3 proteins form a guidance complex with chloroplast precursor proteins in plants. Plant Cell 12, 53–63 (2000). 10634907 10.1105/tpc.12.1.53 PMC140214

[b40] Ohno R., Takumi S. & Nakamura C. Phosphorylation of wheat chloroplast- targeting COR/LEA proteins via 50-kDa protein kinase. Instructions to Authors 101, 1–3 (2006).

[b41] NDong C. . Cold-regulated cereal chloroplast late embryogenesis abundant-like proteins. Molecular characterization and functional analyses. Plant Physiology 129, 1368–1381 (2002). 12114590 10.1104/pp.001925 PMC166530

[b42] Peng J. H., Sun D. F. & Nevo E. Domestication evolution, genetics and genomics in wheat. Molecular Breeding 28, 281–301 (2011).

[b43] Peng J. H., Sun D. F., Peng Y. L. & Nevo E. Gene discovery in *Triticum dicoccoides*, the direct progenitor of cultivated wheats. Cereal Research Communications 41, 1–22 (2012).

[b44] Luo S., Peng J. H., Li K. P., Wang M. & Kuang H. H. Contrasting evolutionary patterns of the *Rp1* resistance gene family in different species of Poaceae. Molecular biology and evolution 28, 313–325 (2011). 20713469 10.1093/molbev/msq216

[b45] Zhu T. T., Nevo E., Sun D. F. & Peng J. H. Phylogenetic analyses unravel the evolutionary history of NAC proteins in plants. Evolution 66, 1833–1848 (2012). 22671550 10.1111/j.1558-5646.2011.01553.x

[b46] Tunnacliffe A. & Wise M. J. The continuing conundrum of the LEA proteins. Naturwissenschaften 94, 791–812 (2007). 17479232 10.1007/s00114-007-0254-y

[b47] Wang B. F., Wang Y. C., Zhang D. W., Li H. Y. & Yang C. P. Verification of the resistance of a *LEA* gene from Tamarix expression in Saccharomyces cerevisiae to abiotic stresses. Journal of Forestry Research 19, 58–62 (2008).

[b48] Vaseva I. I., Grigorova B. S., Simova-Stoilova L. P., Demirevska K. N. & Feller U. Abscisic acid and late embryogenesis abundant protein profile changes in winter wheat under progressive drought stress. Plant Biology 12, 698–707 (2010). 20701692 10.1111/j.1438-8677.2009.00269.x

[b49] Kramer D., Breitenstein B., Kleinwaechter M. & Selmar D. Stress Metabolism in Green Coffee Beans (*Coffea arabica L.*): Expression of Dehydrins and Accumulation of GABA during Drying. Plant and Cell Physiology 51, 546–553 (2010). 20208063 10.1093/pcp/pcq019

[b50] Costa C. D. N. M. . Levels of MeLEA3, a cDNA Sequence Coding for an Atypical Late Embryogenesis Abundant Protein in Cassava, Increase Under *In Vitro* Salt Stress Treatment. Plant Molecular Biology Reporter 29, 997–1005 (2011).

[b51] Shimamura C., Ohno R., Nakamura C. & Takumi S. Improvement of freezing tolerance in tobacco plants expressing a cold-responsive and chloroplast-targeting protein WCOR15 of wheat. Journal of Plant Physiology 163, 213–219 (2006). 16399012 10.1016/j.jplph.2005.06.008

[b52] Baum B. R. & Bailey L. G. The origin of the A genome donor of wheats (*Triticum: Poaceae*) -a perspective based on the sequence variation of the 5S DNA gene units. Genetic Resources and Crop Evolution 51, 183–196 (2004).

[b53] Dudnikov A. J. Allozyme variation in Transcaucasian populations of *Aegilops squarrosa*. Heredity 80, 248–258 (1998).

[b54] Aghaei M. J., Mozafari J., Taleei A. R., Naghavi M. R. & Omidi M. Distribution and diversity of *Aegilops tauschii* in Iran. Genetic Resources and Crop Evolution 55, 341–349 (2008).

[b55] Matsuoka Y., Nishioka E., Kawahara T. & Takumi S. Genealogical analysis of subspecies divergence and spikelet-shape diversification in central Eurasian wild wheat *Aegilops tauschii Coss*. Plant Systematics and Evolution 279, 233–244 (2009).

[b56] Huang L. . Haplotype variations of gene *Ppd-D1* in *Aegilops tauschii* and their implications on wheat origin. Genetic Resources and Crop Evolution 59, 1027–1032 (2012).

[b57] Mayer K. F. . A chromosome-based draft sequence of the hexaploid bread wheat (*Triticum aestivum*) genome. Science 345, 1251788 (2014). 25035500 10.1126/science.1251788

[b58] Marcussen T. . Ancient hybridizations among the ancestral genomes of bread wheat. Science 345, 1250092 (2014). 25035499 10.1126/science.1250092

[b59] Wang S. X., Zhu Y. L., Zhang H. P., Chang C. & Ma C. X. Analysis of Genetic Diversity and Relationship among Wheat Breeding Parents by SSR Markers. Journal of Triticeae Crops 34, 621–627 (2014).

