# Supplementary material for: Molecular evolution of Wcor15 gene enhanced our understanding of the origin of A, B and D genomes in Triticum aestivum
Source: Sci Rep. 2016 Aug 16;6:31706. doi: 10.1038/srep31706 (PMC4985644; doi:10.1038/srep31706)
Supplement: Supplementary Information [file srep31706-s1.pdf]

Molecular evolution of *Wcor15* gene enhanced our understanding of the origin of A, B and D genomes in *Triticum aestivum*

Fangfang Liu[1,2,+], Hongqi Si[1,2,+,*], Chengcheng Wang[1,2], Genlou Sun[1,3,*], Erting Zhou[1], Can Chen[1] & Chuanxi Ma[1,2,4,5,*]

[1]School of Agronomy, Anhui Agricultural University, Hefei 230036, China

[2]Key Laboratory of Wheat Biology and Genetic Improvement on South Yellow & Huai River Valley, Ministry of Agriculture, Hefei 230036, China

[3]Biology Department, Saint Mary's University, Halifax, NS, Canada B3H 3C3

[4]National United Engineering Laboratory for Crop Stress Resistance Breeding, Hefei 230036, China

[5]Anhui Key Laboratory of Crop Biology, Hefei 230036, China

[+] Fangfang Liu (email: 519034790@qq.com) and Hongqi Si (email: sihq2002@163.com) contributed equally to this work.

[*] Corresponding authors:
Hongqi Si, email: sihq2002@163.com; Tel: +86-551-65786213; Fax: +86-551-65786869.
Genlou Sun, email: genlou.sun@smu.ca;
Chuanxi Ma, email: machuanxi@ahau.edu.cn; Tel: +86-551-65786293; Fax: +86-551-65785833

Other authors:
Chengcheng Wang, email: 1554472311 @qq.com;
Erting Zhou, email: 984452023 @qq.com;
Can Chen, email: chencan-l@163.com.

Supplementary Information

Supplementary Fig. S1 Comparison of three homoeologous *Wcor15* sequences identified in Annong0822 on 2AL, 2BL and 2DL.

Supplementary Fig. S2 15 substitutions of bases and 2 insertion and deletion of bases were found among *Wcor15-2B* sequences from 54 hexaploid wheat varieties and 10 tetraploid wheats.

Supplementary Fig. S3 Sequence alignments of the *Wcor15-2A* from hexaploid wheat, *Wcor15-2A 1* from *T.urartu* and *Wcor15-2A 2* from *T.m.boeoticum* and *T.m.monococcum*.

Supplementary Fig. S4 Sequence alignments of the *Wcor15-2B* from 2 hexaploid wheat Annong 0227 and Annong 0822, 10 tetraploid wheat, and *Wcor15* from 1 *Ae. bicornis* S^bS^b, 1 *Ae. longissima* S^1S^1, 3 *Ae. sharonensis* $S^{sh}S^{sh}$, 3 *Ae. Searsii* S^sS^s and 3 *Ae. speltoides* SS.

Supplementary Fig. S5 Sequence alignments of the *Wcor15-2A* from hexaploid wheat, tetraploid wheat, and *T. urartu, T. monococcum linn* and *T. boeoticum boiss.*

Supplementary Fig. S6 Sequence alignments of the *Wcor15-2D* from hexaploid wheat, tetraploid wheat, and diploid *Ae. tauschii* (As 80, As 77, As 2392, As 2386, As 2387 and As 2388).

Supplementary Fig. S7 Distribution of hexaploid wheat with different geographical areas.

Supplementary Fig. S8 Sequence alignments of the *Wcor15-2A* from hexaploid wheat with different geographical areas.

Supplementary Fig. S9 Sequence alignments of the *Wcor15-2B* from hexaploid wheat with different geographical areas.

Supplementary Fig. S10 Sequence alignments of the *Wcor15-2D* from hexaploid wheat with different geographical areas.

Supplementary Fig. S11 Cluster analysis of the *Wcor15-2A* from *T. urartu, T. monococcum* and *T. boeoticum.*

Supplementary Fig. S12 Cluster analysis of the *Wcor15-2B* from *the Sitopsis* section.

Supplementary Fig. S13 Cluster analysis of the *Wcor15-2D* from *Ae. tauschii*.

Supplementary Table S1. Geographic regions and relevant properties of tested 106 hexaploid wheat accessions.

Supplementary Table S2. Homology analysis of 99 Wcor15-2A sequences with the sequence of Annong 0822 in hexaploid wheats by BLAST (https: // blast. ncbi. nlm. nih. gov/ Blast .cgi).

Supplementary Table S3. Homology analysis of 53 Wcor15-2B sequences with the sequence of Annong 0822 in hexaploid wheats by BLAST (https: // blast. ncbi. nlm. nih. Gov / Blast.cgi).

Supplementary Table S4. Homology analysis of 32 Wcor15-2D sequences with the sequence of Annong 0822 in hexaploid wheats by BLAST (https: // blast. ncbi. nlm. nih. Gov / Blast.cgi).

Supplementary Table S5. Homology analysis of the Wcor15-2A DNA sequences in the diploid, tetraploid and hexaploid species.

Supplementary Table S6. Homology analysis of the Wcor15-2D DNA sequences in the diploid and hexaploid species.

```
2AL    CATCATAGCAGCTGACATCTTTTGAAGGCTCTCCTTGAGAGCAAATATAATAAGTGATGT     60
2BL    CATCATAGCA..TGATATCTTTTGAAGGCGCTCCTTGAGAGCAAGTATAATAAGTGATGT     58
2DL    CATCATAGAAGCTGACATTTTTTGAAGGCGCTCCTTGGGAGCAAGTATAATAAGTG....     56

2AL    AAGCGAGATGTAAGACTTTCAATATAATATTTTTAATGAGTTGAAAGAGAGAGAAGATAA    120
2BL    AAGCGGGATGTAAAACTTTCGATATTATATTTTTGCTGAGTTGAAGGTGATAGGAGAGAA    118
2DL    .......ATGTAAGACTTTCAATATTATATTTTTGCTGAGTTGAAGAAGAGGGGAGAGAA    109

2AL    AAGAGAATAGAAGCAGGCTATAGATTAATAACCAGTTGCAGCATGTGCCCTAGTGGCTTT    180
2BL    AAGAGAATAGAAGCATATTACAAATTAACAACCAGTTGTAGCATGTGCCCTAGAGGCTTT    178
2DL    AAGAGAATAGAAGCAGGCTACAGACTAATAACCAGTTGTAGCATGTGCCCTAGAGGCTTT    169

2AL    GTGAGAGAGTGAGGTGACTCATGTATCAACAAAGTAATACTCCCTCCTTCTAGGAATACT    240
2BL    TTGAGAGTGTGAG.TGAGTCATGTATCAACAAAGTAATACTCTCTCCGT............    226
2DL    GTGAAAGAGTGAGGTGAGTTATGTATCAACAAAGTAATACTCCCTTTGCCTAGAAATACT    229

2AL    TGTCGGAGAAACGGATAAAAATGGATGTA...TGTAGAACTAAAATACGTCTAAATACAT    297
2BL    ........AAACTAATATAAACGCATTTA..GATTACTATTTTAATG.ATCTAAATGCTT    275
2DL    TGTTGGAAAAATAGATAAAAATGAATGTACTATCTAGAATTAAAATACGCCCAAATACAT    289

2AL    T.CATTTTCCGACAAATATTTTCAGATGAAGGGAGTACATATCTATATCTAATTATTATA    356
2BL    T.........TATATTAGTTTACAGAGGGA.GTACTACATTTTTATATCTAACTATTATA    325
2DL    CATTTTTTCTGATAAATATTTTCAGACGGATGGAGTACATATTTATATCTAACTATTATA    349

2AL    TTTGCTGACTATAAACTTAAATATAAATGACATGGCAACATTATATAGC...........    405
2BL    CTTGCCAGCTATAAGCGTAATTATAGATGACGCGTCAACATCATATAGCCAGCAGCCGGC    385
2DL    CTTGCCGCATATAAATTTAATTATAGATGACGTGCCAACATCATATAGCCAGCAGCCGGC    409

2AL    .....TATTAACCATGCTCCCGACCATGTATCGGTGATGTCATTATTGTTCATTCAATCA    460
2BL    TATGCTATTAACCATGCTCTCGACCATGTATCGGTGATGTCATTATTGTTCATTCAATCA    445
2DL    TATGCTATTAAACATGTTGTGGACCATGTATCGGCGACGTCATTATTGTTCATTCAATCA    469

2AL    CTTTCCAATT.AATACTTTCTACAGATGGGATATAAATATGGCTAAACTTACCCTTGATT    519
2BL    CTTTCCAATTTAATACTTTCTACAGATAGGATATAAACATGGCTAAACTTACCCTTGATT    505
2DL    CTTTCCAGTTTAATATTTTCTACAGATGGGATATAAATATGGCTGAACTTACCCTTGATT    529

2AL    AATCGACGGACGGTATCAAATATCTTCTGCTATCATAAGTTGAAGCATCTTAAAAGAGCT    579
2BL    AATCGACGGCCGGTATCACATATCTTCTGTTATCATAAGTCGAAGCATCTTAAAAGAGCT    565
2DL    AATCGACGGCCGGTATCAAATATCTCCTGTTATCATAAGTTGAAGCATCTTCAAAGAGCT    589

2AL    CCTGACAAAACCTTGGTTGTACGTAACCTTCAAACGTGGACAGTAGGATTTGTTTCTTCG    639
2BL    CCTGACAAAACCTTGATTGTACGTAACCTTCAAACGTGGACAGTAGGATTTGTTTCTTCG    625
2DL    CCTGACAAAACCTTGGTTGTACGTAACCTTCAAACGTGGACAGTAGGATTTGTTTCTTCG    649

2AL    ATCCGCTCCAAACAGGTCATGTAGGTATACGTGCCTTCGTACGAGACGCTGGTGGGGTAA    699
2BL    ATCCGCCCCAAACAGGTCATGTAGGTATACGTGTGTTCCCGTGAGAGGCGGGTGGGGTAA    685
2DL    ATCCGCTCCAAACAGGTCATGTAGGTATACGTGTGTTCCCGTGAGAGGCGTGTGGGGTAA    709

2AL    GCAGCTA............................CCCATTTCTTTTCTTTTATTGGTAT    731
2BL    GCAGCTAGTAGTAGCAAGCATGGGTGCAGCAGATACCCATTTCTTTTCTTTTATTGGTA.    744
2DL    GCAGCTAGTAGTAGCAAGCATGGGTGCAGCAGATACCCATTTCTTTTCTTTTATTGGTAT    769

2AL    AGGAAATTCGACCGTACATTACCACATAAACCTTGTCGACAACTCTCCGCGCACTGCTCC    791
2BL    AGGAAATTCGACCGTACATTACCACATAA.CCTTGTCGACAACTCTCCGCGCACGGCTGC    803
2DL    GAGAAATTCGACCGTACATTACCACATAA.CCTTGTCGACAGTTCTCCGCGCACGGCTGC    828

2AL    ACCACAGAACCTTGTCGACCGTCGATTGTCGCCATGCGCCGGAGCGATCGGCCCCCGACG    851
2BL    ACCACAGAACCTTGTCGACCGTCGATTGTCGCCATGCGCCGCAGCGATCGGCCCCCGACG    863
2DL    ACCACTCAACCTTGTCGACCGTCGATTGTCGCCATGCGCCGGAGCGATCGCCGCCCGACG    888

2AL    TGTCACCCCCCAGCTCACCCTTCGCCCCTCCGCGTCCGGCCTATAAAAACGACGATGCAC    911
2BL    TGTCACCGCCCACCTCATCCTTCGCCCCTCCGCGTCCAGCCTATAAAAGAGACGATGCAC    923
2DL    TGTCGCCAGCCACCTCACCCTTCGCCCCTCCGCGTCCAGCCTATAAAAACGACGATGCAC    948

2AL    CTCTCGTCCTCCCATTGCATTGCAAGTGAAGCTCACACAACCTACCCTACCCTACCCACC    971
2BL    CTCTCGTCCTCCCATCGTACAGCAAGTGAAGCTCACACAACCTA.....CCCTACCCACC    978
2DL    CTCTCGTCCTCCCATCGGACTGCAAGTGAAGCTCACACGACCTAGCCTACCCTACCCACC   1008
```

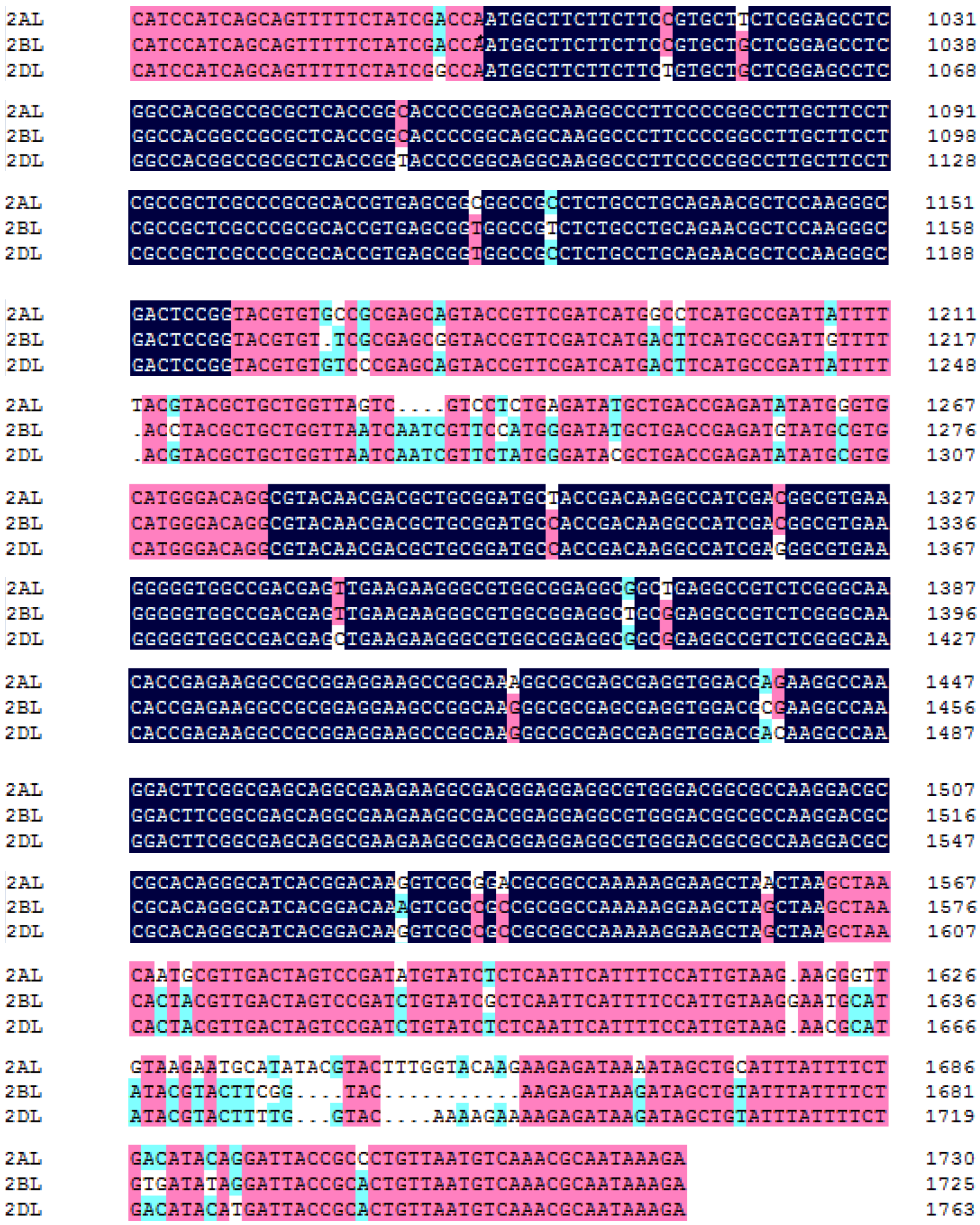

Supplementary Fig. S1 Comparison of three homoeologous *Wcor15* sequences identified in Annong0822 from 2AL, 2BL and 2DL. 2AL (*Wcor15-2A*: KT264885), 2BL (*Wcor15-2B*: KT264957), and 2DL (*Wcor15-2D*: KT265022). The blue parts are two exons.

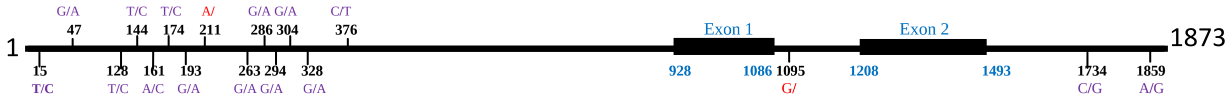

Supplementary Fig. S2 15 substitutions of bases and 2 insertion and deletion of bases were found among *Wcor15-2B* sequences from 54 hexaploid wheat varieties and 10 tetraploid wheats. The purple portions stand for base substitution. The red portions are the typical representation of the base deletion.

```
Wcor15-2A    ATGGCTTCTTCTTCCGTGCTTCTCGGAGCCTCGGCCACGGCCGCGCTCACCGGCACCCCGGCAGGCAAGGCCCTTCCCCGGCCTTGCTTCCTCGCCGCTC  100
Wcor15-2A1   ATGGCTTCTTCTTCCGTGCTTCTCGGAGCCTCGGCCACGGCCGCGCTCACCGGCACCCCGGCAGGCAAGGCCCTTCCCCGGCCTTGCTTCCTCGCCGCTC  100
Wcor15-2A2   ATGGCTTCTTCTTCCGTGCTTCTCGGAGCCTCGGCCACGGCCGCGCTCACCGGCACCCCGGCAGGCAAGGCCCTTCCCCGGCCTTGCTTCCTCGCCGCTC  100

Wcor15-2A    GCCCGCGCACCGTGAGCGGCGGCCGCCTCTGCCTGCAGAACGCTCCAAGGGCGACTCCGGTACGTGTGCCGCGAGCAGTACCGTTCGATCATGGCCTCAT  200
Wcor15-2A1   GCCCGCGCACCGTGAGCGGCGGCCGCCTCTGCCTGCAGAACGCTCCAAGGGCGACTCCGGTACGTGTGCCGCGAGCAGTACCGTTCGATCATGGCCTCAT  200
Wcor15-2A2   GCCCGCGCACCGTGAGCGGCGGCCGTCTCTGCCTGCAGAACGCTCCAAGGGCGACTCCGGTACGTGTGCCGCGAGCAGTACCGTTCGATCATGGCCTCAT  200

Wcor15-2A    GCCGATTATTTTTACGTACGCTGCTGGTTAGTCGTCCTCTGAGATATGCTGACCGAGATATATGGGTGCATGGGACAGGCGTACAACGACGCTGCGGATG  300
Wcor15-2A1   GCCGATTATTTTTACGTACGCTGCTGGTTAGTCGTCCTCTGAGATATGCTGACCGAGATATATGGGTGCATGGGACAGGCGTACAACGACGCTGCGGATG  300
Wcor15-2A2   GCCGATTATTTTTACGTACGCTGCTGGTTAGTCGTCCTCTGAGATATTCTGACCGAGATATATGCATGCATGGG.CAGGCGTACAACGACGCTGCGGATG  299

Wcor15-2A    CTACCGACAAGGCCATCGACGGCGTGAAGGGGGTGGCCGACGAGTTGAAGAAGGGCGTGGCGGAGGCGGCTGAGGCCGTCTCGGGCAACACCGAGAAGGC  400
Wcor15-2A1   CTACCGACAAGGCCATCGACGGCGTGAAGGGGGTGGCCGACGAGTTGAAGAAGGGCGTGGCGGAGGCGGCTGAGGCCGTCTCGGGCAACACCGAGAAGGC  400
Wcor15-2A2   CCACCGACAAGGCCATCGACGGCGTGAAGGGGGTGGCCGACGAGCTGAAGAAGGGCGTGGCGGAGGCGGCGGAGGCCGTCTCGGGCAACACCGAGAAGGC  399

Wcor15-2A    CGCGGAGGAAGCCGGCAAAGGCGCGAGCGAGGTGGACGAGAAGGCCAAGGACTTCGGCGAGCAGGCGAAGAAGGCGACGGAGGAGGCGTGGGACGGCGCC  500
Wcor15-2A1   CGCGGAGGAAGCCGGCAAAGGCGCGAGCGAGGTGGACGAGAAGGCCAAGGACTTCGGCGAGCAGGCGAAGAAGGCGACGGAGGAGGCGTGGGACGGCGCC  500
Wcor15-2A2   CGCGGAGGAAGCCGGCAAGGGCGCGAGCGAGGTCGACGACAAGGCCAAGGACTTCGGCGAGCAGGCGAAGAAGGCGACGGAGGAGGCGTGGGACGGCGCC  499

Wcor15-2A    AAGGACGCCGCACAGGGCATCACGGACAAGGTCGCGGACGCGGCCAAAAAGGAAGCTAACTAA  563
Wcor15-2A1   AAGGACGCCGCACAGGGCATCACGGACAAGGTCGCGGACGCGGCCAAAAAGGAAGCTAACTAA  563
Wcor15-2A2   AAGGACGCCGCACAGGGCATCACGGACAAGGTCGCGGACGCGGCCAAAAAGGAAGCAAACTAA  562
```

Supplementary Fig. S3 Sequence alignments of the *Wcor15-2A* from hexaploid wheat, *Wcor15-2A 1* from *T.urartu* and *Wcor15-2A 2* from *T.m.boeoticum* and *T.m.monococcum*.

Aegilops longissima(Q03-004)	ATCCATCAGCAGTTTTTCTATCGGCCAATGGCTTCTTCTTCCGTGCTGCTCGGAGCCTCG	64
Aegilops bicornis(Q03-021)	ATCCATCAGCAGTTTTTCTATCGGCCAATGGCTTCTTCTTCCGTGCTGCTCGGAGCCTCG	64
Aegilops sharonensis(PI584408)	ATCCATCAGCAGTTTTTCTATCGGCCAATGGCTTCTTCTTCCGTGCTGCTCGGAGCCTCG	64
Aegilops sharonensis(PI584406)	ATCCATCAGCAGTTTTTCTATCGGCCAATGGCTTCTTCTTCCGTGCTGCTCGGAGCCTCG	64
Aegilops sharonensis(PI584395)	ATCCATCAGCAGTTTTTCTATCGGCCAATGGCTTCTTCTTCCGTGCTGCTCGGAGCCTCG	64
Aegilops searsii(PI599142)	ATCCATCAGCAGTTTTTCTATCGGCCAATGGCTTCTTCTTCCGTGCTGCTCGGAGCCTCG	64
Aegilops searsii(PI599124)	ATCCATCAGCAGTTTTTCTATCGGCCAATGGCTTCTTCTTCCGTGCTGCTCGGAGCCTCG	64
Aegilops searsii(PI599126)	ATCCATCAGCAGTTTTTCTATCGGCCAATGGCTTCTTCTTCCGTGCTGCTCGGAGCCTCG	64
Ae speltoides(PI542276)	ATCCATCAGCAGTTTTTCTATCGACCAATGGCTTCTTCTTCCGTGCTGCTCGGAGCCTCG	64
Ae speltoides(PI369663)	ATCCATCAGCAGTTTTTCTATCGACCAATGGCTTCTTCTTCCGTGCTGCTCGGAGCCTCG	64
Ae speltoides(PI369624)	ATCCATCAGCAGTTTTTCTATCGACCAATGGCTTCTTCTTCCGTGCTGCTCGGAGCCTCG	64
Triticum carthlicum(Tc)	ATCCATCAGCAGTTTTTCTATCGACCAATGGCTTCTTCTTCCGTGCTGCTCGGAGCCTCG	960
Triticum durum(Club57)	ATCCATCAGCAGTTTTTCTATCGACCAATGGCTTCTTCTTCCGTGCTGCTCGGAGCCTCG	960
Triticum durum(Dr8)	ATCCATCAGCAGTTTTTCTATCGACCAATGGCTTCTTCTTCCGTGCTGCTCGGAGCCTCG	960
Triticum durum(Simeto-2)	ATCCATCAGCAGTTTTTCTATCGACCAATGGCTTCTTCTTCCGTGCTGCTCGGAGCCTCG	960
Triticum dicoccoides(As829)	ATCCATCAGCAGTTTTTCTATCGACCAATGGCTTCTTCTTCCGTGCTGCTCGGAGCCTCG	960
Triticum dicoccum(PI221401)	ATCCATCAGCAGTTTTTCTATCGACCAATGGCTTCTTCTTCCGTGCTGCTCGGAGCCTCG	960
Triticum dicoccum(PI272527)	ATCCATCAGCAGTTTTTCTATCGACCAATGGCTTCTTCTTCCGTGCTGCTCGGAGCCTCG	960
Triticum dicoccum(PI193873)	ATCCATCAGCAGTTTTTCTATCGACCAATGGCTTCTTCTTCCGTGCTGCTCGGAGCCTCG	960
Triticum dicoccoides(As836)	ATCCATCAGCAGTTTTTCTATCGACCAATGGCTTCTTCTTCCGTGCTGCTCGGAGCCTCG	960
Triticum dicoccoides(As839)	ATCCATCAGCAGTTTTTCTATCGACCAATGGCTTCTTCTTCCGTGCTGCTCGGAGCCTCG	960
Annong 0822	ATCCATCAGCAGTTTTTCTATCGACCAATGGCTTCTTCTTCCGTGCTGCTCGGAGCCTCG	959
Annong 0227	ATCCATCAGCAGTTTTTCTATCGACCAATGGCTTCTTCTTCCGTGCTGCTCGGAGCCTCG	960

Aegilops longissima(Q03-004)	GCCACGGCCGCGCTCACCGGTACCCCGGCAGGCAAGGCCCTTCCCCGGCCTTGCTTCCTC	124
Aegilops bicornis(Q03-021)	GCCACGGCCGCGCTCACCGGTACCCCGGCAGGCAAGGCCCTTCCCCGGCCTTGCTTCCTC	124
Aegilops sharonensis(PI584408)	GCCACGGCCGCGCTCACCGGTACCCCGGCAGGCAAGGCCCTTCCCCGGCCTTGCTTCCTC	124
Aegilops sharonensis(PI584406)	GCCACGGCCGCGCTCACCGGTACCCCGGCAGGCAAGGCCCTTCCCCGGCCTTGCTTCCTC	124
Aegilops sharonensis(PI584395)	GCCACGGCCGCGCTCACCGGTACCCCGGCAGGCAAGGCCCTTCCCCGGCCTTGCTTCCTC	124
Aegilops searsii(PI599142)	GCCACGGCCGCGCTCACCGGTACCCCGGCAGGCAAGGCCCTTCCCCGGCCTTGCTTCCTC	124
Aegilops searsii(PI599124)	GCCACGGCCGCGCTCACCGGTACCCCGGCAGGCAAGGCCCTTCCCCGGCCTTGCTTCCTC	124
Aegilops searsii(PI599126)	GCCACGGCCGCGCTCACCGGTACCCCGGCAGGCAAGGCCCTTCCCCGGCCTTGCTTCCTC	124
Ae speltoides(PI542276)	GCCACGGCCGCGCTCACCGGCACCCCGGCAGGCAAGGCCCTTCCCCGGCCTTGCTTCCTC	124
Ae speltoides(PI369663)	GCCACGGCCGCGCTCACCGGCACCCCGGCAGGCAAGGCCCTTCCCCGGCCTTGCTTCCTC	124
Ae speltoides(PI369624)	GCCACGGCCGCGCTCACCGGCACCCCGGCAGGCAAGGCCCTTCCCCGGCCTTGCTTCCTC	124
Triticum carthlicum(Tc)	GCCACGGCCGCGCTCACCGGCACCCCGGCAGGCAAGGCCCTTCCCCGGCCTTGCTTCCTC	1020
Triticum durum(Club57)	GCCACGGCCGCGCTCACCGGCACCCCGGCAGGCAAGGCCCTTCCCCGGCCTTGCTTCCTC	1020
Triticum durum(Dr8)	GCCACGGCCGCGCTCACCGGCACCCCGGCAGGCAAGGCCCTTCCCCGGCCTTGCTTCCTC	1020
Triticum durum(Simeto-2)	GCCACGGCCGCGCTCACCGGCACCCCGGCAGGCAAGGCCCTTCCCCGGCCTTGCTTCCTC	1020
Triticum dicoccoides(As829)	GCCACGGCCGCGCTCACCGGCACCCCGGCAGGCAAGGCCCTTCCCCGGCCTTGCTTCCTC	1020
Triticum dicoccum(PI221401)	GCCACGGCCGCGCTCACCGGCACCCCGGCAGGCAAGGCCCTTCCCCGGCCTTGCTTCCTC	1020
Triticum dicoccum(PI272527)	GCCACGGCCGCGCTCACCGGCACCCCGGCAGGCAAGGCCCTTCCCCGGCCTTGCTTCCTC	1020
Triticum dicoccum(PI193873)	GCCACGGCCGCGCTCACCGGCACCCCGGCAGGCAAGGCCCTTCCCCGGCCTTGCTTCCTC	1020
Triticum dicoccoides(As836)	GCCACGGCCGCGCTCACCGGCACCCCGGCAGGCAAGGCCCTTCCCCGGCCTTGCTTCCTC	1020
Triticum dicoccoides(As839)	GCCACGGCCGCGCTCACCGGCACCCCGGCAGGCAAGGCCCTTCCCCGGCCTTGCTTCCTC	1020
Annong 0822	GCCACGGCCGCGCTCACCGGCACCCCGGCAGGCAAGGCCCTTCCCCGGCCTTGCTTCCTC	1019
Annong 0227	GCCACGGCCGCGCTCACCGGCACCCCGGCAGGCAAGGCCCTTCCCCGGCCTTGCTTCCTC	1020

Aegilops longissima(Q03-004)	GCCGCTCGCCCGCGCACCGTGAGCGGTGGCCGCCTCTGCCTGCAGAACGCTCCAAGGGCG	184
Aegilops bicornis(Q03-021)	GCCGCTCGCCCGCGCACCGTGAGCGGTGGCCGCCTCTGCCTGCAGAACGCTCCAAGGGCG	184
Aegilops sharonensis(PI584408)	GCCGCTCGCCCGCGCACCGTGAGCGGTGGCCGCCTCTGCCTGCAGAACGCTCCAAGGGCG	184
Aegilops sharonensis(PI584406)	GCCGCTCGCCCGCGCACCGTGAGCGGTGGCCGCCTCTGCCTGCAGAACGCTCCAAGGGCG	184
Aegilops sharonensis(PI584395)	GCCGCTCGCCCGCGCACCGTGAGCGGTGGCCGCCTCTGCCTGCAGAACGCTCCAAGGGCG	184
Aegilops searsii(PI599142)	GCCGCTCGCCCGCGCACCGTGAGCGGTGGCCGCCTCTGCCTGCAGAACGCTCCAAGGGCG	184
Aegilops searsii(PI599124)	GCCGCTCGCCCGCGCACCGTGAGCGGTGGCCGCCTCTGCCTGCAGAACGCTCCAAGGGCG	184
Aegilops searsii(PI599126)	GCCGCTCGCCCGCGCACCGTGAGCGGTGGCCGCCTCTGCCTGCAGAACGCTCCAAGGGCG	184
Ae speltoides(PI542276)	GCCGCTCGCCCGCGCACCGTGAGCGGTGGCCGTCTCTGCCTGCAGAACGCTCCAAGGGCG	184
Ae speltoides(PI369663)	GCCGCTCGCCCGCGCACCGTGAGCGGTGGCCGTCTCTGCCTGCAGAACGCTCCAAGGGCG	184
Ae speltoides(PI369624)	GCCGCTCGCCCGCGCACCGTGAGCGGTGGCCGTCTCTGCCTGCAGAACGCTCCAAGGGCG	184
Triticum carthlicum(Tc)	GCCGCTCGCCCGCGCACCGTGAGCGGTGGCCGTCTCTGCCTGCAGAACGCTCCAAGGGCG	1080
Triticum durum(Club57)	GCCGCTCGCCCGCGCACCGTGAGCGGTGGCCGTCTCTGCCTGCAGAACGCTCCAAGGGCG	1080
Triticum durum(Dr8)	GCCGCTCGCCCGCGCACCGTGAGCGGTGGCCGTCTCTGCCTGCAGAACGCTCCAAGGGCG	1080
Triticum durum(Simeto-2)	GCCGCTCGCCCGCGCACCGTGAGCGGTGGCCGTCTCTGCCTGCAGAACGCTCCAAGGGCG	1080
Triticum dicoccoides(As829)	GCCGCTCGCCCGCGCACCGTGAGCGGTGGCCGTCTCTGCCTGCAGAACGCTCCAAGGGCG	1080
Triticum dicoccum(PI221401)	GCCGCTCGCCCGCGCACCGTGAGCGGTGGCCGTCTCTGCCTGCAGAACGCTCCAAGGGCG	1080
Triticum dicoccum(PI272527)	GCCGCTCGCCCGCGCACCGTGAGCGGTGGCCGTCTCTGCCTGCAGAACGCTCCAAGGGCG	1080
Triticum dicoccum(PI193873)	GCCGCTCGCCCGCGCACCGTGAGCGGTGGCCGTCTCTGCCTGCAGAACGCTCCAAGGGCG	1080
Triticum dicoccoides(As836)	GCCGCTCGCCCGCGCACCGTGAGCGGTGGCCGTCTCTGCCTGCAGAACGCTCCAAGGGCG	1080
Triticum dicoccoides(As839)	GCCGCTCGCCCGCGCACCGTGAGCGGTGGCCGTCTCTGCCTGCAGAACGCTCCAAGGGCG	1080
Annong 0822	GCCGCTCGCCCGCGCACCGTGAGCGGTGGCCGTCTCTGCCTGCAGAACGCTCCAAGGGCG	1079
Annong 0227	GCCGCTCGCCCGCGCACCGTGAGCGGTGGCCGTCTCTGCCTGCAGAACGCTCCAAGGGCG	1080

```
Aegilops longissima(Q03-004)      ACTCCGGTACGTGTGTCCCGAGCAGTACCGTTCGATCATGACTTCATGCCGATTATTTTA   244
Aegilops bicornis(Q03-021)        ACTCCGGTACGTGTGTCCCGAGCAGTACCGTTCGATCATGACTTCATGCCGATTATTTTA   244
Aegilops sharonensis(PI584408)    ACTCCGGTACGTGTGTCCCGAGCAGTACCGTTCGATCATGACTTCATGCCGATTATTTTA   244
Aegilops sharonensis(PI584406)    ACTCCGGTACGTGTGTCCCGAGCAGTACCGTTCGATCATGACTTCATGCCGATTATTTTA   244
Aegilops sharonensis(PI584395)    ACTCCGGTACGTGTGTCCCGAGCAGTACCGTTCGATCATGACTTCATGCCGATTATTTTA   244
Aegilops searsii(PI599142)        ACTCCGGTACGTGTGTCCCGAGCAGTACCGTTCGATCATGACTTCATGCCGATTATTTTA   244
Aegilops searsii(PI599124)        ACTCCGGTACGTGTGTCCCGAGCAGTACCGTTCGATCATGACTTCATGCCGATTATTTTA   244
Aegilops searsii(PI599126)        ACTCCGGTACGTGTGTCCCGAGCAGTACCGTTCGATCATGACTTCATGCCGATTATTTTA   244
Ae speltoides(PI542276)           ACTCCGGTACGTGT.TCGCGAGCGGTACCGTTCGATCATGACTTCATGCCGATTGTTTTA   243
Ae speltoides(PI369663)           ACTCCGGTACGTGT.TCGCGAGCGGTACCGTTCGATCATGACTTCATGCCGATTGTTTTA   243
Ae speltoides(PI369624)           ACTCCGGTACGTGT.TCGCGAGCGGTACCGTTCGATCATGACTTCATGCCGATTGTTTTA   243
Triticum carthlicum(Tc)           ACTCCGGTACGTGT.TCGCGAGCGGTACCGTTCGATCATGACTTCATGCCGATTGTTTTA  1139
Triticum durum(Club57)            ACTCCGGTACGTGT.TCGCGAGCGGTACCGTTCGATCATGACTTCATGCCGATTGTTTTA  1139
Triticum durum(Dr8)               ACTCCGGTACGTGT.TCGCGAGCGGTACCGTTCGATCATGACTTCATGCCGATTGTTTTA  1139
Triticum durum(Simeto-2)          ACTCCGGTACGTGT.TCGCGAGCGGTACCGTTCGATCATGACTTCATGCCGATTGTTTTA  1139
Triticum dicoccoides(As829)       ACTCCGGTACGTGT.TCGCGAGCGGTACCGTTCGATCATGACTTCATGCCGATTGTTTTA  1139
Triticum dicoccum(PI221401)       ACTCCGGTACGTGT.TCGCGAGCGGTACCGTTCGATCATGACTTCATGCCGATTGTTTTA  1139
Triticum dicoccum(PI272527)       ACTCCGGTACGTGT.TCGCGAGCGGTACCGTTCGATCATGACTTCATGCCGATTGTTTTA  1139
Triticum dicoccum(PI193873)       ACTCCGGTACGTGTGTCGCGAGCGGTACCGTTCGATCATGACTTCATGCCGATTGTTTTA  1140
Triticum dicoccoides(As836)       ACTCCGGTACGTGTGTCGCGAGCGGTACCGTTCGATCATGACTTCATGCCGATTGTTTTA  1140
Triticum dicoccoides(As839)       ACTCCGGTACGTGTGTCGCGAGCGGTACCGTTCGATCATGACTTCATGCCGATTGTTTTA  1140
Annong 0822                       ACTCCGGTACGTGTGTCGCGAGCGGTACCGTTCGATCATGACTTCATGCCGATTGTTTTA  1139
Annong 0227                       ACTCCGGTACGTGT.TCGCGAGCGGTACCGTTCGATCATGACTTCATGCCGATTGTTTTA  1139

Aegilops longissima(Q03-004)      CGTACGCTGCTGGTTAATCAATCGTTCTATGGGATACGCTGACCGAGATATATGCGTGCA   304
Aegilops bicornis(Q03-021)        CGTACGCTGCTGGTTAATCAATCGTTCTATGGGATACGCTGACCGAGATATATGCGTGCA   304
Aegilops sharonensis(PI584408)    CGTACGCTGCTGGTTAATCAATCGTTCTATGGGATACGCTGACCGAGATATATGCGTGCA   304
Aegilops sharonensis(PI584406)    CGTACGCTGCTGGTTAATCAATCGTTCTATGGGATACGCTGACCGAGATATATGCGTGCA   304
Aegilops sharonensis(PI584395)    CGTACGCTGCTGGTTAATCAATCGTTCTATGGGATACGCTGACCGAGATATATGCGTGCA   304
Aegilops searsii(PI599142)        CGTACGCTGCTGGTTAATCAATCGTTCTATGGGATACGCTGACCGAGATATATGCGTGCA   304
Aegilops searsii(PI599124)        CGTACGCTGCTGGTTAATCAATCGTTCTATGGGATACGCTGACCGAGATATATGCGTGCA   304
Aegilops searsii(PI599126)        CGTACGCTGCTGGTTAATCAATCGTTCTATGGGATACGCTGACCGAGATATATGCGTGCA   304
Ae speltoides(PI542276)           CCTACGCTGCTGGTTAATCAATCGTTCCATGGGATATGCTGACCGAGATGTATGCGTGCA   303
Ae speltoides(PI369663)           CCTACGCTGCTGGTTAATCAATCGTTCCATGGGATATGCTGACCGAGATGTATGCGTGCA   303
Ae speltoides(PI369624)           CCTACGCTGCTGGTTAATCAATCGTTCCATGGGATATGCTGACCGAGATGTATGCGTGCA   303
Triticum carthlicum(Tc)           CCTACGCTGCTGGTTAATCAATCGTTCCATGGGATATGCTGACCGAGATGTATGCGTGCA  1199
Triticum durum(Club57)            CCTACGCTGCTGGTTAATCAATCGTTCCATGGGATATGCTGACCGAGATGTATGCGTGCA  1199
Triticum durum(Dr8)               CCTACGCTGCTGGTTAATCAATCGTTCCATGGGATATGCTGACCGAGATGTATGCGTGCA  1199
Triticum durum(Simeto-2)          CCTACGCTGCTGGTTAATCAATCGTTCCATGGGATATGCTGACCGAGATGTATGCGTGCA  1199
Triticum dicoccoides(As829)       CCTACGCTGCTGGTTAATCAATCGTTCCATGGGATATGCTGACCGAGATGTATGCGTGCA  1199
Triticum dicoccum(PI221401)       CCTACGCTGCTGGTTAATCAATCGTTCCATGGGATATGCTGACCGAGATGTATGCGTGCA  1199
Triticum dicoccum(PI272527)       CCTACGCTGCTGGTTAATCAATCGTTCCATGGGATATGCTGACCGAGATGTATGCGTGCA  1199
Triticum dicoccum(PI193873)       CCTACGCTGCTGGTTAATCAATCGTTCCATGGGATATGCTGACCGAGATGTATGCGTGCA  1200
Triticum dicoccoides(As836)       CCTACGCTGCTGGTTAATCAATCGTTCCATGGGATATGCTGACCGAGATGTATGCGTGCA  1200
Triticum dicoccoides(As839)       CCTACGCTGCTGGTTAATCAATCGTTCCATGGGATATGCTGACCGAGATGTATGCGTGCA  1200
Annong 0822                       CCTACGCTGCTGGTTAATCAATCGTTCCATGGGATATGCTGACCGAGATGTATGCGTGCA  1199
Annong 0227                       CCTACGCTGCTGGTTAATCAATCGTTCCATGGGATATGCTGACCGAGATGTATGCGTGCA  1199

Aegilops longissima(Q03-004)      TGGGACAGGCGTACAACGACGCTGCGGATGCCACCGACAAGGCCATCGAGGGCGTGAAGG   364
Aegilops bicornis(Q03-021)        TGGGACAGGCGTACAACGACGCTGCGGATGCCACCGACAAGGCCATCGAGGGCGTGAAGG   364
Aegilops sharonensis(PI584408)    TGGGACAGGCGTACAACGACGCTGCGGATGCCACCGACAAGGCCATCGAGGGCGTGAAGG   364
Aegilops sharonensis(PI584406)    TGGGACAGGCGTACAACGACGCTGCGGATGCCACCGACAAGGCCATCGAGGGCGTGAAGG   364
Aegilops sharonensis(PI584395)    TGGGACAGGCGTACAACGACGCTGCGGATGCCACCGACAAGGCCATCGAGGGCGTGAAGG   364
Aegilops searsii(PI599142)        TGGGACAGGCGTACAACGACGCTGCGGATGCCACCGACAAGGCCATCGAGGGCGTGAAGG   364
Aegilops searsii(PI599124)        TGGGACAGGCGTACAACGACGCTGCGGATGCCACCGACAAGGCCATCGAGGGCGTGAAGG   364
Aegilops searsii(PI599126)        TGGGACAGGCGTACAACGACGCTGCGGATGCCACCGACAAGGCCATCGAGGGCGTGAAGG   364
Ae speltoides(PI542276)           TGGGACAGGCGTACAACGACGCTGCGGATGCCACCGACAAGGCCATCGACGGCGTGAAGG   363
Ae speltoides(PI369663)           TGGGACAGGCGTACAACGACGCTGCGGATGCCACCGACAAGGCCATCGACGGCGTGAAGG   363
Ae speltoides(PI369624)           TGGGACAGGCGTACAACGACGCTGCGGATGCCACCGACAAGGCCATCGACGGCGTGAAGG   363
Triticum carthlicum(Tc)           TGGGACAGGCGTACAACGACGCTGCGGATGCCACCGACAAGGCCATCGACGGCGTGAAGG  1259
Triticum durum(Club57)            TGGGACAGGCGTACAACGACGCTGCGGATGCCACCGACAAGGCCATCGACGGCGTGAAGG  1259
Triticum durum(Dr8)               TGGGACAGGCGTACAACGACGCTGCGGATGCCACCGACAAGGCCATCGACGGCGTGAAGG  1259
Triticum durum(Simeto-2)          TGGGACAGGCGTACAACGACGCTGCGGATGCCACCGACAAGGCCATCGACGGCGTGAAGG  1259
Triticum dicoccoides(As829)       TGGGACAGGCGTACAACGACGCTGCGGATGCCACCGACAAGGCCATCGACGGCGTGAAGG  1259
Triticum dicoccum(PI221401)       TGGGACAGGCGTACAACGACGCTGCGGATGCCACCGACAAGGCCATCGACGGCGTGAAGG  1259
Triticum dicoccum(PI272527)       TGGGACAGGCGTACAACGACGCTGCGGATGCCACCGACAAGGCCATCGACGGCGTGAAGG  1259
Triticum dicoccum(PI193873)       TGGGACAGGCGTACAACGACGCTGCGGATGCCACCGACAAGGCCATCGACGGCGTGAAGG  1260
Triticum dicoccoides(As836)       TGGGACAGGCGTACAACGACGCTGCGGATGCCACCGACAAGGCCATCGACGGCGTGAAGG  1260
Triticum dicoccoides(As839)       TGGGACAGGCGTACAACGACGCTGCGGATGCCACCGACAAGGCCATCGACGGCGTGAAGG  1260
Annong 0822                       TGGGACAGGCGTACAACGACGCTGCGGATGCCACCGACAAGGCCATCGACGGCGTGAAGG  1259
Annong 0227                       TGGGACAGGCGTACAACGACGCTGCGGATGCCACCGACAAGGCCATCGACGGCGTGAAGG  1259
```

```
Aegilops longissima(Q03-004)     GGGTGGCCGACGAGCTGAAGAAGGGCGTGGCGGAGGCGGCGGAGGCCGTCTCGGGCAACA   424
Aegilops bicornis(Q03-021)       GGGTGGCCGACGAGCTGAAGAAGGGCGTGGCGGAGGCGGCGGAGGCCGTCTCGGGCAACA   424
Aegilops sharonensis(PI584408)   GGGTGGCCGACGAGCTGAAGAAGGGCGTGGCGGAGGCGGCGGAGGCCGTCTCGGGCAACA   424
Aegilops sharonensis(PI584406)   GGGTGGCCGACGAGCTGAAGAAGGGCGTGGCGGAGGCGGCGGAGGCCGTCTCGGGCAACA   424
Aegilops sharonensis(PI584395)   GGGTGGCCGACGAGCTGAAGAAGGGCGTGGCGGAGGCGGCGGAGGCCGTCTCGGGCAACA   424
Aegilops searsii(PI599142)       GGGTGGCCGACGAGCTGAAGAAGGGCGTGGCGGAGGCGGCGGAGGCCGTCTCGGGCAACA   424
Aegilops searsii(PI599124)       GGGTGGCCGACGAGCTGAAGAAGGGCGTGGCGGAGGCGGCGGAGGCCGTCTCGGGCAACA   424
Aegilops searsii(PI599126)       GGGTGGCCGACGAGCTGAAGAAGGGCGTGGCGGAGGCGGCGGAGGCCGTCTCGGGCAACA   424
Ae speltoides(PI542276)          GGGTGGCCGACGAGTTGAAGAAGGGCGTGGCGGAGGCTGCGGAGGCCGTCTCGGGCAACA   423
Ae speltoides(PI369663)          GGGTGGCCGACGAGTTGAAGAAGGGCGTGGCGGAGGCTGCGGAGGCCGTCTCGGGCAACA   423
Ae speltoides(PI369624)          GGGTGGCCGACGAGTTGAAGAAGGGCGTGGCGGAGGCTGCGGAGGCCGTCTCGGGCAACA   423
Triticum carthlicum(Tc)          GGGTGGCCGACGAGTTGAAGAAGGGCGTGGCGGAGGCTGCGGAGGCCGTCTCGGGCAACA  1319
Triticum durum(Club57)           GGGTGGCCGACGAGTTGAAGAAGGGCGTGGCGGAGGCTGCGGAGGCCGTCTCGGGCAACA  1319
Triticum durum(Dr8)              GGGTGGCCGACGAGTTGAAGAAGGGCGTGGCGGAGGCTGCGGAGGCCGTCTCGGGCAACA  1319
Triticum durum(Simeto-2)         GGGTGGCCGACGAGTTGAAGAAGGGCGTGGCGGAGGCTGCGGAGGCCGTCTCGGGCAACA  1319
Triticum dicoccoides(As829)      GGGTGGCCGACGAGTTGAAGAAGGGCGTGGCGGAGGCTGCGGAGGCCGTCTCGGGCAACA  1319
Triticum dicoccum(PI221401)      GGGTGGCCGACGAGTTGAAGAAGGGCGTGGCGGAGGCTGCGGAGGCCGTCTCGGGCAACA  1319
Triticum dicoccum(PI272527)      GGGTGGCCGACGAGTTGAAGAAGGGCGTGGCGGAGGCTGCGGAGGCCGTCTCGGGCAACA  1319
Triticum dicoccum(PI193873)      GGGTGGCCGACGAGTTGAAGAAGGGCGTGGCGGAGGCTGCGGAGGCCGTCTCGGGCAACA  1320
Triticum dicoccoides(As836)      GGGTGGCCGACGAGTTGAAGAAGGGCGTGGCGGAGGCTGCGGAGGCCGTCTCGGGCAACA  1320
Triticum dicoccoides(As839)      GGGTGGCCGACGAGTTGAAGAAGGGCGTGGCGGAGGCTGCGGAGGCCGTCTCGGGCAACA  1320
Annong 0822                      GGGTGGCCGACGAGTTGAAGAAGGGCGTGGCGGAGGCTGCGGAGGCCGTCTCGGGCAACA  1319
Annong 0227                      GGGTGGCCGACGAGTTGAAGAAGGGCGTGGCGGAGGCTGCGGAGGCCGTCTCGGGCAACA  1319

Aegilops longissima(Q03-004)     CCGAGAAGGCCGCGGAGGAAGCCGGCAAGGGCGCGAGCGAGGTGGACGACAAGGCCAAGG   484
Aegilops bicornis(Q03-021)       CCGAGAAGGCCGCGGAGGAAGCCGGCAAGGGCGCGAGCGAGGTGGACGACAAGGCCAAGG   484
Aegilops sharonensis(PI584408)   CCGAGAAGGCCGCGGAGGAAGCCGGCAAGGGCGCGAGCGAGGTGGACGACAAGGCCAAGG   484
Aegilops sharonensis(PI584406)   CCGAGAAGGCCGCGGAGGAAGCCGGCAAGGGCGCGAGCGAGGTGGACGACAAGGCCAAGG   484
Aegilops sharonensis(PI584395)   CCGAGAAGGCCGCGGAGGAAGCCGGCAAGGGCGCGAGCGAGGTGGACGACAAGGCCAAGG   484
Aegilops searsii(PI599142)       CCGAGAAGGCCGCGGAGGAAGCCGGCAAGGGCGCGAGCGAGGTGGACGACAAGGCCAAGG   484
Aegilops searsii(PI599124)       CCGAGAAGGCCGCGGAGGAAGCCGGCAAGGGCGCGAGCGAGGTGGACGACAAGGCCAAGG   484
Aegilops searsii(PI599126)       CCGAGAAGGCCGCGGAGGAAGCCGGCAAGGGCGCGAGCGAGGTGGACGACAAGGCCAAGG   484
Ae speltoides(PI542276)          CCGAGAAGGCCGCGGAGGAAGCCGGCAAGGGCGCGAGCGAGGTGGACGCGAAGGCCAAGG   483
Ae speltoides(PI369663)          CCGAGAAGGCCGCGGAGGAAGCCGGCAAGGGCGCGAGCGAGGTGGACGCGAAGGCCAAGG   483
Ae speltoides(PI369624)          CCGAGAAGGCCGCGGAGGAAGCCGGCAAGGGCGCGAGCGAGGTGGACGCGAAGGCCAAGG   483
Triticum carthlicum(Tc)          CCGAGAAGGCCGCGGAGGAAGCCGGCAAGGGCGCGAGCGAGGTGGACGCGAAGGCCAAGG  1379
Triticum durum(Club57)           CCGAGAAGGCCGCGGAGGAAGCCGGCAAGGGCGCGAGCGAGGTGGACGCGAAGGCCAAGG  1379
Triticum durum(Dr8)              CCGAGAAGGCCGCGGAGGAAGCCGGCAAGGGCGCGAGCGAGGTGGACGCGAAGGCCAAGG  1379
Triticum durum(Simeto-2)         CCGAGAAGGCCGCGGAGGAAGCCGGCAAGGGCGCGAGCGAGGTGGACGCGAAGGCCAAGG  1379
Triticum dicoccoides(As829)      CCGAGAAGGCCGCGGAGGAAGCCGGCAAGGGCGCGAGCGAGGTGGACGCGAAGGCCAAGG  1379
Triticum dicoccum(PI221401)      CCGAGAAGGCCGCGGAGGAAGCCGGCAAGGGCGCGAGCGAGGTGGACGCGAAGGCCAAGG  1379
Triticum dicoccum(PI272527)      CCGAGAAGGCCGCGGAGGAAGCCGGCAAGGGCGCGAGCGAGGTGGACGCGAAGGCCAAGG  1379
Triticum dicoccum(PI193873)      CCGAGAAGGCCGCGGAGGAAGCCGGCAAGGGCGCGAGCGAGGTGGACGCGAAGGCCAAGG  1380
Triticum dicoccoides(As836)      CCGAGAAGGCCGCGGAGGAAGCCGGCAAGGGCGCGAGCGAGGTGGACGCGAAGGCCAAGG  1380
Triticum dicoccoides(As839)      CCGAGAAGGCCGCGGAGGAAGCCGGCAAGGGCGCGAGCGAGGTGGACGCGAAGGCCAAGG  1380
Annong 0822                      CCGAGAAGGCCGCGGAGGAAGCCGGCAAGGGCGCGAGCGAGGTGGACGCGAAGGCCAAGG  1379
Annong 0227                      CCGAGAAGGCCGCGGAGGAAGCCGGCAAGGGCGCGAGCGAGGTGGACGCGAAGGCCAAGG  1379

Aegilops longissima(Q03-004)     ACTTCGGCGAGCAGGCGAAGAAGGCGACGGAGGAGGCGTGGGACGGCGCCAAGGACGCCG   544
Aegilops bicornis(Q03-021)       ACTTCGGCGAGCAGGCGAAGAAGGCGACGGAGGAGGCGTGGGACGGCGCCAAGGACGCCG   544
Aegilops sharonensis(PI584408)   ACTTCGGCGAGCAGGCGAAGAAGGCGACGGAGGAGGCGTGGGACGGCGCCAAGGACGCCG   544
Aegilops sharonensis(PI584406)   ACTTCGGCGAGCAGGCGAAGAAGGCGACGGAGGAGGCGTGGGACGGCGCCAAGGACGCCG   544
Aegilops sharonensis(PI584395)   ACTTCGGCGAGCAGGCGAAGAAGGCGACGGAGGAGGCGTGGGACGGCGCCAAGGACGCCG   544
Aegilops searsii(PI599142)       ACTTCGGCGAGCAGGCGAAGAAGGCGACGGAGGAGGCGTGGGACGGCGCCAAGGACGCCG   544
Aegilops searsii(PI599124)       ACTTCGGCGAGCAGGCGAAGAAGGCGACGGAGGAGGCGTGGGACGGCGCCAAGGACGCCG   544
Aegilops searsii(PI599126)       ACTTCGGCGAGCAGGCGAAGAAGGCGACGGAGGAGGCGTGGGACGGCGCCAAGGACGCCG   544
Ae speltoides(PI542276)          ACTTCGGCGAGCAGGCGAAGAAGGCGACGGAGGAGGCGTGGGACGGCGCCAAGGACGCCG   543
Ae speltoides(PI369663)          ACTTCGGCGAGCAGGCGAAGAAGGCGACGGAGGAGGCGTGGGACGGCGCCAAGGACGCCG   543
Ae speltoides(PI369624)          ACTTCGGCGAGCAGGCGAAGAAGGCGACGGAGGAGGCGTGGGACGGCGCCAAGGACGCCG   543
Triticum carthlicum(Tc)          ACTTCGGCGAGCAGGCGAAGAAGGCGACGGAGGAGGCGTGGGACGGCGCCAAGGACGCCG  1439
Triticum durum(Club57)           ACTTCGGCGAGCAGGCGAAGAAGGCGACGGAGGAGGCGTGGGACGGCGCCAAGGACGCCG  1439
Triticum durum(Dr8)              ACTTCGGCGAGCAGGCGAAGAAGGCGACGGAGGAGGCGTGGGACGGCGCCAAGGACGCCG  1439
Triticum durum(Simeto-2)         ACTTCGGCGAGCAGGCGAAGAAGGCGACGGAGGAGGCGTGGGACGGCGCCAAGGACGCCG  1439
Triticum dicoccoides(As829)      ACTTCGGCGAGCAGGCGAAGAAGGCGACGGAGGAGGCGTGGGACGGCGCCAAGGACGCCG  1439
Triticum dicoccum(PI221401)      ACTTCGGCGAGCAGGCGAAGAAGGCGACGGAGGAGGCGTGGGACGGCGCCAAGGACGCCG  1439
Triticum dicoccum(PI272527)      ACTTCGGCGAGCAGGCGAAGAAGGCGACGGAGGAGGCGTGGGACGGCGCCAAGGACGCCG  1439
Triticum dicoccum(PI193873)      ACTTCGGCGAGCAGGCGAAGAAGGCGACGGAGGAGGCGTGGGACGGCGCCAAGGACGCCG  1440
Triticum dicoccoides(As836)      ACTTCGGCGAGCAGGCGAAGAAGGCGACGGAGGAGGCGTGGGACGGCGCCAAGGACGCCG  1440
Triticum dicoccoides(As839)      ACTTCGGCGAGCAGGCGAAGAAGGCGACGGAGGAGGCGTGGGACGGCGCCAAGGACGCCG  1440
Annong 0822                      ACTTCGGCGAGCAGGCGAAGAAGGCGACGGAGGAGGCGTGGGACGGCGCCAAGGACGCCG  1439
Annong 0227                      ACTTCGGCGAGCAGGCGAAGAAGGCGACGGAGGAGGCGTGGGACGGCGCCAAGGACGCCG  1439
```

```
Aegilops longissima(Q03-004)        CACAGGGCATCACGGACAA.........................................   563
Aegilops bicornis(Q03-021)          CACAGGGCATCACGGACAA.........................................   563
Aegilops sharonensis(PI584408)      CACAGGGCATCACGGACAA.........................................   563
Aegilops sharonensis(PI584406)      CACAGGGCATCACGGACAA.........................................   563
Aegilops sharonensis(PI584395)      CACAGGGCATCACGGACAA.........................................   563
Aegilops searsii(PI599142)          CACAGGGCATCACGGACAA.........................................   563
Aegilops searsii(PI599124)          CACAGGGCATCACGGACAA.........................................   563
Aegilops searsii(PI599126)          CACAGGGCATCACGGACAA.........................................   563
Ae speltoides(PI542276)             CACAGGGCATCACGGACAA.........................................   562
Ae speltoides(PI369663)             CACAGGGCATCACGGACAA.........................................   562
Ae speltoides(PI369624)             CACAGGGCATCACGGACAA.........................................   562
Triticum carthlicum(Tc)             CACAGGGCATCACGGACAAAGTCGCCGCCGCGGCCAAAAAGGAAGCTAGCTAAGCTAACA  1499
Triticum durum(Club57)              CACAGGGCATCACGGACAAAGTCGCCGCCGCGGCCAAAAAGGAAGCTAGCTAAGCTAACA  1499
Triticum durum(Dr8)                 CACAGGGCATCACGGACAAAGTCGCCGCCGCGGCCAAAAAGGAAGCTAGCTAAGCTAACA  1499
Triticum durum(Simeto-2)            CACAGGGCATCACGGACAAAGTCGCCGCCGCGGCCAAAAAGGAAGCTAGCTAAGCTAACA  1499
Triticum dicoccoides(As829)         CACAGGGCATCACGGACAAAGTCGCCGCCGCGGCCAAAAAGGAAGCTAGCTAAGCTAACA  1499
Triticum dicoccum(PI221401)         CACAGGGCATCACGGACAAAGTCGCCGCCGCGGCCAAAAAGGAAGCTAGCTAAGCTAACA  1499
Triticum dicoccum(PI272527)         CACAGGGCATCACGGACAAAGTCGCCGCCGCGGCCAAAAAGGAAGCTAGCTAAGCTAACA  1499
Triticum dicoccum(PI193873)         CACAGGGCATCACGGACAAAGTCGCCGCCGCGGCCAAAAAGGAAGCTAGCTAAGCTAACA  1500
Triticum dicoccoides(As836)         CACAGGGCATCACGGACAAAGTCGCCGCCGCGGCCAAAAAGGAAGCTAGCTAAGCTAACA  1500
Triticum dicoccoides(As839)         CACAGGGCATCACGGACAAAGTCGCCGCCGCGGCCAAAAAGGAAGCTAGCTAAGCTAACA  1500
Annong 0822                         CACAGGGCATCACGGACAAAGTCGCCGCCGCGGCCAAAAAGGAAGCTAGCTAAGCTAACA  1499
Annong 0227                         CACAGGGCATCACGGACAAAGTCGCCGCCGCGGCCAAAAAGGAAGCTAGCTAAGCTAACA  1499
```

Supplementary Fig. S4 Sequence alignments of the *Wcor15-2B* from 2 hexaploid wheat Annong 0227 and Annong 0822, 10 tetraploid wheat, and *Wcor15* from 1 *Ae. bicornis* S^bS^b, 1 *Ae. longissima* S^1S^1, 3 *Ae. sharonensis* $S^{sh}S^{sh}$, 3 *Ae. Searsii* S^sS^s and 3 *Ae. speltoides* SS. The blue parts are exons. Annong 0227 stands for all the members of the *Wcor15-2B* with the lack of a base G in introns from tetraploid and hexaploid wheat. Annong 0822 stands for all the members of the *Wcor15-2B* with a base G inserted in the introns ninth base from tetraploid and hexaploid wheat.

```
Triticum_boeoticum_boiss_Bo8    ............................................................    0
Triticum_monococcum_linn_Mo4    ............................................................    0
Triticum_monococcum_linn_TL     ............................................................    0
Triticum_urartu_PI428222        CCTTCTCATCCATCATAGCAGCTGACATCTTTTGAAGGCTCTCCTTGAGAGCAAATATAA   60
Triticum_urartu_PI428260        CCTTCTCATCCATCATAGCAGCTGACATCTTTTGAAGGCTCTCCTTGAGAGCAAATATAA   60
Triticum_urartu_PI428266        CCTTCTCATCCATCATAGCAGCTGACATCTTTTGAAGGCTCTCCTTGAGAGCAAATATAA   60
T.dicoccoides_As829             CCTTCTCATCCATCATAGCAGCTGACATCTTTTGAAGGCTCTCCTTGAGAGCAAATATAA   60
T.dicoccoides_As836             CCTTCTCATCCATCATAGCAGCTGACATCTTTTGAAGGCTCTCCTTGAGAGCAAATATAA   60
T.dicoccoides_As839             CCTTCTCATCCATCATAGCAGCTGACATCTTTTGAAGGCTCTCCTTGAGAGCAAATATAA   60
T.dicoccum_PI272527             CCTTCTCATCCATCATAGCAGCTGACATCTTTTGAAGGCTCTCCTTGAGAGCAAATATAA   60
T.dicoccum_PI193873             CCTTCTCATCCATCATAGCAGCTGACATCTTTTGAAGGCTCTCCTTGAGAGCAAATATAA   60
T.dicoccum_PI272527             CCTTCTCATCCATCATAGCAGCTGACATCTTTTGAAGGCTCTCCTTGAGAGCAAATATAA   60
T.durum_Dr8                     CCTTCTCATCCATCATAGCAGCTGACATCTTTTGAAGGCTCTCCTTGAGAGCAAATATAA   60
T.durum_Simeto-2                CCTTCTCATCCATCATAGCAGCTGACATCTTTTGAAGGCTCTCCTTGAGAGCAAATATAA   60
T.durum_Club57                  CCTTCTCATCCATCATAGCAGCTGACATCTTTTGAAGGCTCTCCTTGAGAGCAAATATAA   60
T.carthlicum_Tc                 CCTTCTCATCCATCATAGCAGCTGACATCTTTTGAAGGCTCTCCTTGAGAGCAAATATAA   60
Hexaploid wheat                 CCTTCTCATCCATCATAGCAGCTGACATCTTTTGAAGGCTCTCCTTGAGAGCAAATATAA   60

Triticum_boeoticum_boiss_Bo8    ............................................................    0
Triticum_monococcum_linn_Mo4    ............................................................    0
Triticum_monococcum_linn_TL     ............................................................    0
Triticum_urartu_PI428222        TAAGTGATGTAAGCGAGATGTAAGACTTTCAATATAATATTTTTAATGAGTTGAAAGAGA  120
Triticum_urartu_PI428260        TAAGTGATGTAAGCGAGATGTAAGACTTTCAATATAATATTTTTAATGAGTTGAAAGAGA  120
Triticum_urartu_PI428266        TAAGTGATGTAAGCGAGATGTAAGACTTTCAATATAATATTTTTAATGAGTTGAAAGAGA  120
T.dicoccoides_As829             TAAGTGATGTAAGCGAGATGTAAGACTTTCAATATAATATTTTTAATGAGTTGAAAGAGA  120
T.dicoccoides_As836             TAAGTGATGTAAGCGAGATGTAAGACTTTCAATATAATATTTTTAATGAGTTGAAAGAGA  120
T.dicoccoides_As839             TAAGTGATGTAAGCGAGATGTAAGACTTTCAATATAATATTTTTAATGAGTTGAAAGAGA  120
T.dicoccum_PI272527             TAAGTGATGTAAGCGAGATGTAAGACTTTCAATATAATATTTTTAATGAGTTGAAAGAGA  120
T.dicoccum_PI193873             TAAGTGATGTAAGCGAGATGTAAGACTTTCAATATAATATTTTTAATGAGTTGAAAGAGA  120
T.dicoccum_PI272527             TAAGTGATGTAAGCGAGATGTAAGACTTTCAATATAATATTTTTAATGAGTTGAAAGAGA  120
T.durum_Dr8                     TAAGTGATGTAAGCGAGATGTAAGACTTTCAATATAATATTTTTAATGAGTTGAAAGAGA  120
T.durum_Simeto-2                TAAGTGATGTAAGCGAGATGTAAGACTTTCAATATAATATTTTTAATGAGTTGAAAGAGA  120
T.durum_Club57                  TAAGTGATGTAAGCGAGATGTAAGACTTTCAATATAATATTTTTAATGAGTTGAAAGAGA  120
T.carthlicum_Tc                 TAAGTGATGTAAGCGAGATGTAAGACTTTCAATATAATATTTTTAATGAGTTGAAAGAGA  120
Hexaploid_wheat                 TAAGTGATGTAAGCGAGATGTAAGACTTTCAATATAATATTTTTAATGAGTTGAAAGAGA  120

Triticum_boeoticum_boiss_Bo8    ............................................................    0
Triticum_monococcum_linn_Mo4    ............................................................    0
Triticum_monococcum_linn_TL     ............................................................    0
Triticum_urartu_PI428222        GAGAAGATAAAAGAGAATAGAAGCAGGCTATAGATTAATAACCAGTTGCAGCATGTGCCC  180
Triticum_urartu_PI428260        GAGAAGATAAAAGAGAATAGAAGCAGGCTATAGATTAATAACCAGTTGCAGCATGTGCCC  180
Triticum_urartu_PI428266        GAGAAGATAAAAGAGAATAGAAGCAGGCTATAGATTAATAACCAGTTGCAGCATGTGCCC  180
T.dicoccoides_As829             GAGAAGATAAAAGAGAATAGAAGCAGGCTATAGATTAATAACCAGTTGCAGCATGTGCCC  180
T.dicoccoides_As836             GAGAAGATAAAAGAGAATAGAAGCAGGCTATAGATTAATAACCAGTTGCAGCATGTGCCC  180
T.dicoccoides_As839             GAGAAGATAAAAGAGAATAGAAGCAGGCTATAGATTAATAACCAGTTGCAGCATGTGCCC  180
T.dicoccum_PI272527             GAGAAGATAAAAGAGAATAGAAGCAGGCTATAGATTAATAACCAGTTGCAGCATGTGCCC  180
T.dicoccum_PI193873             GAGAAGATAAAAGAGAATAGAAGCAGGCTATAGATTAATAACCAGTTGCAGCATGTGCCC  180
T.dicoccum_PI272527             GAGAAGATAAAAGAGAATAGAAGCAGGCTATAGATTAATAACCAGTTGCAGCATGTGCCC  180
T.durum_Dr8                     GAGAAGATAAAAGAGAATAGAAGCAGGCTATAGATTAATAACCAGTTGCAGCATGTGCCC  180
T.durum_Simeto-2                GAGAAGATAAAAGAGAATAGAAGCAGGCTATAGATTAATAACCAGTTGCAGCATGTGCCC  180
T.durum_Club57                  GAGAAGATAAAAGAGAATAGAAGCAGGCTATAGATTAATAACCAGTTGCAGCATGTGCCC  180
T.carthlicum_Tc                 GAGAAGATAAAAGAGAATAGAAGCAGGCTATAGATTAATAACCAGTTGCAGCATGTGCCC  180
Hexaploid wheat                 GAGAAGATAAAAGAGAATAGAAGCAGGCTATAGATTAATAACCAGTTGCAGCATGTGCCC  180

Triticum_boeoticum_boiss_Bo8    ............................................................    0
Triticum_monococcum_linn_Mo4    ............................................................    0
Triticum_monococcum_linn_TL     ............................................................    0
Triticum_urartu_PI428222        TAGTGGCTTTGTGAGAGAGTGAGGTGACTCATGTATCAACAAAGTAATACTCCCTCCTTC  240
Triticum_urartu_PI428260        TAGTGGCTTTGTGAGAGAGTGAGGTGACTCATGTATCAACAAAGTAATACTCCCTCCTTC  240
Triticum_urartu_PI428266        TAGTGGCTTTGTGAGAGAGTGAGGTGACTCATGTATCAACAAAGTAATACTCCCTCCTTC  240
T.dicoccoides_As829             TAGTGGCTTTGTGAGAGAGTGAGGTGACTCATGTATCAACAAAGTAATACTCCCTCCTTC  240
T.dicoccoides_As836             TAGTGGCTTTGTGAGAGAGTGAGGTGACTCATGTATCAACAAAGTAATACTCCCTCCTTC  240
T.dicoccoides_As839             TAGTGGCTTTGTGAGAGAGTGAGGTGACTCATGTATCAACAAAGTAATACTCCCTCCTTC  240
T.dicoccum_PI272527             TAGTGGCTTTGTGAGAGAGTGAGGTGACTCATGTATCAACAAAGTAATACTCCCTCCTTC  240
T.dicoccum_PI193873             TAGTGGCTTTGTGAGAGAGTGAGGTGACTCATGTATCAACAAAGTAATACTCCCTCCTTC  240
T.dicoccum_PI272527             TAGTGGCTTTGTGAGAGAGTGAGGTGACTCATGTATCAACAAAGTAATACTCCCTCCTTC  240
T.durum_Dr8                     TAGTGGCTTTGTGAGAGAGTGAGGTGACTCATGTATCAACAAAGTAATACTCCCTCCTTC  240
T.durum_Simeto-2                TAGTGGCTTTGTGAGAGAGTGAGGTGACTCATGTATCAACAAAGTAATACTCCCTCCTTC  240
T.durum_Club57                  TAGTGGCTTTGTGAGAGAGTGAGGTGACTCATGTATCAACAAAGTAATACTCCCTCCTTC  240
T.carthlicum_Tc                 TAGTGGCTTTGTGAGAGAGTGAGGTGACTCATGTATCAACAAAGTAATACTCCCTCCTTC  240
Hexaploid wheat                 TAGTGGCTTTGTGAGAGAGTGAGGTGACTCATGTATCAACAAAGTAATACTCCCTCCTTC  240
```

```
Triticum_boeoticum_boiss_Bo8      ............................................................    0
Triticum_monococcum_linn_Mo4      ............................................................    0
Triticum_monococcum_linn_TL       ............................................................    0
Triticum_urartu_PI428222          TAGGAATACTTGTCGGAGAAACGGATAAAAATGGATGTATGTAGAACTAAAATACGTCTA  300
Triticum_urartu_PI428260          TAGGAATACTTGTCGGAGAAACGGATAAAAATGGATGTATGTAGAACTAAAATACGTCTA  300
Triticum_urartu_PI428266          TAGGAATACTTGTCGGAGAAACGGATAAAAATGGATGTATGTAGAACTAAAATACGTCTA  300
T.dicoccoides_As829               TAGGAATACTTGTCGGAGAAACGGATAAAAATGGATGTATGTAGAACTAAAATACGTCTA  300
T.dicoccoides_As836               TAGGAATACTTGTCGGAGAAACGGATAAAAATGGATGTATGTAGAACTAAAATACGTCTA  300
T.dicoccoides_As839               TAGGAATACTTGTCGGAGAAACGGATAAAAATGGATGTATGTAGAACTAAAATACGTCTA  300
T.dicoccum_PI272527               TAGGAATACTTGTCGGAGAAACGGATAAAAATGGATGTATGTAGAACTAAAATACGTCTA  300
T.dicoccum_PI193873               TAGGAATACTTGTCGGAGAAACGGATAAAAATGGATGTATGTAGAACTAAAATACGTCTA  300
T.dicoccum_PI272527               TAGGAATACTTGTCGGAGAAACGGATAAAAATGGATGTATGTAGAACTAAAATACGTCTA  300
T.durum_Dr8                       TAGGAATACTTGTCGGAGAAACGGATAAAAATGGATGTATGTAGAACTAAAATACGTCTA  300
T.durum_Simeto-2                  TAGGAATACTTGTCGGAGAAACGGATAAAAATGGATGTATGTAGAACTAAAATACGTCTA  300
T.durum_Club57                    TAGGAATACTTGTCGGAGAAACGGATAAAAATGGATGTATGTAGAACTAAAATACGTCTA  300
T.carthlicum_Tc                   TAGGAATACTTGTCGGAGAAACGGATAAAAATGGATGTATGTAGAACTAAAATACGTCTA  300
Hexaploid wheat                   TAGGAATACTTGTCGGAGAAACGGATAAAAATGGATGTATGTAGAACTAAAATACGTCTA  300

Triticum_boeoticum_boiss_Bo8      ............................................................    0
Triticum_monococcum_linn_Mo4      ............................................................    0
Triticum_monococcum_linn_TL       ............................................................    0
Triticum_urartu_PI428222          AATACATTCATTTTCCGACAAATATTTTCAGATGAAGGGAGTACATATCTATATCTAATT  360
Triticum_urartu_PI428260          AATACATTCATTTTCCGACAAATATTTTCAGATGAAGGGAGTACATATCTATATCTAATT  360
Triticum_urartu_PI428266          AATACATTCATTTTCCGACAAATATTTTCAGATGAAGGGAGTACATATCTATATCTAATT  360
T.dicoccoides_As829               AATACATTCATTTTCCGACAAATATTTTCAGATGAAGGGAGTACATATCTATATCTAATT  360
T.dicoccoides_As836               AATACATTCATTTTCCGACAAATATTTTCAGATGAAGGGAGTACATATCTATATCTAATT  360
T.dicoccoides_As839               AATACATTCATTTTCCGACAAATATTTTCAGATGAAGGGAGTACATATCTATATCTAATT  360
T.dicoccum_PI272527               AATACATTCATTTTCCGACAAATATTTTCAGATGAAGGGAGTACATATCTATATCTAATT  360
T.dicoccum_PI193873               AATACATTCATTTTCCGACAAATATTTTCAGATGAAGGGAGTACATATCTATATCTAATT  360
T.dicoccum_PI272527               AATACATTCATTTTCCGACAAATATTTTCAGATGAAGGGAGTACATATCTATATCTAATT  360
T.durum_Dr8                       AATACATTCATTTTCCGACAAATATTTTCAGATGAAGGGAGTACATATCTATATCTAATT  360
T.durum_Simeto-2                  AATACATTCATTTTCCGACAAATATTTTCAGATGAAGGGAGTACATATCTATATCTAATT  360
T.durum_Club57                    AATACATTCATTTTCCGACAAATATTTTCAGATGAAGGGAGTACATATCTATATCTAATT  360
T.carthlicum_Tc                   AATACATTCATTTTCCGACAAATATTTTCAGATGAAGGGAGTACATATCTATATCTAATT  360
Hexaploid_wheat                   AATACATTCATTTTCCGACAAATATTTTCAGATGAAGGGAGTACATATCTATATCTAATT  360

Triticum_boeoticum_boiss_Bo8      ............................................................    0
Triticum_monococcum_linn_Mo4      ............................................................    0
Triticum_monococcum_linn_TL       ............................................................    0
Triticum_urartu_PI428222          ATTATATTTGCTGACTATAAACTTAAATATAAATGACATGGCAACATTATATAGCTATTA  420
Triticum_urartu_PI428260          ATTATATTTGCTGACTATAAACTTAAATATAAATGACATGGCAACATTATATAGCTATTA  420
Triticum_urartu_PI428266          ATTATATTTGCTGACTATAAACTTAAATATAAATGACATGGCAACATTATATAGCTATTA  420
T.dicoccoides_As829               ATTATATTTGCTGACTATAAACTTAAATATAAATGACATGGCAACATTATATAGCTATTA  420
T.dicoccoides_As836               ATTATATTTGCTGACTATAAACTTAAATATAAATGACATGGCAACATTATATAGCTATTA  420
T.dicoccoides_As839               ATTATATTTGCTGACTATAAACTTAAATATAAATGACATGGCAACATTATATAGCTATTA  420
T.dicoccum_PI272527               ATTATATTTGCTGACTATAAACTTAAATATAAATGACATGGCAACATTATATAGCTATTA  420
T.dicoccum_PI193873               ATTATATTTGCTGACTATAAACTTAAATATAAATGACATGGCAACATTATATAGCTATTA  420
T.dicoccum_PI272527               ATTATATTTGCTGACTATAAACTTAAATATAAATGACATGGCAACATTATATAGCTATTA  420
T.durum_Dr8                       ATTATATTTGCTGACTATAAACTTAAATATAAATGACATGGCAACATTATATAGCTATTA  420
T.durum_Simeto-2                  ATTATATTTGCTGACTATAAACTTAAATATAAATGACATGGCAACATTATATAGCTATTA  420
T.durum_Club57                    ATTATATTTGCTGACTATAAACTTAAATATAAATGACATGGCAACATTATATAGCTATTA  420
T.carthlicum_Tc                   ATTATATTTGCTGACTATAAACTTAAATATAAATGACATGGCAACATTATATAGCTATTA  420
Hexaploid wheat                   ATTATATTTGCTGACTATAAACTTAAATATAAATGACATGGCAACATTATATAGCTATTA  420

Triticum_boeoticum_boiss_Bo8      ............................................................    0
Triticum_monococcum_linn_Mo4      ............................................................    0
Triticum_monococcum_linn_TL       ............................................................    0
Triticum_urartu_PI428222          ACCATGCTCCCGACCATGTATCGGTGATGTCATTATTGTTCATTCAATCACTTTCCAATT  480
Triticum_urartu_PI428260          ACCATGCTCCCGACCATGTATCGGTGATGTCATTATTGTTCATTCAATCACTTTCCAATT  480
Triticum_urartu_PI428266          ACCATGCTCCCGACCATGTATCGGTGATGTCATTATTGTTCATTCAATCACTTTCCAATT  480
T.dicoccoides_As829               ACCATGCTCCCGACCATGTATCGGTGATGTCATTATTGTTCATTCAATCACTTTCCAATT  480
T.dicoccoides_As836               ACCATGCTCCCGACCATGTATCGGTGATGTCATTATTGTTCATTCAATCACTTTCCAATT  480
T.dicoccoides_As839               ACCATGCTCCCGACCATGTATCGGTGATGTCATTATTGTTCATTCAATCACTTTCCAATT  480
T.dicoccum_PI272527               ACCATGCTCCCGACCATGTATCGGTGATGTCATTATTGTTCATTCAATCACTTTCCAATT  480
T.dicoccum_PI193873               ACCATGCTCCCGACCATGTATCGGTGATGTCATTATTGTTCATTCAATCACTTTCCAATT  480
T.dicoccum_PI272527               ACCATGCTCCCGACCATGTATCGGTGATGTCATTATTGTTCATTCAATCACTTTCCAATT  480
T.durum_Dr8                       ACCATGCTCCCGACCATGTATCGGTGATGTCATTATTGTTCATTCAATCACTTTCCAATT  480
T.durum_Simeto-2                  ACCATGCTCCCGACCATGTATCGGTGATGTCATTATTGTTCATTCAATCACTTTCCAATT  480
T.durum_Club57                    ACCATGCTCCCGACCATGTATCGGTGATGTCATTATTGTTCATTCAATCACTTTCCAATT  480
T.carthlicum_Tc                   ACCATGCTCCCGACCATGTATCGGTGATGTCATTATTGTTCATTCAATCACTTTCCAATT  480
Hexaploid_wheat                   ACCATGCTCCCGACCATGTATCGGTGATGTCATTATTGTTCATTCAATCACTTTCCAATT  480
```

```
Triticum_boeoticum_boiss_Bo8      ............................................................     0
Triticum_monococcum_linn_Mo4      ............................................................     0
Triticum_monococcum_linn_TL       ............................................................     0
Triticum_urartu_PI428222          AATACTTTCTACAGATGGGATATAAATATGGCTAAACTTACCCTTGATTAATCGACGGAC   540
Triticum_urartu_PI428260          AATACTTTCTACAGATGGGATATAAATATGGCTAAACTTACCCTTGATTAATCGACGGAC   540
Triticum_urartu_PI428266          AATACTTTCTACAGATGGGATATAAATATGGCTAAACTTACCCTTGATTAATCGACGGAC   540
T.dicoccoides_As829               AATACTTTCTACAGATGGGATATAAATATGGCTAAACTTACCCTTGATTAATCGACGGAC   540
T.dicoccoides_As836               AATACTTTCTACAGATGGGATATAAATATGGCTAAACTTACCCTTGATTAATCGACGGAC   540
T.dicoccoides_As839               AATACTTTCTACAGATGGGATATAAATATGGCTAAACTTACCCTTGATTAATCGACGGAC   540
T.dicoccum_PI272527               AATACTTTCTACAGATGGGATATAAATATGGCTAAACTTACCCTTGATTAATCGACGGAC   540
T.dicoccum_PI193873               AATACTTTCTACAGATGGGATATAAATATGGCTAAACTTACCCTTGATTAATCGACGGAC   540
T.dicoccum_PI272527               AATACTTTCTACAGATGGGATATAAATATGGCTAAACTTACCCTTGATTAATCGACGGAC   540
T.durum_Dr8                       AATACTTTCTACAGATGGGATATAAATATGGCTAAACTTACCCTTGATTAATCGACGGAC   540
T.durum_Simeto-2                  AATACTTTCTACAGATGGGATATAAATATGGCTAAACTTACCCTTGATTAATCGACGGAC   540
T.durum_Club57                    AATACTTTCTACAGATGGGATATAAATATGGCTAAACTTACCCTTGATTAATCGACGGAC   540
T.carthlicum_Tc                   AATACTTTCTACAGATGGGATATAAATATGGCTAAACTTACCCTTGATTAATCGACGGAC   540
Hexaploid_wheat                   AATACTTTCTACAGATGGGATATAAATATGGCTAAACTTACCCTTGATTAATCGACGGAC   540

Triticum_boeoticum_boiss_Bo8      ............................................................     0
Triticum_monococcum_linn_Mo4      ............................................................     0
Triticum_monococcum_linn_TL       ............................................................     0
Triticum_urartu_PI428222          GGTATCAAATATCTTCTGCTATCATAAGTTGAAGCATCTTAAAAGAGCTCCTGACAAAAC   600
Triticum_urartu_PI428260          GGTATCAAATATCTTCTGCTATCATAAGTTGAAGCATCTTAAAAGAGCTCCTGACAAAAC   600
Triticum_urartu_PI428266          GGTATCAAATATCTTCTGCTATCATAAGTTGAAGCATCTTAAAAGAGCTCCTGACAAAAC   600
T.dicoccoides_As829               GGTATCAAATATCTTCTGCTATCATAAGTTGAAGCATCTTAAAAGAGCTCCTGACAAAAC   600
T.dicoccoides_As836               GGTATCAAATATCTTCTGCTATCATAAGTTGAAGCATCTTAAAAGAGCTCCTGACAAAAC   600
T.dicoccoides_As839               GGTATCAAATATCTTCTGCTATCATAAGTTGAAGCATCTTAAAAGAGCTCCTGACAAAAC   600
T.dicoccum_PI272527               GGTATCAAATATCTTCTGCTATCATAAGTTGAAGCATCTTAAAAGAGCTCCTGACAAAAC   600
T.dicoccum_PI193873               GGTATCAAATATCTTCTGCTATCATAAGTTGAAGCATCTTAAAAGAGCTCCTGACAAAAC   600
T.dicoccum_PI272527               GGTATCAAATATCTTCTGCTATCATAAGTTGAAGCATCTTAAAAGAGCTCCTGACAAAAC   600
T.durum_Dr8                       GGTATCAAATATCTTCTGCTATCATAAGTTGAAGCATCTTAAAAGAGCTCCTGACAAAAC   600
T.durum_Simeto-2                  GGTATCAAATATCTTCTGCTATCATAAGTTGAAGCATCTTAAAAGAGCTCCTGACAAAAC   600
T.durum_Club57                    GGTATCAAATATCTTCTGCTATCATAAGTTGAAGCATCTTAAAAGAGCTCCTGACAAAAC   600
T.carthlicum_Tc                   GGTATCAAATATCTTCTGCTATCATAAGTTGAAGCATCTTAAAAGAGCTCCTGACAAAAC   600
Hexaploid wheat                   GGTATCAAATATCTTCTGCTATCATAAGTTGAAGCATCTTAAAAGAGCTCCTGACAAAAC   600

Triticum_boeoticum_boiss_Bo8      ............................................................     0
Triticum_monococcum_linn_Mo4      ............................................................     0
Triticum_monococcum_linn_TL       ............................................................     0
Triticum_urartu_PI428222          CTTGGTTGTACGTAACCTTCAAACGTGGACAGTAGGATTTGTTTCTTCGATCCGCTCCAA   660
Triticum_urartu_PI428260          CTTGGTTGTACGTAACCTTCAAACGTGGACAGTAGGATTTGTTTCTTCGATCCGCTCCAA   660
Triticum_urartu_PI428266          CTTGGTTGTACGTAACCTTCAAACGTGGACAGTAGGATTTGTTTCTTCGATCCGCTCCAA   660
T.dicoccoides_As829               CTTGGTTGTACGTAACCTTCAAACGTGGACAGTAGGATTTGTTTCTTCGATCCGCTCCAA   660
T.dicoccoides_As836               CTTGGTTGTACGTAACCTTCAAACGTGGACAGTAGGATTTGTTTCTTCGATCCGCTCCAA   660
T.dicoccoides_As839               CTTGGTTGTACGTAACCTTCAAACGTGGACAGTAGGATTTGTTTCTTCGATCCGCTCCAA   660
T.dicoccum_PI272527               CTTGGTTGTACGTAACCTTCAAACGTGGACAGTAGGATTTGTTTCTTCGATCCGCTCCAA   660
T.dicoccum_PI193873               CTTGGTTGTACGTAACCTTCAAACGTGGACAGTAGGATTTGTTTCTTCGATCCGCTCCAA   660
T.dicoccum_PI272527               CTTGGTTGTACGTAACCTTCAAACGTGGACAGTAGGATTTGTTTCTTCGATCCGCTCCAA   660
T.durum_Dr8                       CTTGGTTGTACGTAACCTTCAAACGTGGACAGTAGGATTTGTTTCTTCGATCCGCTCCAA   660
T.durum_Simeto-2                  CTTGGTTGTACGTAACCTTCAAACGTGGACAGTAGGATTTGTTTCTTCGATCCGCTCCAA   660
T.durum_Club57                    CTTGGTTGTACGTAACCTTCAAACGTGGACAGTAGGATTTGTTTCTTCGATCCGCTCCAA   660
T.carthlicum_Tc                   CTTGGTTGTACGTAACCTTCAAACGTGGACAGTAGGATTTGTTTCTTCGATCCGCTCCAA   660
Hexaploid wheat                   CTTGGTTGTACGTAACCTTCAAACGTGGACAGTAGGATTTGTTTCTTCGATCCGCTCCAA   660

Triticum_boeoticum_boiss_Bo8      ............................................................     0
Triticum_monococcum_linn_Mo4      ............................................................     0
Triticum_monococcum_linn_TL       ............................................................     0
Triticum_urartu_PI428222          ACAGGTCATGTAGGTATACGTGCCTTCGTACGAGACGCTGGTGGGGTAAGCAGCTACCCA   720
Triticum_urartu_PI428260          ACAGGTCATGTAGGTATACGTGCCTTCGTACGAGACGCTGGTGGGGTAAGCAGCTACCCA   720
Triticum_urartu_PI428266          ACAGGTCATGTAGGTATACGTGCCTTCGTACGAGACGCTGGTGGGGTAAGCAGCTACCCA   720
T.dicoccoides_As829               ACAGGTCATGTAGGTATACGTGCCTTCGTACGAGACGCTGGTGGGGTAAGCAGCTACCCA   720
T.dicoccoides_As836               ACAGGTCATGTAGGTATACGTGCCTTCGTACGAGACGCTGGTGGGGTAAGCAGCTACCCA   720
T.dicoccoides_As839               ACAGGTCATGTAGGTATACGTGCCTTCGTACGAGACGCTGGTGGGGTAAGCAGCTACCCA   720
T.dicoccum_PI272527               ACAGGTCATGTAGGTATACGTGCCTTCGTACGAGACGCTGGTGGGGTAAGCAGCTACCCA   720
T.dicoccum_PI193873               ACAGGTCATGTAGGTATACGTGCCTTCGTACGAGACGCTGGTGGGGTAAGCAGCTACCCA   720
T.dicoccum_PI272527               ACAGGTCATGTAGGTATACGTGCCTTCGTACGAGACGCTGGTGGGGTAAGCAGCTACCCA   720
T.durum_Dr8                       ACAGGTCATGTAGGTATACGTGCCTTCGTACGAGACGCTGGTGGGGTAAGCAGCTACCCA   720
T.durum_Simeto-2                  ACAGGTCATGTAGGTATACGTGCCTTCGTACGAGACGCTGGTGGGGTAAGCAGCTACCCA   720
T.durum_Club57                    ACAGGTCATGTAGGTATACGTGCCTTCGTACGAGACGCTGGTGGGGTAAGCAGCTACCCA   720
T.carthlicum_Tc                   ACAGGTCATGTAGGTATACGTGCCTTCGTACGAGACGCTGGTGGGGTAAGCAGCTACCCA   720
Hexaploid wheat                   ACAGGTCATGTAGGTATACGTGCCTTCGTACGAGACGCTGGTGGGGTAAGCAGCTACCCA   720
```

Triticum_boeoticum_boiss_Bo8	....	0
Triticum_monococcum_linn_Mo4	....	0
Triticum_monococcum_linn_TL	....	0
Triticum_urartu_PI428222	TTTCTTTTCTTTTATTGGTATAGGAAATTCGACCGTACATTACCACATAAACCTTGTCGA	780
Triticum_urartu_PI428260	TTTCTTTTCTTTTATTGGTATAGGAAATTCGACCGTACATTACCACATAAACCTTGTCGA	780
Triticum_urartu_PI428266	TTTCTTTTCTTTTATTGGTATAGGAAATTCGACCGTACATTACCACATAAACCTTGTCGA	780
T.dicoccoides_As829	TTTCTTTTCTTTTATTGGTATAGGAAATTCGACCGTACATTACCACATAAACCTTGTCGA	780
T.dicoccoides_As836	TTTCTTTTCTTTTATTGGTATAGGAAATTCGACCGTACATTACCACATAAACCTTGTCGA	780
T.dicoccoides_As839	TTTCTTTTCTTTTATTGGTATAGGAAATTCGACCGTACATTACCACATAAACCTTGTCGA	780
T.dicoccum_PI272527	TTTCTTTTCTTTTATTGGTATAGGAAATTCGACCGTACATTACCACATAAACCTTGTCGA	780
T.dicoccum_PI193873	TTTCTTTTCTTTTATTGGTATAGGAAATTCGACCGTACATTACCACATAAACCTTGTCGA	780
T.dicoccum_PI272527	TTTCTTTTCTTTTATTGGTATAGGAAATTCGACCGTACATTACCACATAAACCTTGTCGA	780
T.durum_Dr8	TTTCTTTTCTTTTATTGGTATAGGAAATTCGACCGTACATTACCACATAAACCTTGTCGA	780
T.durum_Simeto-2	TTTCTTTTCTTTTATTGGTATAGGAAATTCGACCGTACATTACCACATAAACCTTGTCGA	780
T.durum_Club57	TTTCTTTTCTTTTATTGGTATAGGAAATTCGACCGTACATTACCACATAAACCTTGTCGA	780
T.carthlicum_Tc	TTTCTTTTCTTTTATTGGTATAGGAAATTCGACCGTACATTACCACATAAACCTTGTCGA	780
Hexaploid_wheat	TTTCTTTTCTTTTATTGGTATAGGAAATTCGACCGTACATTACCACATAAACCTTGTCGA	780

Triticum_boeoticum_boiss_Bo8	....	0
Triticum_monococcum_linn_Mo4	....	0
Triticum_monococcum_linn_TL	....	0
Triticum_urartu_PI428222	CAACTCTCCGCGCACTGCTCCACCACAGAACCTTGTCGACCGTCGATTGTCGCCATGCGC	840
Triticum_urartu_PI428260	CAACTCTCCGCGCACTGCTCCACCACAGAACCTTGTCGACCGTCGATTGTCGCCATGCGC	840
Triticum_urartu_PI428266	CAACTCTCCGCGCACTGCTCCACCACAGAACCTTGTCGACCGTCGATTGTCGCCATGCGC	840
T.dicoccoides_As829	CAACTCTCCGCGCACTGCTCCACCACAGAACCTTGTCGACCGTCGATTGTCGCCATGCGC	840
T.dicoccoides_As836	CAACTCTCCGCGCACTGCTCCACCACAGAACCTTGTCGACCGTCGATTGTCGCCATGCGC	840
T.dicoccoides_As839	CAACTCTCCGCGCACTGCTCCACCACAGAACCTTGTCGACCGTCGATTGTCGCCATGCGC	840
T.dicoccum_PI272527	CAACTCTCCGCGCACTGCTCCACCACAGAACCTTGTCGACCGTCGATTGTCGCCATGCGC	840
T.dicoccum_PI193873	CAACTCTCCGCGCACTGCTCCACCACAGAACCTTGTCGACCGTCGATTGTCGCCATGCGC	840
T.dicoccum_PI272527	CAACTCTCCGCGCACTGCTCCACCACAGAACCTTGTCGACCGTCGATTGTCGCCATGCGC	840
T.durum_Dr8	CAACTCTCCGCGCACTGCTCCACCACAGAACCTTGTCGACCGTCGATTGTCGCCATGCGC	840
T.durum_Simeto-2	CAACTCTCCGCGCACTGCTCCACCACAGAACCTTGTCGACCGTCGATTGTCGCCATGCGC	840
T.durum_Club57	CAACTCTCCGCGCACTGCTCCACCACAGAACCTTGTCGACCGTCGATTGTCGCCATGCGC	840
T.carthlicum_Tc	CAACTCTCCGCGCACTGCTCCACCACAGAACCTTGTCGACCGTCGATTGTCGCCATGCGC	840
Hexaploid_wheat	CAACTCTCCGCGCACTGCTCCACCACAGAACCTTGTCGACCGTCGATTGTCGCCATGCGC	840

Triticum_boeoticum_boiss_Bo8	....	0
Triticum_monococcum_linn_Mo4	....	0
Triticum_monococcum_linn_TL	....	0
Triticum_urartu_PI428222	CGGAGCGATCGGCCCCCGACGTGTCACCCCCCAGCTCACCCTTCGCCCCTCCGCGTCCGG	900
Triticum_urartu_PI428260	CGGAGCGATCGGCCCCCGACGTGTCACCCCCCAGCTCACCCTTCGCCCCTCCGCGTCCGG	900
Triticum_urartu_PI428266	CGGAGCGATCGGCCCCCGACGTGTCACCCCCCAGCTCACCCTTCGCCCCTCCGCGTCCGG	900
T.dicoccoides_As829	CGGAGCGATCGGCCCCCGACGTGTCACCCCCCAGCTCACCCTTCGCCCCTCCGCGTCCGG	900
T.dicoccoides_As836	CGGAGCGATCGGCCCCCGACGTGTCACCCCCCAGCTCACCCTTCGCCCCTCCGCGTCCGG	900
T.dicoccoides_As839	CGGAGCGATCGGCCCCCGACGTGTCACCCCCCAGCTCACCCTTCGCCCCTCCGCGTCCGG	900
T.dicoccum_PI272527	CGGAGCGATCGGCCCCCGACGTGTCACCCCCCAGCTCACCCTTCGCCCCTCCGCGTCCGG	900
T.dicoccum_PI193873	CGGAGCGATCGGCCCCCGACGTGTCACCCCCCAGCTCACCCTTCGCCCCTCCGCGTCCGG	900
T.dicoccum_PI272527	CGGAGCGATCGGCCCCCGACGTGTCACCCCCCAGCTCACCCTTCGCCCCTCCGCGTCCGG	900
T.durum_Dr8	CGGAGCGATCGGCCCCCGACGTGTCACCCCCCAGCTCACCCTTCGCCCCTCCGCGTCCGG	900
T.durum_Simeto-2	CGGAGCGATCGGCCCCCGACGTGTCACCCCCCAGCTCACCCTTCGCCCCTCCGCGTCCGG	900
T.durum_Club57	CGGAGCGATCGGCCCCCGACGTGTCACCCCCCAGCTCACCCTTCGCCCCTCCGCGTCCGG	900
T.carthlicum_Tc	CGGAGCGATCGGCCCCCGACGTGTCACCCCCCAGCTCACCCTTCGCCCCTCCGCGTCCGG	900
Hexaploid wheat	CGGAGCGATCGGCCCCCGACGTGTCACCCCCCAGCTCACCCTTCGCCCCTCCGCGTCCGG	900

Triticum_boeoticum_boiss_Bo8	....	0
Triticum_monococcum_linn_Mo4	....	0
Triticum_monococcum_linn_TL	....	0
Triticum_urartu_PI428222	CCTATAAAAACGACGATGCACCTCTCGTCCTCCCATTGCATTGCAAGTGAAGCTCACACA	960
Triticum_urartu_PI428260	CCTATAAAAACGACGATGCACCTCTCGTCCTCCCATTGCATTGCAAGTGAAGCTCACACA	960
Triticum_urartu_PI428266	CCTATAAAAACGACGATGCACCTCTCGTCCTCCCATTGCATTGCAAGTGAAGCTCACACA	960
T.dicoccoides_As829	CCTATAAAAACGACGATGCACCTCTCGTCCTCCCATTGCATTGCAAGTGAAGCTCACACA	960
T.dicoccoides_As836	CCTATAAAAACGACGATGCACCTCTCGTCCTCCCATTGCATTGCAAGTGAAGCTCACACA	960
T.dicoccoides_As839	CCTATAAAAACGACGATGCACCTCTCGTCCTCCCATTGCATTGCAAGTGAAGCTCACACA	960
T.dicoccum_PI272527	CCTATAAAAACGACGATGCACCTCTCGTCCTCCCATTGCATTGCAAGTGAAGCTCACACA	960
T.dicoccum_PI193873	CCTATAAAAACGACGATGCACCTCTCGTCCTCCCATTGCATTGCAAGTGAAGCTCACACA	960
T.dicoccum_PI272527	CCTATAAAAACGACGATGCACCTCTCGTCCTCCCATTGCATTGCAAGTGAAGCTCACACA	960
T.durum_Dr8	CCTATAAAAACGACGATGCACCTCTCGTCCTCCCATTGCATTGCAAGTGAAGCTCACACA	960
T.durum_Simeto-2	CCTATAAAAACGACGATGCACCTCTCGTCCTCCCATTGCATTGCAAGTGAAGCTCACACA	960
T.durum_Club57	CCTATAAAAACGACGATGCACCTCTCGTCCTCCCATTGCATTGCAAGTGAAGCTCACACA	960
T.carthlicum_Tc	CCTATAAAAACGACGATGCACCTCTCGTCCTCCCATTGCATTGCAAGTGAAGCTCACACA	960
Hexaploid wheat	CCTATAAAAACGACGATGCACCTCTCGTCCTCCCATTGCATTGCAAGTGAAGCTCACACA	960

```
Triticum_boeoticum_boiss_Bo8       .................................................ATGGCTTCTTC      11
Triticum_monococcum_linn_Mo4       .................................................ATGGCTTCTTC      11
Triticum_monococcum_linn_TL        .................................................ATGGCTTCTTC      11
Triticum_urartu_PI428222           ACCTACCCTACCCTACCCACCCATCCATCAGCAGTTTTTCTATCGACCAATGGCTTCTTC    1020
Triticum_urartu_PI428260           ACCTACCCTACCCTACCCACCCATCCATCAGCAGTTTTTCTATCGACCAATGGCTTCTTC    1020
Triticum_urartu_PI428266           ACCTACCCTACCCTACCCACCCATCCATCAGCAGTTTTTCTATCGACCAATGGCTTCTTC    1020
T.dicoccoides_As829                ACCTACCCTACCCTACCCACCCATCCATCAGCAGTTTTTCTATCGACCAATGGCTTCTTC    1020
T.dicoccoides_As836                ACCTACCCTACCCTACCCACCCATCCATCAGCAGTTTTTCTATCGACCAATGGCTTCTTC    1020
T.dicoccoides_As839                ACCTACCCTACCCTACCCACCCATCCATCAGCAGTTTTTCTATCGACCAATGGCTTCTTC    1020
T.dicoccum_PI272527                ACCTACCCTACCCTACCCACCCATCCATCAGCAGTTTTTCTATCGACCAATGGCTTCTTC    1020
T.dicoccum_PI193873                ACCTACCCTACCCTACCCACCCATCCATCAGCAGTTTTTCTATCGACCAATGGCTTCTTC    1020
T.dicoccum_PI272527                ACCTACCCTACCCTACCCACCCATCCATCAGCAGTTTTTCTATCGACCAATGGCTTCTTC    1020
T.durum_Dr8                        ACCTACCCTACCCTACCCACCCATCCATCAGCAGTTTTTCTATCGACCAATGGCTTCTTC    1020
T.durum_Simeto-2                   ACCTACCCTACCCTACCCACCCATCCATCAGCAGTTTTTCTATCGACCAATGGCTTCTTC    1020
T.durum_Club57                     ACCTACCCTACCCTACCCACCCATCCATCAGCAGTTTTTCTATCGACCAATGGCTTCTTC    1020
T.carthlicum_Tc                    ACCTACCCTACCCTACCCACCCATCCATCAGCAGTTTTTCTATCGACCAATGGCTTCTTC    1020
Hexaploid_wheat                    ACCTACCCTACCCTACCCACCCATCCATCAGCAGTTTTTCTATCGACCAATGGCTTCTTC    1020

Triticum_boeoticum_boiss_Bo8       TTCCGTGCTTCTCGGAGCCTCGGCCACGGCCGCGCTCACCGGCACCCCGGCAGGCAAGGC      71
Triticum_monococcum_linn_Mo4       TTCCGTGCTTCTCGGAGCCTCGGCCACGGCCGCGCTCACCGGCACCCCGGCAGGCAAGGC      71
Triticum_monococcum_linn_TL        TTCCGTGCTTCTCGGAGCCTCGGCCACGGCCGCGCTCACCGGCACCCCGGCAGGCAAGGC      71
Triticum_urartu_PI428222           TTCCGTGCTTCTCGGAGCCTCGGCCACGGCCGCGCTCACCGGCACCCCGGCAGGCAAGGC    1080
Triticum_urartu_PI428260           TTCCGTGCTTCTCGGAGCCTCGGCCACGGCCGCGCTCACCGGCACCCCGGCAGGCAAGGC    1080
Triticum_urartu_PI428266           TTCCGTGCTTCTCGGAGCCTCGGCCACGGCCGCGCTCACCGGCACCCCGGCAGGCAAGGC    1080
T.dicoccoides_As829                TTCCGTGCTTCTCGGAGCCTCGGCCACGGCCGCGCTCACCGGCACCCCGGCAGGCAAGGC    1080
T.dicoccoides_As836                TTCCGTGCTTCTCGGAGCCTCGGCCACGGCCGCGCTCACCGGCACCCCGGCAGGCAAGGC    1080
T.dicoccoides_As839                TTCCGTGCTTCTCGGAGCCTCGGCCACGGCCGCGCTCACCGGCACCCCGGCAGGCAAGGC    1080
T.dicoccum_PI272527                TTCCGTGCTTCTCGGAGCCTCGGCCACGGCCGCGCTCACCGGCACCCCGGCAGGCAAGGC    1080
T.dicoccum_PI193873                TTCCGTGCTTCTCGGAGCCTCGGCCACGGCCGCGCTCACCGGCACCCCGGCAGGCAAGGC    1080
T.dicoccum_PI272527                TTCCGTGCTTCTCGGAGCCTCGGCCACGGCCGCGCTCACCGGCACCCCGGCAGGCAAGGC    1080
T.durum_Dr8                        TTCCGTGCTTCTCGGAGCCTCGGCCACGGCCGCGCTCACCGGCACCCCGGCAGGCAAGGC    1080
T.durum_Simeto-2                   TTCCGTGCTTCTCGGAGCCTCGGCCACGGCCGCGCTCACCGGCACCCCGGCAGGCAAGGC    1080
T.durum_Club57                     TTCCGTGCTTCTCGGAGCCTCGGCCACGGCCGCGCTCACCGGCACCCCGGCAGGCAAGGC    1080
T.carthlicum_Tc                    TTCCGTGCTTCTCGGAGCCTCGGCCACGGCCGCGCTCACCGGCACCCCGGCAGGCAAGGC    1080
Hexaploid_wheat                    TTCCGTGCTTCTCGGAGCCTCGGCCACGGCCGCGCTCACCGGCACCCCGGCAGGCAAGGC    1080

Triticum_boeoticum_boiss_Bo8       CCTTCCCCGGCCTTGCTTCCTCGCCGCTCGCCCGCGCACCGTGAGCGGCGGCCGTCTCTG     131
Triticum_monococcum_linn_Mo4       CCTTCCCCGGCCTTGCTTCCTCGCCGCTCGCCCGCGCACCGTGAGCGGCGGCCGTCTCTG     131
Triticum_monococcum_linn_TL        CCTTCCCCGGCCTTGCTTCCTCGCCGCTCGCCCGCGCACCGTGAGCGGCGGCCGTCTCTG     131
Triticum_urartu_PI428222           CCTTCCCCGGCCTTGCTTCCTCGCCGCTCGCCCGCGCACCGTGAGCGGCGGCCGCCTCTG    1140
Triticum_urartu_PI428260           CCTTCCCCGGCCTTGCTTCCTCGCCGCTCGCCCGCGCACCGTGAGCGGCGGCCGCCTCTG    1140
Triticum_urartu_PI428266           CCTTCCCCGGCCTTGCTTCCTCGCCGCTCGCCCGCGCACCGTGAGCGGCGGCCGCCTCTG    1140
T.dicoccoides_As829                CCTTCCCCGGCCTTGCTTCCTCGCCGCTCGCCCGCGCACCGTGAGCGGCGGCCGCCTCTG    1140
T.dicoccoides_As836                CCTTCCCCGGCCTTGCTTCCTCGCCGCTCGCCCGCGCACCGTGAGCGGCGGCCGCCTCTG    1140
T.dicoccoides_As839                CCTTCCCCGGCCTTGCTTCCTCGCCGCTCGCCCGCGCACCGTGAGCGGCGGCCGCCTCTG    1140
T.dicoccum_PI272527                CCTTCCCCGGCCTTGCTTCCTCGCCGCTCGCCCGCGCACCGTGAGCGGCGGCCGCCTCTG    1140
T.dicoccum_PI193873                CCTTCCCCGGCCTTGCTTCCTCGCCGCTCGCCCGCGCACCGTGAGCGGCGGCCGCCTCTG    1140
T.dicoccum_PI272527                CCTTCCCCGGCCTTGCTTCCTCGCCGCTCGCCCGCGCACCGTGAGCGGCGGCCGCCTCTG    1140
T.durum_Dr8                        CCTTCCCCGGCCTTGCTTCCTCGCCGCTCGCCCGCGCACCGTGAGCGGCGGCCGCCTCTG    1140
T.durum_Simeto-2                   CCTTCCCCGGCCTTGCTTCCTCGCCGCTCGCCCGCGCACCGTGAGCGGCGGCCGCCTCTG    1140
T.durum_Club57                     CCTTCCCCGGCCTTGCTTCCTCGCCGCTCGCCCGCGCACCGTGAGCGGCGGCCGCCTCTG    1140
T.carthlicum_Tc                    CCTTCCCCGGCCTTGCTTCCTCGCCGCTCGCCCGCGCACCGTGAGCGGCGGCCGCCTCTG    1140
Hexaploid_wheat                    CCTTCCCCGGCCTTGCTTCCTCGCCGCTCGCCCGCGCACCGTGAGCGGCGGCCGCCTCTG    1140

Triticum_boeoticum_boiss_Bo8       CCTGCAGAACGCTCCAAGGGCGACTCCGGTACGTGTGCCGCGAGCAGTACCGTTCGATCA     191
Triticum_monococcum_linn_Mo4       CCTGCAGAACGCTCCAAGGGCGACTCCGGTACGTGTGCCGCGAGCAGTACCGTTCGATCA     191
Triticum_monococcum_linn_TL        CCTGCAGAACGCTCCAAGGGCGACTCCGGTACGTGTGCCGCGAGCAGTACCGTTCGATCA     191
Triticum_urartu_PI428222           CCTGCAGAACGCTCCAAGGGCGACTCCGGTACGTGTGCCGCGAGCAGTACCGTTCGATCA    1200
Triticum_urartu_PI428260           CCTGCAGAACGCTCCAAGGGCGACTCCGGTACGTGTGCCGCGAGCAGTACCGTTCGATCA    1200
Triticum_urartu_PI428266           CCTGCAGAACGCTCCAAGGGCGACTCCGGTACGTGTGCCGCGAGCAGTACCGTTCGATCA    1200
T.dicoccoides_As829                CCTGCAGAACGCTCCAAGGGCGACTCCGGTACGTGTGCCGCGAGCAGTACCGTTCGATCA    1200
T.dicoccoides_As836                CCTGCAGAACGCTCCAAGGGCGACTCCGGTACGTGTGCCGCGAGCAGTACCGTTCGATCA    1200
T.dicoccoides_As839                CCTGCAGAACGCTCCAAGGGCGACTCCGGTACGTGTGCCGCGAGCAGTACCGTTCGATCA    1200
T.dicoccum_PI272527                CCTGCAGAACGCTCCAAGGGCGACTCCGGTACGTGTGCCGCGAGCAGTACCGTTCGATCA    1200
T.dicoccum_PI193873                CCTGCAGAACGCTCCAAGGGCGACTCCGGTACGTGTGCCGCGAGCAGTACCGTTCGATCA    1200
T.dicoccum_PI272527                CCTGCAGAACGCTCCAAGGGCGACTCCGGTACGTGTGCCGCGAGCAGTACCGTTCGATCA    1200
T.durum_Dr8                        CCTGCAGAACGCTCCAAGGGCGACTCCGGTACGTGTGCCGCGAGCAGTACCGTTCGATCA    1200
T.durum_Simeto-2                   CCTGCAGAACGCTCCAAGGGCGACTCCGGTACGTGTGCCGCGAGCAGTACCGTTCGATCA    1200
T.durum_Club57                     CCTGCAGAACGCTCCAAGGGCGACTCCGGTACGTGTGCCGCGAGCAGTACCGTTCGATCA    1200
T.carthlicum_Tc                    CCTGCAGAACGCTCCAAGGGCGACTCCGGTACGTGTGCCGCGAGCAGTACCGTTCGATCA    1200
Hexaploid wheat                    CCTGCAGAACGCTCCAAGGGCGACTCCGGTACGTGTGCCGCGAGCAGTACCGTTCGATCA    1200
```

```
Triticum_boeoticum_boiss_Bo8     TGGCCTCATGCCGATTATTTTTACGTACGCTGCTGGTTAGTCGTCCTCTGAGATATTCTG   251
Triticum_monococcum_linn_Mo4     TGGCCTCATGCCGATTATTTTTACGTACGCTGCTGGTTAGTCGTCCTCTGAGATATTCTG   251
Triticum_monococcum_linn_TL      TGGCCTCATGCCGATTATTTTTACGTACGCTGCTGGTTAGTCGTCCTCTGAGATATTCTG   251
Triticum_urartu_PI428222         TGGCCTCATGCCGATTATTTTTACGTACGCTGCTGGTTAGTCGTCCTCTGAGATATGCTG  1260
Triticum_urartu_PI428260         TGGCCTCATGCCGATTATTTTTACGTACGCTGCTGGTTAGTCGTCCTCTGAGATATGCTG  1260
Triticum_urartu_PI428266         TGGCCTCATGCCGATTATTTTTACGTACGCTGCTGGTTAGTCGTCCTCTGAGATATGCTG  1260
T.dicoccoides_As829              TGGCCTCATGCCGATTATTTTTACGTACGCTGCTGGTTAGTCGTCCTCTGAGATATGCTG  1260
T.dicoccoides_As836              TGGCCTCATGCCGATTATTTTTACGTACGCTGCTGGTTAGTCGTCCTCTGAGATATGCTG  1260
T.dicoccoides_As839              TGGCCTCATGCCGATTATTTTTACGTACGCTGCTGGTTAGTCGTCCTCTGAGATATGCTG  1260
T.dicoccum_PI272527              TGGCCTCATGCCGATTATTTTTACGTACGCTGCTGGTTAGTCGTCCTCTGAGATATGCTG  1260
T.dicoccum_PI193873              TGGCCTCATGCCGATTATTTTTACGTACGCTGCTGGTTAGTCGTCCTCTGAGATATGCTG  1260
T.dicoccum_PI272527              TGGCCTCATGCCGATTATTTTTACGTACGCTGCTGGTTAGTCGTCCTCTGAGATATGCTG  1260
T.durum_Dr8                      TGGCCTCATGCCGATTATTTTTACGTACGCTGCTGGTTAGTCGTCCTCTGAGATATGCTG  1260
T.durum_Simeto-2                 TGGCCTCATGCCGATTATTTTTACGTACGCTGCTGGTTAGTCGTCCTCTGAGATATGCTG  1260
T.durum_Club57                   TGGCCTCATGCCGATTATTTTTACGTACGCTGCTGGTTAGTCGTCCTCTGAGATATGCTG  1260
T.carthlicum_Tc                  TGGCCTCATGCCGATTATTTTTACGTACGCTGCTGGTTAGTCGTCCTCTGAGATATGCTG  1260
Hexaploid wheat                  TGGCCTCATGCCGATTATTTTTACGTACGCTGCTGGTTAGTCGTCCTCTGAGATATGCTG  1260

Triticum_boeoticum_boiss_Bo8     ACCGAGATATATGCATGCATGGG.CAGGCGTACAACGACGCTGCGGATGCCACCGACAAG   310
Triticum_monococcum_linn_Mo4     ACCGAGATATATGCATGCATGGG.CAGGCGTACAACGACGCTGCGGATGCCACCGACAAG   310
Triticum_monococcum_linn_TL      ACCGAGATATATGCATGCATGGG.CAGGCGTACAACGACGCTGCGGATGCCACCGACAAG   310
Triticum_urartu_PI428222         ACCGAGATATATGGGTGCATGGGACAGGCGTACAACGACGCTGCGGATGCTACCGACAAG  1320
Triticum_urartu_PI428260         ACCGAGATATATGGGTGCATGGGACAGGCGTACAACGACGCTGCGGATGCTACCGACAAG  1320
Triticum_urartu_PI428266         ACCGAGATATATGGGTGCATGGGACAGGCGTACAACGACGCTGCGGATGCTACCGACAAG  1320
T.dicoccoides_As829              ACCGAGATATATGGGTGCATGGGACAGGCGTACAACGACGCTGCGGATGCTACCGACAAG  1320
T.dicoccoides_As836              ACCGAGATATATGGGTGCATGGGACAGGCGTACAACGACGCTGCGGATGCTACCGACAAG  1320
T.dicoccoides_As839              ACCGAGATATATGGGTGCATGGGACAGGCGTACAACGACGCTGCGGATGCTACCGACAAG  1320
T.dicoccum_PI272527              ACCGAGATATATGGGTGCATGGGACAGGCGTACAACGACGCTGCGGATGCTACCGACAAG  1320
T.dicoccum_PI193873              ACCGAGATATATGGGTGCATGGGACAGGCGTACAACGACGCTGCGGATGCTACCGACAAG  1320
T.dicoccum_PI272527              ACCGAGATATATGGGTGCATGGGACAGGCGTACAACGACGCTGCGGATGCTACCGACAAG  1320
T.durum_Dr8                      ACCGAGATATATGGGTGCATGGGACAGGCGTACAACGACGCTGCGGATGCTACCGACAAG  1320
T.durum_Simeto-2                 ACCGAGATATATGGGTGCATGGGACAGGCGTACAACGACGCTGCGGATGCTACCGACAAG  1320
T.durum_Club57                   ACCGAGATATATGGGTGCATGGGACAGGCGTACAACGACGCTGCGGATGCTACCGACAAG  1320
T.carthlicum_Tc                  ACCGAGATATATGGGTGCATGGGACAGGCGTACAACGACGCTGCGGATGCTACCGACAAG  1320
Hexaploid wheat                  ACCGAGATATATGGGTGCATGGGACAGGCGTACAACGACGCTGCGGATGCTACCGACAAG  1320

Triticum_boeoticum_boiss_Bo8     GCCATCGACGGCGTGAAGGGGGTGGCCGACGAGCTGAAGAAGGGCGTGGCGGAGGCGGCG   370
Triticum_monococcum_linn_Mo4     GCCATCGACGGCGTGAAGGGGGTGGCCGACGAGCTGAAGAAGGGCGTGGCGGAGGCGGCG   370
Triticum_monococcum_linn_TL      GCCATCGACGGCGTGAAGGGGGTGGCCGACGAGCTGAAGAAGGGCGTGGCGGAGGCGGCG   370
Triticum_urartu_PI428222         GCCATCGACGGCGTGAAGGGGGTGGCCGACGAGTTGAAGAAGGGCGTGGCGGAGGCGGCT  1380
Triticum_urartu_PI428260         GCCATCGACGGCGTGAAGGGGGTGGCCGACGAGTTGAAGAAGGGCGTGGCGGAGGCGGCT  1380
Triticum_urartu_PI428266         GCCATCGACGGCGTGAAGGGGGTGGCCGACGAGTTGAAGAAGGGCGTGGCGGAGGCGGCT  1380
T.dicoccoides_As829              GCCATCGACGGCGTGAAGGGGGTGGCCGACGAGTTGAAGAAGGGCGTGGCGGAGGCGGCT  1380
T.dicoccoides_As836              GCCATCGACGGCGTGAAGGGGGTGGCCGACGAGTTGAAGAAGGGCGTGGCGGAGGCGGCT  1380
T.dicoccoides_As839              GCCATCGACGGCGTGAAGGGGGTGGCCGACGAGTTGAAGAAGGGCGTGGCGGAGGCGGCT  1380
T.dicoccum_PI272527              GCCATCGACGGCGTGAAGGGGGTGGCCGACGAGTTGAAGAAGGGCGTGGCGGAGGCGGCT  1380
T.dicoccum_PI193873              GCCATCGACGGCGTGAAGGGGGTGGCCGACGAGTTGAAGAAGGGCGTGGCGGAGGCGGCT  1380
T.dicoccum_PI272527              GCCATCGACGGCGTGAAGGGGGTGGCCGACGAGTTGAAGAAGGGCGTGGCGGAGGCGGCT  1380
T.durum_Dr8                      GCCATCGACGGCGTGAAGGGGGTGGCCGACGAGTTGAAGAAGGGCGTGGCGGAGGCGGCT  1380
T.durum_Simeto-2                 GCCATCGACGGCGTGAAGGGGGTGGCCGACGAGTTGAAGAAGGGCGTGGCGGAGGCGGCT  1380
T.durum_Club57                   GCCATCGACGGCGTGAAGGGGGTGGCCGACGAGTTGAAGAAGGGCGTGGCGGAGGCGGCT  1380
T.carthlicum_Tc                  GCCATCGACGGCGTGAAGGGGGTGGCCGACGAGTTGAAGAAGGGCGTGGCGGAGGCGGCT  1380
Hexaploid_wheat                  GCCATCGACGGCGTGAAGGGGGTGGCCGACGAGTTGAAGAAGGGCGTGGCGGAGGCGGCT  1380

Triticum_boeoticum_boiss_Bo8     GAGGCCGTCTCGGGCAACACCGAGAAGGCCGCGGAGGAAGCCGGCAAGGGCGCGAGCGAG   430
Triticum_monococcum_linn_Mo4     GAGGCCGTCTCGGGCAACACCGAGAAGGCCGCGGAGGAAGCCGGCAAGGGCGCGAGCGAG   430
Triticum_monococcum_linn_TL      GAGGCCGTCTCGGGCAACACCGAGAAGGCCGCGGAGGAAGCCGGCAAGGGCGCGAGCGAG   430
Triticum_urartu_PI428222         GAGGCCGTCTCGGGCAACACCGAGAAGGCCGCGGAGGAAGCCGGCAAAGGCGCGAGCGAG  1440
Triticum_urartu_PI428260         GAGGCCGTCTCGGGCAACACCGAGAAGGCCGCGGAGGAAGCCGGCAAAGGCGCGAGCGAG  1440
Triticum_urartu_PI428266         GAGGCCGTCTCGGGCAACACCGAGAAGGCCGCGGAGGAAGCCGGCAAAGGCGCGAGCGAG  1440
T.dicoccoides_As829              GAGGCCGTCTCGGGCAACACCGAGAAGGCCGCGGAGGAAGCCGGCAAAGGCGCGAGCGAG  1440
T.dicoccoides_As836              GAGGCCGTCTCGGGCAACACCGAGAAGGCCGCGGAGGAAGCCGGCAAAGGCGCGAGCGAG  1440
T.dicoccoides_As839              GAGGCCGTCTCGGGCAACACCGAGAAGGCCGCGGAGGAAGCCGGCAAAGGCGCGAGCGAG  1440
T.dicoccum_PI272527              GAGGCCGTCTCGGGCAACACCGAGAAGGCCGCGGAGGAAGCCGGCAAAGGCGCGAGCGAG  1440
T.dicoccum_PI193873              GAGGCCGTCTCGGGCAACACCGAGAAGGCCGCGGAGGAAGCCGGCAAAGGCGCGAGCGAG  1440
T.dicoccum_PI272527              GAGGCCGTCTCGGGCAACACCGAGAAGGCCGCGGAGGAAGCCGGCAAAGGCGCGAGCGAG  1440
T.durum_Dr8                      GAGGCCGTCTCGGGCAACACCGAGAAGGCCGCGGAGGAAGCCGGCAAAGGCGCGAGCGAG  1440
T.durum_Simeto-2                 GAGGCCGTCTCGGGCAACACCGAGAAGGCCGCGGAGGAAGCCGGCAAAGGCGCGAGCGAG  1440
T.durum_Club57                   GAGGCCGTCTCGGGCAACACCGAGAAGGCCGCGGAGGAAGCCGGCAAAGGCGCGAGCGAG  1440
T.carthlicum_Tc                  GAGGCCGTCTCGGGCAACACCGAGAAGGCCGCGGAGGAAGCCGGCAAAGGCGCGAGCGAG  1440
Hexaploid wheat                  GAGGCCGTCTCGGGCAACACCGAGAAGGCCGCGGAGGAAGCCGGCAAAGGCGCGAGCGAG  1440
```

```
Triticum_boeoticum_boiss_Bo8    GTCGACGACAAGGCCAAGGACTTCGGCGAGCAGGCGAAGAAGGCGACGGAGGAGGCGTGG     490
Triticum_monococcum_linn_Mo4    GTCGACGACAAGGCCAAGGACTTCGGCGAGCAGGCGAAGAAGGCGACGGAGGAGGCGTGG     490
Triticum_monococcum_linn_TL     GTCGACGACAAGGCCAAGGACTTCGGCGAGCAGGCGAAGAAGGCGACGGAGGAGGCGTGG     490
Triticum_urartu_PI428222        GTGGACGAGAAGGCCAAGGACTTCGGCGAGCAGGCGAAGAAGGCGACGGAGGAGGCGTGG    1500
Triticum_urartu_PI428260        GTGGACGAGAAGGCCAAGGACTTCGGCGAGCAGGCGAAGAAGGCGACGGAGGAGGCGTGG    1500
Triticum_urartu_PI428266        GTGGACGAGAAGGCCAAGGACTTCGGCGAGCAGGCGAAGAAGGCGACGGAGGAGGCGTGG    1500
T.dicoccoides_As829             GTGGACGAGAAGGCCAAGGACTTCGGCGAGCAGGCGAAGAAGGCGACGGAGGAGGCGTGG    1500
T.dicoccoides_As836             GTGGACGAGAAGGCCAAGGACTTCGGCGAGCAGGCGAAGAAGGCGACGGAGGAGGCGTGG    1500
T.dicoccoides_As839             GTGGACGAGAAGGCCAAGGACTTCGGCGAGCAGGCGAAGAAGGCGACGGAGGAGGCGTGG    1500
T.dicoccum_PI272527             GTGGACGAGAAGGCCAAGGACTTCGGCGAGCAGGCGAAGAAGGCGACGGAGGAGGCGTGG    1500
T.dicoccum_PI193873             GTGGACGAGAAGGCCAAGGACTTCGGCGAGCAGGCGAAGAAGGCGACGGAGGAGGCGTGG    1500
T.dicoccum_PI272527             GTGGACGAGAAGGCCAAGGACTTCGGCGAGCAGGCGAAGAAGGCGACGGAGGAGGCGTGG    1500
T.durum_Dr8                     GTGGACGAGAAGGCCAAGGACTTCGGCGAGCAGGCGAAGAAGGCGACGGAGGAGGCGTGG    1500
T.durum_Simeto-2                GTGGACGAGAAGGCCAAGGACTTCGGCGAGCAGGCGAAGAAGGCGACGGAGGAGGCGTGG    1500
T.durum_Club57                  GTGGACGAGAAGGCCAAGGACTTCGGCGAGCAGGCGAAGAAGGCGACGGAGGAGGCGTGG    1500
T.carthlicum_Tc                 GTGGACGAGAAGGCCAAGGACTTCGGCGAGCAGGCGAAGAAGGCGACGGAGGAGGCGTGG    1500
Hexaploid wheat                 GTGGACGAGAAGGCCAAGGACTTCGGCGAGCAGGCGAAGAAGGCGACGGAGGAGGCGTGG    1500

Triticum_boeoticum_boiss_Bo8    GACGGCGCCAAGGACGCCGCACAGGGCATCACGGACAAGGTCGCGGACGCGGCCAAAAAG     550
Triticum_monococcum_linn_Mo4    GACGGCGCCAAGGACGCCGCACAGGGCATCACGGACAAGGTCGCGGACGCGGCCAAAAAG     550
Triticum_monococcum_linn_TL     GACGGCGCCAAGGACGCCGCACAGGGCATCACGGACAAGGTCGCGGACGCGGCCAAAAAG     550
Triticum_urartu_PI428222        GACGGCGCCAAGGACGCCGCACAGGGCATCACGGACAAGGTCGCGGACGCGGCCAAAAAG    1560
Triticum_urartu_PI428260        GACGGCGCCAAGGACGCCGCACAGGGCATCACGGACAAGGTCGCGGACGCGGCCAAAAAG    1560
Triticum_urartu_PI428266        GACGGCGCCAAGGACGCCGCACAGGGCATCACGGACAAGGTCGCGGACGCGGCCAAAAAG    1560
T.dicoccoides_As829             GACGGCGCCAAGGACGCCGCACAGGGCATCACGGACAAGGTCGCGGACGCGGCCAAAAAG    1560
T.dicoccoides_As836             GACGGCGCCAAGGACGCCGCACAGGGCATCACGGACAAGGTCGCGGACGCGGCCAAAAAG    1560
T.dicoccoides_As839             GACGGCGCCAAGGACGCCGCACAGGGCATCACGGACAAGGTCGCGGACGCGGCCAAAAAG    1560
T.dicoccum_PI272527             GACGGCGCCAAGGACGCCGCACAGGGCATCACGGACAAGGTCGCGGACGCGGCCAAAAAG    1560
T.dicoccum_PI193873             GACGGCGCCAAGGACGCCGCACAGGGCATCACGGACAAGGTCGCGGACGCGGCCAAAAAG    1560
T.dicoccum_PI272527             GACGGCGCCAAGGACGCCGCACAGGGCATCACGGACAAGGTCGCGGACGCGGCCAAAAAG    1560
T.durum_Dr8                     GACGGCGCCAAGGACGCCGCACAGGGCATCACGGACAAGGTCGCGGACGCGGCCAAAAAG    1560
T.durum_Simeto-2                GACGGCGCCAAGGACGCCGCACAGGGCATCACGGACAAGGTCGCGGACGCGGCCAAAAAG    1560
T.durum_Club57                  GACGGCGCCAAGGACGCCGCACAGGGCATCACGGACAAGGTCGCGGACGCGGCCAAAAAG    1560
T.carthlicum_Tc                 GACGGCGCCAAGGACGCCGCACAGGGCATCACGGACAAGGTCGCGGACGCGGCCAAAAAG    1560
Hexaploid_wheat                 GACGGCGCCAAGGACGCCGCACAGGGCATCACGGACAAGGTCGCGGACGCGGCCAAAAAG    1560

Triticum_boeoticum_boiss_Bo8    GAAGCAAACTAA................................................     562
Triticum_monococcum_linn_Mo4    GAAGCAAACTAA................................................     562
Triticum_monococcum_linn_TL     GAAGCAAACTAA................................................     562
Triticum_urartu_PI428222        GAAGCTAACTAAGCTAACAATGCGTTGACTAGTCCGATATGTATCTCTCAATTCATTTTC    1620
Triticum_urartu_PI428260        GAAGCTAACTAAGCTAACAATGCGTTGACTAGTCCGATATGTATCTCTCAATTCATTTTC    1620
Triticum_urartu_PI428266        GAAGCTAACTAAGCTAACAATGCGTTGACTAGTCCGATATGTATCTCTCAATTCATTTTC    1620
T.dicoccoides_As829             GAAGCTAACTAAGCTAACAATGCGTTGACTAGTCCGATATGTATCTCTCAATTCATTTTC    1620
T.dicoccoides_As836             GAAGCTAACTAAGCTAACAATGCGTTGACTAGTCCGATATGTATCTCTCAATTCATTTTC    1620
T.dicoccoides_As839             GAAGCTAACTAAGCTAACAATGCGTTGACTAGTCCGATATGTATCTCTCAATTCATTTTC    1620
T.dicoccum_PI272527             GAAGCTAACTAAGCTAACAATGCGTTGACTAGTCCGATATGTATCTCTCAATTCATTTTC    1620
T.dicoccum_PI193873             GAAGCTAACTAAGCTAACAATGCGTTGACTAGTCCGATATGTATCTCTCAATTCATTTTC    1620
T.dicoccum_PI272527             GAAGCTAACTAAGCTAACAATGCGTTGACTAGTCCGATATGTATCTCTCAATTCATTTTC    1620
T.durum_Dr8                     GAAGCTAACTAAGCTAACAATGCGTTGACTAGTCCGATATGTATCTCTCAATTCATTTTC    1620
T.durum_Simeto-2                GAAGCTAACTAAGCTAACAATGCGTTGACTAGTCCGATATGTATCTCTCAATTCATTTTC    1620
T.durum_Club57                  GAAGCTAACTAAGCTAACAATGCGTTGACTAGTCCGATATGTATCTCTCAATTCATTTTC    1620
T.carthlicum_Tc                 GAAGCTAACTAAGCTAACAATGCGTTGACTAGTCCGATATGTATCTCTCAATTCATTTTC    1620
Hexaploid wheat                 GAAGCTAACTAAGCTAACAATGCGTTGACTAGTCCGATATGTATCTCTCAATTCATTTTC    1620

Triticum_boeoticum_boiss_Bo8    ............................................................     562
Triticum_monococcum_linn_Mo4    ............................................................     562
Triticum_monococcum_linn_TL     ............................................................     562
Triticum_urartu_PI428222        CATTGTAAGAAGGGTTGTAAGAATGCATATACGTACTTTGGTACAAGAAGAGATAAAATA    1680
Triticum_urartu_PI428260        CATTGTAAGAAGGGTTGTAAGAATGCATATACGTACTTTGGTACAAGAAGAGATAAAATA    1680
Triticum_urartu_PI428266        CATTGTAAGAAGGGTTGTAAGAATGCATATACGTACTTTGGTACAAGAAGAGATAAAATA    1680
T.dicoccoides_As829             CATTGTAAGAAGGGTTGTAAGAATGCATATACGTACTTTGGTACAAGAAGAGATAAAATA    1680
T.dicoccoides_As836             CATTGTAAGAAGGGTTGTAAGAATGCATATACGTACTTTGGTACAAGAAGAGATAAAATA    1680
T.dicoccoides_As839             CATTGTAAGAAGGGTTGTAAGAATGCATATACGTACTTTGGTACAAGAAGAGATAAAATA    1680
T.dicoccum_PI272527             CATTGTAAGAAGGGTTGTAAGAATGCATATACGTACTTTGGTACAAGAAGAGATAAAATA    1680
T.dicoccum_PI193873             CATTGTAAGAAGGGTTGTAAGAATGCATATACGTACTTTGGTACAAGAAGAGATAAAATA    1680
T.dicoccum_PI272527             CATTGTAAGAAGGGTTGTAAGAATGCATATACGTACTTTGGTACAAGAAGAGATAAAATA    1680
T.durum_Dr8                     CATTGTAAGAAGGGTTGTAAGAATGCATATACGTACTTTGGTACAAGAAGAGATAAAATA    1680
T.durum_Simeto-2                CATTGTAAGAAGGGTTGTAAGAATGCATATACGTACTTTGGTACAAGAAGAGATAAAATA    1680
T.durum_Club57                  CATTGTAAGAAGGGTTGTAAGAATGCATATACGTACTTTGGTACAAGAAGAGATAAAATA    1680
T.carthlicum_Tc                 CATTGTAAGAAGGGTTGTAAGAATGCATATACGTACTTTGGTACAAGAAGAGATAAAATA    1680
Hexaploid wheat                 CATTGTAAGAAGGGTTGTAAGAATGCATATACGTACTTTGGTACAAGAAGAGATAAAATA    1680
```

```
Triticum_boeoticum_boiss_Bo8       ............................................................  562
Triticum_monococcum_linn_Mo4       ............................................................  562
Triticum_monococcum_linn_TL        ............................................................  562
Triticum_urartu_PI428222           GCTGCATTTATTTTCTGACATACAGGATTACCGCCCTGTTAATGTCAAACGCAATAAAGA 1740
Triticum_urartu_PI428260           GCTGCATTTATTTTCTGACATACAGGATTACCGCCCTGTTAATGTCAAACGCAATAAAGA 1740
Triticum_urartu_PI428266           GCTGCATTTATTTTCTGACATACAGGATTACCGCCCTGTTAATGTCAAACGCAATAAAGA 1740
T.dicoccoides_As829                GCTGCATTTATTTTCTGACATACAGGATTACCGCCCTGTTAATGTCAAACGCAATAAAGA 1740
T.dicoccoides_As836                GCTGCATTTATTTTCTGACATACAGGATTACCGCCCTGTTAATGTCAAACGCAATAAAGA 1740
T.dicoccoides_As839                GCTGCATTTATTTTCTGACATACAGGATTACCGCCCTGTTAATGTCAAACGCAATAAAGA 1740
T.dicoccum_PI272527                GCTGCATTTATTTTCTGACATACAGGATTACCGCCCTGTTAATGTCAAACGCAATAAAGA 1740
T.dicoccum_PI193873                GCTGCATTTATTTTCTGACATACAGGATTACCGCCCTGTTAATGTCAAACGCAATAAAGA 1740
T.dicoccum_PI272527                GCTGCATTTATTTTCTGACATACAGGATTACCGCCCTGTTAATGTCAAACGCAATAAAGA 1740
T.durum_Dr8                        GCTGCATTTATTTTCTGACATACAGGATTACCGCCCTGTTAATGTCAAACGCAATAAAGA 1740
T.durum_Simeto-2                   GCTGCATTTATTTTCTGACATACAGGATTACCGCCCTGTTAATGTCAAACGCAATAAAGA 1740
T.durum_Club57                     GCTGCATTTATTTTCTGACATACAGGATTACCGCCCTGTTAATGTCAAACGCAATAAAGA 1740
T.carthlicum_Tc                    GCTGCATTTATTTTCTGACATACAGGATTACCGCCCTGTTAATGTCAAACGCAATAAAGA 1740
Hexaploid_wheat                    GCTGCATTTATTTTCTGACATACAGGATTACCGCCCTGTTAATGTCAAACGCAATAAAGA 1740
```

```
Triticum_boeoticum_boiss_Bo8       ............................................................  562
Triticum_monococcum_linn_Mo4       ............................................................  562
Triticum_monococcum_linn_TL        ............................................................  562
Triticum_urartu_PI428222           AAATGATGCTAAAAGGTTTACATAATAGCTATGCGTGAGTTGCTTGAATTTCGAATTCGA 1800
Triticum_urartu_PI428260           AAATGATGCTAAAAGGTTTACATAATAGCTATGCGTGAGTTGCTTGAATTTCGAATTCGA 1800
Triticum_urartu_PI428266           AAATGATGCTAAAAGGTTTACATAATAGCTATGCGTGAGTTGCTTGAATTTCGAATTCGA 1800
T.dicoccoides_As829                AAATGATGCTAAAAGGTTTACATAATAGCTATGCGTGAGTTGCTTGAATTTCGAATTCGA 1800
T.dicoccoides_As836                AAATGATGCTAAAAGGTTTACATAATAGCTATGCGTGAGTTGCTTGAATTTCGAATTCGA 1800
T.dicoccoides_As839                AAATGATGCTAAAAGGTTTACATAATAGCTATGCGTGAGTTGCTTGAATTTCGAATTCGA 1800
T.dicoccum_PI272527                AAATGATGCTAAAAGGTTTACATAATAGCTATGCGTGAGTTGCTTGAATTTCGAATTCGA 1800
T.dicoccum_PI193873                AAATGATGCTAAAAGGTTTACATAATAGCTATGCGTGAGTTGCTTGAATTTCGAATTCGA 1800
T.dicoccum_PI272527                AAATGATGCTAAAAGGTTTACATAATAGCTATGCGTGAGTTGCTTGAATTTCGAATTCGA 1800
T.durum_Dr8                        AAATGATGCTAAAAGGTTTACATAATAGCTATGCGTGAGTTGCTTGAATTTCGAATTCGA 1800
T.durum_Simeto-2                   AAATGATGCTAAAAGGTTTACATAATAGCTATGCGTGAGTTGCTTGAATTTCGAATTCGA 1800
T.durum_Club57                     AAATGATGCTAAAAGGTTTACATAATAGCTATGCGTGAGTTGCTTGAATTTCGAATTCGA 1800
T.carthlicum_Tc                    AAATGATGCTAAAAGGTTTACATAATAGCTATGCGTGAGTTGCTTGAATTTCGAATTCGA 1800
Hexaploid wheat                    AAATGATGCTAAAAGGTTTACATAATAGCTATGCGTGAGTTGCTTGAATTTCGAATTCGA 1800
```

```
Triticum_boeoticum_boiss_Bo8       .........................................                     562
Triticum_monococcum_linn_Mo4       .........................................                     562
Triticum_monococcum_linn_TL        .........................................                     562
Triticum_urartu_PI428222           GTCGGTCGATTCGTGGAAGAAGGAAGGAGGAGACGAGGTGT                    1841
Triticum_urartu_PI428260           GTCGGTCGATTCGTGGAAGAAGGAAGGAGGAGACGAGGTGT                    1841
Triticum_urartu_PI428266           GTCGGTCGATTCGTGGAAGAAGGAAGGAGGAGACGAGGTGT                    1841
T.dicoccoides_As829                GTCGGTCGATTCGTGGAAGAAGGAAGGAGGAGACGAGGTGT                    1841
T.dicoccoides_As836                GTCGGTCGATTCGTGGAAGAAGGAAGGAGGAGACGAGGTGT                    1841
T.dicoccoides_As839                GTCGGTCGATTCGTGGAAGAAGGAAGGAGGAGACGAGGTGT                    1841
T.dicoccum_PI272527                GTCGGTCGATTCGTGGAAGAAGGAAGGAGGAGACGAGGTGT                    1841
T.dicoccum_PI193873                GTCGGTCGATTCGTGGAAGAAGGAAGGAGGAGACGAGGTGT                    1841
T.dicoccum_PI272527                GTCGGTCGATTCGTGGAAGAAGGAAGGAGGAGACGAGGTGT                    1841
T.durum_Dr8                        GTCGGTCGATTCGTGGAAGAAGGAAGGAGGAGACGAGGTGT                    1841
T.durum_Simeto-2                   GTCGGTCGATTCGTGGAAGAAGGAAGGAGGAGACGAGGTGT                    1841
T.durum_Club57                     GTCGGTCGATTCGTGGAAGAAGGAAGGAGGAGACGAGGTGT                    1841
T.carthlicum_Tc                    GTCGGTCGATTCGTGGAAGAAGGAAGGAGGAGACGAGGTGT                    1841
Hexaploid_wheat                    GTCGGTCGATTCGTGGAAGAAGGAAGGAGGAGACGAGGTGT                    1841
```

Supplementary Fig. S5 Sequence alignments of the *Wcor15-2A* from hexaploid wheat, tetraploid wheat, and *T. urartu, T. monococcum linn* and *T. boeoticum boiss.*

```
Aegilops_tauschii_Coss_As_80      ATACTTGTTGGAAAAATAGATAAAAATGAATGTACTATCTAGAATTAAAATACGCCCAAA   60
Aegilops_tauschii_Coss_As_77      ATACTTGTTGGAAAAATAGATAAAAATGAATGTACTATCTAGAATTAAAATACGCCCAAA   60
Aegilops_tauschii_Coss_As_2392    ATACTTGTTGGAAAAATAGATAAAAATGAATGTACTATCTAGAATTAAAATACGCCCAAA   60
Aegilops_tauschii_Coss_As_2386    ATACTTGTTGGAAAAATAGATAAAAATGAATGTACTATCTAGAATTAAAATACGCCCAAA   60
Aegilops_tauschii_Coss_As_2387    ATACTTGTTGGAAAAATAGATAAAAATGAATGTACTATCTAGAATTAAAATACGCCCAAA   60
Aegilops_tauschii_Coss_As_2388    ATACTTGTTGGAAAAATAGATAAAAATGAATGTACTATCTAGAATTAAAATACGCCCAAA   60
Hexaploid_wheat                   ATACTTGTTGGAAAAATAGATAAAAATGAATGTACTATCTAGAATTAAAATACGCCCAAA   60

Aegilops_tauschii_Coss_As_80      TACATCATTTTTTCTGATAAATATTTTCAGACGGATGGAGTACATATTTATATCTAACTA   120
Aegilops_tauschii_Coss_As_77      TACATCATTTTTTCTGATAAATATTTTCAGACGGATGGAGTACATATTTATATCTAACTA   120
Aegilops_tauschii_Coss_As_2392    TACATCATTTTTTCTGATAAATATTTTCAGACGGATGGAGTACATATTTATATCTAACTA   120
Aegilops_tauschii_Coss_As_2386    TACATCATTTTTTCTGATAAATATTTTCAGACGGATGGAGTACATATTTATATCTAACTA   120
Aegilops_tauschii_Coss_As_2387    TACATCATTTTTTCTGATAAATATTTTCAGACGGATGGAGTACATATTTATATCTAACTA   120
Aegilops_tauschii_Coss_As_2388    TACATCATTTTTTCTGATAAATATTTTCAGACGGATGGAGTACATATTTATATCTAACTA   120
Hexaploid_wheat                   TACATCATTTTTTCTGATAAATATTTTCAGACGGATGGAGTACATATTTATATCTAACTA   120

Aegilops_tauschii_Coss_As_80      TTATACTTGCCGCATATAAGTTTAATTATAGATGACGTGCCAACATCATATAGCCAGCAG   180
Aegilops_tauschii_Coss_As_77      TTATACTTGCCGCATATAAGTTTAATTATAGATGACGTGCCAACATCATATAGCCAGCAG   180
Aegilops_tauschii_Coss_As_2392    TTATACTTGCCGCATATAAGTTTAATTATAGATGACGTGCCAACATCATATAGCCAGCAG   180
Aegilops_tauschii_Coss_As_2386    TTATACTTGCCGCATATAAATTTAATTATAGATGACGTGCCAACATCATATAGCCAGCAG   180
Aegilops_tauschii_Coss_As_2387    TTATACTTGCCGCATATAAATTTAATTATAGATGACGTGCCAACATCATATAGCCAGCAG   180
Aegilops_tauschii_Coss_As_2388    TTATACTTGCCGCATATAAATTTAATTATAGATGACGTGCCAACATCATATAGCCAGCAG   180
Hexaploid_wheat                   TTATACTTGCCGCATATAAATTTAATTATAGATGACGTGCCAACATCATATAGCCAGCAG   180

Aegilops_tauschii_Coss_As_80      CCGGCTATGCTATTAAACATGCTGTGGACCATGTATCGGCGACGTCATAATTGTTCATTC   240
Aegilops_tauschii_Coss_As_77      CCGGCTATGCTATTAAACATGCTGTGGACCATGTATCGGCGACGTCATAATTGTTCATTC   240
Aegilops_tauschii_Coss_As_2392    CCGGCTATGCTATTAAACATGCTGTGGACCATGTATCGGCGACGTCATAATTGTTCATTC   240
Aegilops_tauschii_Coss_As_2386    CCGGCTATGCTATTAAACATGTTGTGGACCATGTATCGGCGACGTCATTATTGTTCATTC   240
Aegilops_tauschii_Coss_As_2387    CCGGCTATGCTATTAAACATGTTGTGGACCATGTATCGGCGACGTCATTATTGTTCATTC   240
Aegilops_tauschii_Coss_As_2388    CCGGCTATGCTATTAAACATGTTGTGGACCATGTATCGGCGACGTCATTATTGTTCATTC   240
Hexaploid_wheat                   CCGGCTATGCTATTAAACATGTTGTGGACCATGTATCGGCGACGTCATTATTGTTCATTC   240

Aegilops_tauschii_Coss_As_80      AATCACTTTTCAGTTTAATATTTTCTACAGATGGGATATAAATATGGCTGAACTTACCCT   300
Aegilops_tauschii_Coss_As_77      AATCACTTTTCAGTTTAATATTTTCTACAGATGGGATATAAATATGGCTGAACTTACCCT   300
Aegilops_tauschii_Coss_As_2392    AATCACTTTCCAGTTTAATATTTTCTACAGATGGGATATAAATATGGCTGAACTTACCCT   300
Aegilops_tauschii_Coss_As_2386    AATCACTTTCCAGTTTAATATTTTCTACAGATGGGATATAAATATGGCTGAACTTACCCT   300
Aegilops_tauschii_Coss_As_2387    AATCACTTTCCAGTTTAATATTTTCTACAGATGGGATATAAATATGGCTGAACTTACCCT   300
Aegilops_tauschii_Coss_As_2388    AATCACTTTCCAGTTTAATATTTTCTACAGATGGGATATAAATATGGCTGAACTTACCCT   300
Hexaploid_wheat                   AATCACTTTCCAGTTTAATATTTTCTACAGATGGGATATAAATATGGCTGAACTTACCCT   300

Aegilops_tauschii_Coss_As_80      TGATTAATCGACGGCCGGTATCAAATATCTCCTGTTATCATAAGTTGAAGCATCTTCAAA   360
Aegilops_tauschii_Coss_As_77      TGATTAATCGACGGCCGGTATCAAATATCTCCTGTTATCATAAGTTGAAGCATCTTCAAA   360
Aegilops_tauschii_Coss_As_2392    TGATTAATCGACGGCCGGTATCAAATATCTCCTGTTATCATAAGTTGAAGCATCTTCAAA   360
Aegilops_tauschii_Coss_As_2386    TGATTAATCGACGGCCGGTATCAAATATCTCCTGTTATCATAAGTTGAAGCATCTTCAAA   360
Aegilops_tauschii_Coss_As_2387    TGATTAATCGACGGCCGGTATCAAATATCTCCTGTTATCATAAGTTGAAGCATCTTCAAA   360
Aegilops_tauschii_Coss_As_2388    TGATTAATCGACGGCCGGTATCAAATATCTCCTGTTATCATAAGTTGAAGCATCTTCAAA   360
Hexaploid_wheat                   TGATTAATCGACGGCCGGTATCAAATATCTCCTGTTATCATAAGTTGAAGCATCTTCAAA   360

Aegilops_tauschii_Coss_As_80      GAGCTCCTGACAAAACCTTGGTTGTACGTAACCTTCAAACGTGGACAGTAGGATTTGTTT   420
Aegilops_tauschii_Coss_As_77      GAGCTCCTGACAAAACCTTGGTTGTACGTAACCTTCAAACGTGGACAGTAGGATTTGTTT   420
Aegilops_tauschii_Coss_As_2392    GAGCTCCTGACAAAACCTTGGTTGTACGTAACCTTCAAACGTGGACAGTAGGATTTGTTT   420
Aegilops_tauschii_Coss_As_2386    GAGCTCCTGACAAAACCTTGGTTGTACGTAACCTTCAAACGTGGACAGTAGGATTTGTTT   420
Aegilops_tauschii_Coss_As_2387    GAGCTCCTGACAAAACCTTGGTTGTACGTAACCTTCAAACGTGGACAGTAGGATTTGTTT   420
Aegilops_tauschii_Coss_As_2388    GAGCTCCTGACAAAACCTTGGTTGTACGTAACCTTCAAACGTGGACAGTAGGATTTGTTT   420
Hexaploid_wheat                   GAGCTCCTGACAAAACCTTGGTTGTACGTAACCTTCAAACGTGGACAGTAGGATTTGTTT   420

Aegilops_tauschii_Coss_As_80      CTTCGATCCGCTCCAAACAGGTCATGTAGGTATACGTGTGTTCCCGTGAGAGGCGTGTGG   480
Aegilops_tauschii_Coss_As_77      CTTCGATCCGCTCCAAACAGGTCATGTAGGTATACGTGTGTTCCCGTGAGAGGCGTGTGG   480
Aegilops_tauschii_Coss_As_2392    CTTCGATCCGCTCCAAACAGGTCATGTAGGTATACGTGTGTTCCCGTGAGAGGCGTGTGG   480
Aegilops_tauschii_Coss_As_2386    CTTCGATCCGCTCCAAACAGGTCATGTAGGTATACGTGTGTTCCCGTGAGAGGCGTGTGG   480
Aegilops_tauschii_Coss_As_2387    CTTCGATCCGCTCCAAACAGGTCATGTAGGTATACGTGTGTTCCCGTGAGAGGCGTGTGG   480
Aegilops_tauschii_Coss_As_2388    CTTCGATCCGCTCCAAACAGGTCATGTAGGTATACGTGTGTTCCCGTGAGAGGCGTGTGG   480
Hexaploid_wheat                   CTTCGATCCGCTCCAAACAGGTCATGTAGGTATACGTGTGTTCCCGTGAGAGGCGTGTGG   480

Aegilops_tauschii_Coss_As_80      GGTAAGCAGCTAGTAGTAGCAAGCA.GGGTGCAGCAGATACCCATTTCTTTTCTTTTATT   539
Aegilops_tauschii_Coss_As_77      GGTAAGCAGCTAGTAGTAGCAAGCA.GGGTGCAGCAGATACCCATTTCTTTTCTTTTATT   539
Aegilops_tauschii_Coss_As_2392    GGTAAGCAGCTAGTAGTAGCAAGCA.GGGTGCAGCAGATACCCATTTCTTTTCTTTTATT   539
Aegilops_tauschii_Coss_As_2386    GGTAAGCAGCTAGTAGTAGCAAGCATGGGTGCAGCAGATACCCATTTCTTTTCTTTTATT   540
Aegilops_tauschii_Coss_As_2387    GGTAAGCAGCTAGTAGTAGCAAGCATGGGTGCAGCAGATACCCATTTCTTTTCTTTTATT   540
Aegilops_tauschii_Coss_As_2388    GGTAAGCAGCTAGTAGTAGCAAGCATGGGTGCAGCAGATACCCATTTCTTTTCTTTTATT   540
Hexaploid_wheat                   GGTAAGCAGCTAGTAGTAGCAAGCATGGGTGCAGCAGATACCCATTTCTTTTCTTTTATT   540

Aegilops_tauschii_Coss_As_80      GGTATGAGAAATTCGACCGTACATTACCACATAACCTTGTCGACAGTTCTCCGCGCACGG   599
Aegilops_tauschii_Coss_As_77      GGTATGAGAAATTCGACCGTACATTACCACATAACCTTGTCGACAGTTCTCCGCGCACGG   599
Aegilops_tauschii_Coss_As_2392    GGTATGAGAAATTCGACCGTACATTACCACATAACCTTGTCGACAGTTCTCCGCGCACGG   599
Aegilops_tauschii_Coss_As_2386    GGTATGAGAAATTCGACCGTACATTACCACATAACCTTGTCGACAGTTCTCCGCGCACGG   600
Aegilops_tauschii_Coss_As_2387    GGTATGAGAAATTCGACCGTACATTACCACATAACCTTGTCGACAGTTCTCCGCGCACGG   600
Aegilops_tauschii_Coss_As_2388    GGTATGAGAAATTCGACCGTACATTACCACATAACCTTGTCGACAGTTCTCCGCGCACGG   600
Hexaploid_wheat                   GGTATGAGAAATTCGACCGTACATTACCACATAACCTTGTCGACAGTTCTCCGCGCACGG   600
```

```
Aegilops_tauschii_Coss_As_80     CTGCACCACTCAACCTTGTCGACCGTCGATTGTCGCCATGCGCCGGAGCGATCGCCGCCC   659
Aegilops_tauschii_Coss_As_77     CTGCACCACTCAACCTTGTCGACCGTCGATTGTCGCCATGCGCCGGAGCGATCGCCGCCC   659
Aegilops_tauschii_Coss_As_2392   CTGCACCACTCAACCTTGTCGACCGTCGATTGTCGCCATGCGCCGGAGCGATCGCCGCCC   659
Aegilops_tauschii_Coss_As_2386   CTGCACCACTCAACCTTGTCGACCGTCGATTGTCGCCATGCGCCGGAGCGATCGCCGCCC   660
Aegilops_tauschii_Coss_As_2387   CTGCACCACTCAACCTTGTCGACCGTCGATTGTCGCCATGCGCCGGAGCGATCGCCGCCC   660
Aegilops_tauschii_Coss_As_2388   CTGCACCACTCAACCTTGTCGACCGTCGATTGTCGCCATGCGCCGGAGCGATCGCCGCCC   660
Hexaploid_wheat                  CTGCACCACTCAACCTTGTCGACCGTCGATTGTCGCCATGCGCCGGAGCGATCGCCGCCC   660

Aegilops_tauschii_Coss_As_80     GACGTGTCGCCAGCCACCTCACCCTTCGTCCCTCCGCGTCCAGCCTATAAAAACGACGAT   719
Aegilops_tauschii_Coss_As_77     GACGTGTCGCCAGCCACCTCACCCTTCGTCCCTCCGCGTCCAGCCTATAAAAACGACGAT   719
Aegilops_tauschii_Coss_As_2392   GACGTGTCGCCAGCCACCTCACCCTTCGTCCCTCCGCGTCCAGCCTATAAAAACGACGAT   719
Aegilops_tauschii_Coss_As_2386   GACGTGTCGCCAGCCACCTCACCCTTCGCCCCTCCGCGTCCAGCCTATAAAAACGACGAT   720
Aegilops_tauschii_Coss_As_2387   GACGTGTCGCCAGCCACCTCACCCTTCGCCCCTCCGCGTCCAGCCTATAAAAACGACGAT   720
Aegilops_tauschii_Coss_As_2388   GACGTGTCGCCAGCCACCTCACCCTTCGCCCCTCCGCGTCCAGCCTATAAAAACGACGAT   720
Hexaploid_wheat                  GACGTGTCGCCAGCCACCTCACCCTTCGCCCCTCCGCGTCCAGCCTATAAAAACGACGAT   720

Aegilops_tauschii_Coss_As_80     GCACCTCTCGTCCTCCCATCGAACTGCAAGTGAAGCTCACACGACCTAGCCTACCCTACC   779
Aegilops_tauschii_Coss_As_77     GCACCTCTCGTCCTCCCATCGAACTGCAAGTGAAGCTCACACGACCTAGCCTACCCTACC   779
Aegilops_tauschii_Coss_As_2392   GCACCTCTCGTCCTCCCATCGAACTGCAAGTGAAGCTCACACGACCTAGCCTACCCTACC   779
Aegilops_tauschii_Coss_As_2386   GCACCTCTCGTCCTCCCATCGGACTGCAAGTGAAGCTCACACGACCTAGCCTACCCTACC   780
Aegilops_tauschii_Coss_As_2387   GCACCTCTCGTCCTCCCATCGGACTGCAAGTGAAGCTCACACGACCTAGCCTACCCTACC   780
Aegilops_tauschii_Coss_As_2388   GCACCTCTCGTCCTCCCATCGGACTGCAAGTGAAGCTCACACGACCTAGCCTACCCTACC   780
Hexaploid_wheat                  GCACCTCTCGTCCTCCCATCGGACTGCAAGTGAAGCTCACACGACCTAGCCTACCCTACC   780

Aegilops_tauschii_Coss_As_80     CACCCATCCATCAGCAGTTTTTCTATCGGCCAATGGCTTCTTCTTCCGTGCTGCTCGGAG   839
Aegilops_tauschii_Coss_As_77     CACCCATCCATCAGCAGTTTTTCTATCGGCCAATGGCTTCTTCTTCCGTGCTGCTCGGAG   839
Aegilops_tauschii_Coss_As_2392   CACCCATCCATCAGCAGTTTTTCTATCGGCCAATGGCTTCTTCTTCCGTGCTGCTCGGAG   839
Aegilops_tauschii_Coss_As_2386   CACCCATCCATCAGCAGTTTTTCTATCGGCCAATGGCTTCTTCTTCTGTGCTGCTCGGAG   840
Aegilops_tauschii_Coss_As_2387   CACCCATCCATCAGCAGTTTTTCTATCGGCCAATGGCTTCTTCTTCTGTGCTGCTCGGAG   840
Aegilops_tauschii_Coss_As_2388   CACCCATCCATCAGCAGTTTTTCTATCGGCCAATGGCTTCTTCTTCTGTGCTGCTCGGAG   840
Hexaploid_wheat                  CACCCATCCATCAGCAGTTTTTCTATCGGCCAATGGCTTCTTCTTCTGTGCTGCTCGGAG   840

Aegilops_tauschii_Coss_As_80     CCTCGGCCACGGCCGCGCTCACCGGTACCCCGGCAGGCAAGGCCCTTCCCCGGCCTTGCT   899
Aegilops_tauschii_Coss_As_77     CCTCGGCCACGGCCGCGCTCACCGGTACCCCGGCAGGCAAGGCCCTTCCCCGGCCTTGCT   899
Aegilops_tauschii_Coss_As_2392   CCTCGGCCACGGCCGCGCTCACCGGTACCCCGGCAGGCAAGGCCCTTCCCCGGCCTTGCT   899
Aegilops_tauschii_Coss_As_2386   CCTCGGCCACGGCCGCGCTCACCGGTACCCCGGCAGGCAAGGCCCTTCCCCGGCCTTGCT   900
Aegilops_tauschii_Coss_As_2387   CCTCGGCCACGGCCGCGCTCACCGGTACCCCGGCAGGCAAGGCCCTTCCCCGGCCTTGCT   900
Aegilops_tauschii_Coss_As_2388   CCTCGGCCACGGCCGCGCTCACCGGTACCCCGGCAGGCAAGGCCCTTCCCCGGCCTTGCT   900
Hexaploid_wheat                  CCTCGGCCACGGCCGCGCTCACCGGTACCCCGGCAGGCAAGGCCCTTCCCCGGCCTTGCT   900

Aegilops_tauschii_Coss_As_80     TCCTCGCCGCTCGCCCGCGCACCGTGAGCGGTGGCCGCCTCTGCCTGCAGAACGCTCCAA   959
Aegilops_tauschii_Coss_As_77     TCCTCGCCGCTCGCCCGCGCACCGTGAGCGGTGGCCGCCTCTGCCTGCAGAACGCTCCAA   959
Aegilops_tauschii_Coss_As_2392   TCCTCGCCGCTCGCCCGCGCACCGTGAGCGGTGGCCGCCTCTGCCTGCAGAACGCTCCAA   959
Aegilops_tauschii_Coss_As_2386   TCCTCGCCGCTCGCCCGCGCACCGTGAGCGGTGGCCGCCTCTGCCTGCAGAACGCTCCAA   960
Aegilops_tauschii_Coss_As_2387   TCCTCGCCGCTCGCCCGCGCACCGTGAGCGGTGGCCGCCTCTGCCTGCAGAACGCTCCAA   960
Aegilops_tauschii_Coss_As_2388   TCCTCGCCGCTCGCCCGCGCACCGTGAGCGGTGGCCGCCTCTGCCTGCAGAACGCTCCAA   960
Hexaploid_wheat                  TCCTCGCCGCTCGCCCGCGCACCGTGAGCGGTGGCCGCCTCTGCCTGCAGAACGCTCCAA   960

Aegilops_tauschii_Coss_As_80     GGGCGACTCCGGTACGTGTGTCCCGAGCAGTACCGTTCGATCATGACTTCATGCCGATTA   1019
Aegilops_tauschii_Coss_As_77     GGGCGACTCCGGTACGTGTGTCCCGAGCAGTACCGTTCGATCATGACTTCATGCCGATTA   1019
Aegilops_tauschii_Coss_As_2392   GGGCGACTCCGGTACGTGTGTCCCGAGCAGTACCGTTCGATCATGACTTCATGCCGATTA   1019
Aegilops_tauschii_Coss_As_2386   GGGCGACTCCGGTACGTGTGTCCCGAGCAGTACCGTTCGATCATGACTTCATGCCGATTA   1020
Aegilops_tauschii_Coss_As_2387   GGGCGACTCCGGTACGTGTGTCCCGAGCAGTACCGTTCGATCATGACTTCATGCCGATTA   1020
Aegilops_tauschii_Coss_As_2388   GGGCGACTCCGGTACGTGTGTCCCGAGCAGTACCGTTCGATCATGACTTCATGCCGATTA   1020
Hexaploid_wheat                  GGGCGACTCCGGTACGTGTGTCCCGAGCAGTACCGTTCGATCATGACTTCATGCCGATTA   1020

Aegilops_tauschii_Coss_As_80     TTTTACGTACGCTGCTGGTTAATCAATCGTTCTATGGGATACGCTGACCGAGATATATGC   1079
Aegilops_tauschii_Coss_As_77     TTTTACGTACGCTGCTGGTTAATCAATCGTTCTATGGGATACGCTGACCGAGATATATGC   1079
Aegilops_tauschii_Coss_As_2392   TTTTACGTACGCTGCTGGTTAATCAATCGTTCTATGGGATACGCTGACCGAGATATATGC   1079
Aegilops_tauschii_Coss_As_2386   TTTTACGTACGCTGCTGGTTAATCAATCGTTCTATGGGATACGCTGACCGAGATATATGC   1080
Aegilops_tauschii_Coss_As_2387   TTTTACGTACGCTGCTGGTTAATCAATCGTTCTATGGGATACGCTGACCGAGATATATGC   1080
Aegilops_tauschii_Coss_As_2388   TTTTACGTACGCTGCTGGTTAATCAATCGTTCTATGGGATACGCTGACCGAGATATATGC   1080
Hexaploid_wheat                  TTTTACGTACGCTGCTGGTTAATCAATCGTTCTATGGGATACGCTGACCGAGATATATGC   1080

Aegilops_tauschii_Coss_As_80     GTGCATGGGACAGGCGTACAACGACGCTGCGGATGCCACCGACAAGGCCATCGAGGGCGT   1139
Aegilops_tauschii_Coss_As_77     GTGCATGGGACAGGCGTACAACGACGCTGCGGATGCCACCGACAAGGCCATCGAGGGCGT   1139
Aegilops_tauschii_Coss_As_2392   GTGCATGGGACAGGCGTACAACGACGCTGCGGATGCCACCGACAAGGCCATCGAGGGCGT   1139
Aegilops_tauschii_Coss_As_2386   GTGCATGGGACAGGCGTACAACGACGCTGCGGATGCCACCGACAAGGCCATCGAGGGCGT   1140
Aegilops_tauschii_Coss_As_2387   GTGCATGGGACAGGCGTACAACGACGCTGCGGATGCCACCGACAAGGCCATCGAGGGCGT   1140
Aegilops_tauschii_Coss_As_2388   GTGCATGGGACAGGCGTACAACGACGCTGCGGATGCCACCGACAAGGCCATCGAGGGCGT   1140
Hexaploid_wheat                  GTGCATGGGACAGGCGTACAACGACGCTGCGGATGCCACCGACAAGGCCATCGAGGGCGT   1140

Aegilops_tauschii_Coss_As_80     GAAGGGGGTGGCCGACGAGCTGAAGAAGGGCGTGGCGGAGGCGGCGGAGGCCGTCTCGGG   1199
Aegilops_tauschii_Coss_As_77     GAAGGGGGTGGCCGACGAGCTGAAGAAGGGCGTGGCGGAGGCGGCGGAGGCCGTCTCGGG   1199
Aegilops_tauschii_Coss_As_2392   GAAGGGGGTGGCCGACGAGCTGAAGAAGGGCGTGGCGGAGGCGGCGGAGGCCGTCTCGGG   1199
Aegilops_tauschii_Coss_As_2386   GAAGGGGGTGGCCGACGAGCTGAAGAAGGGCGTGGCGGAGGCGGCGGAGGCCGTCTCGGG   1200
Aegilops_tauschii_Coss_As_2387   GAAGGGGGTGGCCGACGAGCTGAAGAAGGGCGTGGCGGAGGCGGCGGAGGCCGTCTCGGG   1200
Aegilops_tauschii_Coss_As_2388   GAAGGGGGTGGCCGACGAGCTGAAGAAGGGCGTGGCGGAGGCGGCGGAGGCCGTCTCGGG   1200
Hexaploid_wheat                  GAAGGGGGTGGCCGACGAGCTGAAGAAGGGCGTGGCGGAGGCGGCGGAGGCCGTCTCGGG   1200
```

```
Aegilops_tauschii_Coss_As_80     CAACACCGAGAAGGCCGCGGAGGAAGCCGGCAAGGGCGCGAGCGAGGTGGACGACAAGGC  1259
Aegilops_tauschii_Coss_As_77     CAACACCGAGAAGGCCGCGGAGGAAGCCGGCAAGGGCGCGAGCGAGGTGGACGACAAGGC  1259
Aegilops_tauschii_Coss_As_2392   CAACACCGAGAAGGCCGCGGAGGAAGCCGGCAAGGGCGCGAGCGAGGTGGACGACAAGGC  1259
Aegilops_tauschii_Coss_As_2386   CAACACCGAGAAGGCCGCGGAGGAAGCCGGCAAGGGCGCGAGCGAGGTGGACGACAAGGC  1260
Aegilops_tauschii_Coss_As_2387   CAACACCGAGAAGGCCGCGGAGGAAGCCGGCAAGGGCGCGAGCGAGGTGGACGACAAGGC  1260
Aegilops_tauschii_Coss_As_2388   CAACACCGAGAAGGCCGCGGAGGAAGCCGGCAAGGGCGCGAGCGAGGTGGACGACAAGGC  1260
Hexaploid_wheat                  CAACACCGAGAAGGCCGCGGAGGAAGCCGGCAAGGGCGCGAGCGAGGTGGACGACAAGGC  1260

Aegilops_tauschii_Coss_As_80     CAAGGACTTCGGCGAGCAGGCGAAGAAGGCGACGGAGGAGGCGTGGGACGGCGCCAAGGA  1319
Aegilops_tauschii_Coss_As_77     CAAGGACTTCGGCGAGCAGGCGAAGAAGGCGACGGAGGAGGCGTGGGACGGCGCCAAGGA  1319
Aegilops_tauschii_Coss_As_2392   CAAGGACTTCGGCGAGCAGGCGAAGAAGGCGACGGAGGAGGCGTGGGACGGCGCCAAGGA  1319
Aegilops_tauschii_Coss_As_2386   CAAGGACTTCGGCGAGCAGGCGAAGAAGGCGACGGAGGAGGCGTGGGACGGCGCCAAGGA  1320
Aegilops_tauschii_Coss_As_2387   CAAGGACTTCGGCGAGCAGGCGAAGAAGGCGACGGAGGAGGCGTGGGACGGCGCCAAGGA  1320
Aegilops_tauschii_Coss_As_2388   CAAGGACTTCGGCGAGCAGGCGAAGAAGGCGACGGAGGAGGCGTGGGACGGCGCCAAGGA  1320
Hexaploid_wheat                  CAAGGACTTCGGCGAGCAGGCGAAGAAGGCGACGGAGGAGGCGTGGGACGGCGCCAAGGA  1320

Aegilops_tauschii_Coss_As_80     CGCCGCACAGGGCATCACGGACAAGGTCGCCGCCGCGGCCAAAAAGGAAGCTAGCTA     1376
Aegilops_tauschii_Coss_As_77     CGCCGCACAGGGCATCACGGACAAGGTCGCCGCCGCGGCCAAAAAGGAAGCTAGCTA     1376
Aegilops_tauschii_Coss_As_2392   CGCCGCACAGGGCATCACGGACAAGGTCGCCGCCGCGGCCAAAAAGGAAGCTAGCTA     1376
Aegilops_tauschii_Coss_As_2386   CGCCGCACAGGGCATCACGGACAAGGTCGCCGCCGCGGCCAAAAAGGAAGCTAGCTA     1377
Aegilops_tauschii_Coss_As_2387   CGCCGCACAGGGCATCACGGACAAGGTCGCCGCCGCGGCCAAAAAGGAAGCTAGCTA     1377
Aegilops_tauschii_Coss_As_2388   CGCCGCACAGGGCATCACGGACAAGGTCGCCGCCGCGGCCAAAAAGGAAGCTAGCTA     1377
Hexaploid_wheat                  CGCCGCACAGGGCATCACGGACAAGGTCGCCGCCGCGGCCAAAAAGGAAGCTAGCTA     1377
```

Supplementary Fig. S6 Sequence alignments of the *Wcor15-2D* from hexaploid wheat, tetraploid wheat, and diploid *Ae. tauschii* (As 80, As 77, As 2392, As 2386, As 2387 and As 2388).

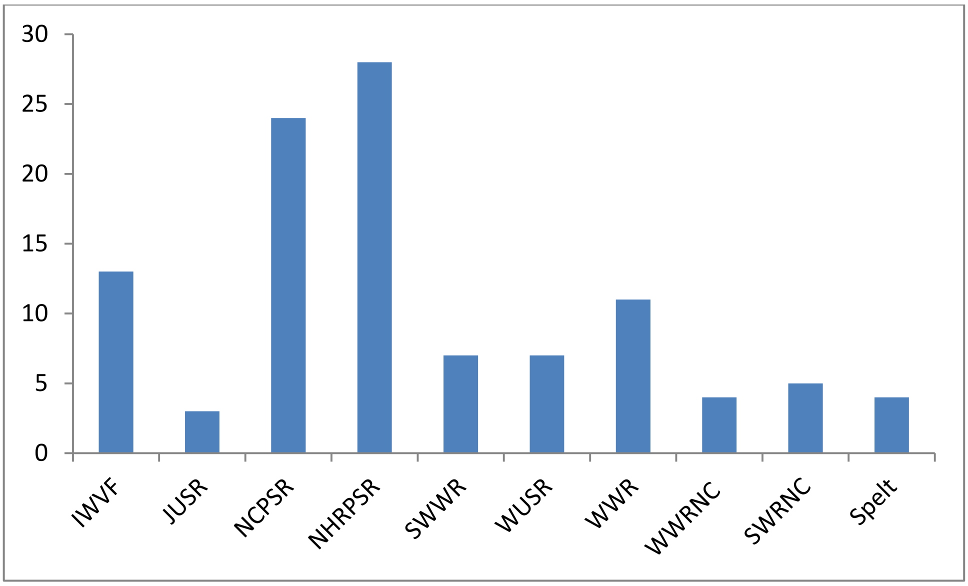

Supplementary Fig. S7 Distribution of hexaploid wheat with different geographical areas. 102 common wheats including 4 varieties from Winter wheat region of North China (WWRNC), 24 varieties from North China plain sub-region of Yellow & Huai river winter wheat region (NCPSR), 28 varieties from North Huai river plain sub-region of Yellow & Huai river winter wheat region (NHRPSR), 7 varieties from West upland sub-region of Yellow & Huai river winter wheat region (WUSR), 3 varietie from Jiaodong upland sub-region of Yellow & Huai river winter wheat region (JUSR), 11 varieties from Winter wheat region of middle and lower reaches of the Yangtze river (WWR), 7 varieties from Southwestern winter wheat region (SWWR), 5 varieties from Spring wheat region of North China (SWRNC),13 varieties from Introduced wheat variety of foreign (IWVF) and 4 spelt wheats.

```
WWRNC    CCTTCTCATCCATCATAGCAGCTGACATCTTTTGAAGGCTCTCCTTGAGAGCAAATATAA   60
NCPSR    CCTTCTCATCCATCATAGCAGCTGACATCTTTTGAAGGCTCTCCTTGAGAGCAAATATAA   60
NHRPSR   CCTTCTCATCCATCATAGCAGCTGACATCTTTTGAAGGCTCTCCTTGAGAGCAAATATAA   60
WUSR     CCTTCTCATCCATCATAGCAGCTGACATCTTTTGAAGGCTCTCCTTGAGAGCAAATATAA   60
JUSR     CCTTCTCATCCATCATAGCAGCTGACATCTTTTGAAGGCTCTCCTTGAGAGCAAATATAA   60
WWR      CCTTCTCATCCATCATAGCAGCTGACATCTTTTGAAGGCTCTCCTTGAGAGCAAATATAA   60
SWWR     CCTTCTCATCCATCATAGCAGCTGACATCTTTTGAAGGCTCTCCTTGAGAGCAAATATAA   60
SWRNC    CCTTCTCATCCATCATAGCAGCTGACATCTTTTGAAGGCTCTCCTTGAGAGCAAATATAA   60
IWVF     CCTTCTCATCCATCATAGCAGCTGACATCTTTTGAAGGCTCTCCTTGAGAGCAAATATAA   60
spelt    CCTTCTCATCCATCATAGCAGCTGACATCTTTTGAAGGCTCTCCTTGAGAGCAAATATAA   60

WWRNC    TAAGTGATGTAAGCGAGATGTAAGACTTTCAATATAATATTTTTAATGAGTTGAAAGAGA   120
NCPSR    TAAGTGATGTAAGCGAGATGTAAGACTTTCAATATAATATTTTTAATGAGTTGAAAGAGA   120
NHRPSR   TAAGTGATGTAAGCGAGATGTAAGACTTTCAATATAATATTTTTAATGAGTTGAAAGAGA   120
WUSR     TAAGTGATGTAAGCGAGATGTAAGACTTTCAATATAATATTTTTAATGAGTTGAAAGAGA   120
JUSR     TAAGTGATGTAAGCGAGATGTAAGACTTTCAATATAATATTTTTAATGAGTTGAAAGAGA   120
WWR      TAAGTGATGTAAGCGAGATGTAAGACTTTCAATATAATATTTTTAATGAGTTGAAAGAGA   120
SWWR     TAAGTGATGTAAGCGAGATGTAAGACTTTCAATATAATATTTTTAATGAGTTGAAAGAGA   120
SWRNC    TAAGTGATGTAAGCGAGATGTAAGACTTTCAATATAATATTTTTAATGAGTTGAAAGAGA   120
IWVF     TAAGTGATGTAAGCGAGATGTAAGACTTTCAATATAATATTTTTAATGAGTTGAAAGAGA   120
spelt    TAAGTGATGTAAGCGAGATGTAAGACTTTCAATATAATATTTTTAATGAGTTGAAAGAGA   120

WWRNC    GAGAAGATAAAAGAGAATAGAAGCAGGCTATAGATTAATAACCAGTTGCAGCATGTGCCC   180
NCPSR    GAGAAGATAAAAGAGAATAGAAGCAGGCTATAGATTAATAACCAGTTGCAGCATGTGCCC   180
NHRPSR   GAGAAGATAAAAGAGAATAGAAGCAGGCTATAGATTAATAACCAGTTGCAGCATGTGCCC   180
WUSR     GAGAAGATAAAAGAGAATAGAAGCAGGCTATAGATTAATAACCAGTTGCAGCATGTGCCC   180
JUSR     GAGAAGATAAAAGAGAATAGAAGCAGGCTATAGATTAATAACCAGTTGCAGCATGTGCCC   180
WWR      GAGAAGATAAAAGAGAATAGAAGCAGGCTATAGATTAATAACCAGTTGCAGCATGTGCCC   180
SWWR     GAGAAGATAAAAGAGAATAGAAGCAGGCTATAGATTAATAACCAGTTGCAGCATGTGCCC   180
SWRNC    GAGAAGATAAAAGAGAATAGAAGCAGGCTATAGATTAATAACCAGTTGCAGCATGTGCCC   180
IWVF     GAGAAGATAAAAGAGAATAGAAGCAGGCTATAGATTAATAACCAGTTGCAGCATGTGCCC   180
spelt    GAGAAGATAAAAGAGAATAGAAGCAGGCTATAGATTAATAACCAGTTGCAGCATGTGCCC   180

WWRNC    TAGTGGCTTTGTGAGAGAGTGAGGTGACTCATGTATCAACAAAGTAATACTCCCTCCTTC   240
NCPSR    TAGTGGCTTTGTGAGAGAGTGAGGTGACTCATGTATCAACAAAGTAATACTCCCTCCTTC   240
NHRPSR   TAGTGGCTTTGTGAGAGAGTGAGGTGACTCATGTATCAACAAAGTAATACTCCCTCCTTC   240
WUSR     TAGTGGCTTTGTGAGAGAGTGAGGTGACTCATGTATCAACAAAGTAATACTCCCTCCTTC   240
JUSR     TAGTGGCTTTGTGAGAGAGTGAGGTGACTCATGTATCAACAAAGTAATACTCCCTCCTTC   240
WWR      TAGTGGCTTTGTGAGAGAGTGAGGTGACTCATGTATCAACAAAGTAATACTCCCTCCTTC   240
SWWR     TAGTGGCTTTGTGAGAGAGTGAGGTGACTCATGTATCAACAAAGTAATACTCCCTCCTTC   240
SWRNC    TAGTGGCTTTGTGAGAGAGTGAGGTGACTCATGTATCAACAAAGTAATACTCCCTCCTTC   240
IWVF     TAGTGGCTTTGTGAGAGAGTGAGGTGACTCATGTATCAACAAAGTAATACTCCCTCCTTC   240
spelt    TAGTGGCTTTGTGAGAGAGTGAGGTGACTCATGTATCAACAAAGTAATACTCCCTCCTTC   240

WWRNC    TAGGAATACTTGTCGGAGAAACGGATAAAAATGGATGTATGTAGAACTAAAATACGTCTA   300
NCPSR    TAGGAATACTTGTCGGAGAAACGGATAAAAATGGATGTATGTAGAACTAAAATACGTCTA   300
NHRPSR   TAGGAATACTTGTCGGAGAAACGGATAAAAATGGATGTATGTAGAACTAAAATACGTCTA   300
WUSR     TAGGAATACTTGTCGGAGAAACGGATAAAAATGGATGTATGTAGAACTAAAATACGTCTA   300
JUSR     TAGGAATACTTGTCGGAGAAACGGATAAAAATGGATGTATGTAGAACTAAAATACGTCTA   300
WWR      TAGGAATACTTGTCGGAGAAACGGATAAAAATGGATGTATGTAGAACTAAAATACGTCTA   300
SWWR     TAGGAATACTTGTCGGAGAAACGGATAAAAATGGATGTATGTAGAACTAAAATACGTCTA   300
SWRNC    TAGGAATACTTGTCGGAGAAACGGATAAAAATGGATGTATGTAGAACTAAAATACGTCTA   300
IWVF     TAGGAATACTTGTCGGAGAAACGGATAAAAATGGATGTATGTAGAACTAAAATACGTCTA   300
spelt    TAGGAATACTTGTCGGAGAAACGGATAAAAATGGATGTATGTAGAACTAAAATACGTCTA   300

WWRNC    AATACATTCATTTTCCGACAAATATTTTCAGATGAAGGGAGTACATATCTATATCTAATT   360
NCPSR    AATACATTCATTTTCCGACAAATATTTTCAGATGAAGGGAGTACATATCTATATCTAATT   360
NHRPSR   AATACATTCATTTTCCGACAAATATTTTCAGATGAAGGGAGTACATATCTATATCTAATT   360
WUSR     AATACATTCATTTTCCGACAAATATTTTCAGATGAAGGGAGTACATATCTATATCTAATT   360
JUSR     AATACATTCATTTTCCGACAAATATTTTCAGATGAAGGGAGTACATATCTATATCTAATT   360
WWR      AATACATTCATTTTCCGACAAATATTTTCAGATGAAGGGAGTACATATCTATATCTAATT   360
SWWR     AATACATTCATTTTCCGACAAATATTTTCAGATGAAGGGAGTACATATCTATATCTAATT   360
SWRNC    AATACATTCATTTTCCGACAAATATTTTCAGATGAAGGGAGTACATATCTATATCTAATT   360
IWVF     AATACATTCATTTTCCGACAAATATTTTCAGATGAAGGGAGTACATATCTATATCTAATT   360
spelt    AATACATTCATTTTCCGACAAATATTTTCAGATGAAGGGAGTACATATCTATATCTAATT   360
```

```
WWRNC     ATTATATTTGCTGACTATAAACTTAAATATAAATGACATGGCAACATTATATAGCTATTA    420
NCPSR     ATTATATTTGCTGACTATAAACTTAAATATAAATGACATGGCAACATTATATAGCTATTA    420
NHRPSR    ATTATATTTGCTGACTATAAACTTAAATATAAATGACATGGCAACATTATATAGCTATTA    420
WUSR      ATTATATTTGCTGACTATAAACTTAAATATAAATGACATGGCAACATTATATAGCTATTA    420
JUSR      ATTATATTTGCTGACTATAAACTTAAATATAAATGACATGGCAACATTATATAGCTATTA    420
WWR       ATTATATTTGCTGACTATAAACTTAAATATAAATGACATGGCAACATTATATAGCTATTA    420
SWWR      ATTATATTTGCTGACTATAAACTTAAATATAAATGACATGGCAACATTATATAGCTATTA    420
SWRNC     ATTATATTTGCTGACTATAAACTTAAATATAAATGACATGGCAACATTATATAGCTATTA    420
IWVF      ATTATATTTGCTGACTATAAACTTAAATATAAATGACATGGCAACATTATATAGCTATTA    420
spelt     ATTATATTTGCTGACTATAAACTTAAATATAAATGACATGGCAACATTATATAGCTATTA    420

WWRNC     ACCATGCTCCCGACCATGTATCGGTGATGTCATTATTGTTCATTCAATCACTTTCCAATT    480
NCPSR     ACCATGCTCCCGACCATGTATCGGTGATGTCATTATTGTTCATTCAATCACTTTCCAATT    480
NHRPSR    ACCATGCTCCCGACCATGTATCGGTGATGTCATTATTGTTCATTCAATCACTTTCCAATT    480
WUSR      ACCATGCTCCCGACCATGTATCGGTGATGTCATTATTGTTCATTCAATCACTTTCCAATT    480
JUSR      ACCATGCTCCCGACCATGTATCGGTGATGTCATTATTGTTCATTCAATCACTTTCCAATT    480
WWR       ACCATGCTCCCGACCATGTATCGGTGATGTCATTATTGTTCATTCAATCACTTTCCAATT    480
SWWR      ACCATGCTCCCGACCATGTATCGGTGATGTCATTATTGTTCATTCAATCACTTTCCAATT    480
SWRNC     ACCATGCTCCCGACCATGTATCGGTGATGTCATTATTGTTCATTCAATCACTTTCCAATT    480
IWVF      ACCATGCTCCCGACCATGTATCGGTGATGTCATTATTGTTCATTCAATCACTTTCCAATT    480
spelt     ACCATGCTCCCGACCATGTATCGGTGATGTCATTATTGTTCATTCAATCACTTTCCAATT    480

WWRNC     AATACTTTCTACAGATGGGATATAAATATGGCTAAACTTACCCTTGATTAATCGACGGAC    540
NCPSR     AATACTTTCTACAGATGGGATATAAATATGGCTAAACTTACCCTTGATTAATCGACGGAC    540
NHRPSR    AATACTTTCTACAGATGGGATATAAATATGGCTAAACTTACCCTTGATTAATCGACGGAC    540
WUSR      AATACTTTCTACAGATGGGATATAAATATGGCTAAACTTACCCTTGATTAATCGACGGAC    540
JUSR      AATACTTTCTACAGATGGGATATAAATATGGCTAAACTTACCCTTGATTAATCGACGGAC    540
WWR       AATACTTTCTACAGATGGGATATAAATATGGCTAAACTTACCCTTGATTAATCGACGGAC    540
SWWR      AATACTTTCTACAGATGGGATATAAATATGGCTAAACTTACCCTTGATTAATCGACGGAC    540
SWRNC     AATACTTTCTACAGATGGGATATAAATATGGCTAAACTTACCCTTGATTAATCGACGGAC    540
IWVF      AATACTTTCTACAGATGGGATATAAATATGGCTAAACTTACCCTTGATTAATCGACGGAC    540
spelt     AATACTTTCTACAGATGGGATATAAATATGGCTAAACTTACCCTTGATTAATCGACGGAC    540

WWRNC     GGTATCAAATATCTTCTGCTATCATAAGTTGAAGCATCTTAAAAGAGCTCCTGACAAAAC    600
NCPSR     GGTATCAAATATCTTCTGCTATCATAAGTTGAAGCATCTTAAAAGAGCTCCTGACAAAAC    600
NHRPSR    GGTATCAAATATCTTCTGCTATCATAAGTTGAAGCATCTTAAAAGAGCTCCTGACAAAAC    600
WUSR      GGTATCAAATATCTTCTGCTATCATAAGTTGAAGCATCTTAAAAGAGCTCCTGACAAAAC    600
JUSR      GGTATCAAATATCTTCTGCTATCATAAGTTGAAGCATCTTAAAAGAGCTCCTGACAAAAC    600
WWR       GGTATCAAATATCTTCTGCTATCATAAGTTGAAGCATCTTAAAAGAGCTCCTGACAAAAC    600
SWWR      GGTATCAAATATCTTCTGCTATCATAAGTTGAAGCATCTTAAAAGAGCTCCTGACAAAAC    600
SWRNC     GGTATCAAATATCTTCTGCTATCATAAGTTGAAGCATCTTAAAAGAGCTCCTGACAAAAC    600
IWVF      GGTATCAAATATCTTCTGCTATCATAAGTTGAAGCATCTTAAAAGAGCTCCTGACAAAAC    600
spelt     GGTATCAAATATCTTCTGCTATCATAAGTTGAAGCATCTTAAAAGAGCTCCTGACAAAAC    600

WWRNC     CTTGGTTGTACGTAACCTTCAAACGTGGACAGTAGGATTTGTTTCTTCGATCCGCTCCAA    660
NCPSR     CTTGGTTGTACGTAACCTTCAAACGTGGACAGTAGGATTTGTTTCTTCGATCCGCTCCAA    660
NHRPSR    CTTGGTTGTACGTAACCTTCAAACGTGGACAGTAGGATTTGTTTCTTCGATCCGCTCCAA    660
WUSR      CTTGGTTGTACGTAACCTTCAAACGTGGACAGTAGGATTTGTTTCTTCGATCCGCTCCAA    660
JUSR      CTTGGTTGTACGTAACCTTCAAACGTGGACAGTAGGATTTGTTTCTTCGATCCGCTCCAA    660
WWR       CTTGGTTGTACGTAACCTTCAAACGTGGACAGTAGGATTTGTTTCTTCGATCCGCTCCAA    660
SWWR      CTTGGTTGTACGTAACCTTCAAACGTGGACAGTAGGATTTGTTTCTTCGATCCGCTCCAA    660
SWRNC     CTTGGTTGTACGTAACCTTCAAACGTGGACAGTAGGATTTGTTTCTTCGATCCGCTCCAA    660
IWVF      CTTGGTTGTACGTAACCTTCAAACGTGGACAGTAGGATTTGTTTCTTCGATCCGCTCCAA    660
spelt     CTTGGTTGTACGTAACCTTCAAACGTGGACAGTAGGATTTGTTTCTTCGATCCGCTCCAA    660

WWRNC     ACAGGTCATGTAGGTATACGTGCCTTCGTACGAGACGCTGGTGGGGTAAGCAGCTACCCA    720
NCPSR     ACAGGTCATGTAGGTATACGTGCCTTCGTACGAGACGCTGGTGGGGTAAGCAGCTACCCA    720
NHRPSR    ACAGGTCATGTAGGTATACGTGCCTTCGTACGAGACGCTGGTGGGGTAAGCAGCTACCCA    720
WUSR      ACAGGTCATGTAGGTATACGTGCCTTCGTACGAGACGCTGGTGGGGTAAGCAGCTACCCA    720
JUSR      ACAGGTCATGTAGGTATACGTGCCTTCGTACGAGACGCTGGTGGGGTAAGCAGCTACCCA    720
WWR       ACAGGTCATGTAGGTATACGTGCCTTCGTACGAGACGCTGGTGGGGTAAGCAGCTACCCA    720
SWWR      ACAGGTCATGTAGGTATACGTGCCTTCGTACGAGACGCTGGTGGGGTAAGCAGCTACCCA    720
SWRNC     ACAGGTCATGTAGGTATACGTGCCTTCGTACGAGACGCTGGTGGGGTAAGCAGCTACCCA    720
IWVF      ACAGGTCATGTAGGTATACGTGCCTTCGTACGAGACGCTGGTGGGGTAAGCAGCTACCCA    720
spelt     ACAGGTCATGTAGGTATACGTGCCTTCGTACGAGACGCTGGTGGGGTAAGCAGCTACCCA    720
```

```
WWRNC     TTTCTTTTCTTTTATTGGTATAGGAAATTCGACCGTACATTACCACATAAACCTTGTCGA   780
NCPSR     TTTCTTTTCTTTTATTGGTATAGGAAATTCGACCGTACATTACCACATAAACCTTGTCGA   780
NHRPSR    TTTCTTTTCTTTTATTGGTATAGGAAATTCGACCGTACATTACCACATAAACCTTGTCGA   780
WUSR      TTTCTTTTCTTTTATTGGTATAGGAAATTCGACCGTACATTACCACATAAACCTTGTCGA   780
JUSR      TTTCTTTTCTTTTATTGGTATAGGAAATTCGACCGTACATTACCACATAAACCTTGTCGA   780
WWR       TTTCTTTTCTTTTATTGGTATAGGAAATTCGACCGTACATTACCACATAAACCTTGTCGA   780
SWWR      TTTCTTTTCTTTTATTGGTATAGGAAATTCGACCGTACATTACCACATAAACCTTGTCGA   780
SWRNC     TTTCTTTTCTTTTATTGGTATAGGAAATTCGACCGTACATTACCACATAAACCTTGTCGA   780
IWVF      TTTCTTTTCTTTTATTGGTATAGGAAATTCGACCGTACATTACCACATAAACCTTGTCGA   780
spelt     TTTCTTTTCTTTTATTGGTATAGGAAATTCGACCGTACATTACCACATAAACCTTGTCGA   780

WWRNC     CAACTCTCCGCGCACTGCTCCACCACAGAACCTTGTCGACCGTCGATTGTCGCCATGCGC   840
NCPSR     CAACTCTCCGCGCACTGCTCCACCACAGAACCTTGTCGACCGTCGATTGTCGCCATGCGC   840
NHRPSR    CAACTCTCCGCGCACTGCTCCACCACAGAACCTTGTCGACCGTCGATTGTCGCCATGCGC   840
WUSR      CAACTCTCCGCGCACTGCTCCACCACAGAACCTTGTCGACCGTCGATTGTCGCCATGCGC   840
JUSR      CAACTCTCCGCGCACTGCTCCACCACAGAACCTTGTCGACCGTCGATTGTCGCCATGCGC   840
WWR       CAACTCTCCGCGCACTGCTCCACCACAGAACCTTGTCGACCGTCGATTGTCGCCATGCGC   840
SWWR      CAACTCTCCGCGCACTGCTCCACCACAGAACCTTGTCGACCGTCGATTGTCGCCATGCGC   840
SWRNC     CAACTCTCCGCGCACTGCTCCACCACAGAACCTTGTCGACCGTCGATTGTCGCCATGCGC   840
IWVF      CAACTCTCCGCGCACTGCTCCACCACAGAACCTTGTCGACCGTCGATTGTCGCCATGCGC   840
spelt     CAACTCTCCGCGCACTGCTCCACCACAGAACCTTGTCGACCGTCGATTGTCGCCATGCGC   840

WWRNC     CGGAGCGATCGGCCCCCGACGTGTCACCCCCCAGCTCACCCTTCGCCCCTCCGCGTCCGG   900
NCPSR     CGGAGCGATCGGCCCCCGACGTGTCACCCCCCAGCTCACCCTTCGCCCCTCCGCGTCCGG   900
NHRPSR    CGGAGCGATCGGCCCCCGACGTGTCACCCCCCAGCTCACCCTTCGCCCCTCCGCGTCCGG   900
WUSR      CGGAGCGATCGGCCCCCGACGTGTCACCCCCCAGCTCACCCTTCGCCCCTCCGCGTCCGG   900
JUSR      CGGAGCGATCGGCCCCCGACGTGTCACCCCCCAGCTCACCCTTCGCCCCTCCGCGTCCGG   900
WWR       CGGAGCGATCGGCCCCCGACGTGTCACCCCCCAGCTCACCCTTCGCCCCTCCGCGTCCGG   900
SWWR      CGGAGCGATCGGCCCCCGACGTGTCACCCCCCAGCTCACCCTTCGCCCCTCCGCGTCCGG   900
SWRNC     CGGAGCGATCGGCCCCCGACGTGTCACCCCCCAGCTCACCCTTCGCCCCTCCGCGTCCGG   900
IWVF      CGGAGCGATCGGCCCCCGACGTGTCACCCCCCAGCTCACCCTTCGCCCCTCCGCGTCCGG   900
spelt     CGGAGCGATCGGCCCCCGACGTGTCACCCCCCAGCTCACCCTTCGCCCCTCCGCGTCCGG   900

WWRNC     CCTATAAAAACGACGATGCACCTCTCGTCCTCCCATTGCATTGCAAGTGAAGCTCACACA   960
NCPSR     CCTATAAAAACGACGATGCACCTCTCGTCCTCCCATTGCATTGCAAGTGAAGCTCACACA   960
NHRPSR    CCTATAAAAACGACGATGCACCTCTCGTCCTCCCATTGCATTGCAAGTGAAGCTCACACA   960
WUSR      CCTATAAAAACGACGATGCACCTCTCGTCCTCCCATTGCATTGCAAGTGAAGCTCACACA   960
JUSR      CCTATAAAAACGACGATGCACCTCTCGTCCTCCCATTGCATTGCAAGTGAAGCTCACACA   960
WWR       CCTATAAAAACGACGATGCACCTCTCGTCCTCCCATTGCATTGCAAGTGAAGCTCACACA   960
SWWR      CCTATAAAAACGACGATGCACCTCTCGTCCTCCCATTGCATTGCAAGTGAAGCTCACACA   960
SWRNC     CCTATAAAAACGACGATGCACCTCTCGTCCTCCCATTGCATTGCAAGTGAAGCTCACACA   960
IWVF      CCTATAAAAACGACGATGCACCTCTCGTCCTCCCATTGCATTGCAAGTGAAGCTCACACA   960
spelt     CCTATAAAAACGACGATGCACCTCTCGTCCTCCCATTGCATTGCAAGTGAAGCTCACACA   960

WWRNC     ACCTACCCTACCCTACCCACCCATCCATCAGCAGTTTTTCTATCGACCAATGGCTTCTTC  1020
NCPSR     ACCTACCCTACCCTACCCACCCATCCATCAGCAGTTTTTCTATCGACCAATGGCTTCTTC  1020
NHRPSR    ACCTACCCTACCCTACCCACCCATCCATCAGCAGTTTTTCTATCGACCAATGGCTTCTTC  1020
WUSR      ACCTACCCTACCCTACCCACCCATCCATCAGCAGTTTTTCTATCGACCAATGGCTTCTTC  1020
JUSR      ACCTACCCTACCCTACCCACCCATCCATCAGCAGTTTTTCTATCGACCAATGGCTTCTTC  1020
WWR       ACCTACCCTACCCTACCCACCCATCCATCAGCAGTTTTTCTATCGACCAATGGCTTCTTC  1020
SWWR      ACCTACCCTACCCTACCCACCCATCCATCAGCAGTTTTTCTATCGACCAATGGCTTCTTC  1020
SWRNC     ACCTACCCTACCCTACCCACCCATCCATCAGCAGTTTTTCTATCGACCAATGGCTTCTTC  1020
IWVF      ACCTACCCTACCCTACCCACCCATCCATCAGCAGTTTTTCTATCGACCAATGGCTTCTTC  1020
spelt     ACCTACCCTACCCTACCCACCCATCCATCAGCAGTTTTTCTATCGACCAATGGCTTCTTC  1020

WWRNC     TTCCGTGCTTCTCGGAGCCTCGGCCACGGCCGCGCTCACCGGCACCCCGGCAGGCAAGGC  1080
NCPSR     TTCCGTGCTTCTCGGAGCCTCGGCCACGGCCGCGCTCACCGGCACCCCGGCAGGCAAGGC  1080
NHRPSR    TTCCGTGCTTCTCGGAGCCTCGGCCACGGCCGCGCTCACCGGCACCCCGGCAGGCAAGGC  1080
WUSR      TTCCGTGCTTCTCGGAGCCTCGGCCACGGCCGCGCTCACCGGCACCCCGGCAGGCAAGGC  1080
JUSR      TTCCGTGCTTCTCGGAGCCTCGGCCACGGCCGCGCTCACCGGCACCCCGGCAGGCAAGGC  1080
WWR       TTCCGTGCTTCTCGGAGCCTCGGCCACGGCCGCGCTCACCGGCACCCCGGCAGGCAAGGC  1080
SWWR      TTCCGTGCTTCTCGGAGCCTCGGCCACGGCCGCGCTCACCGGCACCCCGGCAGGCAAGGC  1080
SWRNC     TTCCGTGCTTCTCGGAGCCTCGGCCACGGCCGCGCTCACCGGCACCCCGGCAGGCAAGGC  1080
IWVF      TTCCGTGCTTCTCGGAGCCTCGGCCACGGCCGCGCTCACCGGCACCCCGGCAGGCAAGGC  1080
spelt     TTCCGTGCTTCTCGGAGCCTCGGCCACGGCCGCGCTCACCGGCACCCCGGCAGGCAAGGC  1080
```

```
WWRNC     CCTTCCCCGGCCTTGCTTCCTCGCCGCTCGCCCGCGCACCGTGAGCGGCGGCCGCCTCTG  1140
NCPSR     CCTTCCCCGGCCTTGCTTCCTCGCCGCTCGCCCGCGCACCGTGAGCGGCGGCCGCCTCTG  1140
NHRPSR    CCTTCCCCGGCCTTGCTTCCTCGCCGCTCGCCCGCGCACCGTGAGCGGCGGCCGCCTCTG  1140
WUSR      CCTTCCCCGGCCTTGCTTCCTCGCCGCTCGCCCGCGCACCGTGAGCGGCGGCCGCCTCTG  1140
JUSR      CCTTCCCCGGCCTTGCTTCCTCGCCGCTCGCCCGCGCACCGTGAGCGGCGGCCGCCTCTG  1140
WWR       CCTTCCCCGGCCTTGCTTCCTCGCCGCTCGCCCGCGCACCGTGAGCGGCGGCCGCCTCTG  1140
SWWR      CCTTCCCCGGCCTTGCTTCCTCGCCGCTCGCCCGCGCACCGTGAGCGGCGGCCGCCTCTG  1140
SWRNC     CCTTCCCCGGCCTTGCTTCCTCGCCGCTCGCCCGCGCACCGTGAGCGGCGGCCGCCTCTG  1140
IWVF      CCTTCCCCGGCCTTGCTTCCTCGCCGCTCGCCCGCGCACCGTGAGCGGCGGCCGCCTCTG  1140
spelt     CCTTCCCCGGCCTTGCTTCCTCGCCGCTCGCCCGCGCACCGTGAGCGGCGGCCGCCTCTG  1140

WWRNC     CCTGCAGAACGCTCCAAGGGCGACTCCGGTACGTGTGCCGCGAGCAGTACCGTTCGATCA  1200
NCPSR     CCTGCAGAACGCTCCAAGGGCGACTCCGGTACGTGTGCCGCGAGCAGTACCGTTCGATCA  1200
NHRPSR    CCTGCAGAACGCTCCAAGGGCGACTCCGGTACGTGTGCCGCGAGCAGTACCGTTCGATCA  1200
WUSR      CCTGCAGAACGCTCCAAGGGCGACTCCGGTACGTGTGCCGCGAGCAGTACCGTTCGATCA  1200
JUSR      CCTGCAGAACGCTCCAAGGGCGACTCCGGTACGTGTGCCGCGAGCAGTACCGTTCGATCA  1200
WWR       CCTGCAGAACGCTCCAAGGGCGACTCCGGTACGTGTGCCGCGAGCAGTACCGTTCGATCA  1200
SWWR      CCTGCAGAACGCTCCAAGGGCGACTCCGGTACGTGTGCCGCGAGCAGTACCGTTCGATCA  1200
SWRNC     CCTGCAGAACGCTCCAAGGGCGACTCCGGTACGTGTGCCGCGAGCAGTACCGTTCGATCA  1200
IWVF      CCTGCAGAACGCTCCAAGGGCGACTCCGGTACGTGTGCCGCGAGCAGTACCGTTCGATCA  1200
spelt     CCTGCAGAACGCTCCAAGGGCGACTCCGGTACGTGTGCCGCGAGCAGTACCGTTCGATCA  1200

WWRNC     TGGCCTCATGCCGATTATTTTTACGTACGCTGCTGGTTAGTCGTCCTCTGAGATATGCTG  1260
NCPSR     TGGCCTCATGCCGATTATTTTTACGTACGCTGCTGGTTAGTCGTCCTCTGAGATATGCTG  1260
NHRPSR    TGGCCTCATGCCGATTATTTTTACGTACGCTGCTGGTTAGTCGTCCTCTGAGATATGCTG  1260
WUSR      TGGCCTCATGCCGATTATTTTTACGTACGCTGCTGGTTAGTCGTCCTCTGAGATATGCTG  1260
JUSR      TGGCCTCATGCCGATTATTTTTACGTACGCTGCTGGTTAGTCGTCCTCTGAGATATGCTG  1260
WWR       TGGCCTCATGCCGATTATTTTTACGTACGCTGCTGGTTAGTCGTCCTCTGAGATATGCTG  1260
SWWR      TGGCCTCATGCCGATTATTTTTACGTACGCTGCTGGTTAGTCGTCCTCTGAGATATGCTG  1260
SWRNC     TGGCCTCATGCCGATTATTTTTACGTACGCTGCTGGTTAGTCGTCCTCTGAGATATGCTG  1260
IWVF      TGGCCTCATGCCGATTATTTTTACGTACGCTGCTGGTTAGTCGTCCTCTGAGATATGCTG  1260
spelt     TGGCCTCATGCCGATTATTTTTACGTACGCTGCTGGTTAGTCGTCCTCTGAGATATGCTG  1260

WWRNC     ACCGAGATATATGGGTGCATGGGACAGGCGTACAACGACGCTGCGGATGCTACCGACAAG  1320
NCPSR     ACCGAGATATATGGGTGCATGGGACAGGCGTACAACGACGCTGCGGATGCTACCGACAAG  1320
NHRPSR    ACCGAGATATATGGGTGCATGGGACAGGCGTACAACGACGCTGCGGATGCTACCGACAAG  1320
WUSR      ACCGAGATATATGGGTGCATGGGACAGGCGTACAACGACGCTGCGGATGCTACCGACAAG  1320
JUSR      ACCGAGATATATGGGTGCATGGGACAGGCGTACAACGACGCTGCGGATGCTACCGACAAG  1320
WWR       ACCGAGATATATGGGTGCATGGGACAGGCGTACAACGACGCTGCGGATGCTACCGACAAG  1320
SWWR      ACCGAGATATATGGGTGCATGGGACAGGCGTACAACGACGCTGCGGATGCTACCGACAAG  1320
SWRNC     ACCGAGATATATGGGTGCATGGGACAGGCGTACAACGACGCTGCGGATGCTACCGACAAG  1320
IWVF      ACCGAGATATATGGGTGCATGGGACAGGCGTACAACGACGCTGCGGATGCTACCGACAAG  1320
spelt     ACCGAGATATATGGGTGCATGGGACAGGCGTACAACGACGCTGCGGATGCTACCGACAAG  1320

WWRNC     GCCATCGACGGCGTGAAGGGGGTGGCCGACGAGTTGAAGAAGGGCGTGGCGGAGGCGGCT  1380
NCPSR     GCCATCGACGGCGTGAAGGGGGTGGCCGACGAGTTGAAGAAGGGCGTGGCGGAGGCGGCT  1380
NHRPSR    GCCATCGACGGCGTGAAGGGGGTGGCCGACGAGTTGAAGAAGGGCGTGGCGGAGGCGGCT  1380
WUSR      GCCATCGACGGCGTGAAGGGGGTGGCCGACGAGTTGAAGAAGGGCGTGGCGGAGGCGGCT  1380
JUSR      GCCATCGACGGCGTGAAGGGGGTGGCCGACGAGTTGAAGAAGGGCGTGGCGGAGGCGGCT  1380
WWR       GCCATCGACGGCGTGAAGGGGGTGGCCGACGAGTTGAAGAAGGGCGTGGCGGAGGCGGCT  1380
SWWR      GCCATCGACGGCGTGAAGGGGGTGGCCGACGAGTTGAAGAAGGGCGTGGCGGAGGCGGCT  1380
SWRNC     GCCATCGACGGCGTGAAGGGGGTGGCCGACGAGTTGAAGAAGGGCGTGGCGGAGGCGGCT  1380
IWVF      GCCATCGACGGCGTGAAGGGGGTGGCCGACGAGTTGAAGAAGGGCGTGGCGGAGGCGGCT  1380
spelt     GCCATCGACGGCGTGAAGGGGGTGGCCGACGAGTTGAAGAAGGGCGTGGCGGAGGCGGCT  1380

WWRNC     GAGGCCGTCTCGGGCAACACCGAGAAGGCCGCGGAGGAAGCCGGCAAAGGCGCGAGCGAG  1440
NCPSR     GAGGCCGTCTCGGGCAACACCGAGAAGGCCGCGGAGGAAGCCGGCAAAGGCGCGAGCGAG  1440
NHRPSR    GAGGCCGTCTCGGGCAACACCGAGAAGGCCGCGGAGGAAGCCGGCAAAGGCGCGAGCGAG  1440
WUSR      GAGGCCGTCTCGGGCAACACCGAGAAGGCCGCGGAGGAAGCCGGCAAAGGCGCGAGCGAG  1440
JUSR      GAGGCCGTCTCGGGCAACACCGAGAAGGCCGCGGAGGAAGCCGGCAAAGGCGCGAGCGAG  1440
WWR       GAGGCCGTCTCGGGCAACACCGAGAAGGCCGCGGAGGAAGCCGGCAAAGGCGCGAGCGAG  1440
SWWR      GAGGCCGTCTCGGGCAACACCGAGAAGGCCGCGGAGGAAGCCGGCAAAGGCGCGAGCGAG  1440
SWRNC     GAGGCCGTCTCGGGCAACACCGAGAAGGCCGCGGAGGAAGCCGGCAAAGGCGCGAGCGAG  1440
IWVF      GAGGCCGTCTCGGGCAACACCGAGAAGGCCGCGGAGGAAGCCGGCAAAGGCGCGAGCGAG  1440
spelt     GAGGCCGTCTCGGGCAACACCGAGAAGGCCGCGGAGGAAGCCGGCAAAGGCGCGAGCGAG  1440
```

```
WWRNC    GTGGACGAGAAGGCCAAGGACTTCGGCGAGCAGGCGAAGAAGGCGACGGAGGAGGCGTGG    1500
NCPSR    GTGGACGAGAAGGCCAAGGACTTCGGCGAGCAGGCGAAGAAGGCGACGGAGGAGGCGTGG    1500
NHRPSR   GTGGACGAGAAGGCCAAGGACTTCGGCGAGCAGGCGAAGAAGGCGACGGAGGAGGCGTGG    1500
WUSR     GTGGACGAGAAGGCCAAGGACTTCGGCGAGCAGGCGAAGAAGGCGACGGAGGAGGCGTGG    1500
JUSR     GTGGACGAGAAGGCCAAGGACTTCGGCGAGCAGGCGAAGAAGGCGACGGAGGAGGCGTGG    1500
WWR      GTGGACGAGAAGGCCAAGGACTTCGGCGAGCAGGCGAAGAAGGCGACGGAGGAGGCGTGG    1500
SWWR     GTGGACGAGAAGGCCAAGGACTTCGGCGAGCAGGCGAAGAAGGCGACGGAGGAGGCGTGG    1500
SWRNC    GTGGACGAGAAGGCCAAGGACTTCGGCGAGCAGGCGAAGAAGGCGACGGAGGAGGCGTGG    1500
IWVF     GTGGACGAGAAGGCCAAGGACTTCGGCGAGCAGGCGAAGAAGGCGACGGAGGAGGCGTGG    1500
spelt    GTGGACGAGAAGGCCAAGGACTTCGGCGAGCAGGCGAAGAAGGCGACGGAGGAGGCGTGG    1500

WWRNC    GACGGCGCCAAGGACGCCGCACAGGGCATCACGGACAAGGTCGCGGACGCGGCCAAAAAG    1560
NCPSR    GACGGCGCCAAGGACGCCGCACAGGGCATCACGGACAAGGTCGCGGACGCGGCCAAAAAG    1560
NHRPSR   GACGGCGCCAAGGACGCCGCACAGGGCATCACGGACAAGGTCGCGGACGCGGCCAAAAAG    1560
WUSR     GACGGCGCCAAGGACGCCGCACAGGGCATCACGGACAAGGTCGCGGACGCGGCCAAAAAG    1560
JUSR     GACGGCGCCAAGGACGCCGCACAGGGCATCACGGACAAGGTCGCGGACGCGGCCAAAAAG    1560
WWR      GACGGCGCCAAGGACGCCGCACAGGGCATCACGGACAAGGTCGCGGACGCGGCCAAAAAG    1560
SWWR     GACGGCGCCAAGGACGCCGCACAGGGCATCACGGACAAGGTCGCGGACGCGGCCAAAAAG    1560
SWRNC    GACGGCGCCAAGGACGCCGCACAGGGCATCACGGACAAGGTCGCGGACGCGGCCAAAAAG    1560
IWVF     GACGGCGCCAAGGACGCCGCACAGGGCATCACGGACAAGGTCGCGGACGCGGCCAAAAAG    1560
spelt    GACGGCGCCAAGGACGCCGCACAGGGCATCACGGACAAGGTCGCGGACGCGGCCAAAAAG    1560

WWRNC    GAAGCTAACTAAGCTAACAATGCGTTGACTAGTCCGATATGTATCTCTCAATTCATTTTC    1620
NCPSR    GAAGCTAACTAAGCTAACAATGCGTTGACTAGTCCGATATGTATCTCTCAATTCATTTTC    1620
NHRPSR   GAAGCTAACTAAGCTAACAATGCGTTGACTAGTCCGATATGTATCTCTCAATTCATTTTC    1620
WUSR     GAAGCTAACTAAGCTAACAATGCGTTGACTAGTCCGATATGTATCTCTCAATTCATTTTC    1620
JUSR     GAAGCTAACTAAGCTAACAATGCGTTGACTAGTCCGATATGTATCTCTCAATTCATTTTC    1620
WWR      GAAGCTAACTAAGCTAACAATGCGTTGACTAGTCCGATATGTATCTCTCAATTCATTTTC    1620
SWWR     GAAGCTAACTAAGCTAACAATGCGTTGACTAGTCCGATATGTATCTCTCAATTCATTTTC    1620
SWRNC    GAAGCTAACTAAGCTAACAATGCGTTGACTAGTCCGATATGTATCTCTCAATTCATTTTC    1620
IWVF     GAAGCTAACTAAGCTAACAATGCGTTGACTAGTCCGATATGTATCTCTCAATTCATTTTC    1620
spelt    GAAGCTAACTAAGCTAACAATGCGTTGACTAGTCCGATATGTATCTCTCAATTCATTTTC    1620

WWRNC    CATTGTAAGAAGGGTTGTAAGAATGCATATACGTACTTTGGTACAAGAAGAGATAAAATA    1680
NCPSR    CATTGTAAGAAGGGTTGTAAGAATGCATATACGTACTTTGGTACAAGAAGAGATAAAATA    1680
NHRPSR   CATTGTAAGAAGGGTTGTAAGAATGCATATACGTACTTTGGTACAAGAAGAGATAAAATA    1680
WUSR     CATTGTAAGAAGGGTTGTAAGAATGCATATACGTACTTTGGTACAAGAAGAGATAAAATA    1680
JUSR     CATTGTAAGAAGGGTTGTAAGAATGCATATACGTACTTTGGTACAAGAAGAGATAAAATA    1680
WWR      CATTGTAAGAAGGGTTGTAAGAATGCATATACGTACTTTGGTACAAGAAGAGATAAAATA    1680
SWWR     CATTGTAAGAAGGGTTGTAAGAATGCATATACGTACTTTGGTACAAGAAGAGATAAAATA    1680
SWRNC    CATTGTAAGAAGGGTTGTAAGAATGCATATACGTACTTTGGTACAAGAAGAGATAAAATA    1680
IWVF     CATTGTAAGAAGGGTTGTAAGAATGCATATACGTACTTTGGTACAAGAAGAGATAAAATA    1680
spelt    CATTGTAAGAAGGGTTGTAAGAATGCATATACGTACTTTGGTACAAGAAGAGATAAAATA    1680

WWRNC    GCTGCATTTATTTTCTGACATACAGGATTACCGCCCTGTTAATGTCAAACGCAATAAAGA    1740
NCPSR    GCTGCATTTATTTTCTGACATACAGGATTACCGCCCTGTTAATGTCAAACGCAATAAAGA    1740
NHRPSR   GCTGCATTTATTTTCTGACATACAGGATTACCGCCCTGTTAATGTCAAACGCAATAAAGA    1740
WUSR     GCTGCATTTATTTTCTGACATACAGGATTACCGCCCTGTTAATGTCAAACGCAATAAAGA    1740
JUSR     GCTGCATTTATTTTCTGACATACAGGATTACCGCCCTGTTAATGTCAAACGCAATAAAGA    1740
WWR      GCTGCATTTATTTTCTGACATACAGGATTACCGCCCTGTTAATGTCAAACGCAATAAAGA    1740
SWWR     GCTGCATTTATTTTCTGACATACAGGATTACCGCCCTGTTAATGTCAAACGCAATAAAGA    1740
SWRNC    GCTGCATTTATTTTCTGACATACAGGATTACCGCCCTGTTAATGTCAAACGCAATAAAGA    1740
IWVF     GCTGCATTTATTTTCTGACATACAGGATTACCGCCCTGTTAATGTCAAACGCAATAAAGA    1740
spelt    GCTGCATTTATTTTCTGACATACAGGATTACCGCCCTGTTAATGTCAAACGCAATAAAGA    1740

WWRNC    AAATGATGCTAAAAGGTTTACATAATAGCTATGCGTGAGTTGCTTGAATTTCGAATTCGA    1800
NCPSR    AAATGATGCTAAAAGGTTTACATAATAGCTATGCGTGAGTTGCTTGAATTTCGAATTCGA    1800
NHRPSR   AAATGATGCTAAAAGGTTTACATAATAGCTATGCGTGAGTTGCTTGAATTTCGAATTCGA    1800
WUSR     AAATGATGCTAAAAGGTTTACATAATAGCTATGCGTGAGTTGCTTGAATTTCGAATTCGA    1800
JUSR     AAATGATGCTAAAAGGTTTACATAATAGCTATGCGTGAGTTGCTTGAATTTCGAATTCGA    1800
WWR      AAATGATGCTAAAAGGTTTACATAATAGCTATGCGTGAGTTGCTTGAATTTCGAATTCGA    1800
SWWR     AAATGATGCTAAAAGGTTTACATAATAGCTATGCGTGAGTTGCTTGAATTTCGAATTCGA    1800
SWRNC    AAATGATGCTAAAAGGTTTACATAATAGCTATGCGTGAGTTGCTTGAATTTCGAATTCGA    1800
IWVF     AAATGATGCTAAAAGGTTTACATAATAGCTATGCGTGAGTTGCTTGAATTTCGAATTCGA    1800
spelt    AAATGATGCTAAAAGGTTTACATAATAGCTATGCGTGAGTTGCTTGAATTTCGAATTCGA    1800
```

```
WWRNC     GTCGGTCGATTCGTGGAAGAAGGAAGGAGGAGACGAGGTGT     1841
NCPSR     GTCGGTCGATTCGTGGAAGAAGGAAGGAGGAGACGAGGTGT     1841
NHRPSR    GTCGGTCGATTCGTGGAAGAAGGAAGGAGGAGACGAGGTGT     1841
WUSR      GTCGGTCGATTCGTGGAAGAAGGAAGGAGGAGACGAGGTGT     1841
JUSR      GTCGGTCGATTCGTGGAAGAAGGAAGGAGGAGACGAGGTGT     1841
WWR       GTCGGTCGATTCGTGGAAGAAGGAAGGAGGAGACGAGGTGT     1841
SWWR      GTCGGTCGATTCGTGGAAGAAGGAAGGAGGAGACGAGGTGT     1841
SWRNC     GTCGGTCGATTCGTGGAAGAAGGAAGGAGGAGACGAGGTGT     1841
IWVF      GTCGGTCGATTCGTGGAAGAAGGAAGGAGGAGACGAGGTGT     1841
spelt     GTCGGTCGATTCGTGGAAGAAGGAAGGAGGAGACGAGGTGT     1841
```

Supplementary Fig. S8 Sequence alignments of the *Wcor15-2A* from hexaploid wheat with different geographical areas. Winter wheat region of North China (WWRNC), North China plain sub-region of Yellow & Huai river winter wheat region (NCPSR), North Huai river plain sub-region of Yellow & Huai river winter wheat region (NHRPSR), West upland sub-region of Yellow & Huai river winter wheat region (WUSR), Jiaodong upland sub-region of Yellow & Huai river winter wheat region (JUSR), Winter wheat region of middle and lower reaches of the Yangtze river (WWR), Southwestern winter wheat region (SWWR), Spring wheat region of North China (SWRNC), Introduced wheat variety of foreign (IWVF) and spelt wheat.

```
IWVF-1       ...........................ATATTATATTTTTGCTGAGTTGAAGGTGATAGG    33
IWVF-2       ...........................ATATTATATTTTTGCTGAGTTGAAGGTGATAGG    33
IWVF-3       ...........................ATATTATATTTTTGCTGAGTTGAAGGTGATAGG    33
JUSR         ...............................TATATTTTTGCTGAGTTGAAGGTGATAGG    29
NCPSR-1      ...........................ATATTATATTTTTGCTGAGTTGAAGGTGATAGG    33
NCPSR-2      ...........................ATATTATATTTTTGCTGAGTTGAAGGTGATAGG    33
NCPSR-3      ...........................ATATTATATTTTTGCTGAGTTGAAGGTGATAGG    33
NHRPSR-1     ...........................ATATTATATTTTTGCTGAGTTGAAGGTGATAGG    33
NHRPSR-2     ...........................ATATTATATTTTTGCTGAGTTGAAGGTGATAGG    33
NHRPSR-3     ...........................ATATTATATTTTTGCTGAGTTGAAGGTGATAGG    33
NHRPSR-4     ...........................ATATTATATTTTTGCTGAGTTGAAGGTGATAGG    33
NHRPSR-5     ...........................ATATTATATTTTTGCTGAGTTGAAGGTGATAGG    33
SWWR-1       ...........................ATATTATATTTTTGCTGAGTTGAAGGTGATAGG    33
SWWR-2       ...........................ATATTATATTTTTGCTGAGTTGAAGGTGATAGG    33
SWWR-3       ...........................ATATTATATTTTTGCTGAGTTGAAGGTGATAGG    33
SWWR-4       ...............................TATATTTTTGCTGAGTTGAAGGTGATAGG    29
WUSR-1       ...........................ATATTATATTTTTGCTGAGTTGAAGGTGATAGG    33
WUSR-2       ...........................ATATTATATTTTTGCTGAGTTGAAGGTGATAGG    33
WWR-1        ...........................ATATTATATTTTTGCTGAGTTGAAGGTGATAGG    33
WWR-2        ...........................ATATTATATTTTTGCTGAGTTGAAGGTGATAGG    33
WWR-3        ...........................ATATTATATTTTTGCTGAGTTGAAGGTGATAGG    33
WWRNC-1      ...........................ATATTATATTTTTGCTGAGTTGAAGGTGATAGG    33
WWRNC-2      ................................ATATTTTTGCTGAGTTGAAGGTGATAGG    28
WWRNC-3      ...........................ATATTATATTTTTGCTGAGTTGAAGGTGATAGG    33
spelta-1     ...........................ATATTATATTTTTGCTGAGTTGAAGGTGATAGG    33
spelta_6     ...........................ATATTATATTTTTGCTGAGTTGAAGGTGATAGG    33
spelta_7     ...........................ATATTATATTTTTGCTGAGTTGAAGGTGATAGG    33
spelta_11    ...........................ATATTATATTTTTGCTGAGTTGAAGGTGATAGG    33
SWRNC-1      .......AGCGGGATGTAAAACTTCCAATATTATATTTTTGCTGAGTTGAAGGTGATAGG    53
SWRNC-2      TGATGTAAGCGGGATGTAAAACTTTCAATATTATATTTTTGCTGAGTTGAAGGTGATAGG    60

IWVF-1       AGAGAAAAGAGAATAGAAGCATATTACAAATTAACAACCAGTTGTAGCATGTGCCCTAGA    93
IWVF-2       AGAGAAAAGAGAATAGAAGCATATTACAAATTAACAACCAGTTGTAGCATGTGCCCTAGA    93
IWVF-3       AGAGAAAAGAGAATAGAAGCATATTACAAATTAACAACCAGTTGTAGCATGTGCCCTAGA    93
JUSR         AGAGAAAAGAGAATAGAAGCATATTACAAATTAACAACCAGTTGTAGCATGTGCCCTAGA    89
NCPSR-1      AGAGAAAAGAGAATAGAAGCATATTACAAATTAACAACCAGTTGTAGCATGTGCCCTAGA    93
NCPSR-2      AGAGAAAAGAGAATAGAAGCATATTACAAATTAACAACCAGTTGTAGCATGTGCCCTAGA    93
NCPSR-3      AGAGAAAAGAGAATAGAAGCATATTACAAATTAACAACCAGTTGTAGCATGTGCCCTAGA    93
NHRPSR-1     AGAGAAAAGAGAATAGAAGCATATTACAAATTAACAACCAGTTGTAGCATGTGCCCTAGA    93
NHRPSR-2     AGAGAAAAGAGAATAGAAGCATATTACAAATTAACAACCAGTTGTAGCATGTGCCCTAGA    93
NHRPSR-3     AGAGAAAAGAGAATAGAAGCATATTACAAATTAACAACCAGTTGTAGCATGTGCCCTAGA    93
NHRPSR-4     AGAGAAAAGAGAATAGAAGCATATTACAAATTAACAACCAGTTGTAGCATGTGCCCTAGA    93
NHRPSR-5     AGAGAAAAGAGAATAGAAGCATATTACAAATTAACAACCAGTTGTAGCATGTGCCCTAGA    93
SWWR-1       AGAGAAAAGAGAATAGAAGCATATTACAAATTAACAACCAGTTGTAGCATGTGCCCTAGA    93
SWWR-2       AGAGAAAAGAGAATAGAAGCATATTACAAATTAACAACCAGTTGTAGCATGTGCCCTAGA    93
SWWR-3       AGAGAAAAGAGAATAGAAGCATATTACAAATTAACAACCAGTTGTAGCATGTGCCCTAGA    93
SWWR-4       AGAGAAAAGAGAATAGAAGCATATTACAAATTAACAACCAGTTGTAGCATGTGCCCTAGA    89
WUSR-1       AGAGAAAAGAGAATAGAAGCATATTACAAATTAACAACCAGTTGTAGCATGTGCCCTAGA    93
WUSR-2       AGAGAAAAGAGAATAGAAGCATATTACAAATTAACAACCAGTTGTAGCATGTGCCCTAGA    93
WWR-1        AGAGAAAAGAGAATAGAAGCATATTACAAATTAACAACCAGTTGTAGCATGTGCCCTAGA    93
WWR-2        AGAGAAAAGAGAATAGAAGCATATTACAAATTAACAACCAGTTGTAGCATGTGCCCTAGA    93
WWR-3        AGAGAAAAGAGAATAGAAGCATATTACAAATTAACAACCAGTTGTAGCATGTGCCCTAGA    93
WWRNC-1      AGAGAAAAGAGAATAGAAGCATATTACAAATTAACAACCAGTTGTAGCATGTGCCCTAGA    93
WWRNC-2      AGAGAAAAGAGAATAGAAGCATATTACAAATTAACAACCAGTTGTAGCATGTGCCCTAGA    88
WWRNC-3      AGAGAAAAGAGAATAGAAGCATATTACAAATTAACAACCAGTTGTAGCATGTGCCCTAGA    93
spelta-1     AGAGAAAAGAGAATAGAAGCATATTACAAATTAACAACCAGTTGTAGCATGTGCCCTAGA    93
spelta_6     AGAGAAAAGAGAATAGAAGCATATTACAAATTAACAACCAGTTGTAGCATGTGCCCTAGA    93
spelta_7     AGAGAAAAGAGAATAGAAGCATATTACAAATTAACAACCAGTTGTAGCATGTGCCCTAGA    93
spelta_11    AGAGAAAAGAGAATAGAAGCATATTACAAATTAACAACCAGTTGTAGCATGTGCCCTAGA    93
SWRNC-1      AGAGAAAAGAGAATAGAAGCATATTACAAATTAACAACCAGTTGTAGCATGTGCCCTAGA   113
SWRNC-2      AGAGAAAAGAGAATAGAAGCATATTACAAATTAACAACCAGTTGTAGCATGTGCCCTAGA   120
```

```
IWVF-1        GGCTTTTTGAGAGTGTGAGTGAGTCATGTATCAACAAAGTAATACTCTCTCCGTAAACTA    153
IWVF-2        GGCTTTTTGAGAGTGTGAGTGAGTCATGTATCAACAAAGTAATACTCTCTCCGTAAACTA    153
IWVF-3        GGCTTTTTGAGAGTGTGAGTGAGTCATGTATCAATAAAGTAATACTCTCTTCGTAAACTA    153
JUSR          GGCTTTTTGAGAGTGTGAGTGAGTCATGTATCAATAAAGTAATACTCTCTTCGTAAACTA    149
NCPSR-1       GGCTTTTTGAGAGTGTGAGTGAGTCATGTATCAATAAAGTAATACTCTCTTCGTAAACTA    153
NCPSR-2       GGCTTTTTGAGAGTGTGAGTGAGTCATGTATCAATAAAGTAATACTCTCTTCGTAAACTA    153
NCPSR-3       GGCTTTTTGAGAGTGTGAGTGAGTCATGTATCAACAAAGTAATACTCTCTCCGTAAACTA    153
NHRPSR-1      GGCTTTTTGAGAGTGTGAGTGAGTCATGTATCAATAAAGTAATACTCTCTTCGTAAACTA    153
NHRPSR-2      GGCTTTTTGAGAGTGTGAGTGAGTCATGTATCAACAAAGTAATACTCTCTCCGTAAACTA    153
NHRPSR-3      GGCTTTTTGAGAGTGTGAGTGAGTCATGTATCAACAAAGTAATACTCTCTCCGTAAACTA    153
NHRPSR-4      GGCTTTTTGAGAGTGTGAGTGAGTCATGTATCAATAAAGTAATACTCTCTTCGTAAACTA    153
NHRPSR-5      GGCTTTTTGAGAGTGTGAGTGAGTCATGTATCAACAAAGTAATACTCTCTCCGTAAACTA    153
SWWR-1        GGCTTTTTGAGAGTGTGAGTGAGTCATGTATCAACAAAGTAATACTCTCTCCGTAAACTA    153
SWWR-2        GGCTTTTTGAGAGTGTGAGTGAGTCATGTATCAACAAAGTAATACTCTCTCCGTAAACTA    153
SWWR-3        GGCTTTTTGAGAGTGTGAGTGAGTCATGTATCAATAAAGTAATACTCTCTTCGTAAACTA    153
SWWR-4        GGCTTTTTGAGAGTGTGAGTGAGTCATGTATCAACAAAGTAATACTCTCTCCGTAAACTA    149
WUSR-1        GGCTTTTTGAGAGTGTGAGTGAGTCATGTATCAACAAAGTAATACTCTCTCCGTAAACTA    153
WUSR-2        GGCTTTTTGAGAGTGTGAGTGAGTCATGTATCAATAAAGTAATACTCTCTTCGTAAACTA    153
WWR-1         GGCTTTTTGAGAGTGTGAGTGAGTCATGTATCAACAAAGTAATACTCTCTCCGTAAACTA    153
WWR-2         GGCTTTTTGAGAGTGTGAGTGAGTCATGTATCAATAAAGTAATACTCTCTTCGTAAACTA    153
WWR-3         GGCTTTTTGAGAGTGTGAGTGAGTCATGTATCAACAAAGTAATACTCTCTCCGTAAACTA    153
WWRNC-1       GGCTTTTTGAGAGTGTGAGTGAGTCATGTATCAACAAAGTAATACTCTCTCCGTAAACTA    153
WWRNC-2       GGCTTTTTGAGAGTGTGAGTGAGTCATGTATCAATAAAGTAATACTCTCTTCGTAAACTA    148
WWRNC-3       GGCTTTTTGAGAGTGTGAGTGAGTCATGTATCAACAAAGTAATACTCTCTCCGTAAACTA    153
spelta-1      GGCTTTTTGAGAGTGTGAGTGAGTCATGTATCAACAAAGTAATACTCTCTCCGTAAACTA    153
spelta_6      GGCTTTTTGAGAGTGTGAGTGAGTCATGTATCAACAAAGTAATACTCTCTCCGTAAACTA    153
spelta_7      GGCTTTTTGAGAGTGTGAGTGAGTCATGTATCAACAAAGTAATACTCTCTCCGTAAACTA    153
spelta_11     GGCTTTTTGAGAGTGTGAGTGAGTCATGTATCAACAAAGTAATACTCTCTCCGTAAACTA    153
SWRNC-1       GGCTTTTTGAGAGTGTGAGTGAGTCATGTATCAACAAAGTAATACTCTCTCCGTAAACTA    173
SWRNC-2       GGCTTTTTGAGAGTGTGAGTGAGTCATGTATCAATAAAGTAATACTCTCTTCGTAAACTA    180

IWVF-1        ATATAAACGCATTTAGATTACTATTTTAATGATCTAAATGCTTTTATATTAGTTTACAGA    213
IWVF-2        ATATAAAAGCATTTAGATTACTATTTTAATGATCTAAATGCTTTTATATTAGTTTACAGA    213
IWVF-3        ATATAAAAGCATTTAGATTACTATTTTAATGATCTAAATGCTTTTATATTAGTTTAC.GA    212
JUSR          ATATAAAAGCATTTAGATTACTATTTTAATGATCTAAATGCTTTTATATTAGTTTAC.GA    208
NCPSR-1       ATATAAAAGCATTTAGATTACTATTTTAATGATCTAAATGCTTTTATATTAGTTTAC.GA    212
NCPSR-2       ATATAAAAGCATTTAGATTACTATTTTAATGATCTAAATGCTTTTATATTAGTTTAC.GA    212
NCPSR-3       ATATAAACGCATTTAGATTACTATTTTAATGATCTAAATGCTTTTATATTAGTTTACAGA    213
NHRPSR-1      ATATAAAAGCATTTAGATTACTATTTTAATGATCTAAATGCTTTTATATTAGTTTAC.GA    212
NHRPSR-2      ATATAAAAGCATTTAGATTATTATTTTAATGATCTAAATGCTTTTATATTAGTTTACAGA    213
NHRPSR-3      ATATAAACGCATTTAGATTACTATTTTAATGATCTAAATGCTTTTATATTAGTTTACAGA    213
NHRPSR-4      ATATAAAAGCATTTAGATTACTATTTTAATGATCTAAATGCTTTTATATTAGTTTAC.GA    212
NHRPSR-5      ATATAAAAGCATTTAGATTACTATTTTAATGATCTAAATGCTTTTATATTAGTTTACAGA    213
SWWR-1        ATATAAACGCATTTAGATTACTATTTTAATGATCTAAATGCTTTTATATTAGTTTACAGA    213
SWWR-2        ATATAAAAGCATTTAGATTACTATTTTAATGATCTAAATGCTTTTATATTAGTTTACAGA    213
SWWR-3        ATATAAAAGCATTTAGATTACTATTTTAATGATCTAAATGCTTTTATATTAGTTTAC.GA    212
SWWR-4        ATATAAACGCATTTAGATTACTATTTTAATGATCTAAATGCTTTTATATTAGTTTACAGA    209
WUSR-1        ATATAAAAGCATTTAGATTACTATTTTAATGATCTAAATGCTTTTATATTAGTTTACAGA    213
WUSR-2        ATATAAAAGCATTTAGATTACTATTTTAATGATCTAAATGCTTTTATATTAGTTTAC.GA    212
WWR-1         ATATAAACGCATTTAGATTACTATTTTAATGATCTAAATGCTTTTATATTAGTTTACAGA    213
WWR-2         ATATAAAAGCATTTAGATTACTATTTTAATGATCTAAATGCTTTTATATTAGTTTAC.GA    212
WWR-3         ATATAAACGCATTTAGATTACTATTTTAATGATCTAAATGCTTTTATATTAGTTTACAGA    213
WWRNC-1       ATATAAAAGCATTTAGATTACTATTTTAATGATCTAAATGCTTTTATATTAGTTTACAGA    213
WWRNC-2       ATATAAAAGCATTTAGATTACTATTTTAATGATCTAAATGCTTTTATATTAGTTTAC.GA    207
WWRNC-3       ATATAAACGCATTTAGATTACTATTTTAATGATCTAAATGCTTTTATATTAGTTTACAGA    213
spelta-1      ATATAAAAGCATTTAGATTACTATTTTAATGATCTAAATGCTTTTATATTAGTTTACAGA    213
spelta_6      ATATAAACGCATTTAGATTACTATTTTAATGATCTAAATACTTTTATATTAGTTTACAGA    213
spelta_7      ATATAAACGCATTTAGATTACTATTTTAATGATCTAAATGCTTTTATATTAGTTTACAGA    213
spelta_11     ATATAAACGCATTTAGATTACTATTTTAATGATCTAAATACTTTTATATTAGTTTACAGA    213
SWRNC-1       ATATAAAAGCATTTAGATTACTATTTTAATGATCTAAATGCTTTTATATTAGTTTACAGA    233
SWRNC-2       ATATAAAAGCATTTAGATTACTATTTTAATGATCTAAATGCTTTTATATTAGTTTAC.GA    239
```

```
IWVF-1       GGGAGTACTACATTTTTATATCTAACTATTATACTTGCCAGCTATAAGCGTAATTATAGA    273
IWVF-2       GGGAGTACTACATTTTTATATCTAACTATTATACTTGCCAGCTATAAGCGTAATTATAGA    273
IWVF-3       GGGAGTACTACATTTTTATATCTAACTATTATACTTGCCAGCTATAAGCGTAATTATAGA    272
JUSR         GGGAGTACTACATTTTTATATCTAACTATTATACTTGCCAGCTATAAGCGTAATTATAGA    268
NCPSR-1      GGGAGTACTACATTTTTATATCTAACTATTATACTTGCCAGCTATAAGCGTAATTATAGA    272
NCPSR-2      GGGAGTACTACATTTTTATATCTAACTATTATACTTGCCAGCTATAAGCGTAATTATAGA    272
NCPSR-3      GGGAGTACTACATTTTTATATCTAACTATTATACTTGCCAGCTATAAGCGTAATTATAGA    273
NHRPSR-1     GGGAGTACTACATTTTTATATCTAACTATTATACTTGCCAGCTATAAGCGTAATTATAGA    272
NHRPSR-2     GGGAGTACTACATTTTTATATCTAACTATTATACTTGCCAGCTATAAGCGTAATTATAGA    273
NHRPSR-3     GGGAGTACTACATTTTTATATCTAACTATTATACTTGCCAGCTATAAGCGTAATTATAGA    273
NHRPSR-4     GGGAGTACTACATTTTTATATCTAACTATTATACTTGCCAGCTATAAGCGTAATTATAGA    272
NHRPSR-5     GGGAGTACTACATTTTTATATCTAACTATTATACTTGCCAGCTATAAGCGTAATTATAGA    273
SWWR-1       GGGAGTACTACATTTTTATATCTAACTATTATACTTGCCAGCTATAAGCGTAATTATAGA    273
SWWR-2       GGGAGTACTACATTTTTATATCTAACTATTATACTTGCCAGCTATAAGCGTAATTATAGA    273
SWWR-3       GGGAGTACTACATTTTTATATCTAACTATTATACTTGCCAGCTATAAGCGTAATTATAGA    272
SWWR-4       GGGAGTACTACATTTTTATATCTAACTATTATACTTGCCAGCTATAAGCGTAATTATAGA    269
WUSR-1       GGGAGTACTACATTTTTATATCTAACTATTATACTTGCCAGCTATAAGCGTAATTATAGA    273
WUSR-2       GGGAGTACTACATTTTTATATCTAACTATTATACTTGCCAGCTATAAGCGTAATTATAGA    272
WWR-1        GGGAGTACTACATTTTTATATCTAACTATTATACTTGCCAGCTATAAGCGTAATTATAGA    273
WWR-2        GGGAGTACTACATTTTTATATCTAACTATTATACTTGCCAGCTATAAGCGTAATTATAGA    272
WWR-3        GGGAGTACTACATTTTTATATCTAACTATTATACTTGCCAGCTATAAGCGTAATTATAGA    273
WWRNC-1      GGGAGTACTACATTTTTATATCTAACTATTATACTTGCCAGCTATAAGCGTAATTATAGA    273
WWRNC-2      GGGAGTACTACATTTTTATATCTAACTATTATACTTGCCAGCTATAAGCGTAATTATAGA    267
WWRNC-3      GGGAGTACTACATTTTTATATCTAACTATTATACTTGCCAGCTATAAGCGTAATTATAGA    273
spelta-1     GGGAGTACTACATTTTTATATCTAACTATTATACTTGCCAGCTATAAGCGTAATTATAGA    273
spelta_6     GGGAGTACTACATTTTTATATCTAACTATTATACGTGCCAGCTATAAGCGTAATTATAGA    273
spelta_7     GGGAGTACTACATTTTTATATCTAACTATTATACTTGCCAGCTATAAGCGTAATTATAGA    273
spelta_11    GGGAGTACTACATTTTTATATCTAACTATTATACGTGCCAGCTATAAGCGTAATTATAGA    273
SWRNC-1      GGGAGTACTACATTTTTATATCTAACTATTATACTTGCCAGCTATAAGCGTAATTATAGA    293
SWRNC-2      GGGAGTACTACATTTTTATATCTAACTATTATACTTGCCAGCTATAAGCGTAATTATAGA    299

IWVF-1       TGACGCGTCAACATCATATAGCCAGCAGCCGGCTATGCTATTAACCATGCTCTCGACCAT    333
IWVF-2       TGACGCGTCAACATCATATAGCCAGCAGCCGGCTATGCTATTAACCATGCTCTCAACCAT    333
IWVF-3       TGACGCGTCAACATCATATAGCCAGCAGCCGGCTATGCTATTAACCATGCTCTCGACCAT    332
JUSR         TGACGCGTCAACATCATATAGCCAGCAGCCGGCTATGCTATTAACCATGCTCTCGACCAT    328
NCPSR-1      TGACGCGTCAACATCATATAGCCAGCAGCCGGCTATGCTATTAACCATGCTCTCGACCAT    332
NCPSR-2      TGACGCGTCAACGTCATATAACCAGCAGCCGGCTATGCTATTAACCATGCTCTCGACCAT    332
NCPSR-3      TGACGCGTCAACATCATATAGCCAGCAGCCGGCTATGCTATTAACCATGCTCTCGACCAT    333
NHRPSR-1     TGACGCGTCAACATCATATAGCCAGCAGCCGGCTATGCTATTAACCATGCTCTCGACCAT    332
NHRPSR-2     TGACGCGTCAACATCATATAGCCAGCAGCCGGCTATGCTATTAACCATGCTCTCAACCAT    333
NHRPSR-3     TGACGCGTCAACATCATATAGCCAGCAGCCGGCTATGCTATTAACCATGCTCTCGACCAT    333
NHRPSR-4     TGACGCGTCAACATCATATAGCCAGCAGCCGGCTATGCTATTAACCATGCTCTCGACCAT    332
NHRPSR-5     TGACGCGTCAACATCATATAGCCAGCAGCCGGCTATGCTATTAACCATGCTCTCAACCAT    333
SWWR-1       TGACGCGTCAACATCATATAGCCAGCAGCCGGCTATGCTATTAACCATGCTCTCGACCAT    333
SWWR-2       TGACGCGTCAACATCATATAGCCAGCAGCCGGCTATGCTATTAACCATGCTCTCAACCAT    333
SWWR-3       TGACGCGTCAACATCATATAGCCAGCAGCCGGCTATGCTATTAACCATGCTCTCGACCAT    332
SWWR-4       TGACGCGTCAACATCATATAGCCAGCAGCCGGCTATGCTATTAACCATGCTCTCGACCAT    329
WUSR-1       TGACGCGTCAACATCATATAGCCAGCAGCCGGCTATGCTATTAACCATGCTCTCAACCAT    333
WUSR-2       TGACGCGTCAACATCATATAGCCAGCAGCCGGCTATGCTATTAACCATGCTCTCGACCAT    332
WWR-1        TGACGCGTCAACATCATATAGCCAGCAGCCGGCTATGCTATTAACCATGCTCTCGACCAT    333
WWR-2        TGACGCGTCAACATCATATAGCCAGCAGCCGGCTATGCTATTAACCATGCTCTCGACCAT    332
WWR-3        TGACGCGTCAACATCATATAGCCAGCAGCCGGCTATGCTATTAACCATGCTCTCGACCAT    333
WWRNC-1      TGACGCGTCAACATCATATAGCCAGCAGCCGGCTATGCTATTAACCATGCTCTCAACCAT    333
WWRNC-2      TGACGCGTCAACATCATATAGCCAGCAGCCGGCTATGCTATTAACCATGCTCTCGACCAT    327
WWRNC-3      TGACGCGTCAACATCATATAGCCAGCAGCCGGCTATGCTATTAACCATGCTCTCGACCAT    333
spelta-1     TGACGCGTCAACATCATATAGCCAGCAGCCGGCTATGCTATTAACCATGCTCTCAACCAT    333
spelta_6     TGACGCGTCAACATCATATAGCCAGCAGCCGGCTATGCTATTAACCATGCTCTCGACCAT    333
spelta_7     TGACGCGTCAACATCATATAGCCAGCAGCCGGCTATGCTATTAACCATGCTCTCAACCAT    333
spelta_11    TGACGCGTCAACATCATATAGCCAGCAGCCGGCTATGCTATTAACCATGCTCTCGACCAT    333
SWRNC-1      TGACGCGTCAACATCATATAGCCAGCAGCCGGCTATGCTATTAACCATGCTCTCAACCAT    353
SWRNC-2      TGACGCGTCAACATCATATAGCCAGCAGCCGGCTATGCTATTAACCATGCTCTCGACCAT    359
```

```
IWVF-1       GTATCGGTGATGTCATTATTGTTCATTCAATCACTTTCCAATTTAATACTTTCTACAGAT    393
IWVF-2       GTATCGGTGATGTCATTATTGTTCATTCAATCACTTTCCAATCTAATACTTTCTACAGAT    393
IWVF-3       GTATCGGTGATGTCATTATTGTTCATTCAATCACTTTCCAATTTAATACTTTCTACAGAT    392
JUSR         GTATCGGTGATGTCATTATTGTTCATTCAATCACTTTCCAATTTAATACTTTCTACAGAT    388
NCPSR-1      GTATCGGTGATGTCATTATTGTTCATTCAATCACTTTCCAATTTAATACTTTCTACAGAT    392
NCPSR-2      GTATCGGTGATGTCATTATTGTTCATTCAATCACTTTCCAATTTAATACTTTCTACAGAT    392
NCPSR-3      GTATCGGTGATGTCATTATTGTTCATTCAATCACTTTCCAATTTAATACTTTCTACAGAT    393
NHRPSR-1     GTATCGGTGATGTCATTATTGTTCATTCAATCACTTTCCAATTTAATACTTTCTACAGAT    392
NHRPSR-2     GTATCGGTGATGTCATTATTGTTCATTCAATCACTTTCCAATCTAATACTTTCTACAGAT    393
NHRPSR-3     GTATCGGTGATGTCATTATTGTTCATTCAATCACTTTCCAATTTAATACTTTCTACAGAT    393
NHRPSR-4     GTATCGGTGATGTCATTATTGTTCATTCAATCACTTTCCAATTTAATACTTTCTACAGAT    392
NHRPSR-5     GTATCGGTGATGTCATTATTGTTCATTCAATCACTTTCCAATCTAATACTTTCTACAGAT    393
SWWR-1       GTATCGGTGATGTCATTATTGTTCATTCAATCACTTTCCAATTTAATACTTTCTACAGAT    393
SWWR-2       GTATCGGTGATGTCATTATTGTTCATTCAATCACTTTCCAATCTAATACTTTCTACAGAT    393
SWWR-3       GTATCGGTGATGTCATTATTGTTCATTCAATCACTTTCCAATTTAATACTTTCTACAGAT    392
SWWR-4       GTATCGGTGATGTCATTATTGTTCATTCAATCACTTTCCAATTTAATACTTTCTACAGAT    389
WUSR-1       GTATCGGTGATGTCATTATTGTTCATTCAATCACTTTCCAATCTAATACTTTCTACAGAT    393
WUSR-2       GTATCGGTGATGTCATTATTGTTCATTCAATCACTTTCCAATTTAATACTTTCTACAGAT    392
WWR-1        GTATCGGTGATGTCATTATTGTTCATTCAATCACTTTCCAATTTAATACTTTCTACAGAT    393
WWR-2        GTATCGGTGATGTCATTATTGTTCATTCAATCACTTTCCAATTTAATACTTTCTACAGAT    392
WWR-3        GTATCGGTGATGTCATTATTGTTCATTCAATCACTTTCCAATTTAATACTTTCTACAGAT    393
WWRNC-1      GTATCGGTGATGTCATTATTGTTCATTCAATCACTTTCCAATCTAATACTTTCTACAGAT    393
WWRNC-2      GTATCGGTGATGTCATTATTGTTCATTCAATCACTTTCCAATTTAATACTTTCTACAGAT    387
WWRNC-3      GTATCGGTGATGTCATTATTGTTCATTCAATCACTTTCCAATTTAATACTTTCTACAGAT    393
spelta-1     GTATCGGTGATGTCATTATTGTTCATTCAATCACTTTCCAATCTAATACTTTCTACAGAT    393
spelta_6     GTATCGGTGATGTCATTATTGTTCATTCAATCACTTTCCAATTTAATACTTTCTACAGAT    393
spelta_7     GTATCGGTGATGTCATTATTGTTCATTCAATCACTTTCCAATCTAATACTTTCTACAGAT    393
spelta_11    GTATCGGTGATGTCATTATTGTTCATTCAATCACTTTCCAATTTAATACTTTCTACAGAT    393
SWRNC-1      GTATCGGTGATGTCATTATTGTTCATTCAATCACTTTCCAATCTAATACTTTCTACAGAT    413
SWRNC-2      GTATCGGTGATGTCATTATTGTTCATTCAATCACTTTCCAATTTAATACTTTCTACAGAT    419
```

```
IWVF-1       AGGATATAAACATGGCTAAACTTACCCTTGATTAATCGACGGCCGGTATCACATATCTTC    453
IWVF-2       AGGATATAAATATGGCTAAACTTACCCTTGATTAATCGACGGCCGGTATCACATATCTTC    453
IWVF-3       AGGATATAAATATGGCTAAACTTACCCTTGATTAATCGACGGCCGGTATCACATATCTTC    452
JUSR         AGGATATAAATATGGCTAAACTTACCCTTGATTAATCGACGGCCGGTATCACATATCTTC    448
NCPSR-1      AGGATATAAATATGGCTAAACTTACCCTTGATTAATCGACGGCCGGTATCACATATCTTC    452
NCPSR-2      AGGATATAAATATGGCTAAACTTACCCTTGATTAATCGACGGCCGGTATCACATATCTTC    452
NCPSR-3      AGGATATAAACATGGCTAAACTTACCCTTGATTAATCGACGGCCGGTATCACATATCTTC    453
NHRPSR-1     AGGATATAAATATGGCTAAACTTACCCTTGATTAATCGACGGCCGGTATCACATATCTTC    452
NHRPSR-2     AGGATATAAATATGGCTAAACTTACCCTTGATTAATCGACGGCCGGTATCACATATCTTC    453
NHRPSR-3     AGGATATAAACATGGCTAAACTTACCCTTGATTAATCGACGGCCGGTATCACATATCTTC    453
NHRPSR-4     AGGATATAAATATGGCTAAACTTACCCTTGATTAATCGACGGCCGGTATCACATATCTTC    452
NHRPSR-5     AGGATATAAATATGGCTAAACTTACCCTTGATTAATCGACGGCCGGTATCACATATCTTC    453
SWWR-1       AGGATATAAACATGGCTAAACTTACCCTTGATTAATCGACGGCCGGTATCACATATCTTC    453
SWWR-2       AGGATATAAATATGGCTAAACTTACCCTTGATTAATCGACGGCCGGTATCACATATCTTC    453
SWWR-3       AGGATATAAATATGGCTAAACTTACCCTTGATTAATCGACGGCCGGTATCACATATCTTC    452
SWWR-4       AGGATATAAACATGGCTAAACTTACCCTTGATTAATCGACGGCCGGTATCACATATCTTC    449
WUSR-1       AGGATATAAATATGGCTAAACTTACCCTTGATTAATCGACGGCCGGTATCACATATCTTC    453
WUSR-2       AGGATATAAATATGGCTAAACTTACCCTTGATTAATCGACGGCCGGTATCACATATCTTC    452
WWR-1        AGGATATAAACATGGCTAAACTTACCCTTGATTAATCGACGGCCGGTATCACATATCTTC    453
WWR-2        AGGATATAAATATGGCTAAACTTACCCTTGATTAATCGACGGCCGGTATCACATATCTTC    452
WWR-3        AGGATATAAACATGGCTAAACTTACCCTTGATTAATCGACGGCCGGTATCACATATCTTC    453
WWRNC-1      AGGATATAAATATGGCTAAACTTACCCTTGATTAATCGACGGCCGGTATCACATATCTTC    453
WWRNC-2      AGGATATAAATATGGCTAAACTTACCCTTGATTAATCGACGGCCGGTATCACATATCTTC    447
WWRNC-3      AGGATATAAACATGGCTAAACTTACCCTTGATTAATCGACGGCCGGTATCACATATCTTC    453
spelta-1     AGGATATAAATATGGCTAAACTTACCCTTGATTAATCGACGGCCGGTATCACATATCTTC    453
spelta_6     AGGATATAAATATGGCTAAACTTACCCTTGATTAATCGACGGCCGGTATCACATATCTTC    453
spelta_7     AGGATATAAATATGGCTAAACTTACCCTTGATTAATCGACGGCCGGTATCACATATCTTC    453
spelta_11    AGGATATAAATATGGCTAAACTTACCCTTGATTAATCGACGGCCGGTATCACATATCTTC    453
SWRNC-1      AGGATATAAATATGGCTAAACTTACCCTTGATTAATCGACGGCCGGTATCACATATCTTC    473
SWRNC-2      AGGATATAAATATGGCTAAACTTACCCTTGATTAATCGACGGCCGGTATCACATATCTTC    479
```

```
IWVF-1      TGTTATCATAAGTCGAAGCATCTTAAAAGAGCTCCTGACAAAACCTTGATTGTACGTAAC   513
IWVF-2      TGTTATCATAAGTCGAAGCATCTTAAAAGAGCTCCTGACAAAACCTTGATTGTACGTAAC   513
IWVF-3      TGTTATCATAAGTCGAAGCATCTTAAAAGAGCTCCTGACAAAACCTTGATTGTACGTAAC   512
JUSR        TGTTATCATAAGTCGAAGCATCTTAAAAGAGCTCCTGACAAAACCTTGATTGTACGTAAC   508
NCPSR-1     TGTTATCATAAGTCGAAGCATCTTAAAAGAGCTCCTGACAAAACCTTGATTGTACGTAAC   512
NCPSR-2     TGTTATCATAAGTCGAAGCATCTTAAAAGAGCTCCTGACAAAACCTTGATTGTACGTAAC   512
NCPSR-3     TGTTATCATAAGTCGAAGCATCTTAAAAGAGCTCCTGACAAAACCTTGATTGTACGTAAC   513
NHRPSR-1    TGTTATCATAAGTCGAAGCATCTTAAAAGAGCTCCTGACAAAACCTTGATTGTACGTAAC   512
NHRPSR-2    TGTTATCATAAGTCGAAGCATCTTAAAAGAGCTCCTGACAAAACCTTGATTGTACGTAAC   513
NHRPSR-3    TGTTATCATAAGTCGAAGCATCTTAAAAGAGCTCCTGACAAAACCTTGATTGTACGTAAC   513
NHRPSR-4    TGTTATCATAAGTCGAAGCATCTTAAAAGAGCTCCTGACAAAACCTTGATTGTACGTAAC   512
NHRPSR-5    TGTTATCATAAGTCGAAGCATCTTAAAAGAGCTCCTGACAAAACCTTGATTGTACGTAAC   513
SWWR-1      TGTTATCATAAGTCGAAGCATCTTAAAAGAGCTCCTGACAAAACCTTGATTGTACGTAAC   513
SWWR-2      TGTTATCATAAGTCGAAGCATCTTAAAAGAGCTCCTGACAAAACCTTGATTGTACGTAAC   513
SWWR-3      TGTTATCATAAGTCGAAGCATCTTAAAAGAGCTCCTGACAAAACCTTGATTGTACGTAAC   512
SWWR-4      TGTTATCATAAGTCGAAGCATCTTAAAAGAGCTCCTGACAAAACCTTGATTGTACGTAAC   509
WUSR-1      TGTTATCATAAGTCGAAGCATCTTAAAAGAGCTCCTGACAAAACCTTGATTGTACGTAAC   513
WUSR-2      TGTTATCATAAGTCGAAGCATCTTAAAAGAGCTCCTGACAAAACCTTGATTGTACGTAAC   512
WWR-1       TGTTATCATAAGTCGAAGCATCTTAAAAGAGCTCCTGACAAAACCTTGATTGTACGTAAC   513
WWR-2       TGTTATCATAAGTCGAAGCATCTTAAAAGAGCTCCTGACAAAACCTTGATTGTACGTAAC   512
WWR-3       TGTTATCATAAGTCGAAGCATCTTAAAAGAGCTCCTGACAAAACCTTGATTGTACGTAAC   513
WWRNC-1     TGTTATCATAAGTCGAAGCATCTTAAAAGAGCTCCTGACAAAACCTTGATTGTACGTAAC   513
WWRNC-2     TGTTATCATAAGTCGAAGCATCTTAAAAGAGCTCCTGACAAAACCTTGATTGTACGTAAC   507
WWRNC-3     TGTTATCATAAGTCGAAGCATCTTAAAAGAGCTCCTGACAAAACCTTGATTGTACGTAAC   513
spelta-1    TGTTATCATAAGTCGAAGCATCTTAAAAGAGCTCCTGACAAAACCTTGATTGTACGTAAC   513
spelta_6    TGTTATCATAAGTCGAAGCATCTTAAAAGAGCTCCTGACAAAACCTTGATTGTACGTAAC   513
spelta_7    TGTTATCATAAGTCGAAGCATCTTAAAAGAGCTCCTGACAAAACCTTGATTGTACGTAAC   513
spelta_11   TGTTATCATAAGTCGAAGCATCTTAAAAGAGCTCCTGACAAAACCTTGATTGTACGTAAC   513
SWRNC-1     TGTTATCATAAGTCGAAGCATCTTAAAAGAGCTCCTGACAAAACCTTGATTGTACGTAAC   533
SWRNC-2     TGTTATCATAAGTCGAAGCATCTTAAAAGAGCTCCTGACAAAACCTTGATTGTACGTAAC   539
```

```
IWVF-1      CTTCAAACGTGGACAGTAGGATTTGTTTCTTCGATCCGCCCCAAACAGGTCATGTAGGTA   573
IWVF-2      CTTCAAACGTGGACAGTAGGATTTGTTTCTTCGATCCGCCCCAAACAGGTCATGTAGGTA   573
IWVF-3      CTTCAAACGTGGACAGTAGGATTTGTTTCTTCGATCCGCCCCAAACAGGTCATGTAGGTA   572
JUSR        CTTCAAACGTGGACAGTAGGATTTGTTTCTTCGATCCGCCCCAAACAGGTCATGTAGGTA   568
NCPSR-1     CTTCAAACGTGGACAGTAGGATTTGTTTCTTCGATCCGCCCCAAACAGGTCATGTAGGTA   572
NCPSR-2     CTTCAAACGTGGACAGTAGGATTTGTTTCTTCGATCCGCCCCAAACAGGTCATGTAGGTA   572
NCPSR-3     CTTCAAACGTGGACAGTAGGATTTGTTTCTTCGATCCGCCCCAAACAGGTCATGTAGGTA   573
NHRPSR-1    CTTCAAACGTGGACAGTAGGATTTGTTTCTTCGATCCGCCCCAAACAGGTCATGTAGGTA   572
NHRPSR-2    CTTCAAACGTGGACAGTAGGATTTGTTTCTTCGATCCGCCCCAAACAGGTCATGTAGGTA   573
NHRPSR-3    CTTCAAACGTGGACAGTAGGATTTGTTTCTTCGATCCGCCCCAAACAGGTCATGTAGGTA   573
NHRPSR-4    CTTCAAACGTGGACAGTAGGATTTGTTTCTTCGATCCGCCCCAAACAGGTCATGTAGGTA   572
NHRPSR-5    CTTCAAACGTGGACAGTAGGATTTGTTTCTTCGATCCGCCCCAAACAGGTCATGTAGGTA   573
SWWR-1      CTTCAAACGTGGACAGTAGGATTTGTTTCTTCGATCCGCCCCAAACAGGTCATGTAGGTA   573
SWWR-2      CTTCAAACGTGGACAGTAGGATTTGTTTCTTCGATCCGCCCCAAACAGGTCATGTAGGTA   573
SWWR-3      CTTCAAACGTGGACAGTAGGATTTGTTTCTTCGATCCGCCCCAAACAGGTCATGTAGGTA   572
SWWR-4      CTTCAAACGTGGACAGTAGGATTTGTTTCTTCGATCCGCCCCAAACAGGTCATGTAGGTA   569
WUSR-1      CTTCAAACGTGGACAGTAGGATTTGTTTCTTCGATCCGCCCCAAACAGGTCATGTAGGTA   573
WUSR-2      CTTCAAACGTGGACAGTAGGATTTGTTTCTTCGATCCGCCCCAAACAGGTCATGTAGGTA   572
WWR-1       CTTCAAACGTGGACAGTAGGATTTGTTTCTTCGATCCGCCCCAAACAGGTCATGTAGGTA   573
WWR-2       CTTCAAACGTGGACAGTAGGATTTGTTTCTTCGATCCGCCCCAAACAGGTCATGTAGGTA   572
WWR-3       CTTCAAACGTGGACAGTAGGATTTGTTTCTTCGATCCGCCCCAAACAGGTCATGTAGGTA   573
WWRNC-1     CTTCAAACGTGGACAGTAGGATTTGTTTCTTCGATCCGCCCCAAACAGGTCATGTAGGTA   573
WWRNC-2     CTTCAAACGTGGACAGTAGGATTTGTTTCTTCGATCCGCCCCAAACAGGTCATGTAGGTA   567
WWRNC-3     CTTCAAACGTGGACAGTAGGATTTGTTTCTTCGATCCGCCCCAAACAGGTCATGTAGGTA   573
spelta-1    CTTCAAACGTGGACAGTAGGATTTGTTTCTTCGATCCGCCCCAAACAGGTCATGTAGGTA   573
spelta_6    CTTCAAACGTGGACAGTAGGATTTGTTTCTTCGATCCGCCCCAAACAGGTCATGTAGGTA   573
spelta_7    CTTCAAACGTGGACAGTAGGATTTGTTTCTTCGATCCGCCCCAAACAGGTCATGTAGGTA   573
spelta_11   CTTCAAACGTGGACAGTAGGATTTGTTTCTTCGATCCGCCCCAAACAGGTCATGTAGGTA   573
SWRNC-1     CTTCAAACGTGGACAGTAGGATTTGTTTCTTCGATCCGCCCCAAACAGGTCATGTAGGTA   593
SWRNC-2     CTTCAAACGTGGACAGTAGGATTTGTTTCTTCGATCCGCCCCAAACAGGTCATGTAGGTA   599
```

IWVF-1	TACGTGTGTTCCCGTGAGAGGCGGGTGGGGTAAGCAGCTAGTAGTAGCAAGCATGGGTGC	633
IWVF-2	TACGTGTGTTCCCGTGAGAGGCGGGTGGGGTAAGCAGCTAGTAGTAGCAAGCATGGGTGC	633
IWVF-3	TACGTGTGTTCCCGTGAGAGGCGGGTGGGGTAAGCAGCTAGTAGTAGCAAGCATGGGTGC	632
JUSR	TACGTGTGTTCCCGTGAGAGGCGGGTGGGGTAAGCAGCTAGTAGTAGCAAGCATGGGTGC	628
NCPSR-1	TACGTGTGTTCCCGTGAGAGGCGGGTGGGGTAAGCAGCTAGTAGTAGCAAGCATGGGTGC	632
NCPSR-2	TACGTGTGTTCCCGTGAGAGGCGGGTGGGGTAAGCAGCTAGTAGTAGCAAGCATGGGTGC	632
NCPSR-3	TACGTGTGTTCCCGTGAGAGGCGGGTGGGGTAAGCAGCTAGTAGTAGCAAGCATGGGTGC	633
NHRPSR-1	TACGTGTGTTCCCGTGAGAGGCGGGTGGGGTAAGCAGCTAGTAGTAGCAAGCATGGGTGC	632
NHRPSR-2	TACGTGTGTTCCCGTGAGAGGCGGGTGGGGTAAGCAGCTAGTAGTAGCAAGCATGGGTGC	633
NHRPSR-3	TACGTGTGTTCCCGTGAGAGGCGGGTGGGGTAAGCAGCTAGTAGTAGCAAGCATGGGTGC	633
NHRPSR-4	TACGTGTGTTCCCGTGAGAGGCGGGTGGGGTAAGCAGCTAGTAGTAGCAAGCATGGGTGC	632
NHRPSR-5	TACGTGTGTTCCCGTGAGAGGCGGGTGGGGTAAGCAGCTAGTAGTAGCAAGCATGGGTGC	633
SWWR-1	TACGTGTGTTCCCGTGAGAGGCGGGTGGGGTAAGCAGCTAGTAGTAGCAAGCATGGGTGC	633
SWWR-2	TACGTGTGTTCCCGTGAGAGGCGGGTGGGGTAAGCAGCTAGTAGTAGCAAGCATGGGTGC	633
SWWR-3	TACGTGTGTTCCCGTGAGAGGCGGGTGGGGTAAGCAGCTAGTAGTAGCAAGCATGGGTGC	632
SWWR-4	TACGTGTGTTCCCGTGAGAGGCGGGTGGGGTAAGCAGCTAGTAGTAGCAAGCATGGGTGC	629
WUSR-1	TACGTGTGTTCCCGTGAGAGGCGGGTGGGGTAAGCAGCTAGTAGTAGCAAGCATGGGTGC	633
WUSR-2	TACGTGTGTTCCCGTGAGAGGCGGGTGGGGTAAGCAGCTAGTAGTAGCAAGCATGGGTGC	632
WWR-1	TACGTGTGTTCCCGTGAGAGGCGGGTGGGGTAAGCAGCTAGTAGTAGCAAGCATGGGTGC	633
WWR-2	TACGTGTGTTCCCGTGAGAGGCGGGTGGGGTAAGCAGCTAGTAGTAGCAAGCATGGGTGC	632
WWR-3	TACGTGTGTTCCCGTGAGAGGCGGGTGGGGTAAGCAGCTAGTAGTAGCAAGCATGGGTGC	633
WWRNC-1	TACGTGTGTTCCCGTGAGAGGCGGGTGGGGTAAGCAGCTAGTAGTAGCAAGCATGGGTGC	633
WWRNC-2	TACGTGTGTTCCCGTGAGAGGCGGGTGGGGTAAGCAGCTAGTAGTAGCAAGCATGGGTGC	627
WWRNC-3	TACGTGTGTTCCCGTGAGAGGCGGGTGGGGTAAGCAGCTAGTAGTAGCAAGCATGGGTGC	633
spelta-1	TACGTGTGTTCCCGTGAGAGGCGGGTGGGGTAAGCAGCTAGTAGTAGCAAGCATGGGTGC	633
spelta_6	TACGTGTGTTCCCGTGAGAGGCGGGTGGGGTAAGCAGCTAGTAGTAGCAAGCATGGGTGC	633
spelta_7	TACGTGTGTTCCCGTGAGAGGCGGGTGGGGTAAGCAGCTAGTAGTAGCAAGCATGGGTGC	633
spelta_11	TACGTGTGTTCCCGTGAGAGGCGGGTGGGGTAAGCAGCTAGTAGTAGCAAGCATGGGTGC	633
SWRNC-1	TACGTGTGTTCCCGTGAGAGGCGGGTGGGGTAAGCAGCTAGTAGTAGCAAGCATGGGTGC	653
SWRNC-2	TACGTGTGTTCCCGTGAGAGGCGGGTGGGGTAAGCAGCTAGTAGTAGCAAGCATGGGTGC	659

IWVF-1	AGCAGATACCCATTTCTTTTCTTTTATTGGTAAGGAAATTCGACCGTACATTACCACATA	693
IWVF-2	AGCAGATACCCATTTCTTTTCTTTTATTGGTAAGGAAATTCGACCGTACATTACCACATA	693
IWVF-3	AGCAGATACCCATTTCTTTTCTTTTATTGGTAAGGAAATTCGACCGTACATTACCACATA	692
JUSR	AGCAGATACCCATTTCTTTTCTTTTATTGGTAAGGAAATTCGACCGTACATTACCACATA	688
NCPSR-1	AGCAGATACCCATTTCTTTTCTTTTATTGGTAAGGAAATTCGACCGTACATTACCACATA	692
NCPSR-2	AGCAGATACCCATTTCTTTTCTTTTATTGGTAAGGAAATTCGACCGTACATTACCACATA	692
NCPSR-3	AGCAGATACCCATTTCTTTTCTTTTATTGGTAAGGAAATTCGACCGTACATTACCACATA	693
NHRPSR-1	AGCAGATACCCATTTCTTTTCTTTTATTGGTAAGGAAATTCGACCGTACATTACCACATA	692
NHRPSR-2	AGCAGATACCCATTTCTTTTCTTTTATTGGTAAGGAAATTCGACCGTACATTACCACATA	693
NHRPSR-3	AGCAGATACCCATTTCTTTTCTTTTATTGGTAAGGAAATTCGACCGTACATTACCACATA	693
NHRPSR-4	AGCAGATACCCATTTCTTTTCTTTTATTGGTAAGGAAATTCGACCGTACATTACCACATA	692
NHRPSR-5	AGCAGATACCCATTTCTTTTCTTTTATTGGTAAGGAAATTCGACCGTACATTACCACATA	693
SWWR-1	AGCAGATACCCATTTCTTTTCTTTTATTGGTAAGGAAATTCGACCGTACATTACCACATA	693
SWWR-2	AGCAGATACCCATTTCTTTTCTTTTATTGGTAAGGAAATTCGACCGTACATTACCACATA	693
SWWR-3	AGCAGATACCCATTTCTTTTCTTTTATTGGTAAGGAAATTCGACCGTACATTACCACATA	692
SWWR-4	AGCAGATACCCATTTCTTTTCTTTTATTGGTAAGGAAATTCGACCGTACATTACCACATA	689
WUSR-1	AGCAGATACCCATTTCTTTTCTTTTATTGGTAAGGAAATTCGACCGTACATTACCACATA	693
WUSR-2	AGCAGATACCCATTTCTTTTCTTTTATTGGTAAGGAAATTCGACCGTACATTACCACATA	692
WWR-1	AGCAGATACCCATTTCTTTTCTTTTATTGGTAAGGAAATTCGACCGTACATTACCACATA	693
WWR-2	AGCAGATACCCATTTCTTTTCTTTTATTGGTAAGGAAATTCGACCGTACATTACCACATA	692
WWR-3	AGCAGATACCCATTTCTTTTCTTTTATTGGTAAGGAAATTCGACCGTACATTACCACATA	693
WWRNC-1	AGCAGATACCCATTTCTTTTCTTTTATTGGTAAGGAAATTCGACCGTACATTACCACATA	693
WWRNC-2	AGCAGATACCCATTTCTTTTCTTTTATTGGTAAGGAAATTCGACCGTACATTACCACATA	687
WWRNC-3	AGCAGATACCCATTTCTTTTCTTTTATTGGTAAGGAAATTCGACCGTACATTACCACATA	693
spelta-1	AGCAGATACCCATTTCTTTTCTTTTATTGGTAAGGAAATTCGACCGTACATTACCACATA	693
spelta_6	AGCAGATACCCATTTCTTTTCTTTTATTGGTAAGGAAATTCGACCGTACATTACCACATA	693
spelta_7	AGCAGATACCCATTTCTTTTCTTTTATTGGTAAGGAAATTCGACCGTACATTACCACATA	693
spelta_11	AGCAGATACCCATTTCTTTTCTTTTATTGGTAAGGAAATTCGACCGTACATTACCACATA	693
SWRNC-1	AGCAGATACCCATTTCTTTTCTTTTATTGGTAAGGAAATTCGACCGTACATTACCACATA	713
SWRNC-2	AGCAGATACCCATTTCTTTTCTTTTATTGGTAAGGAAATTCGACCGTACATTACCACATA	719

```
IWVF-1       ACCTTGTCGACAACTCTCCGCGCACGGCTGCACCACAGAACCTTGTCGACCGTCGATTGT   753
IWVF-2       ACCTTGTCGACAACTCTCCGCGCACGGCTGCACCACAGAACCTTGTCGACCGTCGATTGT   753
IWVF-3       ACCTTGTCGACAACTCTCCGCGCACGGCTGCACCACAGAACCTTGTCGACCGTCGATTGT   752
JUSR         ACCTTGTCGACAACTCTCCGCGCACGGCTGCACCACAGAACCTTGTCGACCGTCGATTGT   748
NCPSR-1      ACCTTGTCGACAACTCTCCGCGCACGGCTGCACCACAGAACCTTGTCGACCGTCGATTGT   752
NCPSR-2      ACCTTGTCGACAACTCTCCGCGCACGGCTGCACCACAGAACCTTGTCGACCGTCGATTGT   752
NCPSR-3      ACCTTGTCGACAACTCTCCGCGCACGGCTGCACCACAGAACCTTGTCGACCGTCGATTGT   753
NHRPSR-1     ACCTTGTCGACAACTCTCCGCGCACGGCTGCACCACAGAACCTTGTCGACCGTCGATTGT   752
NHRPSR-2     ACCTTGTCGACAACTCTCCGCGCACGGCTGCACCACAGAACCTTGTCGACCGTCGATTGT   753
NHRPSR-3     ACCTTGTCGACAACTCTCCGCGCACGGCTGCACCACAGAACCTTGTCGACCGTCGATTGT   753
NHRPSR-4     ACCTTGTCGACAACTCTCCGCGCACGGCTGCACCACAGAACCTTGTCGACCGTCGATTGT   752
NHRPSR-5     ACCTTGTCGACAACTCTCCGCGCACGGCTGCACCACAGAACCTTGTCGACCGTCGATTGT   753
SWWR-1       ACCTTGTCGACAACTCTCCGCGCACGGCTGCACCACAGAACCTTGTCGACCGTCGATTGT   753
SWWR-2       ACCTTGTCGACAACTCTCCGCGCACGGCTGCACCACAGAACCTTGTCGACCGTCGATTGT   753
SWWR-3       ACCTTGTCGACAACTCTCCGCGCACGGCTGCACCACAGAACCTTGTCGACCGTCGATTGT   752
SWWR-4       ACCTTGTCGACAACTCTCCGCGCACGGCTGCACCACAGAACCTTGTCGACCGTCGATTGT   749
WUSR-1       ACCTTGTCGACAACTCTCCGCGCACGGCTGCACCACAGAACCTTGTCGACCGTCGATTGT   753
WUSR-2       ACCTTGTCGACAACTCTCCGCGCACGGCTGCACCACAGAACCTTGTCGACCGTCGATTGT   752
WWR-1        ACCTTGTCGACAACTCTCCGCGCACGGCTGCACCACAGAACCTTGTCGACCGTCGATTGT   753
WWR-2        ACCTTGTCGACAACTCTCCGCGCACGGCTGCACCACAGAACCTTGTCGACCGTCGATTGT   752
WWR-3        ACCTTGTCGACAACTCTCCGCGCACGGCTGCACCACAGAACCTTGTCGACCGTCGATTGT   753
WWRNC-1      ACCTTGTCGACAACTCTCCGCGCACGGCTGCACCACAGAACCTTGTCGACCGTCGATTGT   753
WWRNC-2      ACCTTGTCGACAACTCTCCGCGCACGGCTGCACCACAGAACCTTGTCGACCGTCGATTGT   747
WWRNC-3      ACCTTGTCGACAACTCTCCGCGCACGGCTGCACCACAGAACCTTGTCGACCGTCGATTGT   753
spelta-1     ACCTTGTCGACAACTCTCCGCGCACGGCTGCACCACAGAACCTTGTCGACCGTCGATTGT   753
spelta_6     ACCTTGTCGACAACTCTCCGCGCACGGCTGCACCACAGAACCTTGTCGACCGTCGATTGT   753
spelta_7     ACCTTGTCGACAACTCTCCGCGCACGGCTGCACCACAGAACCTTGTCGACCGTCGATTGT   753
spelta_11    ACCTTGTCGACAACTCTCCGCGCACGGCTGCACCACAGAACCTTGTCGACCGTCGATTGT   753
SWRNC-1      ACCTTGTCGACAACTCTCCGCGCACGGCTGCACCACAGAACCTTGTCGACCGTCGATTGT   773
SWRNC-2      ACCTTGTCGACAACTCTCCGCGCACGGCTGCACCACAGAACCTTGTCGACCGTCGATTGT   779

IWVF-1       CGCCATGCGCCGCAGCGATCGGCCCCCGACGTGTCACCGCCCACCTCATCCTTCGCCCCT   813
IWVF-2       CGCCATGCGCCGCAGCGATCGGCCCCCGACGTGTCACCGCCCACCTCATCCTTCGCCCCT   813
IWVF-3       CGCCATGCGCCGCAGCGATCGGCCCCCGACGTGTCACCGCCCACCTCATCCTTCGCCCCT   812
JUSR         CGCCATGCGCCGCAGCGATCGGCCCCCGACGTGTCACCGCCCACCTCATCCTTCGCCCCT   808
NCPSR-1      CGCCATGCGCCGCAGCGATCGGCCCCCGACGTGTCACCGCCCACCTCATCCTTCGCCCCT   812
NCPSR-2      CGCCATGCGCCGCAGCGATCGGCCCCCGACGTGTCACCGCCCACCTCATCCTTCGCCCCT   812
NCPSR-3      CGCCATGCGCCGCAGCGATCGGCCCCCGACGTGTCACCGCCCACCTCATCCTTCGCCCCT   813
NHRPSR-1     CGCCATGCGCCGCAGCGATCGGCCCCCGACGTGTCACCGCCCACCTCATCCTTCGCCCCT   812
NHRPSR-2     CGCCATGCGCCGCAGCGATCGGCCCCCGACGTGTCACCGCCCACCTCATCCTTCGCCCCT   813
NHRPSR-3     CGCCATGCGCCGCAGCGATCGGCCCCCGACGTGTCACCGCCCACCTCATCCTTCGCCCCT   813
NHRPSR-4     CGCCATGCGCCGCAGCGATCGGCCCCCGACGTGTCACCGCCCACCTCATCCTTCGCCCCT   812
NHRPSR-5     CGCCATGCGCCGCAGCGATCGGCCCCCGACGTGTCACCGCCCACCTCATCCTTCGCCCCT   813
SWWR-1       CGCCATGCGCCGCAGCGATCGGCCCCCGACGTGTCACCGCCCACCTCATCCTTCGCCCCT   813
SWWR-2       CGCCATGCGCCGCAGCGATCGGCCCCCGACGTGTCACCGCCCACCTCATCCTTCGCCCCT   813
SWWR-3       CGCCATGCGCCGCAGCGATCGGCCCCCGACGTGTCACCGCCCACCTCATCCTTCGCCCCT   812
SWWR-4       CGCCATGCGCCGCAGCGATCGGCCCCCGACGTGTCACCGCCCACCTCATCCTTCGCCCCT   809
WUSR-1       CGCCATGCGCCGCAGCGATCGGCCCCCGACGTGTCACCGCCCACCTCATCCTTCGCCCCT   813
WUSR-2       CGCCATGCGCCGCAGCGATCGGCCCCCGACGTGTCACCGCCCACCTCATCCTTCGCCCCT   812
WWR-1        CGCCATGCGCCGCAGCGATCGGCCCCCGACGTGTCACCGCCCACCTCATCCTTCGCCCCT   813
WWR-2        CGCCATGCGCCGCAGCGATCGGCCCCCGACGTGTCACCGCCCACCTCATCCTTCGCCCCT   812
WWR-3        CGCCATGCGCCGCAGCGATCGGCCCCCGACGTGTCACCGCCCACCTCATCCTTCGCCCCT   813
WWRNC-1      CGCCATGCGCCGCAGCGATCGGCCCCCGACGTGTCACCGCCCACCTCATCCTTCGCCCCT   813
WWRNC-2      CGCCATGCGCCGCAGCGATCGGCCCCCGACGTGTCACCGCCCACCTCATCCTTCGCCCCT   807
WWRNC-3      CGCCATGCGCCGCAGCGATCGGCCCCCGACGTGTCACCGCCCACCTCATCCTTCGCCCCT   813
spelta-1     CGCCATGCGCCGCAGCGATCGGCCCCCGACGTGTCACCGCCCACCTCATCCTTCGCCCCT   813
spelta_6     CGCCATGCGCCGCAGCGATCGGCCCCCGACGTGTCACCGCCCACCTCATCCTTCGCCCCT   813
spelta_7     CGCCATGCGCCGCAGCGATCGGCCCCCGACGTGTCACCGCCCACCTCATCCTTCGCCCCT   813
spelta_11    CGCCATGCGCCGCAGCGATCGGCCCCCGACGTGTCACCGCCCACCTCATCCTTCGCCCCT   813
SWRNC-1      CGCCATGCGCCGCAGCGATCGGCCCCCGACGTGTCACCGCCCACCTCATCCTTCGCCCCT   833
SWRNC-2      CGCCATGCGCCGCAGCGATCGGCCCCCGACGTGTCACCGCCCACCTCATCCTTCGCCCCT   839
```

```
IWVF-1       CCGCGTCCAGCCTATAAAAGAGACGATGCACCTCTCGTCCTCCCATCGTACAGCAAGTGA   873
IWVF-2       CCGCGTCCAGCCTATAAAAGAGACGATGCACCTCTCGTCCTCCCATCGTACAGCAAGTGA   873
IWVF-3       CCGCGTCCAGCCTATAAAAGAGACGATGCACCTCTCGTCCTCCCATCGTACAGCAAGTGA   872
JUSR         CCGCGTCCAGCCTATAAAAGAGACGATGCACCTCTCGTCCTCCCATCGTACAGCAAGTGA   868
NCPSR-1      CCGCGTCCAGCCTATAAAAGAGACGATGCACCTCTCGTCCTCCCATCGTACAGCAAGTGA   872
NCPSR-2      CCGCGTCCAGCCTATAAAAGAGACGATGCACCTCTCGTCCTCCCATCGTACAGCAAGTGA   872
NCPSR-3      CCGCGTCCAGCCTATAAAAGAGACGATGCACCTCTCGTCCTCCCATCGTACAGCAAGTGA   873
NHRPSR-1     CCGCGTCCAGCCTATAAAAGAGACGATGCACCTCTCGTCCTCCCATCGTACAGCAAGTGA   872
NHRPSR-2     CCGCGTCCAGCCTATAAAAGAGACGATGCACCTCTCGTCCTCCCATCGTACAGCAAGTGA   873
NHRPSR-3     CCGCGTCCAGCCTATAAAAGAGACGATGCACCTCTCGTCCTCCCATCGTACAGCAAGTGA   873
NHRPSR-4     CCGCGTCCAGCCTATAAAAGAGACGATGCACCTCTCGTCCTCCCATCGTACAGCAAGTGA   872
NHRPSR-5     CCGCGTCCAGCCTATAAAAGAGACGATGCACCTCTCGTCCTCCCATCGTACAGCAAGTGA   873
SWWR-1       CCGCGTCCAGCCTATAAAAGAGACGATGCACCTCTCGTCCTCCCATCGTACAGCAAGTGA   873
SWWR-2       CCGCGTCCAGCCTATAAAAGAGACGATGCACCTCTCGTCCTCCCATCGTACAGCAAGTGA   873
SWWR-3       CCGCGTCCAGCCTATAAAAGAGACGATGCACCTCTCGTCCTCCCATCGTACAGCAAGTGA   872
SWWR-4       CCGCGTCCAGCCTATAAAAGAGACGATGCACCTCTCGTCCTCCCATCGTACAGCAAGTGA   869
WUSR-1       CCGCGTCCAGCCTATAAAAGAGACGATGCACCTCTCGTCCTCCCATCGTACAGCAAGTGA   873
WUSR-2       CCGCGTCCAGCCTATAAAAGAGACGATGCACCTCTCGTCCTCCCATCGTACAGCAAGTGA   872
WWR-1        CCGCGTCCAGCCTATAAAAGAGACGATGCACCTCTCGTCCTCCCATCGTACAGCAAGTGA   873
WWR-2        CCGCGTCCAGCCTATAAAAGAGACGATGCACCTCTCGTCCTCCCATCGTACAGCAAGTGA   872
WWR-3        CCGCGTCCAGCCTATAAAAGAGACGATGCACCTCTCGTCCTCCCATCGTACAGCAAGTGA   873
WWRNC-1      CCGCGTCCAGCCTATAAAAGAGACGATGCACCTCTCGTCCTCCCATCGTACAGCAAGTGA   873
WWRNC-2      CCGCGTCCAGCCTATAAAAGAGACGATGCACCTCTCGTCCTCCCATCGTACAGCAAGTGA   867
WWRNC-3      CCGCGTCCAGCCTATAAAAGAGACGATGCACCTCTCGTCCTCCCATCGTACAGCAAGTGA   873
spelta-1     CCGCGTCCAGCCTATAAAAGAGACGATGCACCTCTCGTCCTCCCATCGTACAGCAAGTGA   873
spelta_6     CCGCGTCCAGCCTATAAAAGAGACGATGCACCTCTCGTCCTCCCATCGTACAGCAAGTGA   873
spelta_7     CCGCGTCCAGCCTATAAAAGAGACGATGCACCTCTCGTCCTCCCATCGTACAGCAAGTGA   873
spelta_11    CCGCGTCCAGCCTATAAAAGAGACGATGCACCTCTCGTCCTCCCATCGTACAGCAAGTGA   873
SWRNC-1      CCGCGTCCAGCCTATAAAAGAGACGATGCACCTCTCGTCCTCCCATCGTACAGCAAGTGA   893
SWRNC-2      CCGCGTCCAGCCTATAAAAGAGACGATGCACCTCTCGTCCTCCCATCGTACAGCAAGTGA   899

IWVF-1       AGCTCACACAACCTACCCTACCCACCCATCCATCAGCAGTTTTTCTATCGACCAATGGCT   933
IWVF-2       AGCTCACACAACCTACCCTACCCACCCATCCATCAGCAGTTTTTCTATCGACCAATGGCT   933
IWVF-3       AGCTCACACAACCTACCCTACCCACCCATCCATCAGCAGTTTTTCTATCGACCAATGGCT   932
JUSR         AGCTCACACAACCTACCCTACCCACCCATCCATCAGCAGTTTTTCTATCGACCAATGGCT   928
NCPSR-1      AGCTCACACAACCTACCCTACCCACCCATCCATCAGCAGTTTTTCTATCGACCAATGGCT   932
NCPSR-2      AGCTCACACAACCTACCCTACCCACCCATCCATCAGCAGTTTTTCTATCGACCAATGGCT   932
NCPSR-3      AGCTCACACAACCTACCCTACCCACCCATCCATCAGCAGTTTTTCTATCGACCAATGGCT   933
NHRPSR-1     AGCTCACACAACCTACCCTACCCACCCATCCATCAGCAGTTTTTCTATCGACCAATGGCT   932
NHRPSR-2     AGCTCACACAACCTACCCTACCCACCCATCCATCAGCAGTTTTTCTATCGACCAATGGCT   933
NHRPSR-3     AGCTCACACAACCTACCCTACCCACCCATCCATCAGCAGTTTTTCTATCGACCAATGGCT   933
NHRPSR-4     AGCTCACACAACCTACCCTACCCACCCATCCATCAGCAGTTTTTCTATCGACCAATGGCT   932
NHRPSR-5     AGCTCACACAACCTACCCTACCCACCCATCCATCAGCAGTTTTTCTATCGACCAATGGCT   933
SWWR-1       AGCTCACACAACCTACCCTACCCACCCATCCATCAGCAGTTTTTCTATCGACCAATGGCT   933
SWWR-2       AGCTCACACAACCTACCCTACCCACCCATCCATCAGCAGTTTTTCTATCGACCAATGGCT   933
SWWR-3       AGCTCACACAACCTACCCTACCCACCCATCCATCAGCAGTTTTTCTATCGACCAATGGCT   932
SWWR-4       AGCTCACACAACCTACCCTACCCACCCATCCATCAGCAGTTTTTCTATCGACCAATGGCT   929
WUSR-1       AGCTCACACAACCTACCCTACCCACCCATCCATCAGCAGTTTTTCTATCGACCAATGGCT   933
WUSR-2       AGCTCACACAACCTACCCTACCCACCCATCCATCAGCAGTTTTTCTATCGACCAATGGCT   932
WWR-1        AGCTCACACAACCTACCCTACCCACCCATCCATCAGCAGTTTTTCTATCGACCAATGGCT   933
WWR-2        AGCTCACACAACCTACCCTACCCACCCATCCATCAGCAGTTTTTCTATCGACCAATGGCT   932
WWR-3        AGCTCACACAACCTACCCTACCCACCCATCCATCAGCAGTTTTTCTATCGACCAATGGCT   933
WWRNC-1      AGCTCACACAACCTACCCTACCCACCCATCCATCAGCAGTTTTTCTATCGACCAATGGCT   933
WWRNC-2      AGCTCACACAACCTACCCTACCCACCCATCCATCAGCAGTTTTTCTATCGACCAATGGCT   927
WWRNC-3      AGCTCACACAACCTACCCTACCCACCCATCCATCAGCAGTTTTTCTATCGACCAATGGCT   933
spelta-1     AGCTCACACAACCTACCCTACCCACCCATCCATCAGCAGTTTTTCTATCGACCAATGGCT   933
spelta_6     AGCTCACACAACCTACCCTACCCACCCATCCATCAGCAGTTTTTCTATCGACCAATGGCT   933
spelta_7     AGCTCACACAACCTACCCTACCCACCCATCCATCAGCAGTTTTTCTATCGACCAATGGCT   933
spelta_11    AGCTCACACAACCTACCCTACCCACCCATCCATCAGCAGTTTTTCTATCGACCAATGGCT   933
SWRNC-1      AGCTCACACAACCTACCCTACCCACCCATCCATCAGCAGTTTTTCTATCGACCAATGGCT   953
SWRNC-2      AGCTCACACAACCTACCCTACCCACCCATCCATCAGCAGTTTTTCTATCGACCAATGGCT   959
```

```
IWVF-1       TCTTCTTCCGTGCTGCTCGGAGCCTCGGCCACGGCCGCGCTCACCGGCACCCCGGCAGGC   993
IWVF-2       TCTTCTTCCGTGCTGCTCGGAGCCTCGGCCACGGCCGCGCTCACCGGCACCCCGGCAGGC   993
IWVF-3       TCTTCTTCCGTGCTGCTCGGAGCCTCGGCCACGGCCGCGCTCACCGGCACCCCGGCAGGC   992
JUSR         TCTTCTTCCGTGCTGCTCGGAGCCTCGGCCACGGCCGCGCTCACCGGCACCCCGGCAGGC   988
NCPSR-1      TCTTCTTCCGTGCTGCTCGGAGCCTCGGCCACGGCCGCGCTCACCGGCACCCCGGCAGGC   992
NCPSR-2      TCTTCTTCCGTGCTGCTCGGAGCCTCGGCCACGGCCGCGCTCACCGGCACCCCGGCAGGC   992
NCPSR-3      TCTTCTTCCGTGCTGCTCGGAGCCTCGGCCACGGCCGCGCTCACCGGCACCCCGGCAGGC   993
NHRPSR-1     TCTTCTTCCGTGCTGCTCGGAGCCTCGGCCACGGCCGCGCTCACCGGCACCCCGGCAGGC   992
NHRPSR-2     TCTTCTTCCGTGCTGCTCGGAGCCTCGGCCACGGCCGCGCTCACCGGCACCCCGGCAGGC   993
NHRPSR-3     TCTTCTTCCGTGCTGCTCGGAGCCTCGGCCACGGCCGCGCTCACCGGCACCCCGGCAGGC   993
NHRPSR-4     TCTTCTTCCGTGCTGCTCGGAGCCTCGGCCACGGCCGCGCTCACCGGCACCCCGGCAGGC   992
NHRPSR-5     TCTTCTTCCGTGCTGCTCGGAGCCTCGGCCACGGCCGCGCTCACCGGCACCCCGGCAGGC   993
SWWR-1       TCTTCTTCCGTGCTGCTCGGAGCCTCGGCCACGGCCGCGCTCACCGGCACCCCGGCAGGC   993
SWWR-2       TCTTCTTCCGTGCTGCTCGGAGCCTCGGCCACGGCCGCGCTCACCGGCACCCCGGCAGGC   993
SWWR-3       TCTTCTTCCGTGCTGCTCGGAGCCTCGGCCACGGCCGCGCTCACCGGCACCCCGGCAGGC   992
SWWR-4       TCTTCTTCCGTGCTGCTCGGAGCCTCGGCCACGGCCGCGCTCACCGGCACCCCGGCAGGC   989
WUSR-1       TCTTCTTCCGTGCTGCTCGGAGCCTCGGCCACGGCCGCGCTCACCGGCACCCCGGCAGGC   993
WUSR-2       TCTTCTTCCGTGCTGCTCGGAGCCTCGGCCACGGCCGCGCTCACCGGCACCCCGGCAGGC   992
WWR-1        TCTTCTTCCGTGCTGCTCGGAGCCTCGGCCACGGCCGCGCTCACCGGCACCCCGGCAGGC   993
WWR-2        TCTTCTTCCGTGCTGCTCGGAGCCTCGGCCACGGCCGCGCTCACCGGCACCCCGGCAGGC   992
WWR-3        TCTTCTTCCGTGCTGCTCGGAGCCTCGGCCACGGCCGCGCTCACCGGCACCCCGGCAGGC   993
WWRNC-1      TCTTCTTCCGTGCTGCTCGGAGCCTCGGCCACGGCCGCGCTCACCGGCACCCCGGCAGGC   993
WWRNC-2      TCTTCTTCCGTGCTGCTCGGAGCCTCGGCCACGGCCGCGCTCACCGGCACCCCGGCAGGC   987
WWRNC-3      TCTTCTTCCGTGCTGCTCGGAGCCTCGGCCACGGCCGCGCTCACCGGCACCCCGGCAGGC   993
spelta-1     TCTTCTTCCGTGCTGCTCGGAGCCTCGGCCACGGCCGCGCTCACCGGCACCCCGGCAGGC   993
spelta_6     TCTTCTTCCGTGCTGCTCGGAGCCTCGGCCACGGCCGCGCTCACCGGCACCCCGGCAGGC   993
spelta_7     TCTTCTTCCGTGCTGCTCGGAGCCTCGGCCACGGCCGCGCTCACCGGCACCCCGGCAGGC   993
spelta_11    TCTTCTTCCGTGCTGCTCGGAGCCTCGGCCACGGCCGCGCTCACCGGCACCCCGGCAGGC   993
SWRNC-1      TCTTCTTCCGTGCTGCTCGGAGCCTCGGCCACGGCCGCGCTCACCGGCACCCCGGCAGGC   1013
SWRNC-2      TCTTCTTCCGTGCTGCTCGGAGCCTCGGCCACGGCCGCGCTCACCGGCACCCCGGCAGGC   1019

IWVF-1       AAGGCCCTTCCCCGGCCTTGCTTCCTCGCCGCTCGCCCGCGCACCGTGAGCGGTGGCCGT   1053
IWVF-2       AAGGCCCTTCCCCGGCCTTGCTTCCTCGCCGCTCGCCCGCGCACCGTGAGCGGTGGCCGT   1053
IWVF-3       AAGGCCCTTCCCCGGCCTTGCTTCCTCGCCGCTCGCCCGCGCACCGTGAGCGGTGGCCGT   1052
JUSR         AAGGCCCTTCCCCGGCCTTGCTTCCTCGCCGCTCGCCCGCGCACCGTGAGCGGTGGCCGT   1048
NCPSR-1      AAGGCCCTTCCCCGGCCTTGCTTCCTCGCCGCTCGCCCGCGCACCGTGAGCGGTGGCCGT   1052
NCPSR-2      AAGGCCCTTCCCCGGCCTTGCTTCCTCGCCGCTCGCCCGCGCACCGTGAGCGGTGGCCGT   1052
NCPSR-3      AAGGCCCTTCCCCGGCCTTGCTTCCTCGCCGCTCGCCCGCGCACCGTGAGCGGTGGCCGT   1053
NHRPSR-1     AAGGCCCTTCCCCGGCCTTGCTTCCTCGCCGCTCGCCCGCGCACCGTGAGCGGTGGCCGT   1052
NHRPSR-2     AAGGCCCTTCCCCGGCCTTGCTTCCTCGCCGCTCGCCCGCGCACCGTGAGCGGTGGCCGT   1053
NHRPSR-3     AAGGCCCTTCCCCGGCCTTGCTTCCTCGCCGCTCGCCCGCGCACCGTGAGCGGTGGCCGT   1053
NHRPSR-4     AAGGCCCTTCCCCGGCCTTGCTTCCTCGCCGCTCGCCCGCGCACCGTGAGCGGTGGCCGT   1052
NHRPSR-5     AAGGCCCTTCCCCGGCCTTGCTTCCTCGCCGCTCGCCCGCGCACCGTGAGCGGTGGCCGT   1053
SWWR-1       AAGGCCCTTCCCCGGCCTTGCTTCCTCGCCGCTCGCCCGCGCACCGTGAGCGGTGGCCGT   1053
SWWR-2       AAGGCCCTTCCCCGGCCTTGCTTCCTCGCCGCTCGCCCGCGCACCGTGAGCGGTGGCCGT   1053
SWWR-3       AAGGCCCTTCCCCGGCCTTGCTTCCTCGCCGCTCGCCCGCGCACCGTGAGCGGTGGCCGT   1052
SWWR-4       AAGGCCCTTCCCCGGCCTTGCTTCCTCGCCGCTCGCCCGCGCACCGTGAGCGGTGGCCGT   1049
WUSR-1       AAGGCCCTTCCCCGGCCTTGCTTCCTCGCCGCTCGCCCGCGCACCGTGAGCGGTGGCCGT   1053
WUSR-2       AAGGCCCTTCCCCGGCCTTGCTTCCTCGCCGCTCGCCCGCGCACCGTGAGCGGTGGCCGT   1052
WWR-1        AAGGCCCTTCCCCGGCCTTGCTTCCTCGCCGCTCGCCCGCGCACCGTGAGCGGTGGCCGT   1053
WWR-2        AAGGCCCTTCCCCGGCCTTGCTTCCTCGCCGCTCGCCCGCGCACCGTGAGCGGTGGCCGT   1052
WWR-3        AAGGCCCTTCCCCGGCCTTGCTTCCTCGCCGCTCGCCCGCGCACCGTGAGCGGTGGCCGT   1053
WWRNC-1      AAGGCCCTTCCCCGGCCTTGCTTCCTCGCCGCTCGCCCGCGCACCGTGAGCGGTGGCCGT   1053
WWRNC-2      AAGGCCCTTCCCCGGCCTTGCTTCCTCGCCGCTCGCCCGCGCACCGTGAGCGGTGGCCGT   1047
WWRNC-3      AAGGCCCTTCCCCGGCCTTGCTTCCTCGCCGCTCGCCCGCGCACCGTGAGCGGTGGCCGT   1053
spelta-1     AAGGCCCTTCCCCGGCCTTGCTTCCTCGCCGCTCGCCCGCGCACCGTGAGCGGTGGCCGT   1053
spelta_6     AAGGCCCTTCCCCGGCCTTGCTTCCTCGCCGCTCGCCCGCGCACCGTGAGCGGTGGCCGT   1053
spelta_7     AAGGCCCTTCCCCGGCCTTGCTTCCTCGCCGCTCGCCCGCGCACCGTGAGCGGTGGCCGT   1053
spelta_11    AAGGCCCTTCCCCGGCCTTGCTTCCTCGCCGCTCGCCCGCGCACCGTGAGCGGTGGCCGT   1053
SWRNC-1      AAGGCCCTTCCCCGGCCTTGCTTCCTCGCCGCTCGCCCGCGCACCGTGAGCGGTGGCCGT   1073
SWRNC-2      AAGGCCCTTCCCCGGCCTTGCTTCCTCGCCGCTCGCCCGCGCACCGTGAGCGGTGGCCGT   1079
```

```
IWVF-1       CTCTGCCTGCAGAACGCTCCAAGGGCGACTCCGGTACGTGT.TCGCGAGCGGTACCGTTC   1112
IWVF-2       CTCTGCCTGCAGAACGCTCCAAGGGCGACTCCGGTACGTGTGTCGCGAGCGGTACCGTTC   1113
IWVF-3       CTCTGCCTGCAGAACGCTCCAAGGGCGACTCCGGTACGTGTGTCGCGAGCGGTACCGTTC   1112
JUSR         CTCTGCCTGCAGAACGCTCCAAGGGCGACTCCGGTACGTGTGTCGCGAGCGGTACCGTTC   1108
NCPSR-1      CTCTGCCTGCAGAACGCTCCAAGGGCGACTCCGGTACGTGTGTCGCGAGCGGTACCGTTC   1112
NCPSR-2      CTCTGCCTGCAGAACGCTCCAAGGGCGACTCCGGTACGTGTGTCGCGAGCGGTACCGTTC   1112
NCPSR-3      CTCTGCCTGCAGAACGCTCCAAGGGCGACTCCGGTACGTGTGTCGCGAGCGGTACCGTTC   1113
NHRPSR-1     CTCTGCCTGCAGAACGCTCCAAGGGCGACTCCGGTACGTGTGTCGCGAGCGGTACCGTTC   1112
NHRPSR-2     CTCTGCCTGCAGAACGCTCCAAGGGCGACTCCGGTACGTGT.TCGCGAGCGGTACCGTTC   1112
NHRPSR-3     CTCTGCCTGCAGAACGCTCCAAGGGCGACTCCGGTACGTGT.TCGCGAGCGGTACCGTTC   1112
NHRPSR-4     CTCTGCCTGCAGAACGCTCCAAGGGCGACTCCGGTACGTGTGTCGCGAGCGGTACCGTTC   1112
NHRPSR-5     CTCTGCCTGCAGAACGCTCCAAGGGCGACTCCGGTACGTGTGTCGCGAGCGGTACCGTTC   1113
SWWR-1       CTCTGCCTGCAGAACGCTCCAAGGGCGACTCCGGTACGTGTGTCGCGAGCGGTACCGTTC   1113
SWWR-2       CTCTGCCTGCAGAACGCTCCAAGGGCGACTCCGGTACGTGTGTCGCGAGCGGTACCGTTC   1113
SWWR-3       CTCTGCCTGCAGAACGCTCCAAGGGCGACTCCGGTACGTGTGTCGCGAGCGGTACCGTTC   1112
SWWR-4       CTCTGCCTGCAGAACGCTCCAAGGGCGACTCCGGTACGTGTGTCGCGAGCGGTACCGTTC   1109
WUSR-1       CTCTGCCTGCAGAACGCTCCAAGGGCGACTCCGGTACGTGTGTCGCGAGCGGTACCGTTC   1113
WUSR-2       CTCTGCCTGCAGAACGCTCCAAGGGCGACTCCGGTACGTGTGTCGCGAGCGGTACCGTTC   1112
WWR-1        CTCTGCCTGCAGAACGCTCCAAGGGCGACTCCGGTACGTGTGTCGCGAGCGGTACCGTTC   1113
WWR-2        CTCTGCCTGCAGAACGCTCCAAGGGCGACTCCGGTACGTGTGTCGCGAGCGGTACCGTTC   1112
WWR-3        CTCTGCCTGCAGAACGCTCCAAGGGCGACTCCGGTACGTGT.TCGCGAGCGGTACCGTTC   1112
WWRNC-1      CTCTGCCTGCAGAACGCTCCAAGGGCGACTCCGGTACGTGTGTCGCGAGCGGTACCGTTC   1113
WWRNC-2      CTCTGCCTGCAGAACGCTCCAAGGGCGACTCCGGTACGTGTGTCGCGAGCGGTACCGTTC   1107
WWRNC-3      CTCTGCCTGCAGAACGCTCCAAGGGCGACTCCGGTACGTGT.TCGCGAGCGGTACCGTTC   1112
spelta-1     CTCTGCCTGCAGAACGCTCCAAGGGCGACTCCGGTACGTGTGTCGCGAGCGGTACCGTTC   1113
spelta_6     CTCTGCCTGCAGAACGCTCCAAGGGCGACTCCGGTACGTGT.TCGCGAGCGGTACCGTTC   1112
spelta_7     CTCTGCCTGCAGAACGCTCCAAGGGCGACTCCGGTACGTGT.TCGCGAGCGGTACCGTTC   1112
spelta_11    CTCTGCCTGCAGAACGCTCCAAGGGCGACTCCGGTACGTGT.TCGCGAGCGGTACCGTTC   1112
SWRNC-1      CTCTGCCTGCAGAACGCTCCAAGGGCGACTCCGGTACGTGTGTCGCGAGCGGTACCGTTC   1133
SWRNC-2      CTCTGCCTGCAGAACGCTCCAAGGGCGACTCCGGTACGTGTGTCGCGAGCGGTACCGTTC   1139

IWVF-1       GATCATGACTTCATGCCGATTGTTTTACCTACGCTGCTGGTTAATCAATCGTTCCATGGG   1172
IWVF-2       GATCATGACTTCATGCCGATTGTTTTACCTACGCTGCTGGTTAATCAATCGTTCCATGGG   1173
IWVF-3       GATCATGACTTCATGCCGATTGTTTTACCTACGCTGCTGGTTAATCAATCGTTCCATGGG   1172
JUSR         GATCATGACTTCATGCCGATTGTTTTACCTACGCTGCTGGTTAATCAATCGTTCCATGGG   1168
NCPSR-1      GATCATGACTTCATGCCGATTGTTTTACCTACGCTGCTGGTTAATCAATCGTTCCATGGG   1172
NCPSR-2      GATCATGACTTCATGCCGATTGTTTTACCTACGCTGCTGGTTAATCAATCGTTCCATGGG   1172
NCPSR-3      GATCATGACTTCATGCCGATTGTTTTACCTACGCTGCTGGTTAATCAATCGTTCCATGGG   1173
NHRPSR-1     GATCATGACTTCATGCCGATTGTTTTACCTACGCTGCTGGTTAATCAATCGTTCCATGGG   1172
NHRPSR-2     GATCATGACTTCATGCCGATTGTTTTACCTACGCTGCTGGTTAATCAATCGTTCCATGGG   1172
NHRPSR-3     GATCATGACTTCATGCCGATTGTTTTACCTACGCTGCTGGTTAATCAATCGTTCCATGGG   1172
NHRPSR-4     GATCATGACTTCATGCCGATTGTTTTACCTACGCTGCTGGTTAATCAATCGTTCCATGGG   1172
NHRPSR-5     GATCATGACTTCATGCCGATTGTTTTACCTACGCTGCTGGTTAATCAATCGTTCCATGGG   1173
SWWR-1       GATCATGACTTCATGCCGATTGTTTTACCTACGCTGCTGGTTAATCAATCGTTCCATGGG   1173
SWWR-2       GATCATGACTTCATGCCGATTGTTTTACCTACGCTGCTGGTTAATCAATCGTTCCATGGG   1173
SWWR-3       GATCATGACTTCATGCCGATTGTTTTACCTACGCTGCTGGTTAATCAATCGTTCCATGGG   1172
SWWR-4       GATCATGACTTCATGCCGATTGTTTTACCTACGCTGCTGGTTAATCAATCGTTCCATGGG   1169
WUSR-1       GATCATGACTTCATGCCGATTGTTTTACCTACGCTGCTGGTTAATCAATCGTTCCATGGG   1173
WUSR-2       GATCATGACTTCATGCCGATTGTTTTACCTACGCTGCTGGTTAATCAATCGTTCCATGGG   1172
WWR-1        GATCATGACTTCATGCCGATTGTTTTACCTACGCTGCTGGTTAATCAATCGTTCCATGGG   1173
WWR-2        GATCATGACTTCATGCCGATTGTTTTACCTACGCTGCTGGTTAATCAATCGTTCCATGGG   1172
WWR-3        GATCATGACTTCATGCCGATTGTTTTACCTACGCTGCTGGTTAATCAATCGTTCCATGGG   1172
WWRNC-1      GATCATGACTTCATGCCGATTGTTTTACCTACGCTGCTGGTTAATCAATCGTTCCATGGG   1173
WWRNC-2      GATCATGACTTCATGCCGATTGTTTTACCTACGCTGCTGGTTAATCAATCGTTCCATGGG   1167
WWRNC-3      GATCATGACTTCATGCCGATTGTTTTACCTACGCTGCTGGTTAATCAATCGTTCCATGGG   1172
spelta-1     GATCATGACTTCATGCCGATTGTTTTACCTACGCTGCTGGTTAATCAATCGTTCCATGGG   1173
spelta_6     GATCATGACTTCATGCCGATTGTTTTACCTACGCTGCTGGTTAATCAATCGTTCCATGGG   1172
spelta_7     GATCATGACTTCATGCCGATTGTTTTACCTACGCTGCTGGTTAATCAATCGTTCCATGGG   1172
spelta_11    GATCATGACTTCATGCCGATTGTTTTACCTACGCTGCTGGTTAATCAATCGTTCCATGGG   1172
SWRNC-1      GATCATGACTTCATGCCGATTGTTTTACCTACGCTGCTGGTTAATCAATCGTTCCATGGG   1193
SWRNC-2      GATCATGACTTCATGCCGATTGTTTTACCTACGCTGCTGGTTAATCAATCGTTCCATGGG   1199
```

```
IWVF-1       ATATGCTGACCGAGATGTATGCGTGCATGGGACAGGCGTACAACGACGCTGCGGATGCCA   1232
IWVF-2       ATATGCTGACCGAGATGTATGCGTGCATGGGACAGGCGTACAACGACGCTGCGGATGCCA   1233
IWVF-3       ATATGCTGACCGAGATGTATGCGTGCATGGGACAGGCGTACAACGACGCTGCGGATGCCA   1232
JUSR         ATATGCTGACCGAGATGTATGCGTGCATGGGACAGGCGTACAACGACGCTGCGGATGCCA   1228
NCPSR-1      ATATGCTGACCGAGATGTATGCGTGCATGGGACAGGCGTACAACGACGCTGCGGATGCCA   1232
NCPSR-2      ATATGCTGACCGAGATGTATGCGTGCATGGGACAGGCGTACAACGACGCTGCGGATGCCA   1232
NCPSR-3      ATATGCTGACCGAGATGTATGCGTGCATGGGACAGGCGTACAACGACGCTGCGGATGCCA   1233
NHRPSR-1     ATATGCTGACCGAGATGTATGCGTGCATGGGACAGGCGTACAACGACGCTGCGGATGCCA   1232
NHRPSR-2     ATATGCTGACCGAGATGTATGCGTGCATGGGACAGGCGTACAACGACGCTGCGGATGCCA   1232
NHRPSR-3     ATATGCTGACCGAGATGTATGCGTGCATGGGACAGGCGTACAACGACGCTGCGGATGCCA   1232
NHRPSR-4     ATATGCTGACCGAGATGTATGCGTGCATGGGACAGGCGTACAACGACGCTGCGGATGCCA   1232
NHRPSR-5     ATATGCTGACCGAGATGTATGCGTGCATGGGACAGGCGTACAACGACGCTGCGGATGCCA   1233
SWWR-1       ATATGCTGACCGAGATGTATGCGTGCATGGGACAGGCGTACAACGACGCTGCGGATGCCA   1233
SWWR-2       ATATGCTGACCGAGATGTATGCGTGCATGGGACAGGCGTACAACGACGCTGCGGATGCCA   1233
SWWR-3       ATATGCTGACCGAGATGTATGCGTGCATGGGACAGGCGTACAACGACGCTGCGGATGCCA   1232
SWWR-4       ATATGCTGACCGAGATGTATGCGTGCATGGGACAGGCGTACAACGACGCTGCGGATGCCA   1229
WUSR-1       ATATGCTGACCGAGATGTATGCGTGCATGGGACAGGCGTACAACGACGCTGCGGATGCCA   1233
WUSR-2       ATATGCTGACCGAGATGTATGCGTGCATGGGACAGGCGTACAACGACGCTGCGGATGCCA   1232
WWR-1        ATATGCTGACCGAGATGTATGCGTGCATGGGACAGGCGTACAACGACGCTGCGGATGCCA   1233
WWR-2        ATATGCTGACCGAGATGTATGCGTGCATGGGACAGGCGTACAACGACGCTGCGGATGCCA   1232
WWR-3        ATATGCTGACCGAGATGTATGCGTGCATGGGACAGGCGTACAACGACGCTGCGGATGCCA   1232
WWRNC-1      ATATGCTGACCGAGATGTATGCGTGCATGGGACAGGCGTACAACGACGCTGCGGATGCCA   1233
WWRNC-2      ATATGCTGACCGAGATGTATGCGTGCATGGGACAGGCGTACAACGACGCTGCGGATGCCA   1227
WWRNC-3      ATATGCTGACCGAGATGTATGCGTGCATGGGACAGGCGTACAACGACGCTGCGGATGCCA   1232
spelta-1     ATATGCTGACCGAGATGTATGCGTGCATGGGACAGGCGTACAACGACGCTGCGGATGCCA   1233
spelta_6     ATATGCTGACCGAGATGTATGCGTGCATGGGACAGGCGTACAACGACGCTGCGGATGCCA   1232
spelta_7     ATATGCTGACCGAGATGTATGCGTGCATGGGACAGGCGTACAACGACGCTGCGGATGCCA   1232
spelta_11    ATATGCTGACCGAGATGTATGCGTGCATGGGACAGGCGTACAACGACGCTGCGGATGCCA   1232
SWRNC-1      ATATGCTGACCGAGATGTATGCGTGCATGGGACAGGCGTACAACGACGCTGCGGATGCCA   1253
SWRNC-2      ATATGCTGACCGAGATGTATGCGTGCATGGGACAGGCGTACAACGACGCTGCGGATGCCA   1259

IWVF-1       CCGACAAGGCCATCGACGGCGTGAAGGGGGTGGCCGACGAGTTGAAGAAGGGCGTGGCGG   1292
IWVF-2       CCGACAAGGCCATCGACGGCGTGAAGGGGGTGGCCGACGAGTTGAAGAAGGGCGTGGCGG   1293
IWVF-3       CCGACAAGGCCATCGACGGCGTGAAGGGGGTGGCCGACGAGTTGAAGAAGGGCGTGGCGG   1292
JUSR         CCGACAAGGCCATCGACGGCGTGAAGGGGGTGGCCGACGAGTTGAAGAAGGGCGTGGCGG   1288
NCPSR-1      CCGACAAGGCCATCGACGGCGTGAAGGGGGTGGCCGACGAGTTGAAGAAGGGCGTGGCGG   1292
NCPSR-2      CCGACAAGGCCATCGACGGCGTGAAGGGGGTGGCCGACGAGTTGAAGAAGGGCGTGGCGG   1292
NCPSR-3      CCGACAAGGCCATCGACGGCGTGAAGGGGGTGGCCGACGAGTTGAAGAAGGGCGTGGCGG   1293
NHRPSR-1     CCGACAAGGCCATCGACGGCGTGAAGGGGGTGGCCGACGAGTTGAAGAAGGGCGTGGCGG   1292
NHRPSR-2     CCGACAAGGCCATCGACGGCGTGAAGGGGGTGGCCGACGAGTTGAAGAAGGGCGTGGCGG   1292
NHRPSR-3     CCGACAAGGCCATCGACGGCGTGAAGGGGGTGGCCGACGAGTTGAAGAAGGGCGTGGCGG   1292
NHRPSR-4     CCGACAAGGCCATCGACGGCGTGAAGGGGGTGGCCGACGAGTTGAAGAAGGGCGTGGCGG   1292
NHRPSR-5     CCGACAAGGCCATCGACGGCGTGAAGGGGGTGGCCGACGAGTTGAAGAAGGGCGTGGCGG   1293
SWWR-1       CCGACAAGGCCATCGACGGCGTGAAGGGGGTGGCCGACGAGTTGAAGAAGGGCGTGGCGG   1293
SWWR-2       CCGACAAGGCCATCGACGGCGTGAAGGGGGTGGCCGACGAGTTGAAGAAGGGCGTGGCGG   1293
SWWR-3       CCGACAAGGCCATCGACGGCGTGAAGGGGGTGGCCGACGAGTTGAAGAAGGGCGTGGCGG   1292
SWWR-4       CCGACAAGGCCATCGACGGCGTGAAGGGGGTGGCCGACGAGTTGAAGAAGGGCGTGGCGG   1289
WUSR-1       CCGACAAGGCCATCGACGGCGTGAAGGGGGTGGCCGACGAGTTGAAGAAGGGCGTGGCGG   1293
WUSR-2       CCGACAAGGCCATCGACGGCGTGAAGGGGGTGGCCGACGAGTTGAAGAAGGGCGTGGCGG   1292
WWR-1        CCGACAAGGCCATCGACGGCGTGAAGGGGGTGGCCGACGAGTTGAAGAAGGGCGTGGCGG   1293
WWR-2        CCGACAAGGCCATCGACGGCGTGAAGGGGGTGGCCGACGAGTTGAAGAAGGGCGTGGCGG   1292
WWR-3        CCGACAAGGCCATCGACGGCGTGAAGGGGGTGGCCGACGAGTTGAAGAAGGGCGTGGCGG   1292
WWRNC-1      CCGACAAGGCCATCGACGGCGTGAAGGGGGTGGCCGACGAGTTGAAGAAGGGCGTGGCGG   1293
WWRNC-2      CCGACAAGGCCATCGACGGCGTGAAGGGGGTGGCCGACGAGTTGAAGAAGGGCGTGGCGG   1287
WWRNC-3      CCGACAAGGCCATCGACGGCGTGAAGGGGGTGGCCGACGAGTTGAAGAAGGGCGTGGCGG   1292
spelta-1     CCGACAAGGCCATCGACGGCGTGAAGGGGGTGGCCGACGAGTTGAAGAAGGGCGTGGCGG   1293
spelta_6     CCGACAAGGCCATCGACGGCGTGAAGGGGGTGGCCGACGAGTTGAAGAAGGGCGTGGCGG   1292
spelta_7     CCGACAAGGCCATCGACGGCGTGAAGGGGGTGGCCGACGAGTTGAAGAAGGGCGTGGCGG   1292
spelta_11    CCGACAAGGCCATCGACGGCGTGAAGGGGGTGGCCGACGAGTTGAAGAAGGGCGTGGCGG   1292
SWRNC-1      CCGACAAGGCCATCGACGGCGTGAAGGGGGTGGCCGACGAGTTGAAGAAGGGCGTGGCGG   1313
SWRNC-2      CCGACAAGGCCATCGACGGCGTGAAGGGGGTGGCCGACGAGTTGAAGAAGGGCGTGGCGG   1319
```

```
IWVF-1        AGGCTGCGGAGGCCGTCTCGGGCAACACCGAGAAGGCCGCGGAGGAAGCCGGCAAGGGCG   1352
IWVF-2        AGGCTGCGGAGGCCGTCTCGGGCAACACCGAGAAGGCCGCGGAGGAAGCCGGCAAGGGCG   1353
IWVF-3        AGGCTGCGGAGGCCGTCTCGGGCAACACCGAGAAGGCCGCGGAGGAAGCCGGCAAGGGCG   1352
JUSR          AGGCTGCGGAGGCCGTCTCGGGCAACACCGAGAAGGCCGCGGAGGAAGCCGGCAAGGGCG   1348
NCPSR-1       AGGCTGCGGAGGCCGTCTCGGGCAACACCGAGAAGGCCGCGGAGGAAGCCGGCAAGGGCG   1352
NCPSR-2       AGGCTGCGGAGGCCGTCTCGGGCAACACCGAGAAGGCCGCGGAGGAAGCCGGCAAGGGCG   1352
NCPSR-3       AGGCTGCGGAGGCCGTCTCGGGCAACACCGAGAAGGCCGCGGAGGAAGCCGGCAAGGGCG   1353
NHRPSR-1      AGGCTGCGGAGGCCGTCTCGGGCAACACCGAGAAGGCCGCGGAGGAAGCCGGCAAGGGCG   1352
NHRPSR-2      AGGCTGCGGAGGCCGTCTCGGGCAACACCGAGAAGGCCGCGGAGGAAGCCGGCAAGGGCG   1352
NHRPSR-3      AGGCTGCGGAGGCCGTCTCGGGCAACACCGAGAAGGCCGCGGAGGAAGCCGGCAAGGGCG   1352
NHRPSR-4      AGGCTGCGGAGGCCGTCTCGGGCAACACCGAGAAGGCCGCGGAGGAAGCCGGCAAGGGCG   1352
NHRPSR-5      AGGCTGCGGAGGCCGTCTCGGGCAACACCGAGAAGGCCGCGGAGGAAGCCGGCAAGGGCG   1353
SWWR-1        AGGCTGCGGAGGCCGTCTCGGGCAACACCGAGAAGGCCGCGGAGGAAGCCGGCAAGGGCG   1353
SWWR-2        AGGCTGCGGAGGCCGTCTCGGGCAACACCGAGAAGGCCGCGGAGGAAGCCGGCAAGGGCG   1353
SWWR-3        AGGCTGCGGAGGCCGTCTCGGGCAACACCGAGAAGGCCGCGGAGGAAGCCGGCAAGGGCG   1352
SWWR-4        AGGCTGCGGAGGCCGTCTCGGGCAACACCGAGAAGGCCGCGGAGGAAGCCGGCAAGGGCG   1349
WUSR-1        AGGCTGCGGAGGCCGTCTCGGGCAACACCGAGAAGGCCGCGGAGGAAGCCGGCAAGGGCG   1353
WUSR-2        AGGCTGCGGAGGCCGTCTCGGGCAACACCGAGAAGGCCGCGGAGGAAGCCGGCAAGGGCG   1352
WWR-1         AGGCTGCGGAGGCCGTCTCGGGCAACACCGAGAAGGCCGCGGAGGAAGCCGGCAAGGGCG   1353
WWR-2         AGGCTGCGGAGGCCGTCTCGGGCAACACCGAGAAGGCCGCGGAGGAAGCCGGCAAGGGCG   1352
WWR-3         AGGCTGCGGAGGCCGTCTCGGGCAACACCGAGAAGGCCGCGGAGGAAGCCGGCAAGGGCG   1352
WWRNC-1       AGGCTGCGGAGGCCGTCTCGGGCAACACCGAGAAGGCCGCGGAGGAAGCCGGCAAGGGCG   1353
WWRNC-2       AGGCTGCGGAGGCCGTCTCGGGCAACACCGAGAAGGCCGCGGAGGAAGCCGGCAAGGGCG   1347
WWRNC-3       AGGCTGCGGAGGCCGTCTCGGGCAACACCGAGAAGGCCGCGGAGGAAGCCGGCAAGGGCG   1352
spelta-1      AGGCTGCGGAGGCCGTCTCGGGCAACACCGAGAAGGCCGCGGAGGAAGCCGGCAAGGGCG   1353
spelta_6      AGGCTGCGGAGGCCGTCTCGGGCAACACCGAGAAGGCCGCGGAGGAAGCCGGCAAGGGCG   1352
spelta_7      AGGCTGCGGAGGCCGTCTCGGGCAACACCGAGAAGGCCGCGGAGGAAGCCGGCAAGGGCG   1352
spelta_11     AGGCTGCGGAGGCCGTCTCGGGCAACACCGAGAAGGCCGCGGAGGAAGCCGGCAAGGGCG   1352
SWRNC-1       AGGCTGCGGAGGCCGTCTCGGGCAACACCGAGAAGGCCGCGGAGGAAGCCGGCAAGGGCG   1373
SWRNC-2       AGGCTGCGGAGGCCGTCTCGGGCAACACCGAGAAGGCCGCGGAGGAAGCCGGCAAGGGCG   1379

IWVF-1        CGAGCGAGGTGGACGCGAAGGCCAAGGACTTCGGCGAGCAGGCGAAGAAGGCGACGGAGG   1412
IWVF-2        CGAGCGAGGTGGACGCGAAGGCCAAGGACTTCGGCGAGCAGGCGAAGAAGGCGACGGAGG   1413
IWVF-3        CGAGCGAGGTGGACGCGAAGGCCAAGGACTTCGGCGAGCAGGCGAAGAAGGCGACGGAGG   1412
JUSR          CGAGCGAGGTGGACGCGAAGGCCAAGGACTTCGGCGAGCAGGCGAAGAAGGCGACGGAGG   1408
NCPSR-1       CGAGCGAGGTGGACGCGAAGGCCAAGGACTTCGGCGAGCAGGCGAAGAAGGCGACGGAGG   1412
NCPSR-2       CGAGCGAGGTGGACGCGAAGGCCAAGGACTTCGGCGAGCAGGCGAAGAAGGCGACGGAGG   1412
NCPSR-3       CGAGCGAGGTGGACGCGAAGGCCAAGGACTTCGGCGAGCAGGCGAAGAAGGCGACGGAGG   1413
NHRPSR-1      CGAGCGAGGTGGACGCGAAGGCCAAGGACTTCGGCGAGCAGGCGAAGAAGGCGACGGAGG   1412
NHRPSR-2      CGAGCGAGGTGGACGCGAAGGCCAAGGACTTCGGCGAGCAGGCGAAGAAGGCGACGGAGG   1412
NHRPSR-3      CGAGCGAGGTGGACGCGAAGGCCAAGGACTTCGGCGAGCAGGCGAAGAAGGCGACGGAGG   1412
NHRPSR-4      CGAGCGAGGTGGACGCGAAGGCCAAGGACTTCGGCGAGCAGGCGAAGAAGGCGACGGAGG   1412
NHRPSR-5      CGAGCGAGGTGGACGCGAAGGCCAAGGACTTCGGCGAGCAGGCGAAGAAGGCGACGGAGG   1413
SWWR-1        CGAGCGAGGTGGACGCGAAGGCCAAGGACTTCGGCGAGCAGGCGAAGAAGGCGACGGAGG   1413
SWWR-2        CGAGCGAGGTGGACGCGAAGGCCAAGGACTTCGGCGAGCAGGCGAAGAAGGCGACGGAGG   1413
SWWR-3        CGAGCGAGGTGGACGCGAAGGCCAAGGACTTCGGCGAGCAGGCGAAGAAGGCGACGGAGG   1412
SWWR-4        CGAGCGAGGTGGACGCGAAGGCCAAGGACTTCGGCGAGCAGGCGAAGAAGGCGACGGAGG   1409
WUSR-1        CGAGCGAGGTGGACGCGAAGGCCAAGGACTTCGGCGAGCAGGCGAAGAAGGCGACGGAGG   1413
WUSR-2        CGAGCGAGGTGGACGCGAAGGCCAAGGACTTCGGCGAGCAGGCGAAGAAGGCGACGGAGG   1412
WWR-1         CGAGCGAGGTGGACGCGAAGGCCAAGGACTTCGGCGAGCAGGCGAAGAAGGCGACGGAGG   1413
WWR-2         CGAGCGAGGTGGACGCGAAGGCCAAGGACTTCGGCGAGCAGGCGAAGAAGGCGACGGAGG   1412
WWR-3         CGAGCGAGGTGGACGCGAAGGCCAAGGACTTCGGCGAGCAGGCGAAGAAGGCGACGGAGG   1412
WWRNC-1       CGAGCGAGGTGGACGCGAAGGCCAAGGACTTCGGCGAGCAGGCGAAGAAGGCGACGGAGG   1413
WWRNC-2       CGAGCGAGGTGGACGCGAAGGCCAAGGACTTCGGCGAGCAGGCGAAGAAGGCGACGGAGG   1407
WWRNC-3       CGAGCGAGGTGGACGCGAAGGCCAAGGACTTCGGCGAGCAGGCGAAGAAGGCGACGGAGG   1412
spelta-1      CGAGCGAGGTGGACGCGAAGGCCAAGGACTTCGGCGAGCAGGCGAAGAAGGCGACGGAGG   1413
spelta_6      CGAGCGAGGTGGACGCGAAGGCCAAGGACTTCGGCGAGCAGGCGAAGAAGGCGACGGAGG   1412
spelta_7      CGAGCGAGGTGGACGCGAAGGCCAAGGACTTCGGCGAGCAGGCGAAGAAGGCGACGGAGG   1412
spelta_11     CGAGCGAGGTGGACGCGAAGGCCAAGGACTTCGGCGAGCAGGCGAAGAAGGCGACGGAGG   1412
SWRNC-1       CGAGCGAGGTGGACGCGAAGGCCAAGGACTTCGGCGAGCAGGCGAAGAAGGCGACGGAGG   1433
SWRNC-2       CGAGCGAGGTGGACGCGAAGGCCAAGGACTTCGGCGAGCAGGCGAAGAAGGCGACGGAGG   1439
```

```
IWVF-1      AGGCGTGGGACGGCGCCAAGGACGCCGCACAGGGCATCACGGACAAAGTCGCCGCCGCGG   1472
IWVF-2      AGGCGTGGGACGGCGCCAAGGACGCCGCACAGGGCATCACGGACAAAGTCGCCGCCGCGG   1473
IWVF-3      AGGCGTGGGACGGCGCCAAGGACGCCGCACAGGGCATCACGGACAAAGTCGCCGCCGCGG   1472
JUSR        AGGCGTGGGACGGCGCCAAGGACGCCGCACAGGGCATCACGGACAAAGTCGCCGCCGCGG   1468
NCPSR-1     AGGCGTGGGACGGCGCCAAGGACGCCGCACAGGGCATCACGGACAAAGTCGCCGCCGCGG   1472
NCPSR-2     AGGCGTGGGACGGCGCCAAGGACGCCGCACAGGGCATCACGGACAAAGTCGCCGCCGCGG   1472
NCPSR-3     AGGCGTGGGACGGCGCCAAGGACGCCGCACAGGGCATCACGGACAAAGTCGCCGCCGCGG   1473
NHRPSR-1    AGGCGTGGGACGGCGCCAAGGACGCCGCACAGGGCATCACGGACAAAGTCGCCGCCGCGG   1472
NHRPSR-2    AGGCGTGGGACGGCGCCAAGGACGCCGCACAGGGCATCACGGACAAAGTCGCCGCCGCGG   1472
NHRPSR-3    AGGCGTGGGACGGCGCCAAGGACGCCGCACAGGGCATCACGGACAAAGTCGCCGCCGCGG   1472
NHRPSR-4    AGGCGTGGGACGGCGCCAAGGACGCCGCACAGGGCATCACGGACAAAGTCGCCGCCGCGG   1472
NHRPSR-5    AGGCGTGGGACGGCGCCAAGGACGCCGCACAGGGCATCACGGACAAAGTCGCCGCCGCGG   1473
SWWR-1      AGGCGTGGGACGGCGCCAAGGACGCCGCACAGGGCATCACGGACAAAGTCGCCGCCGCGG   1473
SWWR-2      AGGCGTGGGACGGCGCCAAGGACGCCGCACAGGGCATCACGGACAAAGTCGCCGCCGCGG   1473
SWWR-3      AGGCGTGGGACGGCGCCAAGGACGCCGCACAGGGCATCACGGACAAAGTCGCCGCCGCGG   1472
SWWR-4      AGGCGTGGGACGGCGCCAAGGACGCCGCACAGGGCATCACGGACAAAGTCGCCGCCGCGG   1469
WUSR-1      AGGCGTGGGACGGCGCCAAGGACGCCGCACAGGGCATCACGGACAAAGTCGCCGCCGCGG   1473
WUSR-2      AGGCGTGGGACGGCGCCAAGGACGCCGCACAGGGCATCACGGACAAAGTCGCCGCCGCGG   1472
WWR-1       AGGCGTGGGACGGCGCCAAGGACGCCGCACAGGGCATCACGGACAAAGTCGCCGCCGCGG   1473
WWR-2       AGGCGTGGGACGGCGCCAAGGACGCCGCACAGGGCATCACGGACAAAGTCGCCGCCGCGG   1472
WWR-3       AGGCGTGGGACGGCGCCAAGGACGCCGCACAGGGCATCACGGACAAAGTCGCCGCCGCGG   1472
WWRNC-1     AGGCGTGGGACGGCGCCAAGGACGCCGCACAGGGCATCACGGACAAAGTCGCCGCCGCGG   1473
WWRNC-2     AGGCGTGGGACGGCGCCAAGGACGCCGCACAGGGCATCACGGACAAAGTCGCCGCCGCGG   1467
WWRNC-3     AGGCGTGGGACGGCGCCAAGGACGCCGCACAGGGCATCACGGACAAAGTCGCCGCCGCGG   1472
spelta-1    AGGCGTGGGACGGCGCCAAGGACGCCGCACAGGGCATCACGGACAAAGTCGCCGCCGCGG   1473
spelta_6    AGGCGTGGGACGGCGCCAAGGACGCCGCACAGGGCATCACGGACAAAGTCGCCGCCGCGG   1472
spelta_7    AGGCGTGGGACGGCGCCAAGGACGCCGCACAGGGCATCACGGACAAAGTCGCCGCCGCGG   1472
spelta_11   AGGCGTGGGACGGCGCCAAGGACGCCGCACAGGGCATCACGGACAAAGTCGCCGCCGCGG   1472
SWRNC-1     AGGCGTGGGACGGCGCCAAGGACGCCGCACAGGGCATCACGGACAAAGTCGCCGCCGCGG   1493
SWRNC-2     AGGCGTGGGACGGCGCCAAGGACGCCGCACAGGGCATCACGGACAAAGTCGCCGCCGCGG   1499

IWVF-1      CCAAAAAGGAAGCTAGCTAAGCTAACACTACGTTGACTAGTCCGATCTGTATCGCTCAAT   1532
IWVF-2      CCAAAAAGGAAGCTAGCTAAGCTAACACTACGTTGACTAGTCCGATCTGTATCGCTCAAT   1533
IWVF-3      CCAAAAAGGAAGCTAGCTAAGCTAACACTACGTTGACTAGTCCGATCTGTATCGCTCAAT   1532
JUSR        CCAAAAAGGAAGCTAGCTAAGCTAACACTACGTTGACTAGTCCGATCTGTATCGCTCAAT   1528
NCPSR-1     CCAAAAAGGAAGCTAGCTAAGCTAACACTACGTTGACTAGTCCGATCTGTATCGCTCAAT   1532
NCPSR-2     CCAAAAAGGAAGCTAGCTAAGCTAACACTACGTTGACTAGTCCGATCTGTATCGCTCAAT   1532
NCPSR-3     CCAAAAAGGAAGCTAGCTAAGCTAACACTACGTTGACTAGTCCGATCTGTATCGCTCAAT   1533
NHRPSR-1    CCAAAAAGGAAGCTAGCTAAGCTAACACTACGTTGACTAGTCCGATCTGTATCGCTCAAT   1532
NHRPSR-2    CCAAAAAGGAAGCTAGCTAAGCTAACACTACGTTGACTAGTCCGATCTGTATCGCTCAAT   1532
NHRPSR-3    CCAAAAAGGAAGCTAGCTAAGCTAACACTACGTTGACTAGTCCGATCTGTATCGCTCAAT   1532
NHRPSR-4    CCAAAAAGGAAGCTAGCTAAGCTAACACTACGTTGACTAGTCCGATCTGTATCGCTCAAT   1532
NHRPSR-5    CCAAAAAGGAAGCTAGCTAAGCTAACACTACGTTGACTAGTCCGATCTGTATCGCTCAAT   1533
SWWR-1      CCAAAAAGGAAGCTAGCTAAGCTAACACTACGTTGACTAGTCCGATCTGTATCGCTCAAT   1533
SWWR-2      CCAAAAAGGAAGCTAGCTAAGCTAACACTACGTTGACTAGTCCGATCTGTATCGCTCAAT   1533
SWWR-3      CCAAAAAGGAAGCTAGCTAAGCTAACACTACGTTGACTAGTCCGATCTGTATCGCTCAAT   1532
SWWR-4      CCAAAAAGGAAGCTAGCTAAGCTAACACTACGTTGACTAGTCCGATCTGTATCGCTCAAT   1529
WUSR-1      CCAAAAAGGAAGCTAGCTAAGCTAACACTACGTTGACTAGTCCGATCTGTATCGCTCAAT   1533
WUSR-2      CCAAAAAGGAAGCTAGCTAAGCTAACACTACGTTGACTAGTCCGATCTGTATCGCTCAAT   1532
WWR-1       CCAAAAAGGAAGCTAGCTAAGCTAACACTACGTTGACTAGTCCGATCTGTATCGCTCAAT   1533
WWR-2       CCAAAAAGGAAGCTAGCTAAGCTAACACTACGTTGACTAGTCCGATCTGTATCGCTCAAT   1532
WWR-3       CCAAAAAGGAAGCTAGCTAAGCTAACACTACGTTGACTAGTCCGATCTGTATCGCTCAAT   1532
WWRNC-1     CCAAAAAGGAAGCTAGCTAAGCTAACACTACGTTGACTAGTCCGATCTGTATCGCTCAAT   1533
WWRNC-2     CCAAAAAGGAAGCTAGCTAAGCTAACACTACGTTGACTAGTCCGATCTGTATCGCTCAAT   1527
WWRNC-3     CCAAAAAGGAAGCTAGCTAAGCTAACACTACGTTGACTAGTCCGATCTGTATCGCTCAAT   1532
spelta-1    CCAAAAAGGAAGCTAGCTAAGCTAACACTACGTTGACTAGTCCGATCTGTATCGCTCAAT   1533
spelta_6    CCAAAAAGGAAGCTAGCTAAGCTAACACTACGTTGACTAGTCCGATCTGTATCGCTCAAT   1532
spelta_7    CCAAAAAGGAAGCTAGCTAAGCTAACACTACGTTGACTAGTCCGATCTGTATCGCTCAAT   1532
spelta_11   CCAAAAAGGAAGCTAGCTAAGCTAACACTACGTTGACTAGTCCGATCTGTATCGCTCAAT   1532
SWRNC-1     CCAAAAAGGAAGCTAGCTAAGCTAACACTACGTTGACTAGTCCGATCTGTATCGCTCAAT   1553
SWRNC-2     CCAAAAAGGAAGCTAGCTAAGCTAACACTACGTTGACTAGTCCGATCTGTATCGCTCAAT   1559
```

```
IWVF-1       TCATTTTCCATTGTAAGGAATGCATATACGTACTTCGGTACAAGAGATAAGATAGCTGTA   1592
IWVF-2       TCATTTTCCATTGTAAGGAATGCATATACGTATTTCGGTACAAGAGATAAGATAGCTGTA   1593
IWVF-3       TCATTTTCCATTGTAAGGAATGCATATACGTACTTCGGTACAAGAGATAAGATAGCTGTA   1592
JUSR         TCATTTTCCATTGTAAGGAATGCATATACGTACTTCGGTACAAGAGATAAGATAGCTGTA   1588
NCPSR-1      TCATTTTCCATTGTAAGGAATGCATATACGTACTTCGGTACAAGAGATAAGATAGCTGTA   1592
NCPSR-2      TCATTTTCCATTGTAAGGAATGCATATACGTACTTCGGTACAAGAGATAAGATAGCTGTA   1592
NCPSR-3      TCATTTTCCATTGTAAGGAATGCATATACGTACTTCGGTACAAGAGATAAGATAGCTGTA   1593
NHRPSR-1     TCATTTTCCATTGTAAGGAATGCATATACGTACTTCGGTACAAGAGATAAGATAGCTGTA   1592
NHRPSR-2     TCATTTTCCATTGTAAGGAATGCATATACGTACTTCGGTACAAGAGATAAGATAGCTGTA   1592
NHRPSR-3     TCATTTTCCATTGTAAGGAATGCATATACGTACTTCGGTACAAGAGATAAGATAGCTGTA   1592
NHRPSR-4     TCATTTTCCATTGTAAGGAATGCATATACGTACTTCGGTACAAGAGATAAGATAGCTGTA   1592
NHRPSR-5     TCATTTTCCATTGTAAGGAATGCATATACGTATTTCGGTACAAGAGATAAGATAGCTGTA   1593
SWWR-1       TCATTTTCCATTGTAAGGAATGCATATACGTACTTCGGTACAAGAGATAAGATAGCTGTA   1593
SWWR-2       TCATTTTCCATTGTAAGGAATGCATATACGTATTTCGGTACAAGAGATAAGATAGCTGTA   1593
SWWR-3       TCATTTTCCATTGTAAGGAATGCATATACGTACTTCGGTACAAGAGATAAGATAGCTGTA   1592
SWWR-4       TCATTTTCCATTGTAAGGAATGCATATACGTACTTCGGTACAAGAGATAAGATAGCTGTA   1589
WUSR-1       TCATTTTCCATTGTAAGGAATGCATATACGTACTTCGGTACAAGAGATAAGATAGCTGTA   1593
WUSR-2       TCATTTTCCATTGTAAGGAATGCATATACGTACTTCGGTACAAGAGATAAGATAGCTGTA   1592
WWR-1        TCATTTTCCATTGTAAGGAATGCATATACGTACTTCGGTACAAGAGATAAGATAGCTGTA   1593
WWR-2        TCATTTTCCATTGTAAGGAATGCATATACGTACTTCGGTACAAGAGATAAGATAGCTGTA   1592
WWR-3        TCATTTTCCATTGTAAGGAATGCATATACGTACTTCGGTACAAGAGATAAGATAGCTGTA   1592
WWRNC-1      TCATTTTCCATTGTAAGGAATGCATATACGTATTTCGGTACAAGAGATAAGATAGCTGTA   1593
WWRNC-2      TCATTTTCCATTGTAAGGAATGCATATACGTACTTCGGTACAAGAGATAAGATAGCTGTA   1587
WWRNC-3      TCATTTTCCATTGTAAGGAATGCATATACGTACTTCGGTACAAGAGATAAGATAGCTGTA   1592
spelta-1     TCATTTTCCATTGTAAGGAATGCATATACGTATTTCGGTACAAGAGATAAGATAGCTGTA   1593
spelta_6     TCATTTTCCATTGTAAGGAATGCATATACGTACTTCGGTACAAGAGATAAGATAGCTGTA   1592
spelta_7     TCATTTTCCATTGTAAGGAATGCATATACGTACTTCGGTACAAGAGATAAGATAGCTGTA   1592
spelta_11    TCATTTTCCATTGTAAGGAATGCATATACGTACTTCGGTACAAGAGATAAGATAGCTGTA   1592
SWRNC-1      TCATTTTCCATTGTAAGGAATGCATATACGTATTTCGGTACAAGAGATAAGATAGCTGTA   1613
SWRNC-2      TCATTTTCCATTGTAAGGAATGCATATACGTACTTCGGTACAAGAGATAAGATAGCTGTA   1619

IWVF-1       TTTATTTTCTGTGATATAGGATTACCGCACTGTTAATGTCAAACGCAATAAAGAAAATGA   1652
IWVF-2       TTTATTTTCTGTGATATAGGATTACCGCACTGTTAATGTCAAACGCAATAAAGAAAATGA   1653
IWVF-3       TTTATTTTCTGTGATATAGGATTACCGCACTGTTAATGTCAAACGCAATAAAGAAAATGA   1652
JUSR         TTTATTTTCTGTGATATAGGATTACCGCACTGTTAATGTCAAACGCAATAAAGAAAATGA   1648
NCPSR-1      TTTATTTTCTGTGATATAGGATTACCGCACTGTTAATGTCAAACGCAATAAAGAAAATGA   1652
NCPSR-2      TTTATTTTCTGTGATATAGGATTACCGCACTGTTAATGTCAAACGCAATAAAGAAAATGA   1652
NCPSR-3      TTTATTTTCTGTGATATAGGATTACCGCACTGTTAATGTCAAACGCAATAAAGAAAATGA   1653
NHRPSR-1     TTTATTTTCTGTGATATAGGATTACCGCACTGTTAATGTCAAACGCAATAAAGAAAATGA   1652
NHRPSR-2     TTTATTTTCTGTGATATAGGATTACCGCACTGTTAATGTCAAACGCAATAAAGAAAATGA   1652
NHRPSR-3     TTTATTTTCTGTGATATAGGATTACCGCACTGTTAATGTCAAACGCAATAAAGAAAATGA   1652
NHRPSR-4     TTTATTTTCTGTGATATAGGATTACCGCACTGTTAATGTCAAACGCAATAAAGAAAATGA   1652
NHRPSR-5     TTTATTTTCTGTGATATAGGATTACCGCACTGTTAATGTCAAACGCAATAAAGAAAATGA   1653
SWWR-1       TTTATTTTCTGTGATATAGGATTACCGCACTGTTAATGTCAAACGCAATAAAGAAAATGA   1653
SWWR-2       TTTATTTTCTGTGATATAGGATTACCGCACTGTTAATGTCAAACGCAATAAAGAAAATGA   1653
SWWR-3       TTTATTTTCTGTGATATAGGATTACCGCACTGTTAATGTCAAACGCAATAAAGAAAATGA   1652
SWWR-4       TTTATTTTCTGTGATATAGGATTACCGCACTGTTAATGTCAAACGCAATAAAGAAAATGA   1649
WUSR-1       TTTATTTTCTGTGATATAGGATTACCGCACTGTTAATGTCAAACGCAATAAAGAAAATGA   1653
WUSR-2       TTTATTTTCTGTGATATAGGATTACCGCACTGTTAATGTCAAACGCAATAAAGAAAATGA   1652
WWR-1        TTTATTTTCTGTGATATAGGATTACCGCACTGTTAATGTCAAACGCAATAAAGAAAATGA   1653
WWR-2        TTTATTTTCTGTGATATAGGATTACCGCACTGTTAATGTCAAACGCAATAAAGAAAATGA   1652
WWR-3        TTTATTTTCTGTGATATAGGATTACCGCACTGTTAATGTCAAACGCAATAAAGAAAATGA   1652
WWRNC-1      TTTATTTTCTGTGATATAGGATTACCGCACTGTTAATGTCAAACGCAATAAAGAAAATGA   1653
WWRNC-2      TTTATTTTCTGTGATATAGGATTACCGCACTGTTAATGTCAAACGCAATAAAGAAAATGA   1647
WWRNC-3      TTTATTTTCTGTGATATAGGATTACCGCACTGTTAATGTCAAACGCAATAAAGAAAATGA   1652
spelta-1     TTTATTTTCTGTGATATAGGATTACCGCACTGTTAATGTCAAACGCAATAAAGAAAATGA   1653
spelta_6     TTTATTTTCTGTGATATAGGATTACCGCACTGTTAATGTCAAACGCAATAAAGAAAATGA   1652
spelta_7     TTTATTTTCTGTGATATAGGATTACCGCACTGTTAATGTCAAACGCAATAAAGAAAATGA   1652
spelta_11    TTTATTTTCTGTGATATAGGATTACCGCACTGTTAATGTCAAACGCAATAAAGAAAATGA   1652
SWRNC-1      TTTATTTTCTGTGATATAGGATTACCGCACTGTTAATGTCAAACGCAATAAAGAAAATGA   1673
SWRNC-2      TTTATTTTCTGTGATATAGGATTACCGCACTGTTAATGTCAAACGCAATAAAGAAAATGA   1679
```

```
IWVF-1        TTTTTCGATAAAATGATGCTAAATTGTTTACATAATAGGCCAATAGCTATGCGTGAGTAG   1712
IWVF-2        TTTTTCGATAAAATGATGCTAAATTGTTTACATAATAGGCCAATAGCTATGCGTGAGTAG   1713
IWVF-3        TTTTTCGATAAAATGATGCTAAATTGTTTACATAATAGGCCAATAGCTATGCGTGAGTAG   1712
JUSR          TTTTTCGATAAAATGATGCTAAATTGTTTACATAATAGGCCAATAGCTATGCGTGAGTAG   1708
NCPSR-1       TTTTTCGATAAAATGATGCTAAATTGTTTACATAATAGGCCAATAGCTATGCGTGAGTAG   1712
NCPSR-2       TTTTTCGATAAAATGATGCTAAATTGTTTACATAATAGGCCAATAGCTATGCGTGAGTAG   1712
NCPSR-3       TTTTTCGATAAAATGATGCTAAATTGTTTACATAATAGGCCAATAGCTATGCGTGAGTAG   1713
NHRPSR-1      TTTTTCGATAAAATGATGCTAAATTGTTTACATAATAGGCCAATAGCTATGCGTGAGTAG   1712
NHRPSR-2      TTTTTCGATAAAATGATGCTAAATTGTTTACATAATAGGCCAATAGCTATGCGTGAGTAG   1712
NHRPSR-3      TTTTTCGATAAAATGATGCTAAATTGTTTACATAATAGGCCAATAGCTATGCGTGAGTAG   1712
NHRPSR-4      TTTTTCGATAAAATGATGCTAAATTGTTTACATAATAGGCCAATAGCTATGCGTGAGTAG   1712
NHRPSR-5      TTTTTCGATAAAATGATGCTAAATTGTTTACATAATAGGCCAATAGCTATGCGTGAGTAG   1713
SWWR-1        TTTTTCGATAAAATGATGCTAAATTGTTTACATAATAGGCCAATAGCTATGCGTGAGTAG   1713
SWWR-2        TTTTTCGATAAAATGATGCTAAATTGTTTACATAATAGGCCAATAGCTATGCGTGAGTAG   1713
SWWR-3        TTTTTCGATAAAATGATGCTAAATTGTTTACATAATAGGCCAATAGCTATGCGTGAGTAG   1712
SWWR-4        TTTTTCGATAAAATGATGCTAAATTGTTTACATAATAGGCCAATAGCTATGCGTGAGTAG   1709
WUSR-1        TTTTTCGATAAAATGATGCTAAATTGTTTACATAATAGGCCAATAGCTATGCGTGAGTAG   1713
WUSR-2        TTTTTCGATAAAATGATGCTAAATTGTTTACATAATAGGCCAATAGCTATGCGTGAGTAG   1712
WWR-1         TTTTTCGATAAAATGATGCTAAATTGTTTACATAATAGGCCAATAGCTATGCGTGAGTAG   1713
WWR-2         TTTTTCGATAAAATGATGCTAAATTGTTTACATAATAGGCCAATAGCTATGCGTGAGTAG   1712
WWR-3         TTTTTCGATAAAATGATGCTAAATTGTTTACATAATAGGCCAATAGCTATGCGTGAGTAG   1712
WWRNC-1       TTTTTCGATAAAATGATGCTAAATTGTTTACATAATAGGCCAATAGCTATGCGTGAGTAG   1713
WWRNC-2       TTTTTCGATAAAATGATGCTAAATTGTTTACATAATAGGCCAATAGCTATGCGTGAGTAG   1707
WWRNC-3       TTTTTCGATAAAATGATGCTAAATTGTTTACATAATAGGCCAATAGCTATGCGTGAGTAG   1712
spelta-1      TTTTTCGATAAAATGATGCTAAATTGTTTACATAATAGGCCAATAGCTATGCGTGAGTAG   1713
spelta_6      TTTTTCGATAAAATGATGCTAAATTGTTTACATAATAGGCCAATAGCTATGCGTGAGTAG   1712
spelta_7      TTTTTCGATAAAATGATGCTAAATTGTTTACATAATAGGCCAATAGCTATGCGTGAGTAG   1712
spelta_11     TTTTTCGATAAAATGATGCTAAATTGTTTACATAATAGGCCAATAGCTATGCGTGAGTAG   1712
SWRNC-1       TTTTTCGATAAAATGATGCTAAATTGTTTACATAATAGGCCAATAGCTATGCGTGAGTAG   1733
SWRNC-2       TTTTTCGATAAAATGATGCTAAATTGTTTACATAATAGGCCAATAGCTATGCGTGAGTAG   1739

IWVF-1        CTTGAATTTCGAATTCGAGTCGGTCGATTCGTAGAAGGAAGGAGGAGACGAGGCGCAACA   1772
IWVF-2        CTTGAATTTCGAATTCGAGTCGGTCGATTCGTAGAAGGAAGGAGGAGACGAGGCGCAACA   1773
IWVF-3        CTTGAATTTCGAATTCGAGTGGGTCGATTCGTAGAAGGAAGGAGGAGACGAGGCGCAACA   1772
JUSR          CTTGAATTTCGAATTCGAGTGGGTCGATTCGTAGAAGGAAGGAGGAGACGAGGCGCAACA   1768
NCPSR-1       CTTGAATTTCGAATTCGAGTGGGTCGATTCGTAGAAGGAAGGAGGAGACGAGGCGCAACA   1772
NCPSR-2       CTTGAATTTCGAATTCGAGTGGGTCGATTCGTAGAAGGAAGGAGGAGACGAGGCGCAACA   1772
NCPSR-3       CTTGAATTTCGAATTCGAGTCGGTCGATTCGTAGAAGGAAGGAGGAGACGAGGCGCAACA   1773
NHRPSR-1      CTTGAATTTCGAATTCGAGTCGGTCGATTCGTAGAAGGAAGGAGGAGACGAGGCGCAACA   1772
NHRPSR-2      CTTGAATTTCGAATTCGAGTCGGTCGATTCGTAGAAGGAAGGAGGAGACGAGGCGCAACA   1772
NHRPSR-3      CTTGAATTTCGAATTCGAGTCGGTCGATTCGTAGAAGGAAGGAGGAGACGAGGCGCAACA   1772
NHRPSR-4      CTTGAATTTCGAATTCGAGTGGGTCGATTCGTAGAAGGAAGGAGGAGACGAGGCGCAACA   1772
NHRPSR-5      CTTGAATTTCGAATTCGAGTCGGTCGATTCGTAGAAGGAAGGAGGAGACGAGGCGCAACA   1773
SWWR-1        CTTGAATTTCGAATTCGAGTCGGTCGATTCGTAGAAGGAAGGAGGAGACGAGGCGCAACA   1773
SWWR-2        CTTGAATTTCGAATTCGAGTCGGTCGATTCGTAGAAGGAAGGAGGAGACGAGGCGCAACA   1773
SWWR-3        CTTGAATTTCGAATTCGAGTGGGTCGATTCGTAGAAGGAAGGAGGAGACGAGGCGCAACA   1772
SWWR-4        CTTGAATTTCGAATTCGAGTGGGTCGATTCGTAGAAGGAAGGAGGAGACGAGGCGCAACA   1769
WUSR-1        CTTGAATTTCGAATTCGAGTCGGTCGATTCGTAGAAGGAAGGAGGAGACGAGGCGCAACA   1773
WUSR-2        CTTGAATTTCGAATTCGAGTGGGTCGATTCGTAGAAGGAAGGAGGAGACGAGGCGCAACA   1772
WWR-1         CTTGAATTTCGAATTCGAGTCGGTCGATTCGTAGAAGGAAGGAGGAGACGAGGCGCAACA   1773
WWR-2         CTTGAATTTCGAATTCGAGTGGGTCGATTCGTAGAAGGAAGGAGGAGACGAGGCGCAACA   1772
WWR-3         CTTGAATTTCGAATTCGAGTCGGTCGATTCGTAGAAGGAAGGAGGAGACGAGGCGCAACA   1772
WWRNC-1       CTTGAATTTCGAATTCGAGTCGGTCGATTCGTAGAAGGAAGGAGGAGACGAGGCGCAACA   1773
WWRNC-2       CTTGAATTTCGAATTCGAGTGGGTCGATTCGTAGAAGGAAGGAGGAGACGAGGCGCAACA   1767
WWRNC-3       CTTGAATTTCGAATTCGAGTCGGTCGATTCGTAGAAGGAAGGAGGAGACGAGGCGCAACA   1772
spelta-1      CTTGAATTTCGAATTCGAGTCGGTCGATTCGTAGAAGGAAGGAGGAGACGAGGCGCAACA   1773
spelta_6      CTTGAATTTCGAATTCGAGTCGGTCGATTCGTAGAAGGAAGGAGGAGACGAGGCGCAACA   1772
spelta_7      CTTGAATTTCGAATTCGAGTCGGTCGATTCGTAGAAGGAAGGAGGAGACGAGGCGCAACA   1772
spelta_11     CTTGAATTTCGAATTCGAGTCGGTCGATTCGTAGAAGGAAGGAGGAGACGAGGCGCAACA   1772
SWRNC-1       CTTGAATTTCGAATTCGAGTCGGTCGATTCGTAGAAGGAAGGAGGAGACGAGGCGCAACA   1793
SWRNC-2       CTTGAATTTCGAATTCGAGTGGGTCGATTCGTAGAAGGAAGGAGGAGACGAGGCGCAACA   1799
```

```
IWVF-1       CCGAGGCCAAAGGCGACAGTGCCGGGGCGAGTGGAGTGATGCCCTGAGACGCTGGCAGGG  1832
IWVF-2       CCGAGGCCAAAGGCGACAGTGCCGGGGCGAGTGGAGTGATGCCCTGAGACGCTGGCAGGG  1833
IWVF-3       CCGAGGCCAAAGGCGACAGTGCCGGGGCGAGTGGAGTGATGCCCTGAGACGCTGGCAGGG  1832
JUSR         CCGAGGCCAAAGGCGACAGTGCCGGGGCGAGTGGAGTGATGCCCTGAGACGCTGGCAGGG  1828
NCPSR-1      CCGAGGCCAAAGGCGACAGTGCCGGGGCGAGTGGAGTGATGCCCTGAGACGCTGGCAGGG  1832
NCPSR-2      CCGAGGCCAAAGGCGACAGTGCCGGGGCGAGTGGAGTGATGCCCTGAGACGCTGGCAGGG  1832
NCPSR-3      CCGAGGCCAAAGGCGACAGTGCCGGGGCGAGTGGAGTGATGCCCTGAGACGCTGGCAGGG  1833
NHRPSR-1     CCGAGGCCAAAGGCGACAGTGCCGGGGCGAGTGGAGTGATGCCCTGAGACGCTGGCAGGG  1832
NHRPSR-2     CCGAGGCCAAAGGCGACAGTGCCGGGGCGAGTGGAGTGATGCCCTGAGACGCTGGCAGGG  1832
NHRPSR-3     CCGAGGCCAAAGGCGACAGTGCCGGGGCGAGTGGAGTGATGCCCTGAGACGCTGGCAGGG  1832
NHRPSR-4     CCGAGGCCAAAGGCGACAGTGCCGGGGCGAGTGGAGTGATGCCCTGAGACGCTGGCAGGG  1832
NHRPSR-5     CCGAGGCCAAAGGCGACAGTGCCGGGGCGAGTGGAGTGATGCCCTGAGACGCTGGCAGGG  1833
SWWR-1       CCGAGGCCAAAGGCGACAGTGCCGGGGCGAGTGGAGTGATGCCCTGAGACGCTGGCAGGG  1833
SWWR-2       CCGAGGCCAAAGGCGACAGTGCCGGGGCGAGTGGAGTGATGCCCTGAGACGCTGGCAGGG  1833
SWWR-3       CCGAGGCCAAAGGCGACAGTGCCGGGGCGAGTGGAGTGATGCCCTGAGACGCTGGCAGGG  1832
SWWR-4       CCGAGGCCAAAGGCGACAGTGCCGGGGCGAGTGGAGTGATGCCCTGAGACGCTGGCAGGG  1829
WUSR-1       CCGAGGCCAAAGGCGACAGTGCCGGGGCGAGTGGAGTGATGCCCTGAGACGCTGGCAGGG  1833
WUSR-2       CCGAGGCCAAAGGCGACAGTGCCGGGGCGAGTGGAGTGATGCCCTGAGACGCTGGCAGGG  1832
WWR-1        CCGAGGCCAAAGGCGACAGTGCCGGGGCGAGTGGAGTGATGCCCTGAGACGCTGGCAGGG  1833
WWR-2        CCGAGGCCAAAGGCGACAGTGCCGGGGCGAGTGGAGTGATGCCCTGAGACGCTGGCAGGG  1832
WWR-3        CCGAGGCCAAAGGCGACAGTGCCGGGGCGAGTGGAGTGATGCCCTGAGACGCTGGCAGGG  1832
WWRNC-1      CCGAGGCCAAAGGCGACAGTGCCGGGGCGAGTGGAGTGATGCCCTGAGACGCTGGCAGGG  1833
WWRNC-2      CCGAGGCCAAAGGCGACAGTGCCGGGGCGAGTGGAGTGATGCCCTGAGACGCTGGCAGGG  1827
WWRNC-3      CCGAGGCCAAAGGCGACAGTGCCGGGGCGAGTGGAGTGATGCCCTGAGACGCTGGCAGGG  1832
spelta-1     CCGAGGCCAAAGGCGACAGTGCCGGGGCGAGTGGAGTGATGCCCTGAGACGCTGGCAGGG  1833
spelta_6     CCGAGGCCAAAGGCGACAGTGCCGGGGCGAGTGGAGTGATGCCCTGAGACGCTGGCAGGG  1832
spelta_7     CCGAGGCCAAAGGCGACAGTGCCGGGGCGAGTGGAGTGATGCCCTGAGACGCTGGCAGGG  1832
spelta_11    CCGAGGCCAAAGGCGACAGTGCCGGGGCGAGTGGAGTGATGCCCTGAGACGCTGGCAGGG  1832
SWRNC-1      CCGAGGCCAAAGGCGACAGTGCCGGGGCGAGTGGAGTGATGCCCTGAGACGCTGGCAGG.  1852
SWRNC-2      CCGAGGCCAAAGGCGACAGTGCCGGGGCGAGTGGAGTGATGCCCTGAGACGCTGGCAGG.  1858

IWVF-1       CCTTGGCCGGGAACGAGTCGTA.................  1854
IWVF-2       CCTTGGCCGGGAACGAGTCGTA.................  1855
IWVF-3       CCTTGGCCGGGAACGAGTCGTAGACGGAGATAACACGGC  1871
JUSR         CCTTGGCCGGGAACGAGTCGTAGACGGAGATAACACGGC  1867
NCPSR-1      CCTTGGCCGGGAACGAGTCGTAGACGGAGATAACACGGC  1871
NCPSR-2      CCTTGGCCGGGAACGAGTCGTAGACGGAGATAACACGGC  1871
NCPSR-3      CCTTGGCCGGGAACGAGTCGTATACGGAGATAACACGGC  1872
NHRPSR-1     CCTTGGCCGGGAACGAGTCGTAGACAGAGATAACACGGC  1871
NHRPSR-2     CCTTGGCCGGGAACGAGTCGTAGACAGAGATAACACGGC  1871
NHRPSR-3     CCTTGGCCGGGAACGAGTCGTA.................  1854
NHRPSR-4     CCTTGGCCGGGAACGAGTCGTA.................  1854
NHRPSR-5     CCTTGGCCGGGAACGAGTCGTA.................  1855
SWWR-1       CCTTGGCCGGGAACGAGTCGTAGACGGAGATAACACGGC  1872
SWWR-2       CCTTGGCCGGGAACGAGTCGTA.................  1855
SWWR-3       CCTTGGCCGGGAACGAGTCGTA.................  1854
SWWR-4       CCTTGGCCGGGAACGAGTCGTAGACGGAGATAACACGGC  1868
WUSR-1       CCTTGGCCGGGAACGAGTCGTAG................  1856
WUSR-2       CCTTGGCCGGGAACGAGTCGTATACGGAGATAACACGGC  1871
WWR-1        CCTTGGCCGGGAACGAGTCGTAGACGGAGATAACACGGC  1872
WWR-2        CCTTGGCCGGGAACGAGTCGTAGACGGAGATAACACGGC  1871
WWR-3        CCTTGGCCGGGAACGAGTCGTA.................  1854
WWRNC-1      CCTTGGCCGGGAACGAGTCGTAGACGGAGATAACACGGC  1872
WWRNC-2      CCTTGGCCGGGAACGAGTCGTAGACGGAGATAAC.....  1861
WWRNC-3      CCTTGGCCGGGAACGAGTCGTA.................  1854
spelta-1     CCTTGGCCGGGAACGAGTCGTAGACGGAGATAACACGGC  1872
spelta_6     CCTTGGCCGGGAACGAGTCGTAGACAGAGATAACACGGC  1871
spelta_7     CCTTGGCCGGGAACGAGTCGTAGACAGAGATAACACGGC  1871
spelta_11    CCTTGGCCGGGAACGAGTCGTAGACGGAGATAACACGGC  1871
SWRNC-1      .......................................  1852
SWRNC-2      .......................................  1858
```

Supplementary Fig. S9 Sequence alignments of the *Wcor15-2B* from hexaploid wheat with different geographical areas. There were 3 different sequences in

IWVF, 3 in NCPSR, 5 in NHRPSR, 4 in SWWR, 2 in WUSR, 3 in WWR, 3 in WWRNC, 2 in SWRNC and 4 spelta wheats.

```
WWRNC    CTCCTTGGGAGCAGTATAATAAGTGATGTAAGACTTTCAATATTATATTTTTGCTGAGTT   60
NCPSR    CTCCTTGGGAGCAGTATAATAAGTGATGTAAGACTTTCAATATTATATTTTTGCTGAGTT   60
NHRPSR   CTCCTTGGGAGCAGTATAATAAGTGATGTAAGACTTTCAATATTATATTTTTGCTGAGTT   60
WUSR     CTCCTTGGGAGCAGTATAATAAGTGATGTAAGACTTTCAATATTATATTTTTGCTGAGTT   60
JUSR     CTCCTTGGGAGCAGTATAATAAGTGATGTAAGACTTTCAATATTATATTTTTGCTGAGTT   60
WWR      CTCCTTGGGAGCAGTATAATAAGTGATGTAAGACTTTCAATATTATATTTTTGCTGAGTT   60
SWWR     CTCCTTGGGAGCAGTATAATAAGTGATGTAAGACTTTCAATATTATATTTTTGCTGAGTT   60
IWVF     CTCCTTGGGAGCAGTATAATAAGTGATGTAAGACTTTCAATATTATATTTTTGCTGAGTT   60
SWRNC    CTCCTTGGGAGCAGTATAATAAGTGATGTAAGACTTTCAATATTATATTTTTGCTGAGTT   60
Spelta   CTCCTTGGGAGCAGTATAATAAGTGATGTAAGACTTTCAATATTATATTTTTGCTGAGTT   60

WWRNC    GAAGAAGAGGGGAGAGAAAAGAGAATAGAAGCAGGCTACAGACTAATAACCAGTTGTAGC   120
NCPSR    GAAGAAGAGGGGAGAGAAAAGAGAATAGAAGCAGGCTACAGACTAATAACCAGTTGTAGC   120
NHRPSR   GAAGAAGAGGGGAGAGAAAAGAGAATAGAAGCAGGCTACAGACTAATAACCAGTTGTAGC   120
WUSR     GAAGAAGAGGGGAGAGAAAAGAGAATAGAAGCAGGCTACAGACTAATAACCAGTTGTAGC   120
JUSR     GAAGAAGAGGGGAGAGAAAAGAGAATAGAAGCAGGCTACAGACTAATAACCAGTTGTAGC   120
WWR      GAAGAAGAGGGGAGAGAAAAGAGAATAGAAGCAGGCTACAGACTAATAACCAGTTGTAGC   120
SWWR     GAAGAAGAGGGGAGAGAAAAGAGAATAGAAGCAGGCTACAGACTAATAACCAGTTGTAGC   120
IWVF     GAAGAAGAGGGGAGAGAAAAGAGAATAGAAGCAGGCTACAGACTAATAACCAGTTGTAGC   120
SWRNC    GAAGAAGAGGGGAGAGAAAAGAGAATAGAAGCAGGCTACAGACTAATAACCAGTTGTAGC   120
Spelta   GAAGAAGAGGGGAGAGAAAAGAGAATAGAAGCAGGCTACAGACTAATAACCAGTTGTAGC   120

WWRNC    ATGTGCCCTAGAGGCTTTGTGAAAGAGTGAGGTGAGTTATGTATCAACAAAGTAATACTC   180
NCPSR    ATGTGCCCTAGAGGCTTTGTGAAAGAGTGAGGTGAGTTATGTATCAACAAAGTAATACTC   180
NHRPSR   ATGTGCCCTAGAGGCTTTGTGAAAGAGTGAGGTGAGTTATGTATCAACAAAGTAATACTC   180
WUSR     ATGTGCCCTAGAGGCTTTGTGAAAGAGTGAGGTGAGTTATGTATCAACAAAGTAATACTC   180
JUSR     ATGTGCCCTAGAGGCTTTGTGAAAGAGTGAGGTGAGTTATGTATCAACAAAGTAATACTC   180
WWR      ATGTGCCCTAGAGGCTTTGTGAAAGAGTGAGGTGAGTTATGTATCAACAAAGTAATACTC   180
SWWR     ATGTGCCCTAGAGGCTTTGTGAAAGAGTGAGGTGAGTTATGTATCAACAAAGTAATACTC   180
IWVF     ATGTGCCCTAGAGGCTTTGTGAAAGAGTGAGGTGAGTTATGTATCAACAAAGTAATACTC   180
SWRNC    ATGTGCCCTAGAGGCTTTGTGAAAGAGTGAGGTGAGTTATGTATCAACAAAGTAATACTC   180
Spelta   ATGTGCCCTAGAGGCTTTGTGAAAGAGTGAGGTGAGTTATGTATCAACAAAGTAATACTC   180

WWRNC    CCTTTGCCTAGAAATACTTGTTGGAAAAATAGATAAAAATGAATGTACTATCTAGAATTA   240
NCPSR    CCTTTGCCTAGAAATACTTGTTGGAAAAATAGATAAAAATGAATGTACTATCTAGAATTA   240
NHRPSR   CCTTTGCCTAGAAATACTTGTTGGAAAAATAGATAAAAATGAATGTACTATCTAGAATTA   240
WUSR     CCTTTGCCTAGAAATACTTGTTGGAAAAATAGATAAAAATGAATGTACTATCTAGAATTA   240
JUSR     CCTTTGCCTAGAAATACTTGTTGGAAAAATAGATAAAAATGAATGTACTATCTAGAATTA   240
WWR      CCTTTGCCTAGAAATACTTGTTGGAAAAATAGATAAAAATGAATGTACTATCTAGAATTA   240
SWWR     CCTTTGCCTAGAAATACTTGTTGGAAAAATAGATAAAAATGAATGTACTATCTAGAATTA   240
IWVF     CCTTTGCCTAGAAATACTTGTTGGAAAAATAGATAAAAATGAATGTACTATCTAGAATTA   240
SWRNC    CCTTTGCCTAGAAATACTTGTTGGAAAAATAGATAAAAATGAATGTACTATCTAGAATTA   240
Spelta   CCTTTGCCTAGAAATACTTGTTGGAAAAATAGATAAAAATGAATGTACTATCTAGAATTA   240

WWRNC    AAATACGCCCAAATACATCATTTTTTCTGATAAATATTTTCAGACGGATGGAGTACATAT   300
NCPSR    AAATACGCCCAAATACATCATTTTTTCTGATAAATATTTTCAGACGGATGGAGTACATAT   300
NHRPSR   AAATACGCCCAAATACATCATTTTTTCTGATAAATATTTTCAGACGGATGGAGTACATAT   300
WUSR     AAATACGCCCAAATACATCATTTTTTCTGATAAATATTTTCAGACGGATGGAGTACATAT   300
JUSR     AAATACGCCCAAATACATCATTTTTTCTGATAAATATTTTCAGACGGATGGAGTACATAT   300
WWR      AAATACGCCCAAATACATCATTTTTTCTGATAAATATTTTCAGACGGATGGAGTACATAT   300
SWWR     AAATACGCCCAAATACATCATTTTTTCTGATAAATATTTTCAGACGGATGGAGTACATAT   300
IWVF     AAATACGCCCAAATACATCATTTTTTCTGATAAATATTTTCAGACGGATGGAGTACATAT   300
SWRNC    AAATACGCCCAAATACATCATTTTTTCTGATAAATATTTTCAGACGGATGGAGTACATAT   300
Spelta   AAATACGCCCAAATACATCATTTTTTCTGATAAATATTTTCAGACGGATGGAGTACATAT   300

WWRNC    TTATATCTAACTATTATACTTGCCGCATATAAATTTAATTATAGATGACGTGCCAACATC   360
NCPSR    TTATATCTAACTATTATACTTGCCGCATATAAATTTAATTATAGATGACGTGCCAACATC   360
NHRPSR   TTATATCTAACTATTATACTTGCCGCATATAAATTTAATTATAGATGACGTGCCAACATC   360
WUSR     TTATATCTAACTATTATACTTGCCGCATATAAATTTAATTATAGATGACGTGCCAACATC   360
JUSR     TTATATCTAACTATTATACTTGCCGCATATAAATTTAATTATAGATGACGTGCCAACATC   360
WWR      TTATATCTAACTATTATACTTGCCGCATATAAATTTAATTATAGATGACGTGCCAACATC   360
SWWR     TTATATCTAACTATTATACTTGCCGCATATAAATTTAATTATAGATGACGTGCCAACATC   360
IWVF     TTATATCTAACTATTATACTTGCCGCATATAAATTTAATTATAGATGACGTGCCAACATC   360
SWRNC    TTATATCTAACTATTATACTTGCCGCATATAAATTTAATTATAGATGACGTGCCAACATC   360
Spelta   TTATATCTAACTATTATACTTGCCGCATATAAATTTAATTATAGATGACGTGCCAACATC   360
```

```
WWRNC     ATATAGCCAGCAGCCGGCTATGCTATTAAACATGTTGTGGACCATGTATCGGCGACGTCA     420
NCPSR     ATATAGCCAGCAGCCGGCTATGCTATTAAACATGTTGTGGACCATGTATCGGCGACGTCA     420
NHRPSR    ATATAGCCAGCAGCCGGCTATGCTATTAAACATGTTGTGGACCATGTATCGGCGACGTCA     420
WUSR      ATATAGCCAGCAGCCGGCTATGCTATTAAACATGTTGTGGACCATGTATCGGCGACGTCA     420
JUSR      ATATAGCCAGCAGCCGGCTATGCTATTAAACATGTTGTGGACCATGTATCGGCGACGTCA     420
WWR       ATATAGCCAGCAGCCGGCTATGCTATTAAACATGTTGTGGACCATGTATCGGCGACGTCA     420
SWWR      ATATAGCCAGCAGCCGGCTATGCTATTAAACATGTTGTGGACCATGTATCGGCGACGTCA     420
IWVF      ATATAGCCAGCAGCCGGCTATGCTATTAAACATGTTGTGGACCATGTATCGGCGACGTCA     420
SWRNC     ATATAGCCAGCAGCCGGCTATGCTATTAAACATGTTGTGGACCATGTATCGGCGACGTCA     420
Spelta    ATATAGCCAGCAGCCGGCTATGCTATTAAACATGTTGTGGACCATGTATCGGCGACGTCA     420

WWRNC     TTATTGTTCATTCAATCACTTTCCAGTTTAATATTTTCTACAGATGGGATATAAATATGG     480
NCPSR     TTATTGTTCATTCAATCACTTTCCAGTTTAATATTTTCTACAGATGGGATATAAATATGG     480
NHRPSR    TTATTGTTCATTCAATCACTTTCCAGTTTAATATTTTCTACAGATGGGATATAAATATGG     480
WUSR      TTATTGTTCATTCAATCACTTTCCAGTTTAATATTTTCTACAGATGGGATATAAATATGG     480
JUSR      TTATTGTTCATTCAATCACTTTCCAGTTTAATATTTTCTACAGATGGGATATAAATATGG     480
WWR       TTATTGTTCATTCAATCACTTTCCAGTTTAATATTTTCTACAGATGGGATATAAATATGG     480
SWWR      TTATTGTTCATTCAATCACTTTCCAGTTTAATATTTTCTACAGATGGGATATAAATATGG     480
IWVF      TTATTGTTCATTCAATCACTTTCCAGTTTAATATTTTCTACAGATGGGATATAAATATGG     480
SWRNC     TTATTGTTCATTCAATCACTTTCCAGTTTAATATTTTCTACAGATGGGATATAAATATGG     480
Spelta    TTATTGTTCATTCAATCACTTTCCAGTTTAATATTTTCTACAGATGGGATATAAATATGG     480

WWRNC     CTGAACTTACCCTTGATTAATCGACGGCCGGTATCAAATATCTCCTGTTATCATAAGTTG     540
NCPSR     CTGAACTTACCCTTGATTAATCGACGGCCGGTATCAAATATCTCCTGTTATCATAAGTTG     540
NHRPSR    CTGAACTTACCCTTGATTAATCGACGGCCGGTATCAAATATCTCCTGTTATCATAAGTTG     540
WUSR      CTGAACTTACCCTTGATTAATCGACGGCCGGTATCAAATATCTCCTGTTATCATAAGTTG     540
JUSR      CTGAACTTACCCTTGATTAATCGACGGCCGGTATCAAATATCTCCTGTTATCATAAGTTG     540
WWR       CTGAACTTACCCTTGATTAATCGACGGCCGGTATCAAATATCTCCTGTTATCATAAGTTG     540
SWWR      CTGAACTTACCCTTGATTAATCGACGGCCGGTATCAAATATCTCCTGTTATCATAAGTTG     540
IWVF      CTGAACTTACCCTTGATTAATCGACGGCCGGTATCAAATATCTCCTGTTATCATAAGTTG     540
SWRNC     CTGAACTTACCCTTGATTAATCGACGGCCGGTATCAAATATCTCCTGTTATCATAAGTTG     540
Spelta    CTGAACTTACCCTTGATTAATCGACGGCCGGTATCAAATATCTCCTGTTATCATAAGTTG     540

WWRNC     AAGCATCTTCAAAGAGCTCCTGACAAAACCTTGGTTGTACGTAACCTTCAAACGTGGACA     600
NCPSR     AAGCATCTTCAAAGAGCTCCTGACAAAACCTTGGTTGTACGTAACCTTCAAACGTGGACA     600
NHRPSR    AAGCATCTTCAAAGAGCTCCTGACAAAACCTTGGTTGTACGTAACCTTCAAACGTGGACA     600
WUSR      AAGCATCTTCAAAGAGCTCCTGACAAAACCTTGGTTGTACGTAACCTTCAAACGTGGACA     600
JUSR      AAGCATCTTCAAAGAGCTCCTGACAAAACCTTGGTTGTACGTAACCTTCAAACGTGGACA     600
WWR       AAGCATCTTCAAAGAGCTCCTGACAAAACCTTGGTTGTACGTAACCTTCAAACGTGGACA     600
SWWR      AAGCATCTTCAAAGAGCTCCTGACAAAACCTTGGTTGTACGTAACCTTCAAACGTGGACA     600
IWVF      AAGCATCTTCAAAGAGCTCCTGACAAAACCTTGGTTGTACGTAACCTTCAAACGTGGACA     600
SWRNC     AAGCATCTTCAAAGAGCTCCTGACAAAACCTTGGTTGTACGTAACCTTCAAACGTGGACA     600
Spelta    AAGCATCTTCAAAGAGCTCCTGACAAAACCTTGGTTGTACGTAACCTTCAAACGTGGACA     600

WWRNC     GTAGGATTTGTTTCTTCGATCCGCTCCAAACAGGTCATGTAGGTATACGTGTGTTCCCGT     660
NCPSR     GTAGGATTTGTTTCTTCGATCCGCTCCAAACAGGTCATGTAGGTATACGTGTGTTCCCGT     660
NHRPSR    GTAGGATTTGTTTCTTCGATCCGCTCCAAACAGGTCATGTAGGTATACGTGTGTTCCCGT     660
WUSR      GTAGGATTTGTTTCTTCGATCCGCTCCAAACAGGTCATGTAGGTATACGTGTGTTCCCGT     660
JUSR      GTAGGATTTGTTTCTTCGATCCGCTCCAAACAGGTCATGTAGGTATACGTGTGTTCCCGT     660
WWR       GTAGGATTTGTTTCTTCGATCCGCTCCAAACAGGTCATGTAGGTATACGTGTGTTCCCGT     660
SWWR      GTAGGATTTGTTTCTTCGATCCGCTCCAAACAGGTCATGTAGGTATACGTGTGTTCCCGT     660
IWVF      GTAGGATTTGTTTCTTCGATCCGCTCCAAACAGGTCATGTAGGTATACGTGTGTTCCCGT     660
SWRNC     GTAGGATTTGTTTCTTCGATCCGCTCCAAACAGGTCATGTAGGTATACGTGTGTTCCCGT     660
Spelta    GTAGGATTTGTTTCTTCGATCCGCTCCAAACAGGTCATGTAGGTATACGTGTGTTCCCGT     660

WWRNC     GAGAGGCGTGTGGGGTAAGCAGCTAGTAGTAGCAAGCATGGGTGCAGCAGATACCCATTT     720
NCPSR     GAGAGGCGTGTGGGGTAAGCAGCTAGTAGTAGCAAGCATGGGTGCAGCAGATACCCATTT     720
NHRPSR    GAGAGGCGTGTGGGGTAAGCAGCTAGTAGTAGCAAGCATGGGTGCAGCAGATACCCATTT     720
WUSR      GAGAGGCGTGTGGGGTAAGCAGCTAGTAGTAGCAAGCATGGGTGCAGCAGATACCCATTT     720
JUSR      GAGAGGCGTGTGGGGTAAGCAGCTAGTAGTAGCAAGCATGGGTGCAGCAGATACCCATTT     720
WWR       GAGAGGCGTGTGGGGTAAGCAGCTAGTAGTAGCAAGCATGGGTGCAGCAGATACCCATTT     720
SWWR      GAGAGGCGTGTGGGGTAAGCAGCTAGTAGTAGCAAGCATGGGTGCAGCAGATACCCATTT     720
IWVF      GAGAGGCGTGTGGGGTAAGCAGCTAGTAGTAGCAAGCATGGGTGCAGCAGATACCCATTT     720
SWRNC     GAGAGGCGTGTGGGGTAAGCAGCTAGTAGTAGCAAGCATGGGTGCAGCAGATACCCATTT     720
Spelta    GAGAGGCGTGTGGGGTAAGCAGCTAGTAGTAGCAAGCATGGGTGCAGCAGATACCCATTT     720
```

```
WWRNC     CTTTTCTTTTATTGGTATGAGAAATTCGACCGTACATTACCACATAACCTTGTCGACAGT   780
NCPSR     CTTTTCTTTTATTGGTATGAGAAATTCGACCGTACATTACCACATAACCTTGTCGACAGT   780
NHRPSR    CTTTTCTTTTATTGGTATGAGAAATTCGACCGTACATTACCACATAACCTTGTCGACAGT   780
WUSR      CTTTTCTTTTATTGGTATGAGAAATTCGACCGTACATTACCACATAACCTTGTCGACAGT   780
JUSR      CTTTTCTTTTATTGGTATGAGAAATTCGACCGTACATTACCACATAACCTTGTCGACAGT   780
WWR       CTTTTCTTTTATTGGTATGAGAAATTCGACCGTACATTACCACATAACCTTGTCGACAGT   780
SWWR      CTTTTCTTTTATTGGTATGAGAAATTCGACCGTACATTACCACATAACCTTGTCGACAGT   780
IWVF      CTTTTCTTTTATTGGTATGAGAAATTCGACCGTACATTACCACATAACCTTGTCGACAGT   780
SWRNC     CTTTTCTTTTATTGGTATGAGAAATTCGACCGTACATTACCACATAACCTTGTCGACAGT   780
Spelta    CTTTTCTTTTATTGGTATGAGAAATTCGACCGTACATTACCACATAACCTTGTCGACAGT   780

WWRNC     TCTCCGCGCACGGCTGCACCACTCAACCTTGTCGACCGTCGATTGTCGCCATGCGCCGGA   840
NCPSR     TCTCCGCGCACGGCTGCACCACTCAACCTTGTCGACCGTCGATTGTCGCCATGCGCCGGA   840
NHRPSR    TCTCCGCGCACGGCTGCACCACTCAACCTTGTCGACCGTCGATTGTCGCCATGCGCCGGA   840
WUSR      TCTCCGCGCACGGCTGCACCACTCAACCTTGTCGACCGTCGATTGTCGCCATGCGCCGGA   840
JUSR      TCTCCGCGCACGGCTGCACCACTCAACCTTGTCGACCGTCGATTGTCGCCATGCGCCGGA   840
WWR       TCTCCGCGCACGGCTGCACCACTCAACCTTGTCGACCGTCGATTGTCGCCATGCGCCGGA   840
SWWR      TCTCCGCGCACGGCTGCACCACTCAACCTTGTCGACCGTCGATTGTCGCCATGCGCCGGA   840
IWVF      TCTCCGCGCACGGCTGCACCACTCAACCTTGTCGACCGTCGATTGTCGCCATGCGCCGGA   840
SWRNC     TCTCCGCGCACGGCTGCACCACTCAACCTTGTCGACCGTCGATTGTCGCCATGCGCCGGA   840
Spelta    TCTCCGCGCACGGCTGCACCACTCAACCTTGTCGACCGTCGATTGTCGCCATGCGCCGGA   840

WWRNC     GCGATCGCCGCCCGACGTGTCGCCAGCCACCTCACCCTTCGCCCCTCCGCGTCCAGCCTA   900
NCPSR     GCGATCGCCGCCCGACGTGTCGCCAGCCACCTCACCCTTCGCCCCTCCGCGTCCAGCCTA   900
NHRPSR    GCGATCGCCGCCCGACGTGTCGCCAGCCACCTCACCCTTCGCCCCTCCGCGTCCAGCCTA   900
WUSR      GCGATCGCCGCCCGACGTGTCGCCAGCCACCTCACCCTTCGCCCCTCCGCGTCCAGCCTA   900
JUSR      GCGATCGCCGCCCGACGTGTCGCCAGCCACCTCACCCTTCGCCCCTCCGCGTCCAGCCTA   900
WWR       GCGATCGCCGCCCGACGTGTCGCCAGCCACCTCACCCTTCGCCCCTCCGCGTCCAGCCTA   900
SWWR      GCGATCGCCGCCCGACGTGTCGCCAGCCACCTCACCCTTCGCCCCTCCGCGTCCAGCCTA   900
IWVF      GCGATCGCCGCCCGACGTGTCGCCAGCCACCTCACCCTTCGCCCCTCCGCGTCCAGCCTA   900
SWRNC     GCGATCGCCGCCCGACGTGTCGCCAGCCACCTCACCCTTCGCCCCTCCGCGTCCAGCCTA   900
Spelta    GCGATCGCCGCCCGACGTGTCGCCAGCCACCTCACCCTTCGCCCCTCCGCGTCCAGCCTA   900

WWRNC     TAAAAACGACGATGCACCTCTCGTCCTCCCATCGGACTGCAAGTGAAGCTCACACGACCT   960
NCPSR     TAAAAACGACGATGCACCTCTCGTCCTCCCATCGGACTGCAAGTGAAGCTCACACGACCT   960
NHRPSR    TAAAAACGACGATGCACCTCTCGTCCTCCCATCGGACTGCAAGTGAAGCTCACACGACCT   960
WUSR      TAAAAACGACGATGCACCTCTCGTCCTCCCATCGGACTGCAAGTGAAGCTCACACGACCT   960
JUSR      TAAAAACGACGATGCACCTCTCGTCCTCCCATCGGACTGCAAGTGAAGCTCACACGACCT   960
WWR       TAAAAACGACGATGCACCTCTCGTCCTCCCATCGGACTGCAAGTGAAGCTCACACGACCT   960
SWWR      TAAAAACGACGATGCACCTCTCGTCCTCCCATCGGACTGCAAGTGAAGCTCACACGACCT   960
IWVF      TAAAAACGACGATGCACCTCTCGTCCTCCCATCGGACTGCAAGTGAAGCTCACACGACCT   960
SWRNC     TAAAAACGACGATGCACCTCTCGTCCTCCCATCGGACTGCAAGTGAAGCTCACACGACCT   960
Spelta    TAAAAACGACGATGCACCTCTCGTCCTCCCATCGGACTGCAAGTGAAGCTCACACGACCT   960

WWRNC     AGCCTACCCTACCCACCCATCCATCAGCAGTTTTTCTATCGGCCAATGGCTTCTTCTTCT   1020
NCPSR     AGCCTACCCTACCCACCCATCCATCAGCAGTTTTTCTATCGGCCAATGGCTTCTTCTTCT   1020
NHRPSR    AGCCTACCCTACCCACCCATCCATCAGCAGTTTTTCTATCGGCCAATGGCTTCTTCTTCT   1020
WUSR      AGCCTACCCTACCCACCCATCCATCAGCAGTTTTTCTATCGGCCAATGGCTTCTTCTTCT   1020
JUSR      AGCCTACCCTACCCACCCATCCATCAGCAGTTTTTCTATCGGCCAATGGCTTCTTCTTCT   1020
WWR       AGCCTACCCTACCCACCCATCCATCAGCAGTTTTTCTATCGGCCAATGGCTTCTTCTTCT   1020
SWWR      AGCCTACCCTACCCACCCATCCATCAGCAGTTTTTCTATCGGCCAATGGCTTCTTCTTCT   1020
IWVF      AGCCTACCCTACCCACCCATCCATCAGCAGTTTTTCTATCGGCCAATGGCTTCTTCTTCT   1020
SWRNC     AGCCTACCCTACCCACCCATCCATCAGCAGTTTTTCTATCGGCCAATGGCTTCTTCTTCT   1020
Spelta    AGCCTACCCTACCCACCCATCCATCAGCAGTTTTTCTATCGGCCAATGGCTTCTTCTTCT   1020

WWRNC     GTGCTGCTCGGAGCCTCGGCCACGGCCGCGCTCACCGGTACCCCGGCAGGCAAGGCCCTT   1080
NCPSR     GTGCTGCTCGGAGCCTCGGCCACGGCCGCGCTCACCGGTACCCCGGCAGGCAAGGCCCTT   1080
NHRPSR    GTGCTGCTCGGAGCCTCGGCCACGGCCGCGCTCACCGGTACCCCGGCAGGCAAGGCCCTT   1080
WUSR      GTGCTGCTCGGAGCCTCGGCCACGGCCGCGCTCACCGGTACCCCGGCAGGCAAGGCCCTT   1080
JUSR      GTGCTGCTCGGAGCCTCGGCCACGGCCGCGCTCACCGGTACCCCGGCAGGCAAGGCCCTT   1080
WWR       GTGCTGCTCGGAGCCTCGGCCACGGCCGCGCTCACCGGTACCCCGGCAGGCAAGGCCCTT   1080
SWWR      GTGCTGCTCGGAGCCTCGGCCACGGCCGCGCTCACCGGTACCCCGGCAGGCAAGGCCCTT   1080
IWVF      GTGCTGCTCGGAGCCTCGGCCACGGCCGCGCTCACCGGTACCCCGGCAGGCAAGGCCCTT   1080
SWRNC     GTGCTGCTCGGAGCCTCGGCCACGGCCGCGCTCACCGGTACCCCGGCAGGCAAGGCCCTT   1080
Spelta    GTGCTGCTCGGAGCCTCGGCCACGGCCGCGCTCACCGGTACCCCGGCAGGCAAGGCCCTT   1080
```

```
WWRNC     CCCCGGCCTTGCTTCCTCGCCGCTCGCCCGCGCACCGTGAGCGGTGGCCGCCTCTGCCTG   1140
NCPSR     CCCCGGCCTTGCTTCCTCGCCGCTCGCCCGCGCACCGTGAGCGGTGGCCGCCTCTGCCTG   1140
NHRPSR    CCCCGGCCTTGCTTCCTCGCCGCTCGCCCGCGCACCGTGAGCGGTGGCCGCCTCTGCCTG   1140
WUSR      CCCCGGCCTTGCTTCCTCGCCGCTCGCCCGCGCACCGTGAGCGGTGGCCGCCTCTGCCTG   1140
JUSR      CCCCGGCCTTGCTTCCTCGCCGCTCGCCCGCGCACCGTGAGCGGTGGCCGCCTCTGCCTG   1140
WWR       CCCCGGCCTTGCTTCCTCGCCGCTCGCCCGCGCACCGTGAGCGGTGGCCGCCTCTGCCTG   1140
SWWR      CCCCGGCCTTGCTTCCTCGCCGCTCGCCCGCGCACCGTGAGCGGTGGCCGCCTCTGCCTG   1140
IWVF      CCCCGGCCTTGCTTCCTCGCCGCTCGCCCGCGCACCGTGAGCGGTGGCCGCCTCTGCCTG   1140
SWRNC     CCCCGGCCTTGCTTCCTCGCCGCTCGCCCGCGCACCGTGAGCGGTGGCCGCCTCTGCCTG   1140
Spelta    CCCCGGCCTTGCTTCCTCGCCGCTCGCCCGCGCACCGTGAGCGGTGGCCGCCTCTGCCTG   1140

WWRNC     CAGAACGCTCCAAGGGCGACTCCGGTACGTGTGTCCCGAGCAGTACCGTTCGATCATGAC   1200
NCPSR     CAGAACGCTCCAAGGGCGACTCCGGTACGTGTGTCCCGAGCAGTACCGTTCGATCATGAC   1200
NHRPSR    CAGAACGCTCCAAGGGCGACTCCGGTACGTGTGTCCCGAGCAGTACCGTTCGATCATGAC   1200
WUSR      CAGAACGCTCCAAGGGCGACTCCGGTACGTGTGTCCCGAGCAGTACCGTTCGATCATGAC   1200
JUSR      CAGAACGCTCCAAGGGCGACTCCGGTACGTGTGTCCCGAGCAGTACCGTTCGATCATGAC   1200
WWR       CAGAACGCTCCAAGGGCGACTCCGGTACGTGTGTCCCGAGCAGTACCGTTCGATCATGAC   1200
SWWR      CAGAACGCTCCAAGGGCGACTCCGGTACGTGTGTCCCGAGCAGTACCGTTCGATCATGAC   1200
IWVF      CAGAACGCTCCAAGGGCGACTCCGGTACGTGTGTCCCGAGCAGTACCGTTCGATCATGAC   1200
SWRNC     CAGAACGCTCCAAGGGCGACTCCGGTACGTGTGTCCCGAGCAGTACCGTTCGATCATGAC   1200
Spelta    CAGAACGCTCCAAGGGCGACTCCGGTACGTGTGTCCCGAGCAGTACCGTTCGATCATGAC   1200

WWRNC     TTCATGCCGATTATTTTACGTACGCTGCTGGTTAATCAATCGTTCTATGGGATACGCTGA   1260
NCPSR     TTCATGCCGATTATTTTACGTACGCTGCTGGTTAATCAATCGTTCTATGGGATACGCTGA   1260
NHRPSR    TTCATGCCGATTATTTTACGTACGCTGCTGGTTAATCAATCGTTCTATGGGATACGCTGA   1260
WUSR      TTCATGCCGATTATTTTACGTACGCTGCTGGTTAATCAATCGTTCTATGGGATACGCTGA   1260
JUSR      TTCATGCCGATTATTTTACGTACGCTGCTGGTTAATCAATCGTTCTATGGGATACGCTGA   1260
WWR       TTCATGCCGATTATTTTACGTACGCTGCTGGTTAATCAATCGTTCTATGGGATACGCTGA   1260
SWWR      TTCATGCCGATTATTTTACGTACGCTGCTGGTTAATCAATCGTTCTATGGGATACGCTGA   1260
IWVF      TTCATGCCGATTATTTTACGTACGCTGCTGGTTAATCAATCGTTCTATGGGATACGCTGA   1260
SWRNC     TTCATGCCGATTATTTTACGTACGCTGCTGGTTAATCAATCGTTCTATGGGATACGCTGA   1260
Spelta    TTCATGCCGATTATTTTACGTACGCTGCTGGTTAATCAATCGTTCTATGGGATACGCTGA   1260

WWRNC     CCGAGATATATGCGTGCATGGGACAGGCGTACAACGACGCTGCGGATGCCACCGACAAGG   1320
NCPSR     CCGAGATATATGCGTGCATGGGACAGGCGTACAACGACGCTGCGGATGCCACCGACAAGG   1320
NHRPSR    CCGAGATATATGCGTGCATGGGACAGGCGTACAACGACGCTGCGGATGCCACCGACAAGG   1320
WUSR      CCGAGATATATGCGTGCATGGGACAGGCGTACAACGACGCTGCGGATGCCACCGACAAGG   1320
JUSR      CCGAGATATATGCGTGCATGGGACAGGCGTACAACGACGCTGCGGATGCCACCGACAAGG   1320
WWR       CCGAGATATATGCGTGCATGGGACAGGCGTACAACGACGCTGCGGATGCCACCGACAAGG   1320
SWWR      CCGAGATATATGCGTGCATGGGACAGGCGTACAACGACGCTGCGGATGCCACCGACAAGG   1320
IWVF      CCGAGATATATGCGTGCATGGGACAGGCGTACAACGACGCTGCGGATGCCACCGACAAGG   1320
SWRNC     CCGAGATATATGCGTGCATGGGACAGGCGTACAACGACGCTGCGGATGCCACCGACAAGG   1320
Spelta    CCGAGATATATGCGTGCATGGGACAGGCGTACAACGACGCTGCGGATGCCACCGACAAGG   1320

WWRNC     CCATCGAGGGCGTGAAGGGGGTGGCCGACGAGCTGAAGAAGGGCGTGGCGGAGGCGGCGG   1380
NCPSR     CCATCGAGGGCGTGAAGGGGGTGGCCGACGAGCTGAAGAAGGGCGTGGCGGAGGCGGCGG   1380
NHRPSR    CCATCGAGGGCGTGAAGGGGGTGGCCGACGAGCTGAAGAAGGGCGTGGCGGAGGCGGCGG   1380
WUSR      CCATCGAGGGCGTGAAGGGGGTGGCCGACGAGCTGAAGAAGGGCGTGGCGGAGGCGGCGG   1380
JUSR      CCATCGAGGGCGTGAAGGGGGTGGCCGACGAGCTGAAGAAGGGCGTGGCGGAGGCGGCGG   1380
WWR       CCATCGAGGGCGTGAAGGGGGTGGCCGACGAGCTGAAGAAGGGCGTGGCGGAGGCGGCGG   1380
SWWR      CCATCGAGGGCGTGAAGGGGGTGGCCGACGAGCTGAAGAAGGGCGTGGCGGAGGCGGCGG   1380
IWVF      CCATCGAGGGCGTGAAGGGGGTGGCCGACGAGCTGAAGAAGGGCGTGGCGGAGGCGGCGG   1380
SWRNC     CCATCGAGGGCGTGAAGGGGGTGGCCGACGAGCTGAAGAAGGGCGTGGCGGAGGCGGCGG   1380
Spelta    CCATCGAGGGCGTGAAGGGGGTGGCCGACGAGCTGAAGAAGGGCGTGGCGGAGGCGGCGG   1380

WWRNC     AGGCCGTCTCGGGCAACACCGAGAAGGCCGCGGAGGAAGCCGGCAAGGGCGCGAGCGAGG   1440
NCPSR     AGGCCGTCTCGGGCAACACCGAGAAGGCCGCGGAGGAAGCCGGCAAGGGCGCGAGCGAGG   1440
NHRPSR    AGGCCGTCTCGGGCAACACCGAGAAGGCCGCGGAGGAAGCCGGCAAGGGCGCGAGCGAGG   1440
WUSR      AGGCCGTCTCGGGCAACACCGAGAAGGCCGCGGAGGAAGCCGGCAAGGGCGCGAGCGAGG   1440
JUSR      AGGCCGTCTCGGGCAACACCGAGAAGGCCGCGGAGGAAGCCGGCAAGGGCGCGAGCGAGG   1440
WWR       AGGCCGTCTCGGGCAACACCGAGAAGGCCGCGGAGGAAGCCGGCAAGGGCGCGAGCGAGG   1440
SWWR      AGGCCGTCTCGGGCAACACCGAGAAGGCCGCGGAGGAAGCCGGCAAGGGCGCGAGCGAGG   1440
IWVF      AGGCCGTCTCGGGCAACACCGAGAAGGCCGCGGAGGAAGCCGGCAAGGGCGCGAGCGAGG   1440
SWRNC     AGGCCGTCTCGGGCAACACCGAGAAGGCCGCGGAGGAAGCCGGCAAGGGCGCGAGCGAGG   1440
Spelta    AGGCCGTCTCGGGCAACACCGAGAAGGCCGCGGAGGAAGCCGGCAAGGGCGCGAGCGAGG   1440
```

```
WWRNC     TGGACGACAAGGCCAAGGACTTCGGCGAGCAGGCGAAGAAGGCGACGGAGGAGGCGTGGG    1500
NCPSR     TGGACGACAAGGCCAAGGACTTCGGCGAGCAGGCGAAGAAGGCGACGGAGGAGGCGTGGG    1500
NHRPSR    TGGACGACAAGGCCAAGGACTTCGGCGAGCAGGCGAAGAAGGCGACGGAGGAGGCGTGGG    1500
WUSR      TGGACGACAAGGCCAAGGACTTCGGCGAGCAGGCGAAGAAGGCGACGGAGGAGGCGTGGG    1500
JUSR      TGGACGACAAGGCCAAGGACTTCGGCGAGCAGGCGAAGAAGGCGACGGAGGAGGCGTGGG    1500
WWR       TGGACGACAAGGCCAAGGACTTCGGCGAGCAGGCGAAGAAGGCGACGGAGGAGGCGTGGG    1500
SWWR      TGGACGACAAGGCCAAGGACTTCGGCGAGCAGGCGAAGAAGGCGACGGAGGAGGCGTGGG    1500
IWVF      TGGACGACAAGGCCAAGGACTTCGGCGAGCAGGCGAAGAAGGCGACGGAGGAGGCGTGGG    1500
SWRNC     TGGACGACAAGGCCAAGGACTTCGGCGAGCAGGCGAAGAAGGCGACGGAGGAGGCGTGGG    1500
Spelta    TGGACGACAAGGCCAAGGACTTCGGCGAGCAGGCGAAGAAGGCGACGGAGGAGGCGTGGG    1500

WWRNC     ACGGCGCCAAGGACGCCGCACAGGGCATCACGGACAAGGTCGCCGCCGCGGCCAAAAAGG    1560
NCPSR     ACGGCGCCAAGGACGCCGCACAGGGCATCACGGACAAGGTCGCCGCCGCGGCCAAAAAGG    1560
NHRPSR    ACGGCGCCAAGGACGCCGCACAGGGCATCACGGACAAGGTCGCCGCCGCGGCCAAAAAGG    1560
WUSR      ACGGCGCCAAGGACGCCGCACAGGGCATCACGGACAAGGTCGCCGCCGCGGCCAAAAAGG    1560
JUSR      ACGGCGCCAAGGACGCCGCACAGGGCATCACGGACAAGGTCGCCGCCGCGGCCAAAAAGG    1560
WWR       ACGGCGCCAAGGACGCCGCACAGGGCATCACGGACAAGGTCGCCGCCGCGGCCAAAAAGG    1560
SWWR      ACGGCGCCAAGGACGCCGCACAGGGCATCACGGACAAGGTCGCCGCCGCGGCCAAAAAGG    1560
IWVF      ACGGCGCCAAGGACGCCGCACAGGGCATCACGGACAAGGTCGCCGCCGCGGCCAAAAAGG    1560
SWRNC     ACGGCGCCAAGGACGCCGCACAGGGCATCACGGACAAGGTCGCCGCCGCGGCCAAAAAGG    1560
Spelta    ACGGCGCCAAGGACGCCGCACAGGGCATCACGGACAAGGTCGCCGCCGCGGCCAAAAAGG    1560

WWRNC     AAGCTAGCTAAGCTAACACTACGTTGACTAGTCCGATCTGTATCTCTCAATTCATTTTCC    1620
NCPSR     AAGCTAGCTAAGCTAACACTACGTTGACTAGTCCGATCTGTATCTCTCAATTCATTTTCC    1620
NHRPSR    AAGCTAGCTAAGCTAACACTACGTTGACTAGTCCGATCTGTATCTCTCAATTCATTTTCC    1620
WUSR      AAGCTAGCTAAGCTAACACTACGTTGACTAGTCCGATCTGTATCTCTCAATTCATTTTCC    1620
JUSR      AAGCTAGCTAAGCTAACACTACGTTGACTAGTCCGATCTGTATCTCTCAATTCATTTTCC    1620
WWR       AAGCTAGCTAAGCTAACACTACGTTGACTAGTCCGATCTGTATCTCTCAATTCATTTTCC    1620
SWWR      AAGCTAGCTAAGCTAACACTACGTTGACTAGTCCGATCTGTATCTCTCAATTCATTTTCC    1620
IWVF      AAGCTAGCTAAGCTAACACTACGTTGACTAGTCCGATCTGTATCTCTCAATTCATTTTCC    1620
SWRNC     AAGCTAGCTAAGCTAACACTACGTTGACTAGTCCGATCTGTATCTCTCAATTCATTTTCC    1620
Spelta    AAGCTAGCTAAGCTAACACTACGTTGACTAGTCCGATCTGTATCTCTCAATTCATTTTCC    1620

WWRNC     ATTGTAAGAACGCATATACGTACTTTTGGTACAAAAGAAAAGAGATAAGATAGCTGTATT    1680
NCPSR     ATTGTAAGAACGCATATACGTACTTTTGGTACAAAAGAAAAGAGATAAGATAGCTGTATT    1680
NHRPSR    ATTGTAAGAACGCATATACGTACTTTTGGTACAAAAGAAAAGAGATAAGATAGCTGTATT    1680
WUSR      ATTGTAAGAACGCATATACGTACTTTTGGTACAAAAGAAAAGAGATAAGATAGCTGTATT    1680
JUSR      ATTGTAAGAACGCATATACGTACTTTTGGTACAAAAGAAAAGAGATAAGATAGCTGTATT    1680
WWR       ATTGTAAGAACGCATATACGTACTTTTGGTACAAAAGAAAAGAGATAAGATAGCTGTATT    1680
SWWR      ATTGTAAGAACGCATATACGTACTTTTGGTACAAAAGAAAAGAGATAAGATAGCTGTATT    1680
IWVF      ATTGTAAGAACGCATATACGTACTTTTGGTACAAAAGAAAAGAGATAAGATAGCTGTATT    1680
SWRNC     ATTGTAAGAACGCATATACGTACTTTTGGTACAAAAGAAAAGAGATAAGATAGCTGTATT    1680
Spelta    ATTGTAAGAACGCATATACGTACTTTTGGTACAAAAGAAAAGAGATAAGATAGCTGTATT    1680

WWRNC     TATTTCTGACATA                                                   1693
NCPSR     TATTTCTGACATA                                                   1693
NHRPSR    TATTTCTGACATA                                                   1693
WUSR      TATTTCTGACATA                                                   1693
JUSR      TATTTCTGACATA                                                   1693
WWR       TATTTCTGACATA                                                   1693
SWWR      TATTTCTGACATA                                                   1693
IWVF      TATTTCTGACATA                                                   1693
SWRNC     TATTTCTGACATA                                                   1693
Spelta    TATTTCTGACATA                                                   1693
```

Supplementary Fig. S10 Sequence alignments of the *Wcor15-2D* from hexaploid wheat with different geographical areas.

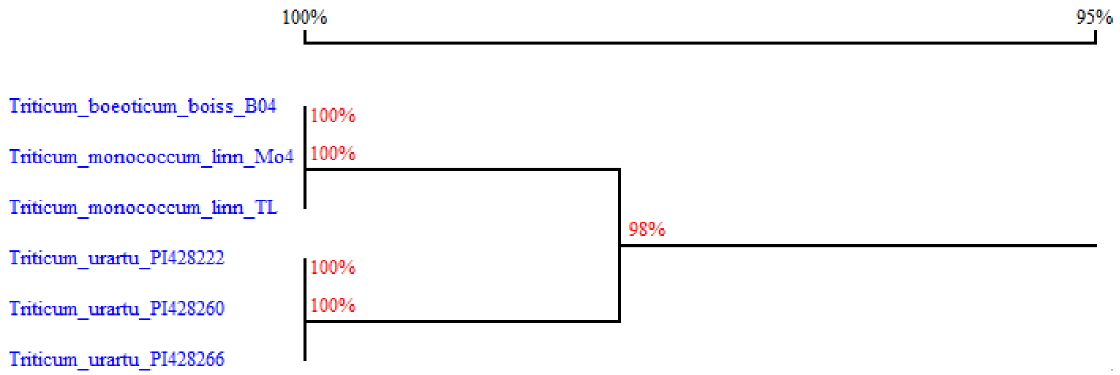

Supplementary Fig. S11 Cluster analysis of the *Wcor15-2A* from *T. urartu, T. monococcum* and *T. boeoticum*

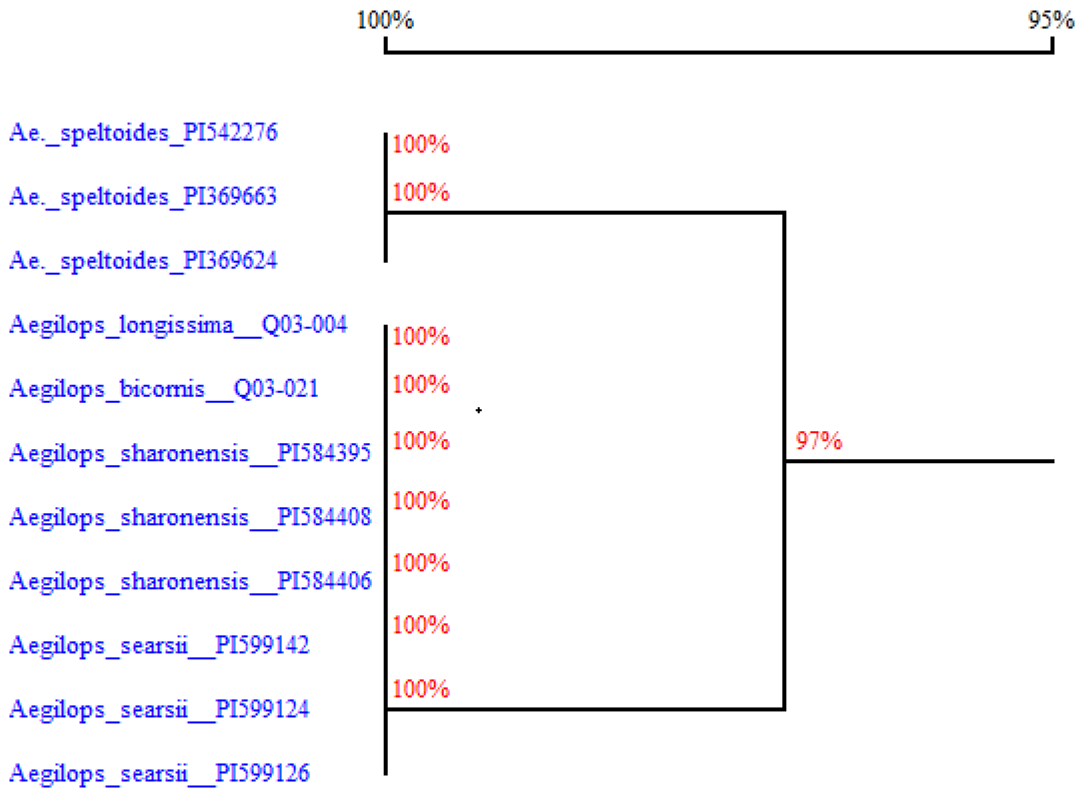

Supplementary Fig. S12 Cluster analysis of the *Wcor15-2B* from *the Sitopsis* section.

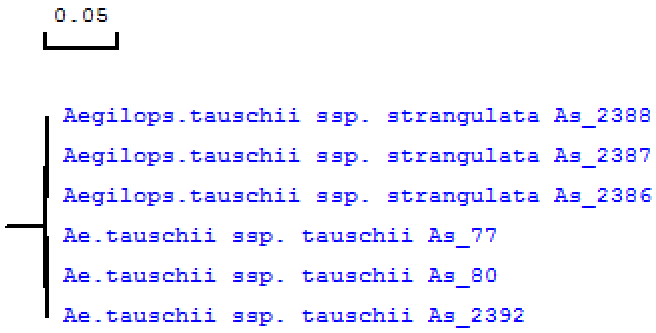

Supplementary Fig. S13 Cluster analysis of the *Wcor15-2D* from *Ae. tauschii*.

NO	Varieties	Accession			Ecotope / Type	NO	Varieties	Accession			Ecotope / Type
		Wcor15-2A	Wcor15-2B	Wcor15-2D				Wcor15-2A	Wcor15-2B	Wcor15-2D	
1	FARO	KT264839	KT264971	KT265036	IWVF	54	Annong 1106	KT264888	KT264958	KT265023	NHRPSR
2	TupatecoR	KT264840	KT264953	KT265037	IWVF	55	Annong 04156	KT264889	KT264984	—	NHRPSR
3	Niuzhijia	KT264841	—	—	IWVF	56	Aizao 64 xi	KT264890	KT264981	KT265025	NHRPSR
4	Xiaobaimai	KT264842	—	—	IWVF	57	Zhoumai 18	KT264891	KT264961	KT265026	NHRPSR
5	Jiangdongmen	KT264843	—	—	IWVF	58	Huaiyin 0454	KT264892	KT264985	—	NHRPSR
6	Dahuangpi	KT264844	—	—	IWVF	59	Zhou 98156	KT264893	KT264986	—	NHRPSR
7	Geerhongmai	KT264845	—	—	IWVF	60	Jun 99-7	KT264894	—	—	NHRPSR
8	Laoqimai	KT264846	—	—	IWVF	61	Luo 9908	KT264895	—	—	NHRPSR
9	Erandu	—	KT264960	—	IWVF	62	Zhengmai 9694	KT264896	—	—	NHRPSR
10	OLSB179	—	KT264966	—	IWVF	63	Wanmai 52	KT264897	—	—	NHRPSR
11	97001-68-1	—	KT264967	—	IWVF	64	Neixiang 203	KT264898	KT264987	—	NHRPSR
12	PH1521	—	KT264970	—	IWVF	65	Taikong 6 hao	KT264899	—	—	NHRPSR
13	MW18	—	KT264972	—	IWVF	66	Zhou 98165	KT264900	—	—	NHRPSR
14	Yannong 22	KT264847	KT264964	KT265029	JUSR	67	Caizhi 9998	KT264901	—	—	NHRPSR
15	Yannong 19	KT264848	—	—	JUSR	68	Xiangcheng 986	KT264902	—	—	NHRPSR
16	Denghai 5348	KT264849	KT264994	—	JUSR	69	Mianmai 39	KT264903	KT264969	KT265034	SWWR
17	Gaoyou 9409	KT264850	—	—	NCPSR	70	Neimai 9	KT264904	KT264990	KT265031	SWWR
18	Handan 6172	KT264851	—	—	NCPSR	71	Chuanmai 42（hong）	KT264905	KT264989	—	SWWR
19	Shannongyoumai 3 hao	KT264852	—	—	NCPSR	72	Chuannong 17	KT264906	KT264988	—	SWWR
20	Shannong 785	KT264853	—	—	NCPSR	73	Chuannong 42	KT264907	—	—	SWWR
21	Shi B02-5289	KT264854	—	—	NCPSR	74	Yunmai 51	KT264908	—	—	SWWR
22	Shi B03-5672	KT264855	KT264974	—	NCPSR	75	Yunmai 52	KT264909	—	—	SWWR

23	Xinmai 89019	KT264856	—	—	NCPSR	76	X9610	KT264910	KT264962	KT265027	WUSR
24	Liangxing 66	KT264857	KT264952	KT265017	NCPSR	77	Xinong 889	KT264911	—	—	WUSR
25	Changhe 25	KT264858	KT264973	KT265018	NCPSR	78	Xiaoyan 6 hao	KT264912	KT264963	KT265028	WUSR
26	Xinmai 18	KT264859	KT264956	KT265021	NCPSR	79	Xinong 4211	KT264913	—	—	WUSR
27	Xingmai 6 hao	KT264860	KT264955	KT265020	NCPSR	80	Xinong 4	KT264914	—	—	WUSR
28	Xinyuan 958	KT264861	—	—	NCPSR	81	Shannong 981	KT264915	—	—	WUSR
29	Xinmai 19023	KT264862	—	—	NCPSR	82	Shannong 138	KT264916	—	—	WUSR
30	Jinan 17	KT264863	KT264975	—	NCPSR	83	Ning 0076	KT264917	KT264991	—	WWR
31	Jimai 19	KT264864	KT264976	—	NCPSR	84	Ning 0078	KT264918	—	—	WWR
32	Jimai 22	KT264865	KT264954	KT265019	NCPSR	85	Annong 1116	KT264919	KT264965	KT265030	WWR
33	Jimai 21	KT264866	KT264977	—	NCPSR	86	Yangmai 16	KT264920	—	KT265035	WWR
34	Shijiazhuang 8 hao	KT264867	—	—	NCPSR	87	Annong 8455	KT264921	—	KT265032	WWR
35	Shijiazhuang 15	KT264868	—	—	NCPSR	88	Shengxuan 3 hao	KT264922	KT264968	KT265033	WWR
36	Shi 03-Y119	KT264869	—	—	NCPSR	89	Y14	KT264923	—	—	WWR
37	Xin 9944	KT264870	—	—	NCPSR	90	Y18	KT264924	—	—	WWR
38	Shannong B263	KT264871	KT264978	—	NCPSR	91	Ning 0310	KT264925	—	—	WWR
39	Shannong 3292	KT264872	KT264979	—	NCPSR	92	Ning 0564	KT264926	—	—	WWR
40	Shannong 5029	KT264873	KT264980	—	NCPSR	93	Emai 19	KT264927	—	—	WWR
41	Annong 9267	KT264874	—	—	NHRPSR	94	Henong 825	KT264880	KT264949	KT265014	WWRNC
42	Huaimai 20	KT264875	—	—	NHRPSR	95	Tai 10604	KT264928	KT264950	KT265015	WWRNC
43	Zhoumai 16	KT264876	—	—	NHRPSR	96	Zhongmai 1187	KT264929	KT264951	KT265016	WWRNC
44	Aikang 58	KT264877	—	—	NHRPSR	97	CP93-12-10-1-4	—	KT264993	—	WWRNC
45	Lian 9791	KT264878	—	—	NHRPSR	98	Xinchun6 hao	KT264930	KT264995	KT265038	SWRNC
46	Zhengmai 3666	KT264879	—	—	NHRPSR	99	Longmai 26	KT264931	KT264996	KT265039	SWRNC
47	Xuzhou 856	KT264881	KT264982	—	NHRPSR	100	Longmai 35	KT264932	KT264997	KT265040	SWRNC

48	Annong 0227	KT264882	KT264983	—	NHRPSR	101	Longmai 36	KT264933	KT264998	KT265041	SWRNC
49	Huaimai 0226	KT264883	—	—	NHRPSR	102	Avocets	KT264934	KT264999	KT265042	SWRNC
50	Xumai 270	KT264884	—	—	NHRPSR	103	*T.spelta* 1	KT264935	KT265000	KT265043	1
51	Annong 0822	KT264885	KT264957	KT265022	NHRPSR	104	*T.spelta* 6	KT264936	KT265001	KT265044	6
52	Bainong 64	KT264886	KT264959	KT265024	NHRPSR	105	*T.spelta* 7	KT264937	KT265002	KT265045	7
53	Huaimai 0208	KT264887	—	—	NHRPSR	106	*T.spelta* 11	KT264938	KT265003	KT265046	11

Supplementary Table S1. Geographic regions and relevant properties of tested 106 hexaploid wheat accessions. WWRNC: Winter wheat region of North China, NCPSR: North China plain sub-region of Yellow & Huai river winter wheat region, NHRPSR: North Huai river plain sub-region of Yellow & Huai river winter wheat region, WUSR: West upland sub-region of Yellow & Huai river winter wheat region, JUSR: Jiaodong upland sub-region of Yellow & Huai river winter wheat region, WWR: Winter wheat region of middle and lower reaches of the Yangtze river, SWWR: Southwestern winter wheat region, SWRNC: Spring wheat region of North China, IWVF: Introduced wheat variety of foreign. "—" represents that the wheat DNA sample was only amplified with appropriate size using corresponding primer but not sequenced.

No	Varieties	Accession	Identity (%)	No	Varieties	Accession	Identity (%)
1	FARO	KT264839	100	51	Aizao 64 xi	KT264890	100
2	TupatecoR	KT264840	100	52	Zhoumai 18	KT264891	100
3	Niuzhijia	KT264841	100	53	Huaiyin 0454	KT264892	100
4	Xiaobaimai	KT264842	100	54	Zhou 98156	KT264893	100
5	Jiangdongmen	KT264843	100	55	Jun 99-7	KT264894	100
6	Dahuangpi	KT264844	100	56	Luo 9908	KT264895	100
7	Geerhongmai	KT264845	100	57	Zhengmai 9694	KT264896	100
8	Laoqimai	KT264846	100	58	Wanmai 52	KT264897	100
9	Yannong 22	KT264847	100	59	Neixiang 203	KT264898	100
10	Yannong 19	KT264848	100	60	Taikong 6 hao	KT264899	100
11	Denghai 5348	KT264849	100	61	Zhou 98165	KT264900	100
12	Gaoyou 9409	KT264850	100	62	Caizhi 9998	KT264901	100
13	Handan 6172	KT264851	100	63	Xiangcheng 986	KT264902	100
14	Shannongyoumai 3 hao	KT264852	100	64	Mianmai 39	KT264903	100
15	Shannong 785	KT264853	100	65	Neimai 9	KT264904	100
16	Shi B02-5289	KT264854	100	66	Chuanmai 42（hong）	KT264905	100
17	Shi B03-5672	KT264855	100	67	Chuannong 17	KT264906	100
18	Xinmai 89019	KT264856	100	68	Chuannong 42	KT264907	100
19	Liangxing 66	KT264857	100	69	Yunmai 51	KT264908	100
20	Changhe 25	KT264858	100	70	Yunmai 52	KT264909	100
21	Xinmai 18	KT264859	100	71	X9610	KT264910	100
22	Xingmai 6 hao	KT264860	100	72	Xinong 889	KT264911	100
23	Xinyuan 958	KT264861	100	73	Xiaoyan 6 hao	KT264912	100
24	Xinmai 19023	KT264862	100	74	Xinong 4211	KT264913	100
25	Jinan 17	KT264863	100	75	Xinong 4	KT264914	100
26	Jimai 19	KT264864	100	76	Shannong 981	KT264915	100
27	Jimai 22	KT264865	100	77	Shannong 138	KT264916	100
28	Jimai 21	KT264866	100	78	Ning 0076	KT264917	100
29	Shijiazhuang 8 hao	KT264867	100	79	Ning 0078	KT264918	100
30	Shijiazhuang 15	KT264868	100	80	Annong 1116	KT264919	100
31	Shi 03-Y119	KT264869	100	81	Yangmai 16	KT264920	100
32	Xin 9944	KT264870	100	82	Annong 8455	KT264921	100

33	Shannong B263	KT264871	100	83	Shengxuan 3 hao	KT264922	100
34	Shannong 3292	KT264872	100	84	Y14	KT264923	100
35	Shannong 5029	KT264873	100	85	Y18	KT264924	100
36	Annong 9267	KT264874	100	86	Ning 0310	KT264925	100
37	Huaimai 20	KT264875	100	87	Ning 0564	KT264926	100
38	Zhoumai 16	KT264876	100	88	Emai 19	KT264927	100
39	Aikang 58	KT264877	100	89	Henong 825	KT264880	100
40	Lian 9791	KT264878	100	90	Tai 10604	KT264928	100
41	Zhengmai 3666	KT264879	100	91	Zhongmai 1187	KT264929	100
42	Xuzhou 856	KT264881	100	92	Xinchun6 hao	KT264930	100
43	Annong 0227	KT264882	100	93	Longmai 26	KT264931	100
44	Huaimai 0226	KT264883	100	94	Longmai 35	KT264932	100
45	Xumai 270	KT264884	100	95	Longmai 36	KT264933	100
46	Annong 0822	KT264885	100	96	Avocets	KT264934	100
47	Bainong 64	KT264886	100	97	*T.spelta* 1	KT264935	100
48	Huaimai 0208	KT264887	100	98	*T.spelta* 6	KT264936	100
49	Annong 1106	KT264888	100	99	*T.spelta* 7	KT264937	100
50	Annong 04156	KT264889	100	100	*T.spelta* 11	KT264938	100

Supplementary Table S2. Homology analysis of 99 Wcor15-2A sequences with the sequence of Annong 0822 in hexaploid wheats by BLAST (https: // blast. ncbi. nlm. nih. gov/ Blast .cgi).

No	Varieties	Accession	Identity (%)	No	Varieties	Accession	Identity (%)
1	FARO	KT264971	100	28	Aizao 64 xi	KT264981	99
2	TupatecoR	KT264953	99	29	Zhoumai 18	KT264961	99
3	Erandu	KT264960	99	30	Huaiyin 0454	KT264985	100
4	OLSB179	KT264966	100	31	Zhou 98156	KT264986	99
5	97001-68-1	KT264967	99	32	Neixiang 203	KT264987	99
6	PH1521	KT264970	99	33	Mianmai 39	KT264969	99
7	MW18	KT264972	100	34	Neimai 9	KT264990	99
8	Yannong 22	KT264964	100	35	Chuanmai 42（hong）	KT264989	100
9	Denghai 5348	KT264994	100	36	Chuannong 17	KT264988	100
10	Shi B03-5672	KT264974	100	37	X9610	KT264962	99
11	Liangxing 66	KT264952	100	38	Xiaoyan 6 hao	KT264963	99
12	Changhe 25	KT264973	100	39	Ning 0076	KT264991	99
13	Xinmai 18	KT264956	99	40	Annong 1116	KT264965	99
14	Xingmai 6 hao	KT264955	100	41	Shengxuan 3 hao	KT264968	100
15	Jinan 17	KT264975	100	42	Henong 825	KT264949	99
16	Jimai 19	KT264976	100	43	Tai 10604	KT264950	99
17	Jimai 22	KT264954	99	44	Zhongmai 1187	KT264951	100
18	Jimai 21	KT264977	100	45	CP93-12-10-1-4	KT264993	99
19	Shannong B263	KT264978	100	46	Xinchun6 hao	KT264995	100
20	Shannong 3292	KT264979	100	47	Longmai 26	KT264996	100
21	Shannong 5029	KT264980	100	48	Longmai 35	KT264997	100
22	Xuzhou 856	KT264982	100	49	Longmai 36	KT264998	99
23	Annong 0227	KT264983	99	50	Avocets	KT264999	100
24	Annong 0822	KT264957	100	51	*T.spelta* 1	KT265000	99
25	Bainong 64	KT264959	99	52	*T.spelta* 6	KT265001	99
26	Annong 1106	KT264958	100	53	*T.spelta* 7	KT265002	99
27	Annong 04156	KT264984	100	54	*T.spelta* 11	KT265003	99

Supplementary Table S3. Homology analysis of 53 Wcor15-2B sequences with the sequence of Annong 0822 in hexaploid wheats by BLAST (https: // blast. ncbi. nlm. nih. Gov / Blast.cgi).

No	Varieties	Accession	Identity (%)	No	Varieties	Accession	Identity (%)
1	FARO	KT265036	100	18	Annong 1116	KT265030	100
2	TupatecoR	KT265037	100	19	Yangmai 16	KT265035	100
3	Yannong 22	KT265029	100	20	Annong 8455	KT265032	100
4	Liangxing 66	KT265017	100	21	Shengxuan 3 hao	KT265033	100
5	Changhe 25	KT265018	100	22	Henong 825	KT265014	100
6	Xinmai 18	KT265021	100	23	Tai 10604	KT265015	100
7	Xingmai 6 hao	KT265020	100	24	Zhongmai 1187	KT265016	100
8	Jimai 22	KT265019	100	25	Xinchun6 hao	KT265038	100
9	Annong 0822	KT265022	100	26	Longmai 26	KT265039	100
10	Bainong 64	KT265024	100	27	Longmai 35	KT265040	100
11	Annong 1106	KT265023	100	28	Longmai 36	KT265041	100
12	Aizao 64 xi	KT265025	100	29	Avocets	KT265042	100
13	Zhoumai 18	KT265026	100	30	*T.spelta* 1	KT265043	100
14	Mianmai 39	KT265034	100	31	*T.spelta* 6	KT265044	100
15	Neimai 9	KT265031	100	32	*T.spelta* 7	KT265045	100
16	X9610	KT265027	100	33	*T.spelta* 11	KT265046	100
17	Xiaoyan 6 hao	KT265028	100				

Supplementary Table S4. Homology analysis of 32 Wcor15-2D sequences with the sequence of Annong 0822 in hexaploid wheats by BLAST (https: // blast. ncbi. nlm. nih. Gov / Blast.cgi).

DNA sequence	Wcor15-2A1 (*T. urartu*)	Wcor15-2A2 (*T. monococcum* and *T. boeoticum*)	Wcor15-2A (tetraploid)	Wcor15-2A (hexaploid)
Wcor15-2A1 (*T. urartu*)	100%	97.87%	100%	100%
Wcor15-2A2 (*T. monococcum* and *T. boeoticum*)	97.87%	100%	97.87%	97.87%
Wcor15-2A (tetraploid)	100%	97.87%	100%	100%
Wcor15-2A (hexaploid)	100%	97.87%	100%	100%

Supplementary Table S5. Homology analysis of the Wcor15-2A DNA sequences in the diploid, tetraploid and hexaploid species.

DNA sequence	Wcor15-2D (As2386)	Wcor15-2D (As2387)	Wcor15-2D (As2388)	Wcor15-2D (As80)	Wcor15-2D (As77)	Wcor15-2D (As2392)	Wcor15-2D (hexaploid)
Wcor15-2D (As2386)	100%	100%	100%	99%	99%	99%	100%
Wcor15-2D (As2387)	100%	100%	100%	99%	99%	99%	100%
Wcor15-2D (As2388)	100%	100%	100%	99%	99%	99%	100%
Wcor15-2D (As80)	99%	99%	99%	100%	100%	99%	99%
Wcor15-2D (As77)	99%	99%	99%	100%	100%	99%	99%
Wcor15-2D (As2392)	99%	99%	99%	99%	99%	99%	99%
Wcor15-2D (hexaploid)	100%	100%	100%	99%	99%	99%	100%

Supplementary Table S6. Homology analysis of the Wcor15-2D DNA sequences in the diploid and hexaploid species.
